# Infiltration of Apoptotic M2 Macrophage Subpopulation Is Negatively Correlated with the Immunotherapy Response in Colorectal Cancer

**DOI:** 10.3390/ijms231911014

**Published:** 2022-09-20

**Authors:** Rui Liu, Chongyin Han, Jiaqi Hu, Baowen Zhang, Wei Luo, Fei Ling

**Affiliations:** 1 Guangdong Key Laboratory of Fermentation and Enzyme Engineering, School of Biology and Biological Engineering, South China University of Technology, Guangzhou 510006, China; 2 The First People’s Hospital of Foshan, Sun Yat-sen University, Foshan 528000, China

**Keywords:** colorectal cancer, tumor-associated macrophages, polarization, apoptosis, S100A6, immunotherapy

## Abstract

The polarization of tumor-associated macrophages (TAMs) plays a key role in tumor development and immunotherapy in colorectal cancer (CRC) patients. However, the impact of apoptosis on TAM polarization and immunotherapy efficacy in patients with different mismatch repair statuses (MMR) remains unclear. Here, we constructed an atlas of macrophage and found a higher rate of infiltration of M2-like TAM subpopulation in pMMR CRC tumor tissues compared with that in dMMR CRC tumor tissues. Importantly, a lower infiltration rate of M2c-like TAMs was associated with immunotherapy response. The M2 polarization trajectory revealed the apoptosis of M2c-like TAMs in dMMR while the differentiation of M2c-like TAMs in pMMR, implying a higher polarization level of M2 in pMMR. Furthermore, we found that a high expression of S100A6 induces the apoptosis of M2c-like TAMs in dMMR. In conclusion, we identified apoptotic TAM subpopulations in the M2 polarization trajectory and found that apoptosis caused by the high expression of S100A6 reduces their infiltration in tumors as well as the level of M2 polarization and contributes to a favorable immunotherapy response. These findings provide new insights into the potential role of apoptosis in suppressing tumors and enhancing immunotherapeutic efficacy.

## 1. Introduction

Colorectal cancer (CRC) shows a large dynamic range of immune responsiveness, with a striking difference between two genetically distinct subtypes: patients with deficient mismatch repair (dMMR) have a high neoantigen burden and a high survival rate and respond to immunotherapy, associated with the decreased infiltration of tumor-promoting macrophages [1,2]. In contrast, patients with proficient mismatch repair (pMMR) have the opposite characteristics [1]. However, how the infiltration of macrophages leads to distinct outcomes in CRC patients with different MMR statuses needs to be elucidated.

Tumor-associated macrophages (TAMs) are the essential components of the innate immune system, and they play a crucial role in tumor development, invasion, and metastasis [3]. The polarization of TAMs profoundly affects tumor development [4]. The “classically activated” M1 and the “alternatively activated” M2 macrophage polarization system have been used to describe the in vitro activation state of TAMs [5]. However, due to the complexity of the tumor immune microenvironment (TME), TAMs exhibit more phenotypes, i.e., a “spectrum” model, in vivo [6]. Numerous studies have shown that although the M2 polarization of TAMs promotes tumor growth and metastasis [7], the apoptosis of TAMs can inhibit tumor growth and improve patients’ anti-tumor immunity [8]. However, the current studies on the development trajectory of TAM polarization toward the M2 phenotype and the effect of apoptosis on M2 polarization are unclear, and it is necessary to construct a well-defined trajectory of M2 polarization and identify intermediate-state cell types in combination with the phenotypic diversity of TAMs.

TAMs are a key component of the immunosuppressive pathways targeted by immune checkpoint inhibitors and, therefore, may provide tools to predict or increase the activity of such treatments [4]. For example, TAMs can express the *PD‐1* ligands *PD‐L1*, as well as the *CTLA‐4* ligands *B7‐1* (*CD80*) and *B7‐2* (*CD86*) [9], which inhibit the phagocytosis of TAMs [10]. How the expression of *PDL1* (and/or *PDL2*) and *CTLA‐4* ligands on TAMs has not been fully elucidated [4]. Therefore, the expression of these triggers of inhibitory immune checkpoints by TAMs needs to be carefully assessed, and the factors influencing the efficacy of immunotherapy by TAMs must be investigated.

In this study, we analyzed the diversity of macrophages in the tumor tissues of dMMR and pMMR patients at the single-cell resolution level and constructed the M2 polarization trajectory of TAMs. The trajectory revealed that S100A6-induced apoptosis decreases the level of TAM polarization. Finally, we explored the impact of the infiltration of immunosuppressive TAMs on immunotherapy efficacy.

## 2. Results

### 2.1. Higher Infiltration of the M2-like TAM Subpopulation in pMMR CRC Tumor Tissues Compared with That in dMMR CRC Tumor Tissues

**Figure 1:**
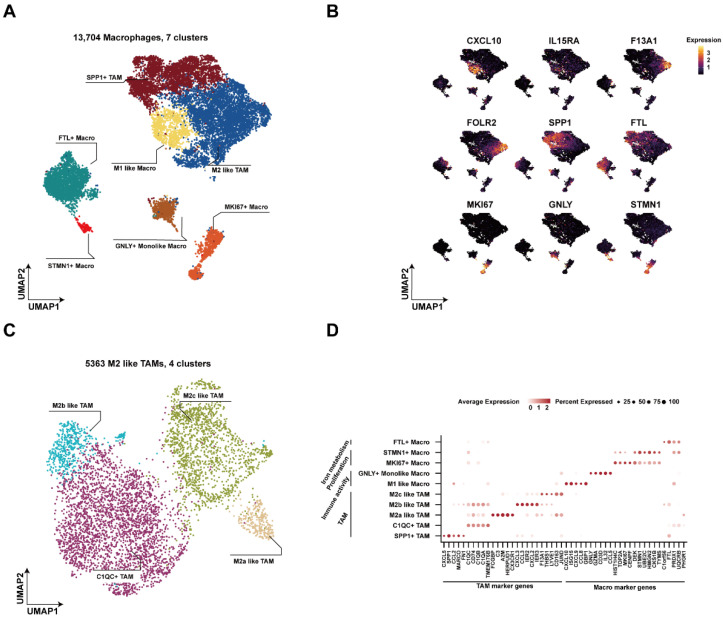
The atlas of macrophages in deficient mismatch repair (dMMR) and proficient mismatch repair (pMMR) tumors: (**A**) a UMAP clustering of macrophages (*n* = 13,704) from single-cell RNA sequencing (scRNA-seq) of CRC patients shows seven clusters; (**B**) expression of marker genes in seven clusters of macrophages; (**C**) a UMAP clustering of M2-like tumor-associated macrophages (TAMs) (*n* = 5363) showing four clusters; (**D**) the dot plot shows the expression of the top five marker genes in all subpopulations of macrophages.

To characterize macrophage heterogeneity within the tumor immune microenvironment (TIME) and investigate the various cell types, gene expression patterns, and functions of TAMs, Uniform Manifold Approximation and Projection (UMAP) was performed on macrophages, and 13,704 macrophages were divided into 8 clusters by initial clustering based on representative markers (Figure 1A and Table S1). Specifically, SPP1+ TAMs overexpressed the classical markers *SPP1* and *MARCO* (Figure 1B,D) [11]; MKI67+ Macros and STMN1+ Macros were proliferation-associated macrophages with a high expression of the *MKI67*, *TOP2A*, and *STMN1* (Figure 1B,D) [12,13]. M1-like Macros and M2-like TAMs were identified based on marker genes (*CXCL10*, *IL15RA*, *F13A1*, and *FOLR2*), respectively [14,15,16]. In addition, we identified GNLY+ Monolike Macros as a unique cluster of macrophages that had cytotoxicity-related gene expression similar to that of CD3E+ monocytes from a previous study [17].

TAMs that typically had the M2 phenotype exhibited higher heterogeneity and were associated with multiple functions that promote tumor progression [18,19]. Therefore, we extracted M2-like TAMs for further subgrouping and obtained four distinct subgroups (Figure 1C and Figure S1B). C1QC+ TAMs expressed the complement components *C1QC*, *C1QB*, and C1QA, confirming the findings of prior research [11]. M2a-like TAMs, M2b-like TAMs, and M2c-like TAMs highly expressed unique marker genes, respectively (Figure S1C) [20,21]. Overall, different cell subtypes were widely distributed in dMMR and pMMR tumor tissues with distinct gene expression patterns (Figure 1D and Figure S1A), and the diversity of macrophage infiltration and function needs additional investigation.

**Figure 2:**
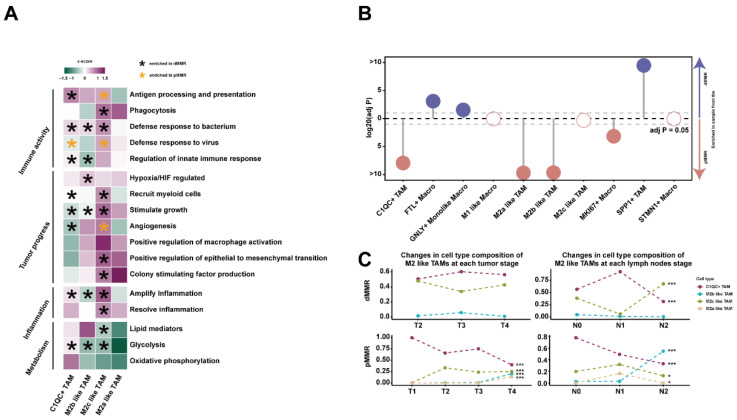
Analysis of macrophage functions and cell ratios: (**A**) the heatmap shows the Gene Set Variation Analysis (GSVA) z-score of the functional activity of M2-like TAMs. These functions were classified into four categories according to the different gene set characteristics. The green color indicates that the activity of this function or pathway is upregulated in TAMs, and the purple color indicates that the activity of this function or pathway is downregulated in TAMs. The asterisk indicates that one function is significantly upregulated in dMMR or pMMR; (**B**) the lollipop plot shows the infiltration of 10 clusters of macrophages in dMMR and pMMR. Solid circles indicate that this cluster of macrophages is significantly enriched in dMMR or pMMR (chi-square test, *p* < 0.05), and hollow circles indicate no significant difference in cell ratios between dMMR and pMMR (*p* > 0.05); (**C**) changes in the cell ratios of M2-like TAMs in dMMR and pMMR at different tumor stages (T1, T2, T3, and T4) and lymph node stages (N0, N1, and N2), *** *p* < 0.001, and * *p* < 0.05.

To assess the functional activity of different cell types, we collected macrophage signature gene sets from published studies and performed Gene Set Variation Analysis (GSVA) (Table S2). M2-like TAMs displayed unique tumor progression or immunomodulation-related capabilities. C1QC+ TAMs exhibited increased immune activity and decreased tumor progression-related functions, indicating that they do not promote tumors (Figure 2A). C1QC+ TAMs showed the strongest antigen processing and presenting capacity, and this function was significantly upregulated in dMMR. However, the activity of antigen processing and presentation function was higher in M2c-like TAMs in pMMR than in dMMR, implying significant differences in the partial immune function activity of TAMs in different tissues. M2b-like TAMs promoted angiogenesis and positively regulated epithelial-mesenchymal transition (EMT), indicating their pro-tumor potential (Figure 2A). Similarly, M2c-like TAMs and M2a-like TAMs also functioned as a “gas pedal” for tumor development. Moreover, M2-like TAMs had different levels of metabolic signaling intensity and inflammatory regulation ability, and most of the functions related to tumor progression and immune activity were upregulated in dMMR (Figure 2A). In addition, SPP1+ TAMs had the strongest pro-angiogenesis function, while STMN1+ Macros and FTL+ Macros exhibited low overall function activity, suggesting that they might be resting macrophages (Figure S1D).

It has been demonstrated that the MMR status of patients affects macrophage infiltration [2,22]. Meanwhile, the enrichment of TAMs promotes tumor progression and metastasis in the TME [23]. We compared the abundance of all macrophages in dMMR and pMMR and found that the cell proportion of most subpopulations was considerably different. Except for SPP1+TAMs, all TAMs associated with tumor progression were enriched in dMMR, while GNLY+ Monolike Macros with the anti-tumor immune function were abundant in pMMR (Figure 2B). The infiltration of macrophages was also associated with the tumor and lymph node stage in patients. In this study, we focused on M2-like TAMs. The proportion of M2 b-like TAMs in pMMR increased with tumor progression whereas the proportion of immune-activated C1QC+ TAMs decreased (Figure 2C). Similar trends of changing cell ratio were not found in dMMR, indicating greater fluctuations in cell proportion with tumor progression. Surprisingly, during cancer progression, M2a-like TAMs were only present in pMMR patients but not in dMMR patients (Figure 2C), and the underlying causes for this phenomenon require further investigation.

### 2.2. Apoptosis of M2c-like TAMs in dMMR While Differentiation of M2c-like TAMs in pMMR

**Figure 3:**
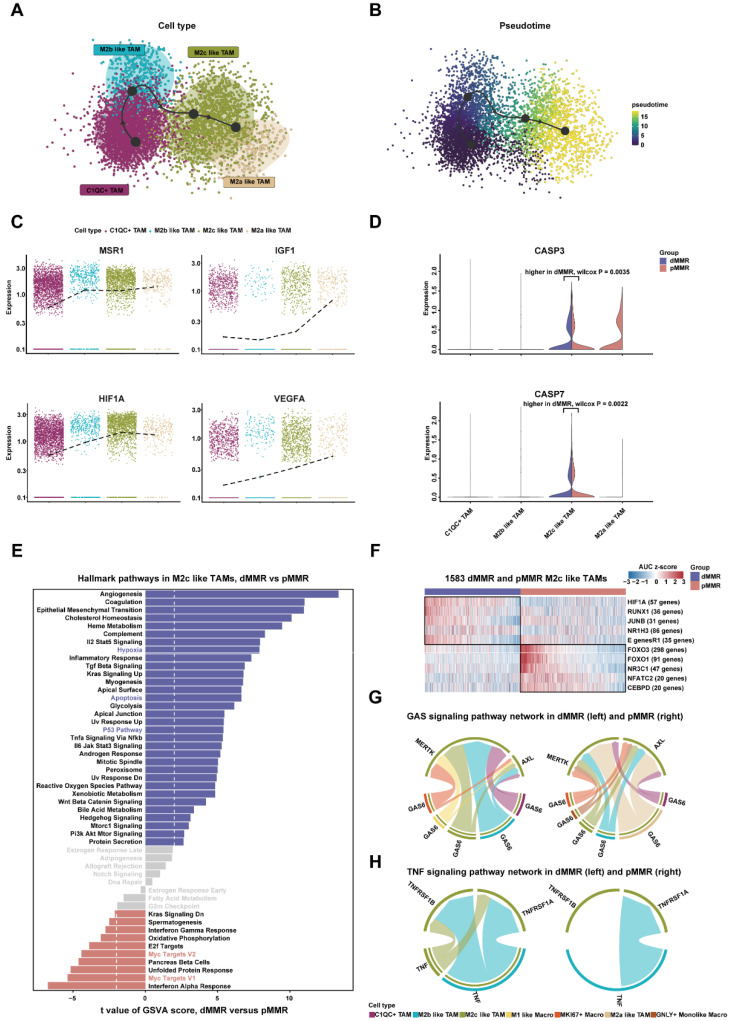
Pseudotime trajectories reveal that apoptosis results in different differentiation fates of M2c-like TAMs: (**A**) pseudotime differentiation trajectory of M2-like TAMs; (**B**) pseudotime shows the direction of differentiation from C1QC+ TAMs to M2a-like TAMs; (**C**) changes in the expression levels of MSR1, IGF1, HIF1A, and VEGFA with cell differentiation. The black dash indicates the average gene expression; (**D**) expression of apoptosis effector genes in subpopulations of M2-like TAMs. The Wilcox test was used to compare expression differences in dMMR and pMMR. *p* < 0.05 is statistically significant; (**E**) differences in hallmark pathway activity scores of M2c-like TAMs between dMMR and pMMR (*n* = 754 and 829, respectively). Proliferation-related pathways are marked in red, and apoptosis-related pathways are marked in blue. T values are from limma; (**F**) the heatmap shows the transcription factor regulatory activity (AUC z-score) obtained from the Single-Cell Regulatory Network Inference and Clustering (SCENIC) analysis. The top five TFs with upregulated transcriptional activity were selected for visualization in dMMR and pMMR, respectively. Limma was used to compare the differences in AUCs between dMMR and pMMR; (**G**) communication among macrophages via the GAS signaling pathway, which promotes cell proliferation; (**H**) communication among macrophages via the TNF signaling pathway, which promotes apoptosis.

To better portray the dynamic changes in the cell types and expression characteristics of M2-like TAMs, we inferred the polarization trajectory of four subpopulations of M2-like TAMs. The C1QC+ TAMs were the origin of the M2 polarization trajectory, which went through the intermediate states of M2b-like TAMs and M2c-like TAMs before eventually differentiating into M2a-like TAMs (Figure 3A,B). *IGF1* and *MSR1* were M2 polarization markers, and their progressively higher expression with polarization indicated that the cells were increasingly characterized by M2 polarization (Figure 3C).

The M2 polarization trajectory terminated at M2a-like TAMs, demonstrating that M2c-like TAMs in dMMR could not successfully differentiate into M2a-like TAMs. It was found that apoptosis inhibited the normal differentiation of M2c-like TAMs. *CASP3* and *CASP7* were significantly overexpressed in M2c-like TAMs in dMMR (Figure 3D), implying a higher apoptotic effect in this cluster of cells. According to the GSVA analysis of hallmark gene sets, M2c-like TAMs exhibited different pathway activities in dMMR and pMMR (Figure 3E). The apoptosis pathway was significantly enriched in dMMR. Meanwhile, the *P53* signaling pathway and the hypoxia pathway, both pro-apoptosis pathways, had upregulated activities in dMMR as well (Figure 3E). *MYC* signaling pathways were enriched in pMMR and associated with the continuing differentiation of M2c-like TAMs in pMMR (Figure 3E).

Furthermore, the SCENIC results showed that *HIF1A*, with the largest variance, and *RUNX1* as candidate transcription factors (TFs) underlie the differences in gene expression in M2c-like TAMs between dMMR and pMMR (Figure 3F). HIF1A is a hypoxia-inducible factor that induces apoptosis via the *P53* pathway, and *RUNX1* is also an apoptosis-promoting TF. Interestingly, *HIF1A* is linked to *P53* expression in tumors [24], and other genes involved in apoptosis also show putative *HIF1A*/*RUNX1* binding sites [25]. These suggest that the upregulation of their activities in dMMR is responsible for the apoptosis of M2c-like TAMs. In contrast, the upregulation of *FOXO1* and *FOXO3* are associated with proliferation, which might explain the continuing differentiation of M2c-like TAMs in pMMR [26].

Finally, we explored whether cell-to-cell communication across all macrophages affects the differentiation endpoints of particular cells. *GAS6* promotes cell proliferation and inhibits apoptosis by binding to its receptors *MERTK* and *AXL* [27]. We found more types of cells involved in the *GAS* signaling pathway network in pMMR, along with a higher communication probability and upregulation of LR pair expression in pMMR (Figure 3G and Figure S2A,B,F). Specifically, M2a-like TAMs transmitted the strongest proliferation signal to M2c-like TAMs in pMMR. *TNF* involves extrinsic apoptosis by specifically binding with its receptor to form a death domain [28], which might “accelerate” the cell death of M2c-like TAMs in dMMR. The *TNF* signaling pathway network was enhanced in dMMR with higher expression of LR pairs (Figure S2E). As the sender of the *TNF* signaling pathway, M2 b-like TAMs conveyed a potent apoptosis signal to M2c-like TAMs (Figure 3H and Figure S2C,D).In conclusion, we suggested that the increased apoptotic effect of M2c-like TAMs in dMMR might contribute to their different cell fates.

### 2.3. High Expression of S100A6 Induced Apoptosis of M2c-like TAMs in dMMR

**Figure 4:**
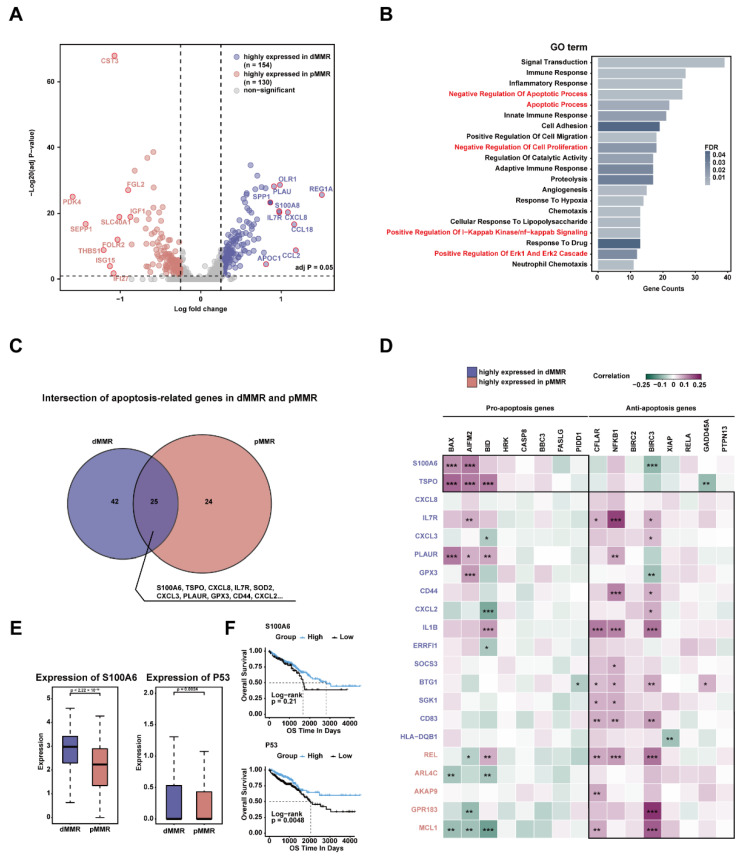
Screening for genes associated with apoptosis of M2c-like TAMs in dMMR: (**A**) a volcano plot shows differentially expressed genes of M2c-like TAMs in dMMR and pMMR. The top 10 differentially expressed genes (DEGs) upregulated in dMMR and pMMR are labeled; (**B**) the top 20 GO terms significantly enriched by the 284 DEGs (FDR < 0.05). Pathways associated with proliferation or apoptosis are marked in red; (**C**) the intersection of DEGs obtained by lasso screening related to dMMR and pMMR apoptosis scores; (**D**) the heatmap shows the Pearson correlation between DEGs and apoptosis-related genes. The significance level of the correlation is indicated by asterisks: *** *p* < 0.001, ** *p* < 0.01, and * *p* < 0.05; (**E**) expression of S100A6 and P53 in dMMR and pMMR. Differences in expression are checked using the Wilcox test; (**F**) survival analysis of S100A6 and P53 in TCGA-CRC data. Log-rank test.

To explore the genes leading to the apoptosis of M2c-like TAMs in dMMR, we performed differential analysis and identified a total of 284 differentially expressed genes (DEGs) (Table S3). Of these, 154 DEGs were significantly upregulated in dMMR, and 130 DEGs were significantly upregulated in pMMR (Figure 4A). They were found to be enriched in some pathways related to apoptosis or proliferation, such as the apoptotic process, the positive regulation of I-kappaB kinase/NF-kappaB signaling, and the positive regulation of *ERK1* and *ERK2* cascade (Figure 4B). Next, we used the Least Absolute Shrinkage and Selection Operator (LASSO) algorithm to further screen DEGs related to apoptosis (Figure S4A). A total of 25 genes were associated with the apoptotic effect of M2c-like TAMs in both dMMR and pMMR (Figure 4C). These genes were used for further analysis. Subsequently, we selected 16 apoptosis-related genes with known functions (8 pro-apoptosis and 8 anti-apoptosis) and analyzed correlations among genes to identify the potential functions of DEGs. The few DEGs with unclear apoptotic effects were removed, and a total of 21 genes were found to show a higher correlation with apoptosis-related genes, which we referred to as apoptosis-related DEGs (Figure 4D). The expression of *S100A6* and *TSPO* was positively correlated with most of the pro-apoptosis genes and negatively correlated with most of the anti-apoptosis genes, indicating that these genes may have pro-apoptosis effects (Figure 4D). The remaining DEGs displayed opposite gene expression relationship patterns, implying their crucial role in apoptosis inhibition. Among them, *CXCL8* is mainly secreted by macrophages and contributes to the immunosuppressive microenvironment [29]. *CXCL3* is also a chemokine secreted by macrophages and associated with the invasion and metastasis of various malignancies [30]. *Rel* is critical for the NF-kappa B-dependent inhibition of apoptosis [31]. *MCL1* is an essential member of the *BCL2* family that can promote macrophage survival [32]. Their upregulated expression might be a potential factor in suppressing the apoptosis of M2c-like TAMs in pMMR.

We proposed that the pro-apoptosis-related DEGs upregulated in dMMR and the anti-apoptosis-related DEGs upregulated in pMMR are the hub genes responsible for the differential apoptotic effects of M2c-like TAMs. Therefore, seven hub genes were finally identified. Among these, *S100A6* and *TSPO* were upregulated in dMMR (Figure 4E and Figure S3B), and *REL*, *ARL4C*, *AKAP9*, *GPR183*, and *MCL1* were upregulated in pMMR (Figure S3B), which might have the largest impact on apoptosis in M2c-like TAMs. The gene with the largest fold change was *S100A6*, and it could upregulate the expression of the apoptosis-inducing gene *P53* to promote apoptosis [33]. In our study, the expression of *P53* was also found to be upregulated in dMMR (Figure 4E). In addition, *P53*, not *S100A6*, was associated with survival in CRC patients (Figure 4F). An examination of the bulk RNA-seq data showed that the expression of *S100A6* and *P53* was higher in dMMR patients than in pMMR patients (Figure S3C). These results suggest that overexpression of *S100A6* might upregulate the expression of *P53* and promote apoptosis of M2c-like TAMs in dMMR.

### 2.4. Lower Infiltration of M2c-like TAMs Was Associated with Immunotherapy Response

**Figure 5:**
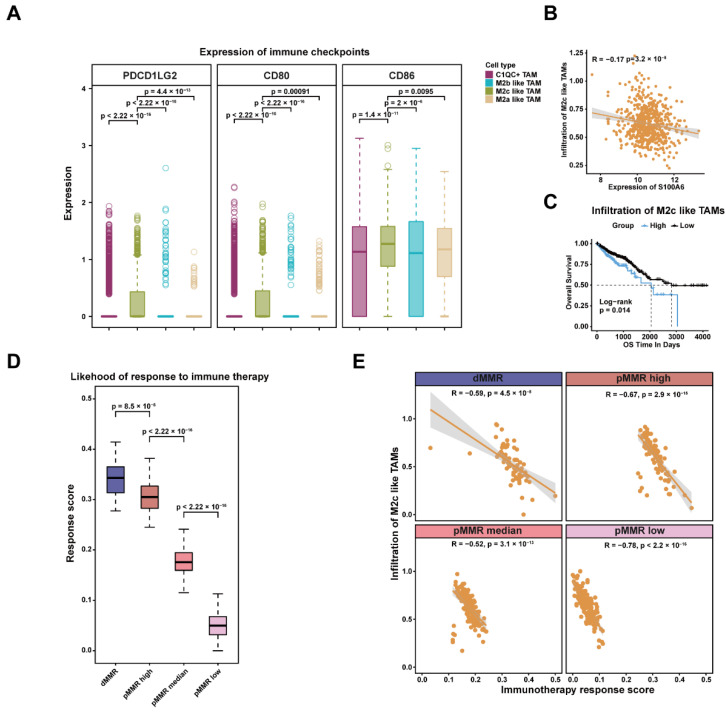
Impact of M2c-like TAMs on immunotherapy: (**A**) expression of immune checkpoints in M2-like TAMs. The Wilcox test was used to examine differences in the expression of the other three clusters of macrophages with M2c-like TAMs; (**B**) the correlation between the infiltration of M2c-like TAMs and the expression of S100A6; (**C**) survival analysis of the infiltration of M2c-like TAMs. Log-rank test; (**D**) immunotherapy response scores obtained by EaSIeR in four groups of CRC patients. The differences in response scores among different groups of patients were examined by the Wilcox test; (**E**) Pearson correlation between the infiltration of M2c-like TAMs and immunotherapy response scores.

The impact of M2c-like TAMs, a cluster of cells with tumor-promoting potential, on immunotherapy response required further evaluation. We examined the expression of reported immune checkpoint molecules on the surface of macrophages and found that M2c-like TAMs highly expressed *CTLA* ligands *CD80* and *CD86*, as well as the *PD1* ligand *PDCD1LG2*, indicating a higher immunosuppressive profile (Figure 5A). *PDCD1LG2* was also found to be highly expressed in M2c-like TAMs in dMMR, which represented the discrepancy in the immunosuppressive characteristics of TAMs in different patients (Figure S4A).To assess the level of infiltration, the top30 marker genes of M2c-like TAMs were used as signature gene sets. We found that the infiltration of M2c-like TAMs was negatively correlated with the expression of *S100A6*, which might be related to the decrease in cell number due to *S100A6*-induced apoptosis (Figure 5B). Patients with lower infiltration of M2c-like TAMs had longer survival times (Figure 5C).

To further investigate the potential relationship between immunosuppressive macrophages and immunotherapy response, we assessed the likelihood of each patient in the TCGA-CRC data to benefit from immune checkpoint blockade (ICB) treatment using EaSIeR. A patient with a higher relative score was more likely to respond to immunotherapy. Due to the great heterogeneity of immunotherapy response in pMMR patients, on the basis of the response scores using the K-means algorithm, they were categorized into three groups: pMMR high, pMMR median, and pMMR low (Figure S4B). dMMR patients were found to have higher response scores, indicating that they were more likely to benefit from immunotherapy (Figure 5D). There were statistically significant differences in response scores across the three groups of pMMR patients (Figure 5D). The infiltration rate of M2c-like TAMs was significantly negatively correlated with immunotherapy response in all four groups of CRC patients, suggesting that patients with lower infiltration rates of M2c-like TAMs might respond to immunotherapy (Figure 5E). We concluded that M2c-like TAMs are a subpopulation of macrophages with highly immunosuppressive profiles and highly expressed immune checkpoint ligands, and a lower infiltration rate of M2c-like TAMs is critical for patients to benefit from immunotherapy.

## 3. Discussion

To elucidate the mechanisms of tumor development, a sufficient understanding of macrophages in the TME of CRC patients with different MMR statuses is essential. In this study, we integrated the scRNA-seq data from dMMR patients and pMMR patients to identify a total of 10 populations of macrophages with distinct gene expression and functional characteristics. Of these, five distinct TAM populations played a critical role in tumor promotion and immunomodulation. The remaining TAMs, with the exception of C1QC+ TAMs, exhibited characteristics associated with tumor development. These findings also confirmed that infiltrating TAMs in tumor tissue tend to promote tumor development [23].

The infiltration of macrophages is an essential feature of the TME and is influenced by the patient’s MMR status [2]. Our results indicated differences in the abundance of some clusters of macrophages between dMMR and pMMR patients. More infiltration of immunoreactive macrophages was demonstrated in dMMR, whereas TAMs were more infiltrated in pMMR, which might explain the poorer clinical performance of pMMR patients [34]. Although TAMs were more enriched in pMMR, many of their pro-tumor functions were upregulated in dMMR, implying that the increased infiltration of TAMs had a greater impact on tumor development than the upregulation of pro-tumor function. Changes in the proportions of macrophages among different tumor grades have revealed their close relationship with tumor progression [35,36]. We speculated that increasing the infiltration of macrophages with tumor-promoting function and decreasing the infiltration of immunoreactive macrophages may drive cancer progression. In conclusion, future studies could focus more on the adverse effects of TAM infiltration on tumor development.

The “spectrum” model of macrophage polarization has received much recognition [37]. TAMs in tumors are commonly believed to polarize toward the M2 phenotype. This study identified the cell types involved in M2 polarization and constructed a well-defined M2 polarization trajectory. We found the M2 polarization of TAMs to be a tumor-promoting differentiation trajectory characterized by a steadily increasing M2 phenotype involving the upregulation of colony-stimulating factors, hypoxia, and angiogenesis factors and the downregulation of the immune function [38]. More infiltration of polarization terminal TAMs in advanced patients suggested that TAMs are likely to polarize toward the terminal along with tumor progression. M2a-like TAMs specific to pMMR revealed a different cell fate from their predecessor cell state M2c-like TAMs, suggesting a higher degree of M2 polarization of TAMs in pMMR, which may promote tumor progression and metastasis in such patients [39,40].

The increased apoptosis of M2c-like TAMs in dMMR was an essential reason for its failure to differentiate normally into M2a-like TAMs. This was confirmed by the upregulation of pro-apoptosis genes, pro-apoptosis pathways, and the regulatory activity of TFs in dMMR. Based on the differential expression of M2c-like TAMs in dMMR and pMMR, we finally identified seven hub genes. According to the difference in ranking, we found that *S100A6* was a marker of apoptosis in M2c-like TAMs, and its expression and that of *P53* were upregulated in dMMR. In addition, *P53* signaling pathway activity was upregulated in M2c like TAMs in dMMR. *S100A6* is an important calcium signal-regulating gene, and the calcium-binding protein it encodes is a member of the S100 family, which is associated with DNA damage and a variety of cancers [41]. *S100A6* has previously been shown to be a favorable prognostic marker for urothelial cancer. Previous research has demonstrated that when *S100A6* binds to RAGE, it causes apoptosis in epithelial cells by activating Jun N-terminal kinase and plays a crucial role in osteosarcoma metastasis [42,43,44]. *S100A6* can interact with *P53* in a calcium-dependent manner and upregulate its transcriptional activity, which may induce the high expression of *P53*-regulated downstream pro-apoptosis genes, such as *CASP3* and *CASP7* [33]. *P53* is a crucial tumor suppressor gene capable of inducing apoptosis [45]. *P53*-induced apoptosis in TAMs is an important mechanism to inhibit tumor growth. The mechanism of *S100A6* identified in these studies combined with the gene expression patterns in ours suggested that *S100A6* as a hub gene has the potential to trigger the apoptosis of M2c-like TAMs and, thus, inhibit their differentiation and reduce the degree of M2 polarization of TAMs in dMMR [33,45].

Exploring the impact of TAMs’ characteristics on the efficacy of immunotherapy in patients has become an essential focus of tumor immunology research [46]. TAMs can express a variety of immune checkpoint molecules that inhibit the immune response and promote tumor escape [4]. M2c-like TAMs in this study specifically expressed *PD1* and *CTLA4* ligands, and their highly immunosuppressive nature suggested that they might act as detrimental factors in anti-tumor immunity. We found a negative correlation between the level of M2c-like TAM infiltration and the predicted response of patients with different MMR statuses to immunotherapy, suggesting that immunotherapy may be ineffective in patients with a high level of infiltration of M2c-like TAMs. In addition, such patients had significantly lower survival rates. Moreover, the infiltration of M2c-like TAMs was likely to be reduced by the high expression of *S100A6*. These findings suggested that the infiltration of M2c-like TAMs has an important clinical value. In conclusion, *S100A6*-mediated apoptosis may reduce the infiltration of M2c-like TAMs, which will improve patient survival as well as immunotherapy efficacy.

However, this study has several limitations. While we identified a specific M2c-like TAM subtype, further validation is needed for the population of TAMs via flow cytometry. Further knockdown experiments could confirm the exact mechanism of how *S100A6* triggers apoptosis in M2c-like TAMs and affects their infiltration, which may provide more evidence. Whether M2c-like TAMs can be used as immunotherapeutic targets needs to be further investigated.

In short, we constructed a more comprehensive macrophage atlas and M2 polarization trajectory, elucidating the extensive heterogeneity and diversity of macrophages in the TME of dMMR and pMMR patients. The excessive apoptosis of the M2c-like TAM subpopulation in dMMR patients may reduce the level of infiltration and inhibit tumor development. The apoptosis-associated hub DEG S100A6 provides new clues to potential immunotherapeutic targets.

## 4. Materials and Methods

### 4.1. Data Processing

The single-cell RNA sequencing (scRNA-seq) data used in this paper were obtained from the GEO database, and the access number was GSE178341 [1]. We extracted the expression matrix of 18,976 tumor tissue macrophages from 28 pMMR and 34 dMMR patients for screening. Genes and cells that meet the following screening criteria were retained: (1) A gene was expressed in at least 10 cells, and a cell expressed at least 200 genes; (2) the sum of the counts per cell was less than 25,000, and the number of the expressed genes was less than 5000; and (3) the mitochondrial gene content was less than 15%, and the ribosomal gene content was less than 20%. Finally, a macrophage expression matrix containing 13,704 cells with 14,551 genes was obtained, and this expression matrix was used in the next analysis. In addition, the bulk RNA sequencing data from TCGA-COAD and TCGA-READ were downloaded from the UCSC Xena database (http://xena.ucsc.edu/ (accessed on 20 February 2022)) and integrated as TCGA-CRC. For subsequent analyses, the format Log2 (FPKM+1) was used.

### 4.2. Cell Clustering and Atlas Construction

To cluster all macrophages, the R package Seurat was used (version 4.0.5) [47]. In the initial clustering, we selected 2500 high variable genes for subsequent analysis, and the default parameters were used for the PCA. Since the data were obtained from multiple patient samples, Harmony (version 0.1.0) was used to integrate the data to remove batch effects [48]. Subsequently, the first 14 PCs were selected for dimensionality reduction, and the resolution parameter in FindClusters was set to 0.1. The macrophages were eventually classified into 7 subpopulations. Next, we extracted M2-like TAMs for the second clustering. In all, 2000 high variable genes were selected for subsequent analysis, and the default parameters were used for the PCA. The first 12 PCs were picked for dimensionality reduction, and the resolution parameter in FindClusters was set to 0.2. The visualization of the macrophage atlas was presented using UMAP.

### 4.3. Identification of Differentially Expressed Genes and Function Analysis

To identify differentially expressed genes for each subpopulation, we used the FindAllMarkers function in Seurat, and log FC > 0.25 and adj *p* < 0.05 as screening thresholds. To identify the differentially expressed genes of M2c-like TAMs in dMMR and pMMR, an analysis was performed using FindMarkers with the same thresholds as before. To characterize the functional differences among the subpopulations, macrophage-function-related gene sets were collected from previous studies for GSVA (version 1.42.0) [49]. Hallmark gene sets were downloaded from the MSigDB database, and the R package limma (version 3.50.1) was used to analyze the differences in pathway activity scores between dMMR and pMMR [50]. When assessing the immune infiltration scores, the kcdf parameter was set to “ssGSEA” for analysis. The GO pathway enrichment analysis of apoptosis-related genes was performed using the enrichGO function in the R package ClusterProfiler (version 4.2.2) with adj *p* < 0.05 [51].

### 4.4. Cell Abundance in Different Tissues

We counted a total of 10 clusters of macrophages in dMMR and pMMR, using chisq.test (R package stats, version 4.1.1) to determine whether there are differences in cell abundance in different tissues. The patients were then divided into different groups according to their tumor stage and node stage to observe the changes in cell abundance among different groups.

### 4.5. Inference of the Cell Differentiation Trajectory

To infer the differentiation polarization of M2-like TAMs, the “slingshot” method in the R package dyno (version 0.1.2) was used [52]. We used all the DEGs of the four subpopulations of M2-like TAMs as input for trajectory inference. After obtaining the cell differentiation trajectory, we finally determined the direction of the differentiation trajectory on the basis of the changes in the expression of some signature genes of macrophage differentiation.

### 4.6. Inference of Transcription Factors

The R package SCENIC (version 1.2.4) was used to identify candidate TFs from all the genes expressed by M2c-like TAMs and to analyze their transcriptional activities [53]. Subsequently, the top5 TFs with upregulated transcriptional activity in dMMR and pMMR were selected for visualization, respectively.

### 4.7. Analysis of Cell-to-Cell Communication

Cell-to-cell interactions were implemented by the R package CellChat (version 1.1.3) [54]. We imported all the human ligand–receptor interaction data in CellChatDB for analysis and then selected the LR pairs related to apoptosis or proliferation for further visualization.

### 4.8. Identification of Apoptosis-Related Hub Genes

We performed lasso regression to select the genes correlated with the apoptosis of M2c-like TAMs in dMMR. The apoptosis scores obtained from the apoptosis gene sets in hallmark were used as dependent variables, and all the DEGs were used as independent variables. The glmnet function in the R package glmnet was used for lasso regression, and the parameter lambda was selected by using 3-fold cross-validation. According to the above criteria, the data in dMMR and pMMR were regressed separately, and the intersection of the obtained genes was taken. The correlations between the intersecting genes and the apoptosis genes were calculated, and the genes that met the following criteria were removed: (1) positively correlated with more than half of the pro-apoptosis and anti-apoptosis genes at the same time, and (2) negatively correlated with more than half of the pro-apoptosis and anti-apoptosis genes at the same time. These genes were considered to have unclear apoptotic effects.

### 4.9. Immune Response Prediction

To predict the likelihood of patients responding to immune checkpoint inhibitors (ICI) treatment, the R package EaSIeR (version 1.0.0) was applied to calculate the immunotherapy response score for each patient [55]. This method integrated the quantitative descriptive information about the patient’s tumor mutation burden (TMB) and the immune microenvironment to predict the immune response. EaSIeR provided the likelihood of each patient benefitting from the ICI treatment, with a higher relative score indicating a higher likelihood of a patient to respond to immunotherapy.

### 4.10. Statistical Analysis

In this study, all the statistical analyses were implemented in R (version 4.1.1), and *p* < 0.05 was considered statistically significant. The detailed code is available from the GitHub link https://github.com/lhdLR66/CRC_Macro (accessed on 13 July 2022).

## Data Availability

Publicly available datasets were analyzed in this study. These data can be found here: GSE178341: https://www.ncbi.nlm.nih.gov/geo/query/acc.cgi?acc=GSE178341 (accessed on 13 July 2022); TCGA: https://portal.gdc.cancer.gov/repository (accessed on 13 July 2022).

## Supplementary Materials

The following supporting information can be downloaded at: https://www.mdpi.com/article/10.3390/ijms231911014/s1.
