# Supplementary material for: Infiltration of Apoptotic M2 Macrophage Subpopulation Is Negatively Correlated with the Immunotherapy Response in Colorectal Cancer
Source: Int J Mol Sci. 2022 Sep 20;23(19):11014. doi: 10.3390/ijms231911014 (PMC9569653; doi:10.3390/ijms231911014)
Supplement: Supplementary file 1 [file ijms-23-11014-s001.zip › Supplementary Table S1.pdf]

Table S1. Signature genes of 10 cluster of macrophages

cluster	gene	avg_log2FC	pct.1	pct.2	p_val	p_val_adj
SPP1+ TAM	CXCL5	2.709225	0.25	0.052	5.12E-249	7.46E-245
SPP1+ TAM	SPP1	2.112633	0.874	0.532	0	0
SPP1+ TAM	CCL2	1.87206	0.455	0.245	4.87E-148	7.08E-144
SPP1+ TAM	MARCO	1.77252	0.507	0.118	0	0
SPP1+ TAM	FN1	1.680068	0.285	0.087	2.10E-193	3.06E-189
SPP1+ TAM	S100A8	1.602719	0.748	0.352	0	0
SPP1+ TAM	CCL7	1.59955	0.267	0.048	3.65E-294	5.31E-290
SPP1+ TAM	FBP1	1.390352	0.657	0.337	0	0
SPP1+ TAM	VCAN	1.312989	0.398	0.109	0	0
SPP1+ TAM	SDC2	1.312898	0.628	0.228	0	0
SPP1+ TAM	CXCL8	1.268275	0.715	0.513	2.97E-129	4.32E-125
SPP1+ TAM	IL1RN	1.247884	0.379	0.184	1.71E-136	2.49E-132
SPP1+ TAM	SLC11A1	1.241196	0.8	0.378	0	0
SPP1+ TAM	INHBA	1.219283	0.278	0.064	1.65E-254	2.41E-250
SPP1+ TAM	RGCC	1.163989	0.585	0.243	0	0
SPP1+ TAM	CSTB	1.113414	0.987	0.912	0	0
SPP1+ TAM	LPL	1.099019	0.265	0.059	6.58E-251	9.57E-247
SPP1+ TAM	G0S2	1.085525	0.358	0.179	4.53E-112	6.59E-108
SPP1+ TAM	CHI3L1	1.073942	0.268	0.076	1.35E-193	1.96E-189
SPP1+ TAM	C15orf48	1.059949	0.923	0.611	0	0
SPP1+ TAM	AQP9	1.051157	0.559	0.134	0	0
SPP1+ TAM	PLAUR	1.029317	0.92	0.684	0	0
SPP1+ TAM	TIMP1	1.005549	0.861	0.678	4.48E-181	6.52E-177
SPP1+ TAM	RETN	0.983188	0.266	0.071	2.44E-209	3.55E-205
SPP1+ TAM	CLEC5A	0.968242	0.518	0.241	5.54E-241	8.06E-237
SPP1+ TAM	MT1H	0.965268	0.136	0.097	1.37E-11	2.00E-07

SPP1+ TAM	S100A6	0.951896	0.994	0.909	0	0
SPP1+ TAM	CCL20	0.936667	0.221	0.099	1.63E-81	2.38E-77
SPP1+ TAM	FABP5	0.912485	0.867	0.718	5.99E-163	8.72E-159
SPP1+ TAM	OLR1	0.910078	0.599	0.282	1.60E-274	2.33E-270
SPP1+ TAM	CHIT1	0.907272	0.156	0.048	4.42E-95	6.44E-91
SPP1+ TAM	BCL2A1	0.896854	0.746	0.459	4.80E-279	6.99E-275
SPP1+ TAM	ADM	0.894273	0.391	0.178	4.35E-158	6.33E-154
SPP1+ TAM	CTSL	0.889946	0.954	0.824	9.94E-216	1.45E-211
SPP1+ TAM	ANPEP	0.877486	0.485	0.158	0	0
SPP1+ TAM	S100A9	0.876528	0.943	0.731	9.75E-263	1.42E-258
SPP1+ TAM	VIM	0.874455	0.997	0.926	0	0
SPP1+ TAM	FTH1	0.866493	1	0.998	0	0
SPP1+ TAM	MMP9	0.8597	0.592	0.446	8.05E-68	1.17E-63
SPP1+ TAM	SOD2	0.849284	0.894	0.728	6.55E-157	9.53E-153
SPP1+ TAM	CCL18	0.831358	0.499	0.351	1.16E-58	1.69E-54
SPP1+ TAM	CD44	0.830392	0.931	0.715	0	0
SPP1+ TAM	FLNA	0.803175	0.726	0.418	2.15E-287	3.12E-283
SPP1+ TAM	GCHFR	0.80294	0.567	0.344	4.41E-150	6.42E-146
SPP1+ TAM	S100A10	0.774697	0.988	0.895	0	0
SPP1+ TAM	SLAMF9	0.77011	0.276	0.044	0	0
SPP1+ TAM	CYP27A1	0.769551	0.412	0.172	5.35E-199	7.79E-195
SPP1+ TAM	TREM1	0.763532	0.549	0.242	2.94E-271	4.28E-267
SPP1+ TAM	HK2	0.760463	0.392	0.15	6.91E-218	1.00E-213
SPP1+ TAM	CD300E	0.74056	0.292	0.077	4.39E-234	6.39E-230
SPP1+ TAM	SLC2A3	0.738193	0.644	0.443	8.74E-122	1.27E-117
SPP1+ TAM	MT1G	0.730753	0.272	0.212	8.57E-14	1.25E-09
SPP1+ TAM	EMP1	0.722784	0.447	0.139	0	0

SPP1+ TAM	EMP3	0.680332	0.924	0.751	3.24E-185	4.71E-181
SPP1+ TAM	PLIN2	0.678827	0.75	0.579	5.13E-115	7.47E-111
SPP1+ TAM	CXCL1	0.673984	0.31	0.212	3.49E-34	5.08E-30
SPP1+ TAM	TGM2	0.666265	0.468	0.231	2.47E-168	3.60E-164
SPP1+ TAM	MGST1	0.641525	0.334	0.095	7.62E-254	1.11E-249
SPP1+ TAM	PHLDA1	0.637939	0.463	0.248	6.03E-138	8.78E-134
SPP1+ TAM	LTA4H	0.637428	0.57	0.345	7.57E-159	1.10E-154
SPP1+ TAM	UPP1	0.630655	0.656	0.408	1.11E-192	1.62E-188
SPP1+ TAM	CD9	0.622535	0.878	0.674	2.15E-156	3.13E-152
SPP1+ TAM	SLC7A11	0.62243	0.194	0.044	2.60E-170	3.79E-166
SPP1+ TAM	ATP13A3	0.621819	0.586	0.394	1.05E-123	1.52E-119
SPP1+ TAM	NCF2	0.615731	0.75	0.524	2.19E-200	3.18E-196
SPP1+ TAM	GPNMB	0.614423	0.952	0.745	1.92E-185	2.79E-181
SPP1+ TAM	SH3BGRL3	0.590053	0.984	0.923	1.21E-225	1.76E-221
SPP1+ TAM	GLIPR2	0.57952	0.64	0.354	9.45E-231	1.38E-226
SPP1+ TAM	BCAT1	0.561055	0.637	0.396	5.68E-174	8.27E-170
SPP1+ TAM	MMP19	0.552315	0.466	0.228	1.40E-155	2.04E-151
SPP1+ TAM	ITGAX	0.546314	0.61	0.387	1.43E-148	2.08E-144
SPP1+ TAM	HAMP	0.541056	0.173	0.092	6.29E-39	9.16E-35
SPP1+ TAM	APOC1	0.540406	0.861	0.755	1.07E-40	1.55E-36
SPP1+ TAM	NDRG1	0.534561	0.414	0.23	1.07E-111	1.56E-107
SPP1+ TAM	SLC39A8	0.532185	0.442	0.293	4.45E-76	6.47E-72
SPP1+ TAM	MATK	0.529874	0.293	0.085	2.57E-211	3.73E-207

SPP1+ TAM	BNIP3	0.5245	0.399	0.218	2.91E-111	4.24E-107
SPP1+ TAM	CSF1	0.50602	0.171	0.051	3.31E-111	4.82E-107
SPP1+ TAM	GK	0.50401	0.566	0.323	8.97E-155	1.31E-150
SPP1+ TAM	SPARC	0.503855	0.26	0.111	2.81E-103	4.09E-99
SPP1+ TAM	FNDC3B	0.49588	0.646	0.431	1.75E-126	2.55E-122
SPP1+ TAM	RALA	0.47745	0.656	0.446	3.82E-132	5.55E-128
SPP1+ TAM	ACTN1	0.475578	0.662	0.466	2.37E-127	3.44E-123
SPP1+ TAM	ADAM10	0.471271	0.613	0.405	6.84E-133	9.96E-129
SPP1+ TAM	CYP1B1	0.470377	0.183	0.032	1.38E-194	2.01E-190
SPP1+ TAM	IL3RA	0.457092	0.255	0.108	1.95E-103	2.83E-99
SPP1+ TAM	CTSK	0.45047	0.158	0.09	2.12E-28	3.08E-24
SPP1+ TAM	FGR	0.449722	0.485	0.262	8.01E-146	1.16E-141
SPP1+ TAM	RAB13	0.437833	0.649	0.411	4.72E-160	6.87E-156
SPP1+ TAM	TNFAIP6	0.432127	0.143	0.037	1.42E-108	2.07E-104
SPP1+ TAM	SEMA3C	0.430487	0.264	0.061	9.99E-238	1.45E-233
SPP1+ TAM	LSP1	0.42381	0.782	0.581	9.05E-107	1.32E-102
SPP1+ TAM	YWHAZ	0.419845	0.874	0.716	3.32E-124	4.83E-120
SPP1+ TAM	ITGA5	0.412515	0.338	0.151	3.07E-132	4.47E-128
SPP1+ TAM	MT1M	0.409808	0.105	0.054	1.17E-25	1.70E-21
SPP1+ TAM	FLT1	0.407065	0.178	0.025	1.56E-223	2.27E-219
SPP1+ TAM	CD109	0.406519	0.311	0.142	9.16E-115	1.33E-110
SPP1+ TAM	PGK1	0.404839	0.919	0.801	1.81E-94	2.63E-90
SPP1+ TAM	FCN1	0.403354	0.157	0.058	1.15E-72	1.67E-68

SPP1+ TAM	PDE4DIP	0.39909	0.5	0.309	5.09E-101	7.40E-97
SPP1+ TAM	HSD3B7	0.396776	0.385	0.186	3.65E-135	5.32E-131
SPP1+ TAM	PDLIM7	0.39529	0.401	0.203	4.94E-126	7.19E-122
SPP1+ TAM	TM4SF19	0.394642	0.178	0.027	1.17E-213	1.71E-209
SPP1+ TAM	ADAM8	0.390766	0.464	0.301	2.54E-70	3.70E-66
SPP1+ TAM	FAM195A	0.385926	0.421	0.215	4.45E-133	6.48E-129
SPP1+ TAM	SLC2A1	0.38566	0.155	0.046	1.10E-101	1.60E-97
SPP1+ TAM	MT1F	0.382665	0.348	0.249	6.08E-33	8.84E-29
SPP1+ TAM	ARID5B	0.382177	0.404	0.242	2.02E-82	2.94E-78
SPP1+ TAM	P4HA1	0.381392	0.492	0.327	2.45E-80	3.57E-76
SPP1+ TAM	CORO1C	0.379791	0.728	0.528	7.98E-120	1.16E-115
SPP1+ TAM	SDC4	0.377268	0.335	0.157	1.31E-116	1.91E-112
SPP1+ TAM	ANXA2	0.374908	0.966	0.869	2.22E-121	3.23E-117
SPP1+ TAM	CTSD	0.368945	0.998	0.956	1.23E-72	1.79E-68
SPP1+ TAM	CD82	0.35991	0.411	0.214	2.56E-120	3.73E-116
SPP1+ TAM	ANXA1	0.357749	0.873	0.755	1.26E-50	1.83E-46
SPP1+ TAM	TSPO	0.355473	0.899	0.762	3.22E-96	4.69E-92
SPP1+ TAM	BASP1	0.348266	0.587	0.441	3.37E-59	4.91E-55
SPP1+ TAM	SLC16A3	0.346183	0.84	0.672	5.93E-86	8.64E-82
SPP1+ TAM	PTPN12	0.343638	0.556	0.392	4.73E-76	6.88E-72
SPP1+ TAM	OSCAR	0.342827	0.414	0.233	2.44E-101	3.56E-97
SPP1+ TAM	KCNN4	0.342691	0.257	0.086	3.51E-148	5.11E-144
SPP1+ TAM	VAT1	0.338523	0.528	0.326	4.94E-105	7.18E-101
SPP1+ TAM	TMEM38B	0.337702	0.322	0.143	2.81E-128	4.09E-124
SPP1+ TAM	FAM129B	0.335798	0.332	0.171	3.82E-94	5.56E-90
SPP1+ TAM	PDXK	0.334832	0.75	0.586	4.12E-96	6.00E-92
SPP1+ TAM	TPM4	0.331634	0.875	0.745	7.91E-74	1.15E-69
SPP1+ TAM	ASAP1	0.330902	0.436	0.279	4.35E-76	6.34E-72

SPP1+ TAM	HM13	0.328765	0.651	0.517	2.01E-65	2.93E-61
SPP1+ TAM	CXXC5	0.326127	0.282	0.112	3.19E-128	4.64E-124
SPP1+ TAM	CALM1	0.325252	0.948	0.845	2.13E-82	3.10E-78
SPP1+ TAM	CLIP4	0.324902	0.28	0.12	2.79E-111	4.06E-107
SPP1+ TAM	TNNT1	0.320605	0.145	0.035	2.17E-116	3.16E-112
SPP1+ TAM	SLC6A6	0.317474	0.451	0.298	1.30E-69	1.89E-65
SPP1+ TAM	HPCAL1	0.316112	0.452	0.272	2.32E-95	3.38E-91
SPP1+ TAM	RAI14	0.315909	0.148	0.023	1.33E-172	1.94E-168
SPP1+ TAM	TCIRG1	0.312752	0.632	0.473	1.28E-76	1.86E-72
SPP1+ TAM	PPARG	0.312433	0.183	0.037	5.74E-179	8.36E-175
SPP1+ TAM	TDP2	0.307271	0.483	0.298	2.09E-93	3.05E-89
SPP1+ TAM	BCL6	0.303191	0.211	0.084	7.82E-92	1.14E-87
SPP1+ TAM	CD55	0.302116	0.431	0.257	9.61E-89	1.40E-84
SPP1+ TAM	MFSD12	0.299239	0.535	0.399	6.48E-54	9.44E-50
SPP1+ TAM	LRP12	0.294932	0.254	0.123	1.17E-78	1.70E-74
SPP1+ TAM	PDPN	0.291102	0.289	0.161	2.55E-60	3.71E-56
SPP1+ TAM	SNTB1	0.290734	0.254	0.094	8.33E-126	1.21E-121
SPP1+ TAM	TNS1	0.288056	0.234	0.083	1.66E-120	2.41E-116
SPP1+ TAM	PLEC	0.2867	0.442	0.308	1.14E-53	1.66E-49
SPP1+ TAM	UBE2J1	0.28654	0.536	0.368	3.01E-74	4.38E-70
SPP1+ TAM	MT1E	0.283392	0.292	0.214	6.19E-21	9.01E-17
SPP1+ TAM	NRIP3	0.283199	0.196	0.056	2.26E-134	3.28E-130
SPP1+ TAM	ENO2	0.280426	0.181	0.054	1.53E-118	2.23E-114
SPP1+ TAM	CKAP4	0.278079	0.213	0.074	3.08E-115	4.48E-111
SPP1+ TAM	LAMP1	0.277887	0.905	0.777	1.53E-74	2.23E-70
SPP1+ TAM	ZNF281	0.276523	0.302	0.158	5.50E-79	8.00E-75
SPP1+ TAM	H2AFY	0.275873	0.88	0.754	5.84E-68	8.50E-64
SPP1+ TAM	BCAP31	0.27528	0.85	0.726	4.65E-70	6.77E-66
SPP1+ TAM	PNP	0.273841	0.317	0.171	2.68E-77	3.90E-73
SPP1+ TAM	MSANTD3	0.273184	0.204	0.069	2.47E-114	3.60E-110
SPP1+ TAM	YWHAG	0.272609	0.616	0.472	1.50E-58	2.19E-54

SPP1+ TAM	PTRF	0.271452	0.169	0.062	8.75E-83	1.27E-78
SPP1+ TAM	NUS1	0.270418	0.392	0.246	4.61E-67	6.70E-63
SPP1+ TAM	DFNA5	0.270048	0.252	0.112	1.48E-90	2.16E-86
SPP1+ TAM	STXBP2	0.267934	0.589	0.463	1.41E-45	2.05E-41
SPP1+ TAM	PGD	0.265619	0.635	0.492	2.33E-58	3.39E-54
SPP1+ TAM	QPCT	0.264449	0.201	0.077	1.86E-91	2.71E-87
SPP1+ TAM	IL6	0.260946	0.137	0.092	2.12E-14	3.08E-10
SPP1+ TAM	RMDN3	0.257986	0.35	0.229	6.21E-50	9.03E-46
SPP1+ TAM	MMP14	0.257505	0.677	0.581	1.21E-29	1.76E-25
SPP1+ TAM	CLIC4	0.256222	0.437	0.294	2.01E-58	2.92E-54
SPP1+ TAM	CAPN2	0.252654	0.363	0.222	5.44E-62	7.91E-58
SPP1+ TAM	DENND5A	0.250098	0.295	0.153	1.53E-76	2.22E-72
C1QC+ TAM	C1QC	0.843537	0.989	0.855	0	0
C1QC+ TAM	CD74	0.799115	1	0.984	0	0
C1QC+ TAM	C1QB	0.778598	0.99	0.863	0	0
C1QC+ TAM	C1QA	0.728422	0.991	0.887	4.89E-297	7.11E-293
C1QC+ TAM	TMEM176B	0.702841	0.933	0.748	2.89E-249	4.20E-245
C1QC+ TAM	TMEM176A	0.644478	0.885	0.662	3.92E-221	5.70E-217
C1QC+ TAM	HLA-DMB	0.640107	0.946	0.759	3.99E-228	5.81E-224
C1QC+ TAM	FUCA1	0.627854	0.644	0.462	2.17E-96	3.15E-92
C1QC+ TAM	MGST2	0.614314	0.781	0.496	8.45E-250	1.23E-245
C1QC+ TAM	AMICA1	0.55051	0.411	0.24	2.63E-90	3.82E-86
C1QC+ TAM	IFI30	0.530534	0.672	0.456	4.15E-128	6.04E-124
C1QC+ TAM	IGSF6	0.51647	0.873	0.667	3.57E-158	5.19E-154
C1QC+ TAM	SLC25A6	0.514716	0.924	0.766	8.30E-183	1.21E-178
C1QC+ TAM	ADAMDEC1	0.501318	0.594	0.345	8.66E-139	1.26E-134
C1QC+ TAM	HSPB1	0.483401	0.844	0.696	5.30E-84	7.71E-80
C1QC+ TAM	ACP5	0.476041	0.887	0.761	1.45E-85	2.10E-81
C1QC+ TAM	C1orf54	0.452542	0.764	0.526	1.80E-156	2.62E-152
C1QC+ TAM	LST1	0.445271	0.956	0.808	2.61E-178	3.80E-174

C1QC+ TAM	ABI3	0.441441	0.668	0.413	2.50E-167	3.63E-163
C1QC+ TAM	LY86	0.422311	0.715	0.505	5.54E-130	8.06E-126
C1QC+ TAM	CD300A	0.415225	0.646	0.455	2.28E-105	3.32E-101
C1QC+ TAM	PRMT9	0.408597	0.374	0.226	2.10E-70	3.06E-66
C1QC+ TAM	SPINT2	0.388951	0.766	0.567	2.08E-130	3.02E-126
C1QC+ TAM	NPC2	0.377247	0.999	0.961	1.32E-193	1.92E-189
C1QC+ TAM	PPIB	0.370437	0.897	0.742	2.74E-107	3.99E-103
C1QC+ TAM	SYNGR2	0.365995	0.821	0.644	1.77E-107	2.58E-103
C1QC+ TAM	CYBA	0.34898	0.997	0.954	3.92E-162	5.71E-158
C1QC+ TAM	DNASE2	0.34274	0.617	0.45	1.66E-77	2.41E-73
C1QC+ TAM	RENBP	0.338656	0.615	0.408	9.24E-120	1.35E-115
C1QC+ TAM	PLD3	0.338473	0.903	0.749	8.66E-81	1.26E-76
C1QC+ TAM	TSPAN4	0.323943	0.611	0.449	5.22E-77	7.60E-73
C1QC+ TAM	FKBP2	0.309413	0.782	0.603	1.84E-101	2.67E-97
C1QC+ TAM	HSPA6	0.291083	0.248	0.192	1.76E-14	2.56E-10
C1QC+ TAM	HIGD2A	0.286965	0.806	0.638	2.05E-102	2.99E-98
C1QC+ TAM	ID3	0.266794	0.308	0.244	9.11E-16	1.33E-11
C1QC+ TAM	ABHD12	0.258804	0.598	0.448	3.05E-67	4.44E-63
M2a like TAM	FCGBP	2.960541	0.745	0.093	6.66E-290	9.68E-286
M2a like TAM	C3	2.94352	0.828	0.097	0	0
M2a like TAM	A2M	2.43899	1	0.562	1.96E-165	2.85E-161
M2a like TAM	HERPUD1	2.313171	0.996	0.689	1.34E-157	1.95E-153
M2a like TAM	CX3CR1	2.213108	0.719	0.031	0	0
M2a like TAM	PDK4	2.13155	0.872	0.26	1.28E-145	1.87E-141
M2a like TAM	PLD4	1.989522	0.843	0.081	0	0
M2a like TAM	OLFML3	1.984142	0.876	0.159	1.72E-246	2.50E-242

M2a like TAM	MAF	1.783158	0.978	0.545	2.73E-121	3.98E-117
M2a like TAM	STAB1	1.776856	0.974	0.567	1.47E-106	2.14E-102
M2a like TAM	USP53	1.767723	0.807	0.136	1.50E-242	2.19E-238
M2a like TAM	FOLR2	1.747475	0.872	0.322	2.46E-115	3.59E-111
M2a like TAM	SLCO2B1	1.664223	0.989	0.658	2.61E-118	3.80E-114
M2a like TAM	SLC40A1	1.63274	0.978	0.499	4.74E-94	6.89E-90
M2a like TAM	PABPC4	1.530615	0.989	0.552	5.23E-120	7.61E-116
M2a like TAM	AXL	1.522079	0.96	0.381	1.36E-136	1.98E-132
M2a like TAM	HTRA1	1.521455	0.631	0.172	5.18E-98	7.54E-94
M2a like TAM	CADM1	1.498987	0.799	0.175	7.25E-183	1.05E-178
M2a like TAM	MS4A7	1.492477	1	0.831	4.54E-110	6.61E-106
M2a like TAM	SEPP1	1.484321	0.894	0.396	3.35E-79	4.88E-75
M2a like TAM	KLF2	1.482478	0.818	0.419	2.86E-62	4.16E-58
M2a like TAM	IRS2	1.455004	0.901	0.302	7.78E-140	1.13E-135
M2a like TAM	CXCR4	1.443785	0.967	0.559	3.47E-86	5.05E-82
M2a like TAM	C5AR1	1.425133	0.953	0.603	1.91E-85	2.78E-81
M2a like TAM	ZFHX3	1.407122	0.865	0.305	3.17E-125	4.62E-121
M2a like TAM	FGL2	1.399721	0.982	0.677	5.05E-91	7.35E-87
M2a like TAM	CEBPD	1.399093	1	0.696	3.38E-107	4.92E-103
M2a like TAM	DAB2	1.397088	0.993	0.729	3.95E-90	5.74E-86
M2a like TAM	REL	1.395232	0.949	0.468	7.49E-103	1.09E-98
M2a like TAM	AP1B1	1.390214	0.96	0.494	1.87E-110	2.72E-106
M2a like TAM	IGF1	1.390094	0.759	0.113	8.34E-228	1.21E-223
M2a like TAM	PLXDC2	1.377636	0.978	0.56	2.57E-101	3.73E-97
M2a like TAM	ZFP36L2	1.343837	0.996	0.664	1.77E-87	2.58E-83
M2a like TAM	OGFRL1	1.333307	0.964	0.483	3.23E-109	4.71E-105

M2a like TAM	GPR34	1.319216	0.971	0.5	7.13E-106	1.04E-101
M2a like TAM	GRASP	1.299099	0.755	0.17	2.44E-158	3.55E-154
M2a like TAM	CSF1R	1.296494	0.996	0.665	5.86E-102	8.53E-98
M2a like TAM	LTC4S	1.296261	0.73	0.13	4.54E-186	6.60E-182
M2a like TAM	MEF2C	1.287488	0.949	0.396	4.23E-112	6.15E-108
M2a like TAM	AKAP13	1.269223	0.967	0.48	1.66E-111	2.41E-107
M2a like TAM	SEC14L1	1.268618	0.938	0.413	1.96E-113	2.86E-109
M2a like TAM	ANKH	1.267995	0.821	0.339	1.51E-90	2.19E-86
M2a like TAM	RNASE1	1.264939	0.909	0.689	6.04E-46	8.79E-42
M2a like TAM	PELI1	1.260668	0.752	0.169	7.39E-159	1.08E-154
M2a like TAM	EPB41L2	1.256795	0.843	0.238	8.62E-147	1.25E-142
M2a like TAM	CD81	1.247938	1	0.78	1.50E-103	2.18E-99
M2a like TAM	MERTK	1.245345	0.92	0.401	3.07E-106	4.47E-102
M2a like TAM	GNAS	1.244851	0.996	0.721	2.02E-108	2.94E-104
M2a like TAM	MCL1	1.223216	1	0.799	2.95E-97	4.29E-93
M2a like TAM	GAL3ST4	1.212287	0.836	0.301	3.07E-104	4.46E-100
M2a like TAM	ADAM28	1.208342	0.85	0.271	8.32E-124	1.21E-119
M2a like TAM	SORL1	1.202314	0.755	0.205	9.03E-123	1.31E-118
M2a like TAM	PABPC1	1.201547	1	0.863	8.61E-108	1.25E-103
M2a like TAM	CD93	1.192813	0.887	0.398	1.69E-83	2.46E-79
M2a like TAM	SGK1	1.186081	0.993	0.702	1.59E-70	2.31E-66
M2a like TAM	CELF2	1.184572	0.967	0.524	8.17E-95	1.19E-90
M2a like TAM	MEF2A	1.169414	0.847	0.335	4.38E-99	6.37E-95
M2a like TAM	ITPR2	1.167126	0.912	0.406	3.27E-96	4.76E-92
M2a like TAM	TSC22D3	1.162222	1	0.696	4.26E-84	6.20E-80
M2a like TAM	PIK3R1	1.158497	0.858	0.292	5.83E-119	8.48E-115

M2a like TAM	HLA-DPA1	1.142354	0.989	0.959	1.89E-57	2.76E-53
M2a like TAM	FILIP1L	1.128479	0.661	0.164	2.35E-109	3.42E-105
M2a like TAM	HPGDS	1.12633	0.639	0.061	2.14E-293	3.11E-289
M2a like TAM	TCF4	1.126313	0.876	0.374	2.67E-87	3.89E-83
M2a like TAM	ST6GAL1	1.113491	0.777	0.187	1.40E-149	2.03E-145
M2a like TAM	TNFRSF1B	1.104753	0.942	0.495	3.71E-87	5.39E-83
M2a like TAM	CSF2RA	1.100788	0.887	0.377	1.40E-96	2.04E-92
M2a like TAM	ETS2	1.09234	0.949	0.519	1.34E-76	1.95E-72
M2a like TAM	SKIL	1.086077	0.872	0.365	2.76E-93	4.02E-89
M2a like TAM	SH2B3	1.083745	0.876	0.398	1.29E-90	1.87E-86
M2a like TAM	ARL4C	1.079011	0.96	0.579	9.02E-66	1.31E-61
M2a like TAM	KLF6	1.072712	0.971	0.779	3.17E-42	4.62E-38
M2a like TAM	RGS1	1.068873	1	0.818	1.47E-78	2.13E-74
M2a like TAM	ABCC5	1.065134	0.759	0.219	2.19E-116	3.19E-112
M2a like TAM	NR4A1	1.065024	0.788	0.315	8.59E-74	1.25E-69
M2a like TAM	NR4A3	1.060241	0.588	0.163	1.57E-82	2.28E-78
M2a like TAM	SLA	1.05993	0.953	0.541	4.04E-82	5.88E-78
M2a like TAM	CYFIP1	1.055932	0.927	0.548	3.36E-75	4.89E-71
M2a like TAM	PER1	1.049836	0.843	0.285	5.06E-113	7.36E-109
M2a like TAM	DDX3X	1.044539	0.96	0.581	1.73E-74	2.52E-70
M2a like TAM	HLA-DQA2	1.041895	0.832	0.245	6.00E-104	8.72E-100
M2a like TAM	SLC4A7	1.038872	0.832	0.343	3.29E-85	4.79E-81
M2a like TAM	BMP2K	1.028616	0.92	0.493	7.23E-73	1.05E-68
M2a like TAM	DST	1.024902	0.755	0.176	2.92E-143	4.25E-139
M2a like TAM	GNB4	1.024374	0.927	0.55	1.89E-69	2.75E-65
M2a like TAM	KCTD12	1.023772	0.978	0.728	4.30E-74	6.26E-70
M2a like TAM	DOCK4	1.023225	0.821	0.324	1.02E-86	1.48E-82
M2a like TAM	FKBP5	1.022941	0.92	0.425	1.37E-86	2.00E-82
M2a like TAM	TGFBR1	1.022774	0.836	0.348	1.83E-86	2.67E-82
M2a like TAM	ATRX	1.018949	0.872	0.389	6.64E-85	9.67E-81
M2a like TAM	SRGAP1	1.015225	0.62	0.097	2.52E-177	3.66E-173
M2a like TAM	MAT2A	1.014157	0.891	0.491	2.57E-64	3.74E-60
M2a like TAM	WLS	1.00617	0.704	0.116	5.37E-191	7.82E-187

M2a like TAM	TMCC3	1.002768	0.748	0.189	7.91E-129	1.15E-124
M2a like TAM	FCGRT	1.000322	1	0.85	8.35E-66	1.21E-61
M2a like TAM	ITGAV	0.998979	0.843	0.364	1.51E-80	2.20E-76
M2a like TAM	RHOB	0.990618	0.894	0.634	3.22E-31	4.68E-27
M2a like TAM	ADORA3	0.987706	0.785	0.219	1.04E-116	1.51E-112
M2a like TAM	DDX5	0.986014	1	0.83	1.82E-89	2.65E-85
M2a like TAM	LPAR6	0.981702	0.916	0.43	7.50E-80	1.09E-75
M2a like TAM	DDX17	0.979189	0.974	0.574	4.76E-77	6.93E-73
M2a like TAM	HLA-DOA	0.970366	0.785	0.385	4.66E-62	6.78E-58
M2a like TAM	GATM	0.96738	0.799	0.28	2.94E-91	4.28E-87
M2a like TAM	ADAP2	0.964105	0.953	0.558	2.54E-74	3.70E-70
M2a like TAM	QKI	0.962759	0.978	0.624	2.61E-73	3.79E-69
M2a like TAM	NCOA4	0.955	0.964	0.586	8.46E-71	1.23E-66
M2a like TAM	ZFAND5	0.946549	0.942	0.683	2.20E-41	3.20E-37
M2a like TAM	RNF144B	0.945349	0.814	0.32	3.24E-80	4.72E-76
M2a like TAM	AAK1	0.943721	0.847	0.369	1.68E-82	2.44E-78
M2a like TAM	PHACTR1	0.940546	0.777	0.34	2.20E-60	3.20E-56
M2a like TAM	KLF7	0.936362	0.715	0.182	1.28E-119	1.86E-115
M2a like TAM	SLC1A3	0.932108	0.818	0.341	6.90E-74	1.00E-69
M2a like TAM	CPM	0.932038	0.814	0.448	2.55E-51	3.71E-47
M2a like TAM	EIF4B	0.929952	0.898	0.484	4.03E-72	5.86E-68
M2a like TAM	SMAP2	0.924684	0.901	0.455	6.56E-68	9.54E-64
M2a like TAM	FCHO2	0.924641	0.81	0.316	8.66E-84	1.26E-79
M2a like TAM	SESN1	0.924414	0.62	0.131	1.03E-127	1.50E-123
M2a like TAM	TLR7	0.922481	0.675	0.132	1.44E-149	2.10E-145
M2a like TAM	KDM6B	0.922312	0.719	0.277	8.57E-69	1.25E-64
M2a like TAM	TBXAS1	0.920267	0.956	0.494	1.28E-80	1.86E-76
M2a like TAM	VSIG4	0.919078	0.894	0.532	2.60E-42	3.79E-38
M2a like TAM	P2RY13	0.91787	0.701	0.198	4.96E-97	7.21E-93
M2a like TAM	HLA-DRB5	0.91549	0.927	0.524	1.73E-49	2.52E-45
M2a like TAM	ZFP36	0.915414	0.993	0.747	9.53E-54	1.39E-49
M2a like TAM	MARCKS	0.907495	1	0.762	8.75E-65	1.27E-60
M2a like TAM	SIGLEC10	0.90655	0.803	0.338	8.25E-71	1.20E-66
M2a like TAM	CREBRF	0.901523	0.755	0.278	2.28E-77	3.32E-73
M2a like TAM	TCHH	0.900088	0.35	0.049	7.37E-107	1.07E-102
M2a like TAM	ENTPD1	0.899735	0.85	0.414	2.50E-67	3.63E-63

M2a like TAM	CTNND1	0.895386	0.861	0.388	2.16E-78	3.14E-74
M2a like TAM	AP2A2	0.894482	0.872	0.38	1.30E-78	1.89E-74
M2a like TAM	TTC3	0.892703	0.883	0.369	9.31E-83	1.35E-78
M2a like TAM	PAG1	0.889432	0.799	0.358	9.64E-65	1.40E-60
M2a like TAM	ITM2B	0.884352	1	0.947	9.00E-83	1.31E-78
M2a like TAM	FRMD4A	0.881328	0.763	0.302	1.25E-73	1.81E-69
M2a like TAM	BIN1	0.880558	0.533	0.145	3.35E-78	4.87E-74
M2a like TAM	PIK3IP1	0.879188	0.679	0.203	6.63E-91	9.65E-87
M2a like TAM	GAS6	0.875779	0.701	0.241	8.88E-76	1.29E-71
M2a like TAM	SH3BP5	0.867019	0.781	0.305	6.45E-73	9.39E-69
M2a like TAM	FOXN3	0.86694	0.832	0.402	1.43E-67	2.08E-63
M2a like TAM	NFATC2	0.864576	0.672	0.141	2.76E-138	4.02E-134
M2a like TAM	GPRIN3	0.863594	0.792	0.357	1.08E-61	1.57E-57
M2a like TAM	NUDT3	0.861074	0.792	0.256	1.64E-99	2.39E-95
M2a like TAM	THEMIS2	0.859913	0.909	0.469	1.75E-69	2.55E-65
M2a like TAM	APBB1IP	0.848571	0.781	0.31	5.25E-77	7.64E-73
M2a like TAM	KCNQ3	0.846044	0.445	0.005	0	0
M2a like TAM	UNC5B	0.84563	0.584	0.09	4.78E-171	6.95E-167
M2a like TAM	SH3TC1	0.84545	0.675	0.155	1.77E-128	2.57E-124
M2a like TAM	MTUS1	0.843695	0.504	0.041	1.39E-269	2.02E-265
M2a like TAM	DPYSL2	0.841928	0.916	0.504	1.18E-63	1.72E-59
M2a like TAM	MAP3K8	0.840355	0.763	0.329	1.92E-63	2.79E-59
M2a like TAM	PRDM1	0.84026	0.763	0.32	3.51E-58	5.11E-54
M2a like TAM	IL1RAP	0.838802	0.613	0.178	2.21E-83	3.21E-79
M2a like TAM	SESN3	0.831982	0.558	0.087	9.74E-157	1.42E-152
M2a like TAM	LCP2	0.828309	0.905	0.531	1.13E-47	1.64E-43
M2a like TAM	CD4	0.827763	0.971	0.657	3.83E-56	5.58E-52
M2a like TAM	KLF9	0.824117	0.69	0.226	5.12E-80	7.46E-76
M2a like TAM	ZG16	0.819778	0.566	0.038	0	0
M2a like TAM	AKR1B1	0.808573	0.96	0.616	1.29E-58	1.88E-54
M2a like TAM	GYPC	0.807486	0.704	0.338	2.76E-46	4.02E-42
M2a like TAM	MYCBP2	0.80644	0.796	0.345	7.23E-67	1.05E-62
M2a like TAM	C3AR1	0.805995	0.978	0.677	1.18E-52	1.72E-48
M2a like TAM	SUSD6	0.804819	0.836	0.386	7.31E-64	1.06E-59
M2a like TAM	CPEB4	0.804501	0.737	0.322	1.69E-54	2.46E-50
M2a like TAM	TPT1	0.800325	1	0.988	3.42E-76	4.98E-72
M2a like TAM	TRA2B	0.799483	0.88	0.537	1.12E-41	1.63E-37

M2a like TAM	FPR1	0.798788	0.818	0.378	7.34E-55	1.07E-50
M2a like TAM	ARHGAP21	0.796635	0.635	0.176	9.58E-93	1.39E-88
M2a like TAM	PFKFB3	0.791831	0.737	0.321	1.22E-54	1.77E-50
M2a like TAM	NFKBID	0.790446	0.635	0.213	2.63E-68	3.83E-64
M2a like TAM	AKAP9	0.78852	0.839	0.369	2.79E-66	4.06E-62
M2a like TAM	SLC15A3	0.786974	0.872	0.511	2.46E-44	3.58E-40
M2a like TAM	PREX1	0.78614	0.781	0.348	3.01E-63	4.38E-59
M2a like TAM	ANKRD11	0.782158	0.763	0.314	1.41E-65	2.05E-61
M2a like TAM	CCNL1	0.782056	0.912	0.512	8.58E-53	1.25E-48
M2a like TAM	CEP350	0.781678	0.734	0.289	8.45E-67	1.23E-62
M2a like TAM	ZC3HAV1	0.779478	0.642	0.265	3.45E-52	5.02E-48
M2a like TAM	MAN2A1	0.779013	0.726	0.256	6.75E-79	9.82E-75
M2a like TAM	STK17B	0.777488	0.832	0.36	5.88E-67	8.55E-63
M2a like TAM	USP9X	0.776528	0.821	0.356	8.74E-69	1.27E-64
M2a like TAM	KLHL24	0.774478	0.653	0.227	2.34E-71	3.40E-67
M2a like TAM	PALD1	0.774124	0.573	0.105	1.04E-134	1.52E-130
M2a like TAM	TACC1	0.770664	0.88	0.478	1.59E-57	2.31E-53
M2a like TAM	C6orf62	0.769032	0.949	0.623	1.35E-49	1.96E-45
M2a like TAM	UBL3	0.768481	0.766	0.318	1.14E-68	1.66E-64
M2a like TAM	MALT1	0.767548	0.588	0.175	1.97E-75	2.87E-71
M2a like TAM	GTF2I	0.766321	0.861	0.419	1.03E-61	1.49E-57
M2a like TAM	CEBPB	0.765631	0.989	0.793	5.69E-40	8.28E-36
M2a like TAM	PLXNC1	0.765257	0.792	0.45	2.46E-42	3.58E-38
M2a like TAM	ZNF331	0.764605	0.682	0.406	4.02E-24	5.86E-20
M2a like TAM	FUS	0.762186	0.934	0.587	5.67E-48	8.24E-44
M2a like TAM	SAP30	0.755925	0.642	0.256	5.77E-51	8.39E-47
M2a like TAM	NCKAP1L	0.754241	0.923	0.524	6.30E-57	9.17E-53
M2a like TAM	FRMD4B	0.754084	0.836	0.422	2.68E-55	3.90E-51
M2a like TAM	ITM2C	0.753917	0.642	0.156	8.17E-104	1.19E-99
M2a like TAM	GNAI2	0.753164	0.993	0.764	6.64E-59	9.66E-55
M2a like TAM	TREM2	0.752333	0.912	0.55	1.25E-40	1.82E-36
M2a like TAM	NAIP	0.751098	0.869	0.458	1.05E-54	1.53E-50
M2a like TAM	MGAT4A	0.745216	0.865	0.468	1.91E-52	2.78E-48
M2a like TAM	ARRDC3	0.745137	0.752	0.378	3.18E-39	4.62E-35
M2a like TAM	BOD1L1	0.743829	0.73	0.25	5.80E-79	8.43E-75
M2a like TAM	NIPBL	0.743482	0.745	0.303	2.20E-64	3.20E-60
M2a like TAM	VEGFA	0.742428	0.653	0.287	5.24E-43	7.62E-39
M2a like TAM	UVSSA	0.741696	0.573	0.109	1.06E-129	1.55E-125
M2a like TAM	GNA13	0.735289	0.894	0.458	1.40E-53	2.04E-49

M2a like TAM	PDGFB	0.734547	0.445	0.087	8.78E-95	1.28E-90
M2a like TAM	APPL2	0.733847	0.624	0.153	5.68E-106	8.26E-102
M2a like TAM	SWAP70	0.732612	0.777	0.382	4.26E-52	6.19E-48
M2a like TAM	RSRP1	0.73253	0.858	0.431	2.54E-47	3.69E-43
M2a like TAM	PLXDC1	0.731687	0.464	0.027	0	0
M2a like TAM	GOLGB1	0.730584	0.704	0.27	6.73E-64	9.80E-60
M2a like TAM	SFMBT2	0.729388	0.58	0.213	2.04E-55	2.97E-51
M2a like TAM	MDM2	0.728785	0.599	0.238	5.35E-49	7.79E-45
M2a like TAM	CREM	0.726749	0.909	0.556	4.96E-42	7.22E-38
M2a like TAM	NLRP3	0.726116	0.547	0.161	2.79E-67	4.06E-63
M2a like TAM	ARRDC2	0.725381	0.588	0.155	8.51E-87	1.24E-82
M2a like TAM	SIPA1	0.724794	0.737	0.286	1.23E-70	1.80E-66
M2a like TAM	TGFBR2	0.72472	0.788	0.363	1.97E-56	2.87E-52
M2a like TAM	PNISR	0.724011	0.894	0.455	6.75E-56	9.82E-52
M2a like TAM	PRPF8	0.72106	0.73	0.266	4.67E-77	6.79E-73
M2a like TAM	IGFBP4	0.720716	0.5	0.173	6.77E-45	9.84E-41
M2a like TAM	TNRC18	0.72029	0.602	0.158	9.56E-91	1.39E-86
M2a like TAM	INPP5D	0.720223	0.682	0.221	1.95E-79	2.84E-75
M2a like TAM	MXD4	0.718519	0.679	0.208	4.02E-87	5.85E-83
M2a like TAM	VPS13C	0.715838	0.81	0.36	8.47E-60	1.23E-55
M2a like TAM	PICALM	0.711454	0.898	0.505	1.52E-50	2.20E-46
M2a like TAM	RANBP2	0.71128	0.745	0.314	5.91E-56	8.60E-52
M2a like TAM	MIS18BP1	0.709171	0.588	0.223	2.45E-51	3.57E-47
M2a like TAM	TNRC6B	0.708617	0.712	0.291	9.13E-60	1.33E-55
M2a like TAM	HELZ	0.70551	0.734	0.292	8.51E-64	1.24E-59
M2a like TAM	TLR2	0.704918	0.796	0.416	1.18E-43	1.72E-39
M2a like TAM	HNRNPUL1	0.703413	0.788	0.362	5.04E-58	7.33E-54
M2a like TAM	EPS8	0.702456	0.562	0.105	5.19E-124	7.55E-120
M2a like TAM	SRGAP2	0.701202	0.675	0.244	1.39E-66	2.02E-62
M2a like TAM	PTEN	0.699334	0.85	0.407	4.17E-60	6.06E-56
M2a like TAM	PRPF38B	0.698484	0.774	0.429	2.19E-40	3.19E-36
M2a like TAM	PLVAP	0.697948	0.376	0.015	0	0
M2a like TAM	FGD4	0.695022	0.682	0.284	5.16E-53	7.51E-49
M2a like TAM	DAGLB	0.692193	0.569	0.132	2.43E-99	3.54E-95
M2a like TAM	ADRBK1	0.691006	0.752	0.32	1.37E-58	2.00E-54
M2a like TAM	NRIP1	0.690383	0.682	0.259	1.41E-60	2.05E-56
M2a like TAM	BAZ2B	0.690071	0.704	0.27	2.21E-64	3.21E-60
M2a like TAM	MYO9B	0.688102	0.821	0.429	1.41E-50	2.06E-46
M2a like TAM	RASSF2	0.687654	0.631	0.267	2.70E-47	3.94E-43
M2a like TAM	TRIB1	0.686969	0.573	0.223	2.10E-45	3.06E-41

M2a like TAM	PPP1R10	0.686519	0.69	0.269	8.48E-60	1.23E-55
M2a like TAM	IFNGR1	0.686176	0.985	0.672	1.95E-41	2.84E-37
M2a like TAM	PDCD4	0.684172	0.672	0.213	1.02E-76	1.48E-72
M2a like TAM	WBP1L	0.684039	0.639	0.189	7.24E-81	1.05E-76
M2a like TAM	ZNF217	0.683617	0.599	0.181	1.23E-73	1.79E-69
M2a like TAM	STAG2	0.682025	0.723	0.282	3.15E-64	4.59E-60
M2a like TAM	IRAK3	0.681261	0.719	0.297	2.44E-55	3.55E-51
M2a like TAM	FMNL3	0.679232	0.573	0.177	1.09E-69	1.58E-65
M2a like TAM	WSB1	0.676595	0.956	0.626	3.88E-43	5.65E-39
M2a like TAM	DAAM1	0.676561	0.5	0.135	2.13E-70	3.10E-66
M2a like TAM	JDP2	0.676366	0.642	0.214	6.89E-70	1.00E-65
M2a like TAM	GLIPR1	0.675461	0.956	0.617	3.14E-42	4.57E-38
M2a like TAM	FSCN1	0.675191	0.42	0.097	4.44E-70	6.46E-66
M2a like TAM	PDGFC	0.674759	0.562	0.145	5.33E-83	7.76E-79
M2a like TAM	PRRC2B	0.674433	0.595	0.17	2.84E-81	4.14E-77
M2a like TAM	FOXO3	0.673097	0.682	0.27	2.75E-54	4.00E-50
M2a like TAM	ZSWIM6	0.672952	0.661	0.249	9.86E-60	1.43E-55
M2a like TAM	CEP170	0.67145	0.774	0.364	2.38E-49	3.46E-45
M2a like TAM	NUFIP2	0.671442	0.777	0.34	1.62E-58	2.36E-54
M2a like TAM	PLXNB2	0.670399	0.701	0.289	2.01E-57	2.92E-53
M2a like TAM	CD163L1	0.670029	0.504	0.139	1.63E-64	2.37E-60
M2a like TAM	IL10RA	0.668946	0.858	0.419	2.42E-53	3.53E-49
M2a like TAM	VASH1	0.666952	0.653	0.244	8.45E-61	1.23E-56
M2a like TAM	C2	0.666857	0.752	0.441	2.70E-30	3.92E-26
M2a like TAM	TMEM173	0.666661	0.642	0.264	5.46E-50	7.94E-46
M2a like TAM	CPED1	0.66342	0.558	0.126	1.71E-101	2.48E-97
M2a like TAM	IL2RA	0.662946	0.529	0.214	2.34E-36	3.40E-32
M2a like TAM	GPR82	0.662675	0.485	0.151	1.77E-55	2.57E-51
M2a like TAM	CXCL16	0.661427	0.985	0.722	1.40E-38	2.03E-34
M2a like TAM	LPIN2	0.661221	0.606	0.223	1.79E-56	2.61E-52
M2a like TAM	ARID1B	0.661161	0.763	0.323	5.33E-59	7.75E-55
M2a like TAM	NFIC	0.661105	0.755	0.366	1.74E-47	2.53E-43
M2a like TAM	ITGA9	0.660741	0.544	0.15	4.29E-73	6.24E-69
M2a like TAM	UTRN	0.660296	0.73	0.302	7.11E-56	1.03E-51
M2a like TAM	MTR	0.657877	0.547	0.153	4.75E-75	6.92E-71
M2a like TAM	ATP8B4	0.656549	0.635	0.199	4.49E-74	6.54E-70
M2a like TAM	ZMYM2	0.654239	0.657	0.242	2.89E-60	4.20E-56
M2a like TAM	ATP2B1	0.652877	0.774	0.371	2.04E-47	2.97E-43
M2a like TAM	METTL7A	0.652012	0.631	0.266	1.51E-45	2.20E-41
M2a like TAM	SETX	0.651992	0.73	0.309	5.08E-56	7.39E-52
M2a like TAM	ITGB5	0.651485	0.701	0.266	1.22E-61	1.77E-57

M2a like TAM	TCF12	0.649071	0.606	0.206	3.98E-63	5.80E-59
M2a like TAM	KDM5A	0.648945	0.679	0.288	1.60E-53	2.33E-49
M2a like TAM	MRC2	0.647928	0.493	0.099	1.43E-101	2.08E-97
M2a like TAM	RBMX	0.647782	0.832	0.469	4.52E-39	6.58E-35
M2a like TAM	CCND1	0.647454	0.591	0.2	8.76E-57	1.28E-52
M2a like TAM	GATAD1	0.646464	0.675	0.24	4.81E-66	7.01E-62
M2a like TAM	PCDH12	0.645523	0.314	0.014	2.61E-267	3.80E-263
M2a like TAM	DDX6	0.642185	0.726	0.295	8.94E-59	1.30E-54
M2a like TAM	MYH9	0.641903	0.858	0.528	1.38E-39	2.01E-35
M2a like TAM	ABHD5	0.641876	0.522	0.291	8.36E-19	1.22E-14
M2a like TAM	SASH1	0.641221	0.599	0.21	7.18E-58	1.04E-53
M2a like TAM	POU2F2	0.640507	0.518	0.183	1.08E-47	1.57E-43
M2a like TAM	FAM49A	0.639646	0.803	0.389	1.40E-47	2.04E-43
M2a like TAM	CCDC50	0.639034	0.69	0.3	2.29E-51	3.33E-47
M2a like TAM	GALNT1	0.639009	0.785	0.409	1.39E-43	2.02E-39
M2a like TAM	KTN1	0.63842	0.934	0.587	2.16E-46	3.14E-42
M2a like TAM	RGS10	0.637342	0.982	0.771	8.79E-39	1.28E-34
M2a like TAM	RPN2	0.634639	0.982	0.68	2.25E-40	3.28E-36
M2a like TAM	DOCK2	0.634122	0.781	0.387	5.58E-47	8.12E-43
M2a like TAM	PHC2	0.633563	0.821	0.365	1.97E-58	2.87E-54
M2a like TAM	MXD1	0.631919	0.704	0.414	1.26E-24	1.83E-20
M2a like TAM	ATM	0.631918	0.697	0.307	1.95E-47	2.84E-43
M2a like TAM	WASF2	0.631744	0.876	0.52	8.43E-42	1.23E-37
M2a like TAM	ZMAT3	0.629756	0.504	0.156	1.17E-57	1.70E-53
M2a like TAM	CLN8	0.629653	0.788	0.398	5.01E-44	7.28E-40
M2a like TAM	GRN	0.629232	1	0.902	4.58E-47	6.67E-43
M2a like TAM	FAM53C	0.6292	0.591	0.259	4.23E-38	6.15E-34
M2a like TAM	MYO1F	0.628504	0.792	0.44	2.75E-40	4.00E-36
M2a like TAM	SF3B1	0.627321	0.865	0.494	1.92E-44	2.79E-40
M2a like TAM	PCM1	0.626173	0.73	0.317	2.21E-52	3.22E-48
M2a like TAM	LPAR5	0.625014	0.511	0.111	1.77E-93	2.57E-89
M2a like TAM	PAPOLG	0.624231	0.522	0.124	5.72E-87	8.32E-83
M2a like TAM	TBC1D4	0.621206	0.496	0.106	9.55E-93	1.39E-88
M2a like TAM	KMT2C	0.620999	0.719	0.306	9.76E-53	1.42E-48
M2a like TAM	SNX9	0.620186	0.712	0.345	1.61E-44	2.35E-40
M2a like TAM	DOCK10	0.620142	0.759	0.345	9.93E-50	1.44E-45
M2a like TAM	EIF3L	0.619852	0.887	0.518	1.04E-43	1.52E-39
M2a like TAM	FAM133B	0.619479	0.752	0.394	5.71E-42	8.31E-38
M2a like TAM	PIK3C2A	0.61815	0.606	0.186	4.53E-72	6.59E-68
M2a like TAM	ZDHHC14	0.618003	0.445	0.095	3.41E-85	4.96E-81

M2a like TAM	OSBPL1A	0.617696	0.646	0.266	2.45E-50	3.56E-46
M2a like TAM	PIK3R5	0.617322	0.584	0.173	1.56E-73	2.27E-69
M2a like TAM	EBI3	0.617258	0.496	0.111	1.47E-86	2.14E-82
M2a like TAM	AMD1	0.617037	0.777	0.418	4.31E-40	6.27E-36
M2a like TAM	FAM105A	0.616575	0.818	0.407	8.02E-47	1.17E-42
M2a like TAM	EIF5	0.616322	0.967	0.587	6.29E-46	9.16E-42
M2a like TAM	HP1BP3	0.615781	0.763	0.376	9.25E-47	1.35E-42
M2a like TAM	HES1	0.615089	0.438	0.183	5.11E-27	7.44E-23
M2a like TAM	SSH2	0.614713	0.599	0.197	5.59E-65	8.14E-61
M2a like TAM	PSME4	0.613316	0.628	0.214	1.66E-62	2.41E-58
M2a like TAM	HGSNAT	0.61224	0.65	0.24	3.29E-59	4.79E-55
M2a like TAM	ELF1	0.611777	0.891	0.501	1.28E-46	1.86E-42
M2a like TAM	AKNA	0.609667	0.588	0.183	2.62E-67	3.82E-63
M2a like TAM	FCER1A	0.608629	0.241	0.021	2.86E-118	4.16E-114
M2a like TAM	SRRM2	0.607915	0.898	0.529	2.48E-42	3.61E-38
M2a like TAM	HLA-E	0.606486	0.996	0.878	9.58E-46	1.39E-41
M2a like TAM	DOCK8	0.606113	0.828	0.42	4.30E-45	6.26E-41
M2a like TAM	BPTF	0.606106	0.708	0.308	1.66E-50	2.42E-46
M2a like TAM	PTPRJ	0.605813	0.737	0.368	5.67E-43	8.25E-39
M2a like TAM	ITSN1	0.605398	0.518	0.137	1.46E-74	2.12E-70
M2a like TAM	SLC9A9	0.604774	0.482	0.116	3.97E-76	5.78E-72
M2a like TAM	ARAP1	0.604713	0.646	0.274	7.61E-48	1.11E-43
M2a like TAM	ATXN1	0.60422	0.682	0.295	9.88E-47	1.44E-42
M2a like TAM	MLXIP	0.603923	0.635	0.219	3.15E-64	4.58E-60
M2a like TAM	IL6ST	0.603612	0.65	0.274	1.42E-46	2.06E-42
M2a like TAM	NFE2L2	0.603313	0.894	0.565	2.07E-36	3.01E-32
M2a like TAM	SPIN1	0.603278	0.602	0.226	5.31E-54	7.73E-50
M2a like TAM	TK2	0.603159	0.628	0.199	1.79E-71	2.60E-67
M2a like TAM	HERC1	0.601639	0.639	0.233	7.09E-59	1.03E-54
M2a like TAM	SYAP1	0.601389	0.65	0.336	4.90E-31	7.13E-27
M2a like TAM	MAP3K2	0.600407	0.825	0.413	1.74E-47	2.52E-43
M2a like TAM	CAMK1D	0.597805	0.591	0.18	2.98E-69	4.34E-65
M2a like TAM	ENPP2	0.597768	0.734	0.329	2.52E-41	3.67E-37
M2a like TAM	RNASET2	0.597747	0.989	0.763	1.42E-32	2.07E-28
M2a like TAM	ACSL1	0.597107	0.697	0.335	2.42E-36	3.52E-32
M2a like TAM	IL16	0.5937	0.529	0.151	1.67E-68	2.43E-64
M2a like TAM	LPP	0.591949	0.741	0.358	4.72E-43	6.87E-39
M2a like TAM	SCAMP2	0.591735	0.898	0.526	3.80E-42	5.53E-38
M2a like TAM	TLR4	0.590706	0.745	0.382	3.07E-38	4.47E-34
M2a like TAM	MED13L	0.59025	0.609	0.205	1.90E-62	2.77E-58
M2a like TAM	SCAF11	0.590019	0.905	0.558	1.59E-38	2.31E-34

M2a like TAM	TLN1	0.589962	0.901	0.536	3.21E-40	4.67E-36
M2a like TAM	MAPKAPK2	0.589924	0.675	0.305	1.18E-43	1.72E-39
M2a like TAM	GNAQ	0.589061	0.883	0.459	4.05E-45	5.89E-41
M2a like TAM	MAP4K4	0.588044	0.438	0.091	5.22E-86	7.60E-82
M2a like TAM	ZNF106	0.588039	0.737	0.365	1.24E-42	1.80E-38
M2a like TAM	TRPS1	0.58803	0.577	0.234	1.90E-42	2.76E-38
M2a like TAM	ABCC4	0.58788	0.442	0.07	2.85E-118	4.15E-114
M2a like TAM	SRSF5	0.586269	0.931	0.6	3.73E-38	5.43E-34
M2a like TAM	SLC43A2	0.585014	0.723	0.361	1.28E-37	1.86E-33
M2a like TAM	MGAT5	0.584839	0.518	0.159	2.24E-60	3.26E-56
M2a like TAM	RNASE6	0.584145	0.989	0.736	1.29E-36	1.88E-32
M2a like TAM	APPL1	0.583724	0.748	0.359	2.20E-44	3.20E-40
M2a like TAM	NKTR	0.583528	0.65	0.268	7.95E-49	1.16E-44
M2a like TAM	TP53INP1	0.581928	0.482	0.107	4.31E-86	6.27E-82
M2a like TAM	IRF2BP2	0.581553	0.825	0.475	5.97E-36	8.68E-32
M2a like TAM	ZCCHC11	0.58139	0.54	0.149	1.44E-72	2.09E-68
M2a like TAM	NCOA3	0.581232	0.602	0.217	3.64E-55	5.29E-51
M2a like TAM	CD302	0.581168	0.821	0.412	3.26E-44	4.74E-40
M2a like TAM	AHCYL1	0.580306	0.697	0.366	3.10E-37	4.52E-33
M2a like TAM	GCLC	0.580171	0.675	0.272	2.96E-49	4.31E-45
M2a like TAM	USP36	0.580056	0.504	0.168	7.46E-51	1.09E-46
M2a like TAM	TPCN1	0.579558	0.551	0.173	2.04E-61	2.96E-57
M2a like TAM	SAMSN1	0.57918	0.887	0.619	8.82E-29	1.28E-24
M2a like TAM	CH25H	0.57896	0.215	0.074	1.89E-18	2.75E-14
M2a like TAM	SLC38A10	0.57877	0.635	0.256	2.33E-50	3.39E-46
M2a like TAM	WNT5A	0.577921	0.401	0.098	5.34E-61	7.78E-57
M2a like TAM	HCLS1	0.577613	0.931	0.618	5.48E-38	7.97E-34
M2a like TAM	SERPINB9	0.577317	0.719	0.381	6.33E-33	9.21E-29
M2a like TAM	CHD1	0.576803	0.573	0.255	1.33E-37	1.93E-33
M2a like TAM	FAM120A	0.576735	0.847	0.471	3.78E-40	5.51E-36
M2a like TAM	CASS4	0.576459	0.427	0.091	1.66E-79	2.42E-75
M2a like TAM	CCNH	0.576389	0.661	0.268	1.49E-51	2.17E-47
M2a like TAM	PNN	0.573899	0.686	0.294	1.07E-48	1.55E-44
M2a like TAM	HOOK3	0.572502	0.712	0.329	3.66E-43	5.32E-39
M2a like TAM	ARID1A	0.570229	0.715	0.325	4.90E-46	7.14E-42
M2a like TAM	MFSD1	0.568726	0.989	0.764	5.91E-38	8.60E-34
M2a like TAM	TM6SF1	0.56844	0.712	0.324	8.02E-46	1.17E-41
M2a like TAM	PRKAB1	0.568032	0.423	0.075	1.77E-101	2.57E-97
M2a like TAM	SH3KBP1	0.565464	0.741	0.393	5.86E-38	8.52E-34
M2a like TAM	AKAP11	0.56421	0.555	0.187	1.46E-56	2.12E-52

M2a like TAM	GLS	0.562839	0.723	0.324	1.58E-45	2.31E-41
M2a like TAM	ELL2	0.561274	0.558	0.299	3.40E-22	4.94E-18
M2a like TAM	B4GALT1	0.559567	0.799	0.425	1.29E-37	1.88E-33
M2a like TAM	PTGS2	0.559463	0.394	0.188	4.84E-18	7.05E-14
M2a like TAM	EP300	0.559195	0.631	0.241	1.36E-51	1.98E-47
M2a like TAM	NCOR2	0.558921	0.639	0.261	5.43E-48	7.91E-44
M2a like TAM	ELMO1	0.557943	0.595	0.199	8.92E-59	1.30E-54
M2a like TAM	TBC1D12	0.557675	0.474	0.111	4.60E-80	6.69E-76
M2a like TAM	HGF	0.557554	0.416	0.087	1.26E-76	1.83E-72
M2a like TAM	PHF20	0.556772	0.682	0.292	4.69E-47	6.82E-43
M2a like TAM	PTPRE	0.556029	0.796	0.478	4.00E-26	5.82E-22
M2a like TAM	PRKCSH	0.555823	0.777	0.363	6.67E-50	9.71E-46
M2a like TAM	SON	0.555355	0.942	0.651	7.05E-34	1.03E-29
M2a like TAM	FCHSD2	0.555117	0.518	0.175	2.80E-52	4.07E-48
M2a like TAM	LARP1	0.554228	0.675	0.346	6.77E-36	9.85E-32
M2a like TAM	EFCAB14	0.553393	0.755	0.37	7.04E-43	1.02E-38
M2a like TAM	DCP2	0.552818	0.631	0.268	6.78E-44	9.87E-40
M2a like TAM	FABP1	0.55246	0.449	0.089	2.98E-87	4.34E-83
M2a like TAM	AFF4	0.552334	0.682	0.318	1.33E-42	1.94E-38
M2a like TAM	TBC1D9	0.551995	0.609	0.239	5.67E-49	8.25E-45
M2a like TAM	CSDE1	0.55074	0.927	0.64	2.59E-37	3.77E-33
M2a like TAM	EIF3A	0.547662	0.894	0.541	1.18E-35	1.72E-31
M2a like TAM	N4BP2L2	0.547457	0.909	0.542	2.04E-38	2.96E-34
M2a like TAM	CYSLTR1	0.546496	0.54	0.167	2.86E-61	4.16E-57
M2a like TAM	PDE4B	0.546069	0.693	0.296	2.57E-45	3.74E-41
M2a like TAM	KCNE1	0.545532	0.361	0.14	6.33E-27	9.20E-23
M2a like TAM	RIN3	0.545183	0.661	0.301	1.77E-42	2.58E-38
M2a like TAM	PRPF4B	0.545133	0.675	0.311	1.07E-41	1.56E-37
M2a like TAM	ATP1A1	0.544879	0.872	0.537	4.18E-34	6.09E-30
M2a like TAM	SESN2	0.543459	0.38	0.108	3.06E-47	4.45E-43
M2a like TAM	PRKACB	0.542918	0.599	0.237	8.02E-47	1.17E-42
M2a like TAM	EEF1A1	0.542247	1	0.988	2.85E-50	4.15E-46
M2a like TAM	RND3	0.542127	0.296	0.08	3.44E-37	5.00E-33
M2a like TAM	STARD7	0.54097	0.708	0.37	3.21E-36	4.68E-32
M2a like TAM	PRKDC	0.536547	0.697	0.306	8.40E-45	1.22E-40
M2a like TAM	EPS15	0.536259	0.661	0.292	2.71E-42	3.94E-38
M2a like TAM	TRIM14	0.53493	0.58	0.262	1.54E-36	2.23E-32
M2a like TAM	ARHGEF6	0.534221	0.551	0.191	1.04E-52	1.51E-48
M2a like TAM	FAM53B	0.533073	0.471	0.107	3.00E-81	4.36E-77
M2a like TAM	IER5L	0.531651	0.515	0.18	1.13E-46	1.64E-42
M2a like TAM	RBM39	0.531149	0.909	0.572	2.20E-32	3.20E-28

M2a like TAM	ST8SIA4	0.531116	0.763	0.385	3.69E-39	5.37E-35
M2a like TAM	RREB1	0.53075	0.609	0.285	7.12E-34	1.04E-29
M2a like TAM	CXCL12	0.530486	0.332	0.064	6.22E-66	9.05E-62
M2a like TAM	MBP	0.529456	0.664	0.311	2.54E-38	3.69E-34
M2a like TAM	IPO7	0.529148	0.693	0.301	2.22E-47	3.23E-43
M2a like TAM	TBL1XR1	0.528443	0.777	0.364	1.10E-44	1.61E-40
M2a like TAM	LARP4B	0.52835	0.588	0.223	1.09E-50	1.58E-46
M2a like TAM	KAT6A	0.527424	0.588	0.182	4.75E-66	6.91E-62
M2a like TAM	SLC8A1	0.527212	0.785	0.425	1.97E-34	2.87E-30
M2a like TAM	TIAM1	0.527177	0.504	0.136	1.85E-68	2.69E-64
M2a like TAM	DENND3	0.525774	0.62	0.268	1.84E-41	2.67E-37
M2a like TAM	PHF3	0.525153	0.599	0.244	3.66E-44	5.33E-40
M2a like TAM	RCC2	0.525095	0.577	0.239	5.73E-41	8.34E-37
M2a like TAM	HOMER3	0.523102	0.566	0.215	1.35E-46	1.96E-42
M2a like TAM	LAT2	0.522777	0.774	0.403	2.11E-36	3.07E-32
M2a like TAM	PHKB	0.522226	0.661	0.271	4.78E-48	6.95E-44
M2a like TAM	ZBTB4	0.521193	0.569	0.197	1.97E-53	2.86E-49
M2a like TAM	PRMT2	0.520099	0.858	0.491	8.71E-37	1.27E-32
M2a like TAM	TBC1D14	0.519843	0.522	0.185	4.47E-47	6.51E-43
M2a like TAM	SNX29	0.519519	0.547	0.212	2.67E-42	3.88E-38
M2a like TAM	IDS	0.519178	0.712	0.414	4.16E-27	6.05E-23
M2a like TAM	VEGFB	0.518573	0.741	0.363	3.66E-39	5.33E-35
M2a like TAM	CANX	0.51796	0.978	0.756	2.56E-27	3.72E-23
M2a like TAM	HDAC9	0.517289	0.332	0.022	2.76E-209	4.02E-205
M2a like TAM	SENP6	0.516947	0.573	0.228	4.26E-44	6.21E-40
M2a like TAM	ADRBK2	0.51682	0.628	0.264	1.55E-42	2.26E-38
M2a like TAM	CSNK1D	0.516808	0.664	0.302	1.22E-42	1.77E-38
M2a like TAM	PKIB	0.516737	0.485	0.152	3.14E-47	4.57E-43
M2a like TAM	PTGER4	0.51655	0.58	0.276	7.50E-29	1.09E-24
M2a like TAM	GAA	0.514766	0.828	0.437	2.32E-36	3.37E-32
M2a like TAM	SLC7A8	0.514173	0.664	0.322	1.24E-32	1.81E-28
M2a like TAM	ALOX5	0.51282	0.741	0.398	1.12E-31	1.64E-27
M2a like TAM	TMC8	0.512444	0.427	0.142	7.62E-44	1.11E-39
M2a like TAM	HMHA1	0.512423	0.635	0.248	1.37E-50	1.99E-46
M2a like TAM	ASH1L	0.512193	0.591	0.238	2.94E-43	4.28E-39
M2a like TAM	ITPRIPL2	0.511319	0.653	0.295	2.10E-39	3.06E-35
M2a like TAM	CAT	0.510866	0.796	0.426	4.03E-39	5.87E-35
M2a like TAM	RCSD1	0.510417	0.646	0.256	1.32E-47	1.93E-43
M2a like TAM	JMJD1C	0.509216	0.679	0.348	6.96E-32	1.01E-27
M2a like TAM	TCF7L2	0.50882	0.442	0.15	7.45E-43	1.08E-38
M2a like TAM	MACC1	0.508086	0.332	0.096	3.42E-39	4.98E-35

M2a like TAM	ERGIC1	0.507859	0.657	0.31	6.11E-37	8.89E-33
M2a like TAM	CYP4V2	0.507377	0.46	0.126	1.03E-61	1.50E-57
M2a like TAM	MAP3K5	0.506863	0.507	0.167	5.30E-51	7.71E-47
M2a like TAM	LMBRD1	0.506632	0.734	0.356	9.13E-39	1.33E-34
M2a like TAM	RNF149	0.506563	0.916	0.617	3.84E-30	5.58E-26
M2a like TAM	ARHGAP4	0.506219	0.715	0.349	3.15E-38	4.58E-34
M2a like TAM	SERPINF1	0.505151	0.558	0.213	9.38E-40	1.37E-35
M2a like TAM	CELF1	0.504684	0.785	0.432	8.32E-34	1.21E-29
M2a like TAM	BAIAP2	0.504311	0.464	0.135	6.75E-56	9.82E-52
M2a like TAM	ST3GAL1	0.503923	0.613	0.296	1.04E-31	1.51E-27
M2a like TAM	THOC2	0.503623	0.682	0.304	4.09E-42	5.96E-38
M2a like TAM	HNRNPA3	0.503179	0.971	0.716	4.53E-30	6.60E-26
M2a like TAM	ANTXR1	0.502971	0.332	0.05	5.79E-90	8.42E-86
M2a like TAM	IFNGR2	0.502426	0.934	0.641	1.79E-30	2.61E-26
M2a like TAM	PLXND1	0.501602	0.701	0.419	1.26E-24	1.83E-20
M2a like TAM	LDLRAD4	0.501304	0.504	0.171	5.64E-48	8.21E-44
M2a like TAM	FAM135A	0.501243	0.42	0.08	9.48E-89	1.38E-84
M2a like TAM	KLHL6	0.500773	0.547	0.241	3.35E-34	4.88E-30
M2a like TAM	PLEKHA1	0.500541	0.467	0.117	2.35E-70	3.42E-66
M2a like TAM	USF2	0.499897	0.803	0.475	3.06E-34	4.45E-30
M2a like TAM	CHKA	0.499746	0.46	0.128	1.07E-58	1.55E-54
M2a like TAM	SARAF	0.49823	0.985	0.79	4.00E-30	5.83E-26
M2a like TAM	CD28	0.497872	0.266	0.039	2.08E-75	3.03E-71
M2a like TAM	PLCB2	0.497692	0.518	0.181	2.21E-48	3.22E-44
M2a like TAM	TSPAN14	0.497425	0.734	0.372	4.25E-35	6.18E-31
M2a like TAM	BCLAF1	0.497424	0.723	0.386	7.11E-33	1.03E-28
M2a like TAM	GAK	0.497394	0.606	0.256	6.68E-42	9.71E-38
M2a like TAM	SCARB1	0.496654	0.504	0.166	7.15E-51	1.04E-46
M2a like TAM	EPHX1	0.496583	0.536	0.21	3.94E-40	5.73E-36
M2a like TAM	SELPLG	0.495288	0.599	0.281	2.05E-33	2.99E-29
M2a like TAM	SIDT1	0.49462	0.328	0.034	6.98E-137	1.02E-132
M2a like TAM	RHBDF2	0.494307	0.602	0.254	1.10E-39	1.60E-35
M2a like TAM	WNT5B	0.49406	0.328	0.02	4.57E-218	6.65E-214
M2a like TAM	PXDC1	0.493772	0.536	0.182	5.05E-52	7.34E-48
M2a like TAM	TGFB1	0.493433	0.901	0.535	2.51E-32	3.65E-28
M2a like TAM	IST1	0.492468	0.712	0.357	1.80E-35	2.62E-31
M2a like TAM	AOAH	0.492447	0.606	0.241	7.90E-43	1.15E-38
M2a like TAM	BRD4	0.492198	0.69	0.349	1.05E-35	1.52E-31
M2a like TAM	ARRB2	0.491566	0.898	0.611	1.13E-30	1.64E-26
M2a like TAM	SYK	0.491519	0.818	0.428	5.00E-38	7.27E-34

M2a like TAM	CNTRL	0.491381	0.511	0.177	4.21E-48	6.13E-44
M2a like TAM	SEMA4C	0.490698	0.387	0.076	1.27E-77	1.85E-73
M2a like TAM	CREB3L2	0.490434	0.65	0.305	5.71E-36	8.30E-32
M2a like TAM	ITSN2	0.489616	0.672	0.324	9.30E-37	1.35E-32
M2a like TAM	FARP1	0.489559	0.456	0.121	5.68E-61	8.27E-57
M2a like TAM	RAPGEF6	0.489301	0.38	0.055	1.73E-110	2.51E-106
M2a like TAM	ABHD17A	0.488492	0.642	0.253	1.13E-49	1.64E-45
M2a like TAM	POLR2A	0.487484	0.569	0.257	1.13E-33	1.65E-29
M2a like TAM	SGMS1	0.486924	0.485	0.16	2.73E-47	3.98E-43
M2a like TAM	BNC2	0.486684	0.328	0.055	1.17E-81	1.71E-77
M2a like TAM	GIT2	0.485777	0.628	0.314	1.07E-31	1.55E-27
M2a like TAM	MUM1	0.484739	0.464	0.127	6.44E-61	9.37E-57
M2a like TAM	PUM2	0.481425	0.628	0.254	6.36E-45	9.25E-41
M2a like TAM	PRKAR2A	0.47982	0.599	0.282	4.04E-32	5.88E-28
M2a like TAM	GPATCH2L	0.479194	0.584	0.219	3.63E-47	5.28E-43
M2a like TAM	ARL5A	0.479143	0.555	0.213	4.78E-43	6.96E-39
M2a like TAM	MAGT1	0.479045	0.704	0.38	7.05E-30	1.03E-25
M2a like TAM	LUC7L3	0.479016	0.766	0.39	1.19E-36	1.74E-32
M2a like TAM	EEF2	0.47861	0.974	0.76	2.52E-29	3.66E-25
M2a like TAM	ETNK1	0.478242	0.701	0.359	1.10E-33	1.60E-29
M2a like TAM	ELF2	0.478189	0.504	0.204	4.66E-37	6.77E-33
M2a like TAM	KANSL1	0.477805	0.624	0.262	1.05E-41	1.53E-37
M2a like TAM	PARP4	0.477712	0.551	0.208	9.90E-46	1.44E-41
M2a like TAM	CDK13	0.476141	0.518	0.201	5.84E-40	8.49E-36
M2a like TAM	SETD2	0.475802	0.507	0.162	2.42E-53	3.52E-49
M2a like TAM	CSF3R	0.475065	0.555	0.234	1.67E-37	2.42E-33
M2a like TAM	RB1CC1	0.474733	0.562	0.223	8.30E-41	1.21E-36
M2a like TAM	C16orf72	0.474624	0.624	0.299	2.19E-34	3.19E-30
M2a like TAM	ARHGAP5	0.474181	0.445	0.161	4.34E-37	6.31E-33
M2a like TAM	RCAN1	0.47407	0.573	0.283	1.29E-24	1.88E-20
M2a like TAM	P3H2	0.473734	0.325	0.041	4.32E-110	6.29E-106
M2a like TAM	ALPK3	0.473622	0.223	0.015	3.25E-137	4.74E-133
M2a like TAM	CHD7	0.473062	0.35	0.048	2.69E-107	3.92E-103
M2a like TAM	RSF1	0.472098	0.573	0.287	1.33E-28	1.93E-24
M2a like TAM	SEPN1	0.471903	0.551	0.207	1.47E-44	2.14E-40
M2a like TAM	SCN1B	0.471497	0.339	0.067	3.18E-68	4.62E-64
M2a like TAM	LACC1	0.471434	0.628	0.285	5.02E-36	7.31E-32
M2a like TAM	TRAK1	0.470711	0.438	0.139	5.34E-47	7.77E-43

M2a like TAM	AFF3	0.470335	0.259	0.009	4.57E-259	6.65E-255
M2a like TAM	MKLN1	0.470322	0.529	0.213	1.45E-37	2.11E-33
M2a like TAM	RBM26	0.470015	0.569	0.255	2.77E-34	4.03E-30
M2a like TAM	ANKRD9	0.469939	0.511	0.173	5.42E-49	7.88E-45
M2a like TAM	ZKSCAN1	0.469783	0.511	0.2	6.53E-38	9.50E-34
M2a like TAM	PKN2	0.469781	0.485	0.157	5.30E-50	7.72E-46
M2a like TAM	TRIP12	0.468948	0.701	0.352	3.21E-34	4.68E-30
M2a like TAM	KIAA0355	0.46856	0.42	0.09	2.04E-76	2.97E-72
M2a like TAM	LPCAT1	0.467929	0.515	0.205	1.28E-37	1.86E-33
M2a like TAM	RAPH1	0.467764	0.387	0.105	4.36E-50	6.34E-46
M2a like TAM	PHC3	0.467471	0.453	0.159	3.29E-41	4.79E-37
M2a like TAM	TMEM259	0.467395	0.635	0.302	7.76E-35	1.13E-30
M2a like TAM	HPS3	0.467318	0.562	0.238	2.51E-37	3.66E-33
M2a like TAM	PERP	0.466883	0.208	0.055	1.62E-26	2.35E-22
M2a like TAM	ACER3	0.464268	0.715	0.423	3.27E-26	4.76E-22
M2a like TAM	CUX1	0.464014	0.58	0.264	1.58E-33	2.30E-29
M2a like TAM	IREB2	0.46318	0.547	0.221	6.05E-39	8.80E-35
M2a like TAM	SNRNP200	0.463151	0.58	0.252	7.35E-37	1.07E-32
M2a like TAM	DIP2B	0.461719	0.489	0.17	2.70E-44	3.93E-40
M2a like TAM	BRD2	0.46162	0.719	0.4	3.72E-27	5.42E-23
M2a like TAM	FLI1	0.461075	0.558	0.244	4.19E-34	6.10E-30
M2a like TAM	ABR	0.460321	0.65	0.285	1.92E-40	2.80E-36
M2a like TAM	CHD2	0.460298	0.536	0.21	2.13E-40	3.09E-36
M2a like TAM	KPNA6	0.460088	0.54	0.227	1.60E-35	2.33E-31
M2a like TAM	ZMYND11	0.460023	0.493	0.163	1.05E-48	1.52E-44
M2a like TAM	RIF1	0.459881	0.54	0.214	3.84E-39	5.58E-35
M2a like TAM	PRKCA	0.459317	0.485	0.173	9.07E-42	1.32E-37
M2a like TAM	PTPN18	0.459184	0.734	0.369	2.36E-35	3.43E-31
M2a like TAM	SIPA1L2	0.459148	0.478	0.159	3.86E-47	5.61E-43
M2a like TAM	ATP7A	0.458491	0.434	0.124	3.84E-53	5.59E-49
M2a like TAM	SLC26A2	0.457981	0.449	0.168	7.56E-36	1.10E-31
M2a like TAM	CPNE1	0.457241	0.558	0.255	2.79E-33	4.06E-29
M2a like TAM	EPN1	0.456803	0.672	0.349	1.17E-30	1.70E-26
M2a like TAM	KIAA0100	0.456237	0.493	0.161	5.85E-50	8.52E-46
M2a like TAM	AP2B1	0.456176	0.639	0.31	2.12E-34	3.08E-30
M2a like TAM	SIGLEC12	0.456113	0.504	0.154	9.25E-54	1.35E-49
M2a like TAM	MTSS1	0.455724	0.467	0.185	3.49E-33	5.07E-29
M2a like TAM	GCC2	0.455505	0.628	0.288	1.52E-35	2.22E-31
M2a like TAM	OGT	0.454909	0.533	0.197	3.10E-44	4.51E-40
M2a like TAM	NFIL3	0.454105	0.478	0.227	2.62E-24	3.81E-20
M2a like TAM	SMURF2	0.452863	0.54	0.205	1.93E-41	2.81E-37

M2a like TAM	RAPGEF1	0.452673	0.664	0.343	1.24E-28	1.80E-24
M2a like TAM	CACUL1	0.452502	0.693	0.339	4.00E-34	5.83E-30
M2a like TAM	PLEKHA2	0.452489	0.682	0.377	1.14E-25	1.66E-21
M2a like TAM	SMARCA2	0.451326	0.606	0.274	1.50E-35	2.18E-31
M2a like TAM	ATF7IP	0.450276	0.493	0.184	1.36E-39	1.98E-35
M2a like TAM	IFRD1	0.450237	0.584	0.282	2.08E-28	3.03E-24
M2a like TAM	TAOK3	0.449823	0.814	0.487	1.19E-27	1.74E-23
M2a like TAM	TNPO1	0.449068	0.668	0.353	6.78E-29	9.87E-25
M2a like TAM	ASXL2	0.449058	0.529	0.202	1.23E-41	1.79E-37
M2a like TAM	KIAA0141	0.44888	0.434	0.143	2.06E-44	3.00E-40
M2a like TAM	HEATR3	0.448122	0.409	0.119	1.32E-48	1.92E-44
M2a like TAM	PIAS1	0.44557	0.675	0.337	1.96E-32	2.85E-28
M2a like TAM	CCNG2	0.445371	0.511	0.18	5.40E-45	7.86E-41
M2a like TAM	LPCAT2	0.444508	0.741	0.37	3.23E-31	4.70E-27
M2a like TAM	PLA2G16	0.444157	0.445	0.119	3.12E-57	4.54E-53
M2a like TAM	SEL1L	0.44373	0.661	0.311	6.47E-35	9.42E-31
M2a like TAM	ICOSLG	0.443228	0.387	0.11	4.45E-47	6.48E-43
M2a like TAM	HIPK1	0.442978	0.609	0.262	1.07E-38	1.56E-34
M2a like TAM	KMT2A	0.442921	0.485	0.174	1.39E-41	2.02E-37
M2a like TAM	FGD2	0.442777	0.664	0.291	5.16E-40	7.51E-36
M2a like TAM	TNFRSF11A	0.442723	0.314	0.056	3.12E-70	4.55E-66
M2a like TAM	CNOT6L	0.442622	0.522	0.194	5.61E-43	8.17E-39
M2a like TAM	SAMD4B	0.441738	0.511	0.177	1.32E-46	1.92E-42
M2a like TAM	TNFRSF21	0.441634	0.613	0.293	3.99E-31	5.81E-27
M2a like TAM	SMARCA5	0.441368	0.682	0.345	9.48E-31	1.38E-26
M2a like TAM	TRIM38	0.44102	0.613	0.29	2.72E-32	3.95E-28
M2a like TAM	GOLGA2	0.440608	0.434	0.157	1.57E-37	2.28E-33
M2a like TAM	C6orf48	0.440146	0.73	0.371	4.99E-34	7.26E-30
M2a like TAM	STARD13	0.439616	0.365	0.081	9.13E-63	1.33E-58
M2a like TAM	TNFRSF1A	0.439216	0.861	0.538	1.81E-26	2.63E-22
M2a like TAM	ARID3B	0.437999	0.361	0.089	3.85E-54	5.61E-50
M2a like TAM	LAPTM5	0.437663	1	0.924	1.45E-30	2.11E-26
M2a like TAM	SH3PXD2A	0.437546	0.387	0.113	2.28E-45	3.31E-41
M2a like TAM	NAV1	0.437457	0.401	0.132	1.08E-39	1.57E-35
M2a like TAM	CNPY3	0.437401	0.905	0.57	1.73E-29	2.52E-25
M2a like TAM	APAF1	0.437023	0.394	0.113	8.32E-48	1.21E-43
M2a like TAM	QPRT	0.437011	0.358	0.063	1.35E-80	1.96E-76
M2a like TAM	UFL1	0.436478	0.577	0.271	1.03E-31	1.50E-27
M2a like TAM	IL18	0.436366	0.796	0.436	1.09E-31	1.59E-27
M2a like TAM	PPP1R12A	0.435903	0.723	0.376	2.85E-30	4.15E-26
M2a like TAM	PHIP	0.435827	0.668	0.353	6.30E-27	9.16E-23

M2a like TAM	STX6	0.435444	0.602	0.257	4.29E-39	6.24E-35
M2a like TAM	FNIP2	0.434963	0.766	0.447	2.17E-24	3.16E-20
M2a like TAM	ZMIZ1	0.434824	0.631	0.325	6.41E-27	9.33E-23
M2a like TAM	FAM20C	0.434417	0.566	0.284	3.27E-24	4.77E-20
M2a like TAM	TAB2	0.434238	0.606	0.261	1.09E-37	1.58E-33
M2a like TAM	MKNK1	0.434217	0.766	0.432	2.49E-29	3.62E-25
M2a like TAM	SYNRG	0.433611	0.482	0.201	4.43E-32	6.45E-28
M2a like TAM	SIRT1	0.433344	0.401	0.115	2.39E-48	3.48E-44
M2a like TAM	CAMK2D	0.433321	0.496	0.188	3.05E-38	4.44E-34
M2a like TAM	ZC3H13	0.432484	0.613	0.324	2.02E-26	2.93E-22
M2a like TAM	HSD17B12	0.432348	0.741	0.402	1.88E-31	2.73E-27
M2a like TAM	EVI5	0.431245	0.518	0.202	2.30E-38	3.34E-34
M2a like TAM	LONP2	0.431178	0.555	0.219	3.41E-41	4.96E-37
M2a like TAM	TM9SF2	0.430719	0.88	0.519	6.66E-31	9.69E-27
M2a like TAM	ABCG1	0.430152	0.573	0.277	5.81E-28	8.46E-24
M2a like TAM	PCF11	0.430016	0.566	0.27	4.49E-28	6.53E-24
M2a like TAM	POLR2J3	0.429674	0.62	0.338	4.62E-24	6.72E-20
M2a like TAM	MEF2D	0.429189	0.438	0.138	2.87E-46	4.17E-42
M2a like TAM	PLK3	0.42915	0.54	0.252	3.15E-28	4.58E-24
M2a like TAM	WDR91	0.429075	0.405	0.1	5.27E-61	7.67E-57
M2a like TAM	PPP1R15B	0.42899	0.54	0.247	1.35E-29	1.97E-25
M2a like TAM	GPCPD1	0.428457	0.653	0.316	1.75E-31	2.54E-27
M2a like TAM	ARID4A	0.428057	0.584	0.25	3.27E-36	4.76E-32
M2a like TAM	HUWE1	0.427581	0.522	0.218	5.41E-35	7.88E-31
M2a like TAM	ARGLU1	0.427392	0.715	0.372	1.66E-31	2.42E-27
M2a like TAM	TIPARP	0.426573	0.398	0.149	4.62E-31	6.73E-27
M2a like TAM	LNPEP	0.426385	0.624	0.266	2.38E-38	3.47E-34
M2a like TAM	RNF150	0.426293	0.263	0.012	3.72E-222	5.42E-218
M2a like TAM	MB21D2	0.424803	0.423	0.128	1.64E-46	2.39E-42
M2a like TAM	CLCN7	0.424768	0.562	0.265	5.63E-30	8.19E-26
M2a like TAM	ZNF618	0.424708	0.361	0.103	6.68E-43	9.72E-39
M2a like TAM	WDR82	0.424452	0.628	0.307	1.07E-30	1.55E-26
M2a like TAM	TSPYL1	0.424441	0.599	0.238	1.44E-43	2.10E-39
M2a like TAM	SLC46A3	0.424198	0.474	0.176	4.14E-37	6.03E-33
M2a like TAM	AIG1	0.423774	0.547	0.219	8.52E-39	1.24E-34
M2a like TAM	USP22	0.42361	0.584	0.237	4.32E-41	6.28E-37
M2a like TAM	YWHAB	0.422598	0.993	0.779	9.98E-26	1.45E-21
M2a like TAM	R3HDM2	0.422491	0.496	0.183	4.46E-40	6.49E-36
M2a like TAM	TRIM44	0.422362	0.613	0.322	2.96E-26	4.30E-22
M2a like TAM	PSAP	0.421968	1	0.95	5.60E-26	8.14E-22
M2a like TAM	TANC2	0.421882	0.445	0.162	2.63E-36	3.83E-32

M2a like TAM	SORT1	0.421286	0.551	0.24	9.43E-33	1.37E-28
M2a like TAM	TTC14	0.420687	0.555	0.222	1.42E-38	2.07E-34
M2a like TAM	CTNNB1	0.420598	0.912	0.592	2.97E-30	4.32E-26
M2a like TAM	SNX13	0.419778	0.496	0.185	7.37E-40	1.07E-35
M2a like TAM	SNX5	0.419654	0.821	0.514	1.03E-25	1.50E-21
M2a like TAM	PRKACA	0.419612	0.515	0.226	2.19E-32	3.19E-28
M2a like TAM	ARIH1	0.419529	0.471	0.189	2.65E-33	3.86E-29
M2a like TAM	NR3C1	0.419257	0.646	0.336	1.68E-27	2.45E-23
M2a like TAM	MAMDC2	0.418679	0.226	0.013	9.93E-159	1.44E-154
M2a like TAM	RAB3IL1	0.417822	0.361	0.097	2.27E-47	3.30E-43
M2a like TAM	HNRNPH3	0.417816	0.763	0.406	3.23E-32	4.70E-28
M2a like TAM	BTBD7	0.416716	0.434	0.146	1.28E-40	1.86E-36
M2a like TAM	ARSD	0.416607	0.427	0.138	4.13E-43	6.00E-39
M2a like TAM	EIF4G3	0.416249	0.507	0.189	7.93E-40	1.15E-35
M2a like TAM	USP33	0.415974	0.496	0.184	1.91E-40	2.77E-36
M2a like TAM	MAFF	0.415084	0.394	0.156	1.70E-26	2.47E-22
M2a like TAM	MSI2	0.414887	0.35	0.074	4.30E-64	6.25E-60
M2a like TAM	SPEN	0.413407	0.456	0.197	1.02E-27	1.48E-23
M2a like TAM	RC3H1	0.413261	0.431	0.148	1.71E-38	2.49E-34
M2a like TAM	IVD	0.413085	0.409	0.106	1.08E-57	1.57E-53
M2a like TAM	FAM174B	0.412813	0.307	0.045	3.51E-87	5.11E-83
M2a like TAM	ATF6B	0.412585	0.569	0.256	4.69E-33	6.82E-29
M2a like TAM	NET1	0.412459	0.299	0.063	2.48E-54	3.61E-50
M2a like TAM	KCNQ1	0.412445	0.332	0.077	2.72E-53	3.96E-49
M2a like TAM	BBX	0.412278	0.646	0.311	2.94E-31	4.28E-27
M2a like TAM	WDFY4	0.412118	0.442	0.134	5.44E-49	7.92E-45
M2a like TAM	MAVS	0.411037	0.456	0.154	2.16E-43	3.15E-39
M2a like TAM	CD2AP	0.410846	0.511	0.199	2.46E-38	3.58E-34
M2a like TAM	PTAFR	0.410602	0.759	0.456	4.16E-22	6.05E-18
M2a like TAM	BCL2	0.408708	0.387	0.125	2.50E-39	3.63E-35
M2a like TAM	ABHD2	0.408497	0.737	0.431	7.59E-22	1.10E-17
M2a like TAM	PRRC2A	0.408366	0.496	0.201	2.91E-35	4.24E-31
M2a like TAM	JMY	0.407995	0.314	0.059	1.00E-65	1.46E-61
M2a like TAM	SLC39A10	0.407845	0.456	0.163	5.10E-38	7.42E-34
M2a like TAM	RERE	0.40772	0.456	0.14	2.22E-49	3.23E-45
M2a like TAM	FBXO11	0.407103	0.478	0.202	2.29E-31	3.34E-27
M2a like TAM	TET3	0.407009	0.442	0.153	1.30E-39	1.88E-35
M2a like TAM	KIAA1109	0.40679	0.485	0.164	1.75E-44	2.54E-40
M2a like TAM	PRKX	0.40599	0.46	0.156	1.31E-42	1.91E-38
M2a like TAM	PLA2G15	0.405957	0.533	0.26	2.67E-25	3.88E-21
M2a like TAM	HDLBP	0.404868	0.693	0.418	6.46E-22	9.40E-18

M2a like TAM	PAPD5	0.404787	0.434	0.141	4.61E-43	6.71E-39
M2a like TAM	GNAI3	0.404214	0.825	0.515	5.84E-23	8.50E-19
M2a like TAM	NF1	0.403725	0.544	0.232	1.96E-33	2.86E-29
M2a like TAM	SMARCC1	0.403718	0.507	0.208	1.76E-34	2.56E-30
M2a like TAM	SKI	0.403038	0.434	0.15	1.69E-39	2.46E-35
M2a like TAM	GGA1	0.402926	0.69	0.354	8.53E-29	1.24E-24
M2a like TAM	PURA	0.402588	0.485	0.208	1.69E-30	2.46E-26
M2a like TAM	TMEM106A	0.402584	0.551	0.232	2.08E-34	3.02E-30
M2a like TAM	RRM2B	0.40251	0.478	0.166	8.32E-42	1.21E-37
M2a like TAM	LFNG	0.402439	0.365	0.137	1.01E-27	1.47E-23
M2a like TAM	YTHDF3	0.402273	0.562	0.246	2.31E-32	3.36E-28
M2a like TAM	SREK1	0.401939	0.588	0.287	3.76E-27	5.46E-23
M2a like TAM	TMEM245	0.401599	0.471	0.176	5.34E-37	7.77E-33
M2a like TAM	NFAM1	0.401538	0.504	0.205	2.19E-34	3.19E-30
M2a like TAM	TSC22D2	0.40139	0.438	0.135	4.09E-46	5.95E-42
M2a like TAM	CCDC88B	0.401262	0.383	0.112	6.24E-45	9.09E-41
M2a like TAM	BRWD1	0.400679	0.467	0.209	2.40E-26	3.50E-22
M2a like TAM	HNRNPH1	0.400054	0.887	0.554	8.65E-28	1.26E-23
M2a like TAM	CCNI	0.399839	0.985	0.769	8.02E-23	1.17E-18
M2a like TAM	MBD6	0.399393	0.38	0.11	5.35E-45	7.79E-41
M2a like TAM	PURB	0.399291	0.485	0.227	6.34E-25	9.23E-21
M2a like TAM	MGAT1	0.399245	0.916	0.668	2.96E-19	4.31E-15
M2a like TAM	TMEM123	0.398749	0.92	0.643	1.39E-22	2.03E-18
M2a like TAM	TTYH2	0.398605	0.358	0.082	2.01E-58	2.92E-54
M2a like TAM	TPR	0.398537	0.642	0.33	7.80E-28	1.14E-23
M2a like TAM	PIAS2	0.398505	0.412	0.164	1.26E-28	1.84E-24
M2a like TAM	DHX36	0.398134	0.661	0.352	2.50E-27	3.64E-23
M2a like TAM	ITGA6	0.398073	0.281	0.078	7.49E-35	1.09E-30
M2a like TAM	LRMP	0.397085	0.365	0.109	3.71E-41	5.40E-37
M2a like TAM	GANAB	0.396867	0.661	0.326	2.63E-30	3.83E-26
M2a like TAM	GNA12	0.396378	0.478	0.234	2.36E-22	3.44E-18
M2a like TAM	SLC25A36	0.396315	0.529	0.209	1.75E-37	2.54E-33
M2a like TAM	DNM2	0.396213	0.642	0.301	1.99E-34	2.90E-30
M2a like TAM	NBPF1	0.396018	0.423	0.133	2.30E-43	3.35E-39
M2a like TAM	2-Sep	0.395666	0.883	0.575	2.23E-25	3.24E-21
M2a like TAM	TBC1D5	0.39556	0.518	0.22	2.86E-33	4.16E-29
M2a like TAM	DIP2A	0.395289	0.405	0.145	9.58E-35	1.39E-30
M2a like TAM	TBC1D1	0.395012	0.533	0.244	2.47E-29	3.60E-25
M2a like TAM	RASA3	0.394422	0.405	0.118	9.30E-48	1.35E-43
M2a like TAM	TRIM28	0.394363	0.577	0.267	4.04E-31	5.89E-27
M2a like TAM	KLHL5	0.394203	0.526	0.218	5.49E-35	7.99E-31

M2a like TAM	RAB21	0.394116	0.653	0.351	2.22E-25	3.23E-21
M2a like TAM	DNAJB6	0.394067	0.894	0.631	1.44E-22	2.09E-18
M2a like TAM	RABEP1	0.393856	0.5	0.207	2.47E-33	3.59E-29
M2a like TAM	SPG7	0.393765	0.504	0.211	1.92E-32	2.80E-28
M2a like TAM	PCYOX1	0.393174	0.391	0.155	1.01E-27	1.47E-23
M2a like TAM	PAQR8	0.393131	0.391	0.133	8.30E-36	1.21E-31
M2a like TAM	ARHGAP12	0.392698	0.38	0.117	5.66E-41	8.24E-37
M2a like TAM	YPEL5	0.392172	0.664	0.388	1.85E-21	2.69E-17
M2a like TAM	EZR	0.391889	0.745	0.439	9.77E-23	1.42E-18
M2a like TAM	TRPM2	0.39178	0.485	0.185	1.57E-36	2.28E-32
M2a like TAM	DYRK1A	0.39121	0.449	0.169	9.28E-35	1.35E-30
M2a like TAM	TLR3	0.3908	0.281	0.045	4.69E-71	6.82E-67
M2a like TAM	CTTNBP2NL	0.390085	0.467	0.177	5.03E-36	7.32E-32
M2a like TAM	NAGA	0.390083	0.77	0.458	2.47E-23	3.59E-19
M2a like TAM	TBC1D15	0.389583	0.5	0.211	2.41E-31	3.51E-27
M2a like TAM	YY1	0.388512	0.69	0.419	1.73E-21	2.51E-17
M2a like TAM	HNRNPL	0.388248	0.566	0.254	8.85E-32	1.29E-27
M2a like TAM	CTDSP2	0.387673	0.599	0.275	8.33E-32	1.21E-27
M2a like TAM	ANKRD44	0.387521	0.504	0.237	3.70E-26	5.39E-22
M2a like TAM	RILPL2	0.387231	0.668	0.385	6.79E-22	9.88E-18
M2a like TAM	MBTPS1	0.387004	0.445	0.174	6.67E-32	9.71E-28
M2a like TAM	MIA3	0.386965	0.493	0.195	8.76E-35	1.27E-30
M2a like TAM	UPF1	0.386948	0.409	0.148	1.04E-34	1.51E-30
M2a like TAM	JOSD1	0.38694	0.493	0.237	1.39E-24	2.02E-20
M2a like TAM	CTSF	0.386113	0.343	0.069	2.34E-66	3.41E-62
M2a like TAM	GPR160	0.385907	0.478	0.188	1.11E-32	1.61E-28
M2a like TAM	NUMA1	0.38572	0.471	0.172	5.56E-38	8.10E-34
M2a like TAM	LYPLA1	0.385298	0.821	0.473	6.26E-30	9.10E-26
M2a like TAM	TRIP11	0.385122	0.471	0.194	7.83E-31	1.14E-26
M2a like TAM	SH3GL1	0.384914	0.412	0.161	8.35E-31	1.21E-26
M2a like TAM	SLC35E1	0.384789	0.46	0.176	1.33E-34	1.93E-30
M2a like TAM	ZCCHC6	0.384568	0.434	0.213	2.76E-20	4.02E-16
M2a like TAM	TNFSF12	0.384542	0.766	0.433	6.10E-27	8.88E-23
M2a like TAM	WAS	0.384333	0.73	0.414	8.26E-26	1.20E-21
M2a like TAM	IFI16	0.384308	0.931	0.645	2.94E-22	4.28E-18
M2a like TAM	DDX24	0.38423	0.763	0.464	4.81E-21	7.00E-17
M2a like TAM	CBL	0.384019	0.485	0.212	5.96E-28	8.67E-24
M2a like TAM	TMF1	0.383409	0.566	0.261	3.23E-29	4.70E-25
M2a like TAM	IBTK	0.383086	0.536	0.251	9.99E-29	1.45E-24
M2a like TAM	CAP1	0.382931	0.993	0.82	1.37E-20	1.99E-16
M2a like TAM	YIPF4	0.382445	0.664	0.328	4.77E-30	6.95E-26

M2a like TAM	ARHGEF40	0.38223	0.383	0.121	2.09E-39	3.05E-35
M2a like TAM	ABI2	0.382145	0.427	0.133	6.15E-45	8.96E-41
M2a like TAM	PHACTR4	0.381949	0.485	0.218	5.83E-27	8.49E-23
M2a like TAM	TPP2	0.381795	0.485	0.218	9.22E-27	1.34E-22
M2a like TAM	RALGDS	0.38173	0.547	0.264	5.49E-26	7.98E-22
M2a like TAM	EIF4H	0.381181	0.693	0.413	6.51E-22	9.47E-18
M2a like TAM	AGPAT5	0.38076	0.416	0.14	2.84E-39	4.13E-35
M2a like TAM	HNRNPC	0.380678	0.964	0.73	4.02E-21	5.85E-17
M2a like TAM	CEP68	0.380338	0.358	0.109	1.09E-38	1.59E-34
M2a like TAM	IRF8	0.380128	0.752	0.457	2.73E-21	3.97E-17
M2a like TAM	CNOT2	0.379892	0.591	0.281	1.76E-30	2.56E-26
M2a like TAM	TLR5	0.379881	0.387	0.123	2.28E-38	3.31E-34
M2a like TAM	RAB14	0.379679	0.799	0.453	1.61E-27	2.34E-23
M2a like TAM	ZNF609	0.379001	0.358	0.121	1.85E-32	2.69E-28
M2a like TAM	ZNF292	0.377584	0.511	0.23	1.03E-27	1.50E-23
M2a like TAM	SDCCAG8	0.3763	0.588	0.334	3.67E-20	5.34E-16
M2a like TAM	CHD9	0.374997	0.726	0.418	9.06E-26	1.32E-21
M2a like TAM	EIF4G1	0.374982	0.606	0.334	1.43E-22	2.09E-18
M2a like TAM	ZNF638	0.374155	0.551	0.243	1.04E-32	1.51E-28
M2a like TAM	IKBKB	0.373913	0.474	0.163	1.49E-42	2.17E-38
M2a like TAM	SLC29A1	0.373894	0.434	0.206	1.16E-21	1.68E-17
M2a like TAM	MBNL2	0.373512	0.412	0.14	7.92E-38	1.15E-33
M2a like TAM	PDXDC1	0.373484	0.555	0.225	1.32E-36	1.92E-32
M2a like TAM	TRIM8	0.373309	0.442	0.183	1.60E-28	2.33E-24
M2a like TAM	PTGS1	0.373137	0.566	0.297	2.37E-23	3.45E-19
M2a like TAM	GRAMD4	0.373088	0.42	0.161	6.74E-31	9.81E-27
M2a like TAM	MANBA	0.372963	0.617	0.303	2.34E-28	3.40E-24
M2a like TAM	RHOT1	0.372946	0.493	0.229	1.69E-24	2.46E-20
M2a like TAM	TTC37	0.372229	0.547	0.247	4.56E-30	6.64E-26
M2a like TAM	MED13	0.372162	0.533	0.232	1.67E-30	2.43E-26
M2a like TAM	RBBP6	0.371915	0.485	0.258	1.31E-18	1.90E-14
M2a like TAM	SLC2A13	0.371857	0.321	0.093	1.42E-37	2.07E-33
M2a like TAM	UNC93B1	0.371822	0.821	0.499	9.96E-23	1.45E-18
M2a like TAM	GNPTG	0.371355	0.478	0.248	9.25E-21	1.35E-16
M2a like TAM	CLSTN1	0.371221	0.401	0.151	3.11E-31	4.52E-27
M2a like TAM	TYK2	0.371204	0.401	0.145	2.27E-33	3.30E-29
M2a like TAM	OXA1L	0.370957	0.708	0.362	5.58E-29	8.13E-25
M2a like TAM	SEC24D	0.370945	0.533	0.241	1.97E-29	2.86E-25
M2a like TAM	FYTTD1	0.370885	0.62	0.325	5.63E-26	8.19E-22
M2a like TAM	TBC1D9B	0.370559	0.518	0.224	2.99E-31	4.35E-27
M2a like TAM	KCNK6	0.370448	0.544	0.264	1.56E-24	2.27E-20

M2a like TAM	ATP1B1	0.370423	0.62	0.324	2.15E-23	3.13E-19
M2a like TAM	ZNF451	0.37006	0.453	0.189	7.05E-29	1.03E-24
M2a like TAM	PDS5A	0.369707	0.562	0.28	5.03E-26	7.31E-22
M2a like TAM	FAM13A	0.369673	0.376	0.099	1.28E-49	1.86E-45
M2a like TAM	POGZ	0.369497	0.467	0.196	6.74E-30	9.81E-26
M2a like TAM	TAPBP	0.368846	0.927	0.647	1.09E-21	1.59E-17
M2a like TAM	LCOR	0.368721	0.533	0.232	2.29E-30	3.33E-26
M2a like TAM	HBP1	0.368629	0.493	0.236	2.63E-24	3.82E-20
M2a like TAM	USP7	0.368323	0.507	0.215	1.78E-31	2.59E-27
M2a like TAM	CTDNEP1	0.368104	0.759	0.457	1.26E-22	1.83E-18
M2a like TAM	ATXN2	0.367853	0.464	0.21	4.19E-25	6.10E-21
M2a like TAM	GIGYF2	0.367537	0.445	0.185	3.67E-29	5.34E-25
M2a like TAM	SECISBP2L	0.367506	0.46	0.162	8.66E-40	1.26E-35
M2a like TAM	KDM5C	0.367493	0.387	0.13	1.40E-36	2.04E-32
M2a like TAM	ZNRF2	0.367227	0.46	0.199	1.64E-26	2.38E-22
M2a like TAM	HAVCR2	0.367215	0.88	0.563	1.03E-21	1.50E-17
M2a like TAM	PIEZO1	0.367169	0.354	0.135	8.07E-27	1.17E-22
M2a like TAM	BAG6	0.367113	0.58	0.286	3.28E-27	4.77E-23
M2a like TAM	HEXA	0.367046	0.938	0.603	3.14E-24	4.58E-20
M2a like TAM	RHOBTB3	0.366416	0.482	0.211	1.27E-26	1.84E-22
M2a like TAM	KIAA2013	0.366228	0.569	0.249	6.44E-33	9.37E-29
M2a like TAM	GLG1	0.36607	0.613	0.301	1.78E-28	2.59E-24
M2a like TAM	ICE2	0.365994	0.412	0.147	6.06E-35	8.82E-31
M2a like TAM	ADD1	0.365465	0.566	0.27	1.05E-26	1.52E-22
M2a like TAM	IL6R	0.364848	0.482	0.219	2.65E-25	3.85E-21
M2a like TAM	KMT2E	0.36448	0.723	0.415	2.29E-23	3.33E-19
M2a like TAM	GAPVD1	0.364423	0.493	0.203	2.96E-32	4.31E-28
M2a like TAM	CLPTM1L	0.363993	0.595	0.286	6.97E-29	1.01E-24
M2a like TAM	CLINT1	0.363955	0.54	0.236	1.13E-30	1.64E-26
M2a like TAM	HEATR5A	0.363749	0.347	0.118	1.22E-31	1.77E-27
M2a like TAM	ARHGEF12	0.363645	0.42	0.138	1.85E-39	2.69E-35
M2a like TAM	FAM168A	0.36362	0.365	0.108	1.63E-41	2.37E-37
M2a like TAM	PTBP1	0.36351	0.686	0.374	4.31E-26	6.27E-22
M2a like TAM	SRGAP3	0.36342	0.38	0.108	2.01E-44	2.92E-40
M2a like TAM	CCNY	0.362999	0.518	0.223	6.92E-31	1.01E-26
M2a like TAM	UBR5	0.362804	0.474	0.192	2.68E-31	3.89E-27
M2a like TAM	DDX27	0.362671	0.485	0.217	2.62E-27	3.81E-23
M2a like TAM	UGGT1	0.362651	0.464	0.196	9.69E-29	1.41E-24
M2a like TAM	SPRY1	0.362592	0.193	0.048	2.69E-27	3.91E-23
M2a like TAM	CASC4	0.362507	0.599	0.282	1.24E-29	1.81E-25
M2a like TAM	PPIP5K2	0.362475	0.54	0.239	1.98E-30	2.88E-26

M2a like TAM	NFAT5	0.36233	0.409	0.157	1.92E-29	2.79E-25
M2a like TAM	ZFX	0.36214	0.431	0.159	4.19E-34	6.10E-30
M2a like TAM	SUGP2	0.362114	0.485	0.21	2.30E-28	3.35E-24
M2a like TAM	ARHGAP9	0.361991	0.482	0.218	8.51E-27	1.24E-22
M2a like TAM	NCOA1	0.361932	0.401	0.165	3.03E-26	4.41E-22
M2a like TAM	RBL2	0.361844	0.438	0.177	2.49E-29	3.63E-25
M2a like TAM	SPPL3	0.361571	0.536	0.244	2.77E-29	4.03E-25
M2a like TAM	EIF3F	0.361554	0.891	0.57	6.15E-24	8.95E-20
M2a like TAM	DISC1	0.361477	0.347	0.079	4.48E-57	6.52E-53
M2a like TAM	INF2	0.361411	0.369	0.178	9.46E-18	1.38E-13
M2a like TAM	CPQ	0.361154	0.682	0.367	4.57E-26	6.65E-22
M2a like TAM	METTL9	0.360921	0.599	0.26	5.00E-35	7.28E-31
M2a like TAM	LMAN1	0.360789	0.719	0.408	2.17E-23	3.15E-19
M2a like TAM	RASAL2	0.360522	0.383	0.157	1.22E-25	1.78E-21
M2a like TAM	VCPIP1	0.360123	0.464	0.186	3.93E-31	5.73E-27
M2a like TAM	KHSRP	0.360112	0.493	0.244	4.08E-22	5.94E-18
M2a like TAM	UBA1	0.360018	0.591	0.34	1.65E-20	2.40E-16
M2a like TAM	ZNF207	0.359345	0.77	0.49	6.41E-20	9.32E-16
M2a like TAM	ARHGAP1	0.358615	0.383	0.129	1.38E-35	2.00E-31
M2a like TAM	CYLD	0.358514	0.569	0.284	4.33E-25	6.30E-21
M2a like TAM	ETV5	0.358455	0.682	0.375	3.70E-23	5.39E-19
M2a like TAM	ATG4C	0.358157	0.536	0.223	3.14E-34	4.57E-30
M2a like TAM	SLC22A23	0.358148	0.274	0.027	9.12E-115	1.33E-110
M2a like TAM	ZNF791	0.357349	0.387	0.141	7.09E-31	1.03E-26
M2a like TAM	TMEM154	0.356264	0.288	0.045	1.19E-74	1.73E-70
M2a like TAM	WDR81	0.35626	0.434	0.139	5.70E-43	8.29E-39
M2a like TAM	TOR1AIP1	0.356236	0.555	0.243	2.31E-31	3.35E-27
M2a like TAM	SLC22A18	0.355809	0.529	0.262	3.91E-25	5.69E-21
M2a like TAM	FECH	0.355543	0.438	0.165	1.02E-33	1.48E-29
M2a like TAM	GALNT10	0.355044	0.372	0.115	3.09E-39	4.50E-35
M2a like TAM	OFD1	0.354528	0.42	0.143	2.28E-36	3.32E-32
M2a like TAM	PARVG	0.354275	0.704	0.37	3.20E-27	4.66E-23
M2a like TAM	U2SURP	0.353949	0.599	0.341	1.01E-19	1.47E-15
M2a like TAM	BIRC2	0.353667	0.58	0.265	5.94E-31	8.65E-27
M2a like TAM	YME1L1	0.35335	0.672	0.406	5.05E-19	7.34E-15
M2a like TAM	SF3A1	0.353131	0.496	0.216	1.90E-29	2.76E-25
M2a like TAM	PI4KA	0.352975	0.445	0.152	8.97E-40	1.31E-35
M2a like TAM	LRP10	0.352689	0.657	0.354	6.74E-25	9.81E-21
M2a like TAM	MAML2	0.352603	0.434	0.171	4.54E-30	6.61E-26
M2a like TAM	UBALD2	0.352447	0.606	0.332	9.08E-21	1.32E-16
M2a like TAM	TES	0.35219	0.471	0.235	1.89E-20	2.75E-16

M2a like TAM	SLC7A5	0.351609	0.292	0.099	1.93E-26	2.81E-22
M2a like TAM	SEL1L3	0.351602	0.445	0.177	5.73E-30	8.34E-26
M2a like TAM	PREPL	0.351588	0.369	0.112	1.02E-39	1.48E-35
M2a like TAM	LIMS1	0.351436	0.945	0.714	7.14E-16	1.04E-11
M2a like TAM	MARK2	0.351325	0.431	0.162	4.23E-33	6.15E-29
M2a like TAM	UBXN2B	0.351149	0.496	0.197	3.27E-34	4.76E-30
M2a like TAM	GAB3	0.350823	0.405	0.145	1.81E-33	2.63E-29
M2a like TAM	BEX4	0.349632	0.737	0.407	8.72E-26	1.27E-21
M2a like TAM	INPPL1	0.349132	0.423	0.179	2.69E-26	3.91E-22
M2a like TAM	AKT1	0.348593	0.464	0.21	1.62E-24	2.36E-20
M2a like TAM	XIAP	0.348191	0.562	0.309	2.00E-19	2.91E-15
M2a like TAM	TAF1D	0.348044	0.588	0.341	1.63E-18	2.37E-14
M2a like TAM	WDR6	0.347771	0.416	0.163	1.83E-29	2.66E-25
M2a like TAM	FRAT1	0.347368	0.303	0.077	2.44E-42	3.55E-38
M2a like TAM	NCOA7	0.347355	0.434	0.164	9.13E-33	1.33E-28
M2a like TAM	GSPT1	0.347337	0.682	0.411	3.79E-20	5.52E-16
M2a like TAM	UBE2G2	0.34731	0.529	0.252	9.54E-26	1.39E-21
M2a like TAM	C6orf89	0.34728	0.544	0.25	2.10E-28	3.05E-24
M2a like TAM	ARMCX3	0.346917	0.562	0.309	2.37E-19	3.45E-15
M2a like TAM	CORO7	0.346496	0.496	0.23	2.34E-25	3.41E-21
M2a like TAM	PPP1R9B	0.345752	0.485	0.228	1.02E-24	1.48E-20
M2a like TAM	QARS	0.345718	0.62	0.302	4.37E-29	6.36E-25
M2a like TAM	CD164	0.34571	0.953	0.748	5.75E-19	8.36E-15
M2a like TAM	BLNK	0.345699	0.409	0.138	1.34E-37	1.95E-33
M2a like TAM	BAZ1B	0.345542	0.493	0.244	4.49E-22	6.54E-18
M2a like TAM	NCKAP5	0.345445	0.223	0.02	7.73E-102	1.12E-97
M2a like TAM	SMARCA1	0.344662	0.175	0.003	1.39E-252	2.03E-248
M2a like TAM	EPHB2	0.344656	0.394	0.134	1.45E-34	2.10E-30
M2a like TAM	RBM25	0.344006	0.796	0.505	2.60E-18	3.78E-14
M2a like TAM	RUNX2	0.343898	0.288	0.062	8.35E-51	1.22E-46
M2a like TAM	MAN1A2	0.343559	0.445	0.192	7.26E-26	1.06E-21
M2a like TAM	CTNS	0.343268	0.35	0.126	1.70E-29	2.48E-25
M2a like TAM	RNF216	0.343037	0.354	0.127	3.90E-30	5.68E-26
M2a like TAM	CLK1	0.342782	0.715	0.406	3.60E-22	5.24E-18
M2a like TAM	SPECC1	0.342256	0.35	0.127	1.65E-28	2.41E-24
M2a like TAM	RBM12	0.341908	0.431	0.171	4.06E-30	5.91E-26
M2a like TAM	MEPCE	0.341682	0.354	0.129	1.50E-28	2.18E-24
M2a like TAM	GUSB	0.341674	0.745	0.44	1.42E-21	2.07E-17
M2a like TAM	TOP1	0.341386	0.807	0.488	4.03E-23	5.87E-19
M2a like TAM	RGL2	0.341318	0.339	0.113	5.77E-32	8.40E-28

M2a like TAM	NPEPPS	0.34102	0.558	0.255	3.35E-29	4.87E-25
M2a like TAM	SUCLG2	0.340859	0.602	0.292	1.42E-27	2.07E-23
M2a like TAM	OSBPL8	0.3408	0.748	0.416	5.56E-25	8.10E-21
M2a like TAM	SURF4	0.340583	0.686	0.427	7.89E-17	1.15E-12
M2a like TAM	AIDA	0.340392	0.544	0.269	1.62E-24	2.36E-20
M2a like TAM	PIK3CD	0.340364	0.347	0.097	1.51E-42	2.20E-38
M2a like TAM	SRSF6	0.340307	0.602	0.287	1.26E-29	1.84E-25
M2a like TAM	AQR	0.340179	0.391	0.156	1.93E-26	2.81E-22
M2a like TAM	TBC1D22A	0.339732	0.456	0.19	1.08E-28	1.57E-24
M2a like TAM	NSA2	0.339685	0.781	0.506	5.37E-17	7.81E-13
M2a like TAM	PLAGL1	0.339636	0.409	0.153	5.79E-31	8.42E-27
M2a like TAM	KLF3	0.338796	0.482	0.21	6.40E-28	9.32E-24
M2a like TAM	SLC12A7	0.338311	0.453	0.173	5.03E-32	7.32E-28
M2a like TAM	EPRS	0.338148	0.569	0.277	2.25E-25	3.27E-21
M2a like TAM	NHLRC3	0.337804	0.584	0.328	8.64E-20	1.26E-15
M2a like TAM	SLK	0.337273	0.478	0.199	3.10E-29	4.51E-25
M2a like TAM	FEZ2	0.337246	0.723	0.394	1.41E-26	2.05E-22
M2a like TAM	ATF4	0.337128	0.781	0.442	1.20E-26	1.74E-22
M2a like TAM	MPZL1	0.337079	0.551	0.271	1.12E-25	1.62E-21
M2a like TAM	SSR1	0.336825	0.901	0.639	1.16E-17	1.69E-13
M2a like TAM	NCF4	0.33577	0.752	0.424	5.62E-24	8.18E-20
M2a like TAM	ANKRD17	0.335523	0.489	0.218	7.34E-27	1.07E-22
M2a like TAM	NFIA	0.335176	0.259	0.048	5.15E-54	7.50E-50
M2a like TAM	CLPB	0.334589	0.409	0.153	6.84E-32	9.95E-28
M2a like TAM	CETN3	0.334581	0.336	0.12	2.86E-28	4.17E-24
M2a like TAM	CFAP97	0.334514	0.416	0.173	1.73E-26	2.51E-22
M2a like TAM	DCP1A	0.334198	0.431	0.16	2.22E-33	3.22E-29
M2a like TAM	DHRS7	0.333957	0.792	0.511	2.16E-18	3.14E-14
M2a like TAM	STK10	0.333776	0.391	0.167	1.15E-23	1.67E-19
M2a like TAM	GPBP1	0.333223	0.646	0.365	1.16E-21	1.68E-17
M2a like TAM	SNRK	0.333026	0.358	0.118	2.46E-33	3.58E-29
M2a like TAM	POU2F1	0.33292	0.383	0.113	2.49E-42	3.62E-38
M2a like TAM	NMT1	0.332838	0.588	0.304	7.74E-22	1.13E-17
M2a like TAM	FEM1B	0.332729	0.5	0.228	2.74E-26	3.98E-22
M2a like TAM	ATG7	0.332475	0.558	0.273	3.26E-26	4.74E-22
M2a like TAM	EMP2	0.332194	0.376	0.16	4.18E-21	6.08E-17
M2a like TAM	REPIN1	0.332189	0.485	0.22	2.21E-25	3.21E-21
M2a like TAM	RLIM	0.332126	0.423	0.174	1.25E-27	1.82E-23
M2a like TAM	SOS2	0.332068	0.38	0.139	6.56E-30	9.55E-26
M2a like TAM	TP53I13	0.331321	0.511	0.218	1.46E-30	2.12E-26
M2a like TAM	PHF10	0.331303	0.358	0.118	1.59E-33	2.32E-29

M2a like TAM	BMPR2	0.331263	0.493	0.197	3.21E-32	4.67E-28
M2a like TAM	CTTNBP2	0.330593	0.215	0.023	5.68E-83	8.26E-79
M2a like TAM	RASAL3	0.33056	0.361	0.135	5.12E-28	7.44E-24
M2a like TAM	DNM1L	0.330537	0.478	0.222	2.32E-24	3.37E-20
M2a like TAM	PQLC1	0.330257	0.489	0.235	4.46E-23	6.49E-19
M2a like TAM	RICTOR	0.32993	0.442	0.19	3.95E-25	5.74E-21
M2a like TAM	MITF	0.329541	0.529	0.27	1.66E-20	2.41E-16
M2a like TAM	XPO6	0.329495	0.478	0.207	1.04E-28	1.51E-24
M2a like TAM	UHRF2	0.329465	0.307	0.088	2.08E-35	3.02E-31
M2a like TAM	RBM6	0.329187	0.511	0.236	6.31E-26	9.18E-22
M2a like TAM	ALDH9A1	0.329058	0.518	0.276	4.35E-20	6.33E-16
M2a like TAM	VPS36	0.328933	0.547	0.275	9.96E-23	1.45E-18
M2a like TAM	TAF10	0.328732	0.609	0.336	9.78E-22	1.42E-17
M2a like TAM	CEPT1	0.328716	0.485	0.212	3.03E-28	4.41E-24
M2a like TAM	CREBBP	0.328393	0.449	0.186	6.03E-28	8.78E-24
M2a like TAM	STX7	0.328137	0.748	0.479	2.75E-18	4.01E-14
M2a like TAM	SCAMP5	0.32812	0.234	0.022	1.70E-106	2.48E-102
M2a like TAM	SMAD5	0.328026	0.416	0.154	3.25E-32	4.73E-28
M2a like TAM	STIM2	0.327935	0.358	0.116	1.85E-34	2.69E-30
M2a like TAM	SCRG1	0.327563	0.19	0.024	4.91E-63	7.14E-59
M2a like TAM	PPP2R5E	0.327097	0.504	0.257	6.30E-21	9.17E-17
M2a like TAM	VPS35	0.327028	0.858	0.572	1.84E-18	2.68E-14
M2a like TAM	NFATC3	0.326857	0.478	0.203	3.95E-29	5.75E-25
M2a like TAM	PHF20L1	0.326736	0.536	0.259	2.58E-24	3.75E-20
M2a like TAM	ARFIP1	0.326643	0.471	0.173	4.21E-37	6.13E-33
M2a like TAM	ICA1	0.326452	0.325	0.068	4.07E-58	5.92E-54
M2a like TAM	FLII	0.326393	0.547	0.294	3.07E-20	4.46E-16
M2a like TAM	TCERG1	0.326266	0.431	0.18	2.66E-26	3.87E-22
M2a like TAM	FOXRED2	0.325849	0.296	0.072	1.68E-43	2.44E-39
M2a like TAM	RBM23	0.325613	0.489	0.203	3.50E-30	5.09E-26
M2a like TAM	ZNF518A	0.32525	0.423	0.174	2.35E-26	3.42E-22
M2a like TAM	CARD8	0.325207	0.558	0.274	5.04E-26	7.33E-22
M2a like TAM	PNPO	0.325199	0.438	0.213	4.06E-21	5.91E-17
M2a like TAM	UBN2	0.324938	0.303	0.092	3.69E-32	5.37E-28
M2a like TAM	PKN1	0.324631	0.562	0.295	1.24E-22	1.81E-18
M2a like TAM	PGM2	0.324485	0.478	0.218	5.67E-25	8.25E-21
M2a like TAM	RSRC2	0.324287	0.726	0.412	1.68E-22	2.44E-18
M2a like TAM	DNAJC10	0.324244	0.624	0.296	3.21E-28	4.68E-24
M2a like TAM	FNBP4	0.324148	0.489	0.221	4.72E-26	6.87E-22
M2a like TAM	DIRC2	0.323665	0.398	0.147	8.91E-31	1.30E-26
M2a like TAM	CES2	0.323339	0.365	0.129	5.13E-31	7.47E-27

M2a like TAM	YPEL2	0.3232	0.38	0.133	5.91E-32	8.60E-28
M2a like TAM	RCOR1	0.322843	0.398	0.165	8.22E-25	1.20E-20
M2a like TAM	NDUFS1	0.322757	0.471	0.236	1.98E-20	2.88E-16
M2a like TAM	CHD6	0.322729	0.391	0.134	1.82E-34	2.65E-30
M2a like TAM	PTCD3	0.322632	0.391	0.147	1.21E-29	1.77E-25
M2a like TAM	UHRF1BP1	0.322539	0.394	0.155	6.59E-27	9.59E-23
M2a like TAM	SEC61A1	0.322151	0.639	0.358	1.21E-20	1.76E-16
M2a like TAM	ZC3H18	0.321635	0.398	0.143	1.65E-32	2.40E-28
M2a like TAM	CCDC47	0.321547	0.73	0.417	7.65E-22	1.11E-17
M2a like TAM	DOCK7	0.32133	0.412	0.158	2.38E-29	3.46E-25
M2a like TAM	MICAL1	0.321325	0.511	0.237	1.37E-25	1.99E-21
M2a like TAM	F11R	0.321223	0.453	0.217	5.97E-21	8.69E-17
M2a like TAM	STARD8	0.320549	0.318	0.08	2.86E-44	4.16E-40
M2a like TAM	SPG11	0.320406	0.46	0.218	8.31E-23	1.21E-18
M2a like TAM	DPP9	0.320342	0.431	0.193	1.17E-23	1.70E-19
M2a like TAM	SND1	0.320281	0.584	0.325	1.50E-19	2.19E-15
M2a like TAM	RAMP1	0.320168	0.219	0.027	1.41E-72	2.04E-68
M2a like TAM	KIF13B	0.320116	0.343	0.1	4.69E-39	6.82E-35
M2a like TAM	GOLGA3	0.319528	0.398	0.16	2.79E-26	4.06E-22
M2a like TAM	TAF5L	0.318749	0.299	0.089	5.40E-33	7.86E-29
M2a like TAM	SLC18B1	0.318529	0.347	0.1	4.77E-39	6.93E-35
M2a like TAM	ZNF189	0.318451	0.288	0.083	1.98E-33	2.88E-29
M2a like TAM	NINJ1	0.318002	0.861	0.623	1.54E-16	2.25E-12
M2a like TAM	NISCH	0.317923	0.504	0.216	3.02E-28	4.39E-24
M2a like TAM	CSNK2A1	0.317627	0.555	0.282	1.61E-23	2.34E-19
M2a like TAM	MMP2	0.317491	0.241	0.052	5.89E-42	8.57E-38
M2a like TAM	LARP7	0.317211	0.635	0.393	2.39E-15	3.48E-11
M2a like TAM	SLC23A2	0.316867	0.391	0.158	2.15E-26	3.13E-22
M2a like TAM	MON2	0.316849	0.347	0.122	2.98E-29	4.33E-25
M2a like TAM	ELK4	0.316669	0.416	0.17	5.54E-27	8.06E-23
M2a like TAM	GATAD2B	0.316625	0.376	0.131	2.17E-32	3.16E-28
M2a like TAM	SPTLC3	0.315931	0.215	0.025	3.17E-78	4.61E-74
M2a like TAM	SEC23IP	0.315249	0.431	0.195	5.30E-23	7.72E-19
M2a like TAM	AGO2	0.314733	0.372	0.15	2.32E-24	3.37E-20
M2a like TAM	PIKFYVE	0.314421	0.412	0.157	4.66E-30	6.78E-26
M2a like TAM	UBE4A	0.314361	0.474	0.21	2.09E-26	3.05E-22
M2a like TAM	PIK3CG	0.313674	0.314	0.091	3.37E-36	4.90E-32
M2a like TAM	SIAH2	0.31367	0.511	0.256	4.52E-21	6.58E-17
M2a like TAM	NCK2	0.313385	0.252	0.063	2.67E-36	3.89E-32
M2a like TAM	CLOCK	0.313256	0.35	0.142	8.53E-23	1.24E-18
M2a like TAM	TIA1	0.313189	0.449	0.188	7.94E-27	1.16E-22

M2a like TAM	LRRC58	0.312945	0.496	0.251	1.24E-20	1.81E-16
M2a like TAM	RAB20	0.312759	0.745	0.467	1.42E-17	2.07E-13
M2a like TAM	UBC	0.312729	1	0.883	4.90E-17	7.13E-13
M2a like TAM	NBL1	0.312686	0.314	0.094	7.45E-33	1.08E-28
M2a like TAM	THUMPD1	0.312645	0.442	0.21	6.99E-21	1.02E-16
M2a like TAM	SACM1L	0.312565	0.398	0.155	2.18E-28	3.18E-24
M2a like TAM	TOP2B	0.312529	0.496	0.23	1.05E-24	1.53E-20
M2a like TAM	ESYT1	0.31218	0.445	0.237	5.42E-17	7.89E-13
M2a like TAM	USP4	0.312171	0.431	0.198	2.48E-22	3.61E-18
M2a like TAM	RNPEPL1	0.311929	0.595	0.302	8.71E-23	1.27E-18
M2a like TAM	STK38L	0.311861	0.434	0.192	3.03E-23	4.41E-19
M2a like TAM	PI4K2A	0.311839	0.401	0.18	1.55E-21	2.25E-17
M2a like TAM	USP11	0.3115	0.35	0.149	2.52E-21	3.66E-17
M2a like TAM	ITCH	0.311409	0.427	0.164	1.40E-30	2.04E-26
M2a like TAM	GNAI1	0.311386	0.212	0.015	8.52E-122	1.24E-117
M2a like TAM	MSL1	0.311362	0.464	0.19	2.46E-29	3.58E-25
M2a like TAM	FBXW11	0.311289	0.456	0.187	3.67E-29	5.33E-25
M2a like TAM	MAEA	0.311249	0.387	0.159	1.09E-25	1.58E-21
M2a like TAM	SERINC5	0.311135	0.507	0.263	8.00E-19	1.16E-14
M2a like TAM	LRRC41	0.311062	0.423	0.171	1.88E-28	2.73E-24
M2a like TAM	EDEM1	0.310544	0.489	0.253	1.75E-19	2.55E-15
M2a like TAM	LTN1	0.310186	0.383	0.145	2.14E-28	3.11E-24
M2a like TAM	USP47	0.310091	0.423	0.193	8.98E-22	1.31E-17
M2a like TAM	GNG7	0.309996	0.27	0.039	2.80E-75	4.07E-71
M2a like TAM	MICAL2	0.309971	0.336	0.131	3.67E-23	5.34E-19
M2a like TAM	ADPRH	0.309638	0.409	0.157	3.11E-29	4.53E-25
M2a like TAM	SP100	0.309567	0.704	0.422	1.32E-18	1.93E-14
M2a like TAM	UBXN7	0.309413	0.365	0.138	5.51E-27	8.02E-23
M2a like TAM	TBC1D2B	0.309268	0.423	0.186	1.08E-23	1.57E-19
M2a like TAM	NCOR1	0.309234	0.675	0.39	2.33E-20	3.39E-16
M2a like TAM	EPM2AIP1	0.309037	0.35	0.137	5.16E-24	7.51E-20
M2a like TAM	DAP	0.308993	0.504	0.228	2.81E-26	4.08E-22
M2a like TAM	KDM2B	0.308927	0.38	0.16	3.90E-23	5.68E-19
M2a like TAM	THRAP3	0.308728	0.708	0.408	1.53E-20	2.22E-16
M2a like TAM	MAGED2	0.308719	0.467	0.207	1.09E-25	1.59E-21
M2a like TAM	EYA2	0.308338	0.23	0.015	9.47E-147	1.38E-142
M2a like TAM	PABPN1	0.30818	0.628	0.365	5.04E-18	7.34E-14
M2a like TAM	LRRK1	0.30809	0.376	0.122	1.96E-35	2.85E-31
M2a like TAM	CCDC170	0.307949	0.31	0.103	2.22E-28	3.23E-24
M2a like TAM	UBAC2	0.307453	0.485	0.264	1.67E-17	2.44E-13

M2a like TAM	TNFRSF10B	0.306719	0.339	0.108	1.03E-33	1.50E-29
M2a like TAM	TM9SF4	0.306716	0.398	0.157	6.59E-28	9.58E-24
M2a like TAM	ZDHHC7	0.306637	0.478	0.212	4.32E-26	6.29E-22
M2a like TAM	THADA	0.305931	0.314	0.094	5.65E-35	8.22E-31
M2a like TAM	FAM102B	0.305852	0.394	0.16	6.28E-25	9.13E-21
M2a like TAM	RNF115	0.305825	0.504	0.247	5.85E-23	8.52E-19
M2a like TAM	SIGLEC8	0.305767	0.212	0.021	3.76E-89	5.47E-85
M2a like TAM	MKNK2	0.305677	0.453	0.204	1.23E-23	1.79E-19
M2a like TAM	SMARCC2	0.305631	0.332	0.123	6.35E-26	9.24E-22
M2a like TAM	TMEM248	0.30555	0.661	0.404	3.50E-18	5.09E-14
M2a like TAM	ZBTB37	0.305499	0.292	0.086	7.56E-33	1.10E-28
M2a like TAM	ZSWIM8	0.305375	0.365	0.127	1.61E-31	2.35E-27
M2a like TAM	CLCN3	0.305226	0.412	0.166	1.19E-27	1.74E-23
M2a like TAM	SMCR8	0.304974	0.354	0.118	2.05E-32	2.99E-28
M2a like TAM	MGRN1	0.304894	0.409	0.17	9.51E-26	1.38E-21
M2a like TAM	APOBR	0.304686	0.464	0.252	5.76E-16	8.39E-12
M2a like TAM	PRKD3	0.304674	0.365	0.125	7.11E-32	1.03E-27
M2a like TAM	GHDC	0.304363	0.339	0.126	3.24E-26	4.71E-22
M2a like TAM	ANAPC5	0.304325	0.558	0.278	4.65E-23	6.77E-19
M2a like TAM	LRPPRC	0.304271	0.467	0.213	2.76E-24	4.02E-20
M2a like TAM	PPM1F	0.304226	0.361	0.126	2.17E-30	3.16E-26
M2a like TAM	ZADH2	0.303932	0.296	0.096	1.50E-28	2.18E-24
M2a like TAM	RGS12	0.303911	0.405	0.172	1.47E-23	2.14E-19
M2a like TAM	CSNK2A2	0.303895	0.38	0.149	1.51E-26	2.19E-22
M2a like TAM	PCMTD1	0.30335	0.493	0.232	7.50E-24	1.09E-19
M2a like TAM	PBX2	0.303309	0.343	0.133	9.58E-25	1.39E-20
M2a like TAM	LRP3	0.303254	0.237	0.036	4.41E-65	6.42E-61
M2a like TAM	DHX9	0.303051	0.573	0.284	2.77E-24	4.03E-20
M2a like TAM	PCBP2	0.303013	0.956	0.721	3.31E-17	4.81E-13
M2a like TAM	TNRC6A	0.302479	0.453	0.205	2.11E-23	3.08E-19
M2a like TAM	DIP2C	0.30188	0.208	0.017	2.51E-106	3.66E-102
M2a like TAM	UBE2H	0.301664	0.405	0.18	1.10E-21	1.60E-17
M2a like TAM	USO1	0.301578	0.533	0.296	1.29E-17	1.87E-13
M2a like TAM	HNRNPUL2	0.301558	0.518	0.27	2.31E-20	3.37E-16
M2a like TAM	SKIV2L2	0.301531	0.369	0.158	1.01E-21	1.48E-17
M2a like TAM	SRSF11	0.301346	0.737	0.499	3.10E-15	4.51E-11
M2a like TAM	SBNO1	0.30103	0.464	0.212	1.27E-23	1.85E-19
M2a like TAM	STAU2	0.301029	0.383	0.125	6.43E-36	9.36E-32
M2a like TAM	DENND4C	0.300711	0.423	0.171	7.46E-27	1.09E-22
M2a like TAM	TM7SF3	0.30051	0.504	0.251	9.38E-22	1.37E-17
M2a like TAM	MYO1D	0.300058	0.263	0.052	5.68E-51	8.27E-47

M2a like TAM	ACBD3	0.299889	0.489	0.252	1.24E-18	1.81E-14
M2a like TAM	BROX	0.299483	0.573	0.288	5.84E-24	8.49E-20
M2a like TAM	PTAR1	0.299453	0.391	0.146	1.53E-28	2.22E-24
M2a like TAM	GFM1	0.299434	0.423	0.19	1.49E-22	2.17E-18
M2a like TAM	SF1	0.298959	0.719	0.466	7.51E-17	1.09E-12
M2a like TAM	ZEB1	0.298781	0.175	0.016	4.94E-83	7.19E-79
M2a like TAM	C16orf58	0.298714	0.314	0.08	1.12E-43	1.62E-39
M2a like TAM	HADHA	0.298224	0.745	0.467	1.80E-17	2.62E-13
M2a like TAM	HIP1	0.298059	0.31	0.107	7.79E-27	1.13E-22
M2a like TAM	MPRIP	0.297434	0.325	0.112	2.02E-28	2.94E-24
M2a like TAM	NSD1	0.297252	0.431	0.214	2.21E-19	3.22E-15
M2a like TAM	SMAD4	0.297233	0.376	0.145	1.45E-26	2.11E-22
M2a like TAM	GGA2	0.297228	0.591	0.308	2.52E-23	3.67E-19
M2a like TAM	CIRBP	0.297196	0.942	0.687	1.90E-17	2.76E-13
M2a like TAM	ABHD15	0.29709	0.328	0.107	1.13E-31	1.64E-27
M2a like TAM	CREB1	0.296521	0.511	0.248	2.80E-22	4.07E-18
M2a like TAM	OGDH	0.296247	0.423	0.188	2.51E-23	3.65E-19
M2a like TAM	PDPR	0.29609	0.361	0.132	1.02E-28	1.49E-24
M2a like TAM	TCEAL3	0.295944	0.504	0.228	4.35E-26	6.33E-22
M2a like TAM	UACA	0.295699	0.27	0.07	1.97E-36	2.86E-32
M2a like TAM	EIF2S3	0.295698	0.704	0.409	3.88E-22	5.65E-18
M2a like TAM	GALNT2	0.295404	0.394	0.176	2.05E-21	2.98E-17
M2a like TAM	NDRG3	0.295318	0.394	0.126	9.55E-39	1.39E-34
M2a like TAM	ABCG2	0.295318	0.245	0.053	6.24E-42	9.07E-38
M2a like TAM	MCM3AP	0.295042	0.361	0.148	3.53E-23	5.14E-19
M2a like TAM	DFFA	0.2949	0.398	0.157	9.82E-28	1.43E-23
M2a like TAM	DEPTOR	0.294728	0.252	0.036	8.73E-73	1.27E-68
M2a like TAM	RMND5A	0.294489	0.398	0.131	7.73E-37	1.13E-32
M2a like TAM	GAPT	0.294469	0.226	0.055	3.58E-33	5.22E-29
M2a like TAM	CDC42BPB	0.294447	0.332	0.106	8.91E-33	1.30E-28
M2a like TAM	RPRD2	0.294426	0.318	0.113	1.48E-26	2.16E-22
M2a like TAM	CSNK1G2	0.294277	0.354	0.136	9.06E-26	1.32E-21
M2a like TAM	MPHOSPH8	0.294247	0.606	0.338	8.05E-19	1.17E-14
M2a like TAM	DUS1L	0.294172	0.427	0.18	6.48E-26	9.43E-22
M2a like TAM	AZIN1	0.294036	0.653	0.397	9.47E-17	1.38E-12
M2a like TAM	SCAMP4	0.293367	0.38	0.176	1.65E-19	2.40E-15
M2a like TAM	FXR1	0.293345	0.628	0.333	2.97E-23	4.32E-19
M2a like TAM	FMNL1	0.293296	0.668	0.398	6.88E-17	1.00E-12
M2a like TAM	ARL5B	0.292984	0.35	0.152	1.01E-18	1.48E-14
M2a like TAM	SETD7	0.292852	0.398	0.186	1.18E-19	1.72E-15
M2a like TAM	ZNF330	0.292516	0.58	0.328	9.58E-19	1.39E-14

M2a like TAM	RNF141	0.292417	0.456	0.188	3.71E-28	5.40E-24
M2a like TAM	FAM217B	0.292386	0.292	0.101	6.95E-25	1.01E-20
M2a like TAM	MOB3A	0.292372	0.482	0.226	4.45E-23	6.48E-19
M2a like TAM	TMC6	0.292096	0.588	0.342	1.05E-16	1.53E-12
M2a like TAM	CASC3	0.291859	0.361	0.152	4.45E-22	6.48E-18
M2a like TAM	PPP6R3	0.291723	0.405	0.178	3.02E-22	4.40E-18
M2a like TAM	NEMF	0.291126	0.606	0.357	2.45E-17	3.56E-13
M2a like TAM	YTHDC2	0.291112	0.365	0.153	3.39E-22	4.93E-18
M2a like TAM	INTS1	0.290739	0.383	0.151	4.11E-26	5.99E-22
M2a like TAM	RAB35	0.290667	0.474	0.23	1.36E-21	1.98E-17
M2a like TAM	UBLCP1	0.2906	0.489	0.219	2.65E-25	3.86E-21
M2a like TAM	NFE2L1	0.290506	0.507	0.244	4.81E-23	7.00E-19
M2a like TAM	ZBTB44	0.290387	0.354	0.146	3.66E-22	5.32E-18
M2a like TAM	ARID5A	0.290332	0.584	0.332	1.44E-16	2.09E-12
M2a like TAM	SCRN1	0.289925	0.339	0.132	1.80E-23	2.62E-19
M2a like TAM	HNRNPK	0.289242	0.985	0.78	1.61E-17	2.35E-13
M2a like TAM	RASL10A	0.289236	0.186	0.024	1.22E-61	1.77E-57
M2a like TAM	SNX30	0.288872	0.343	0.13	3.15E-25	4.58E-21
M2a like TAM	AKIRIN1	0.288585	0.526	0.254	3.18E-23	4.63E-19
M2a like TAM	C3orf58	0.288519	0.321	0.115	1.86E-25	2.70E-21
M2a like TAM	SEC63	0.287952	0.577	0.345	4.34E-16	6.32E-12
M2a like TAM	CACNB4	0.28762	0.186	0.011	2.04E-131	2.97E-127
M2a like TAM	SH3RF1	0.287385	0.292	0.096	4.09E-27	5.96E-23
M2a like TAM	JAG1	0.287114	0.193	0.061	2.67E-19	3.89E-15
M2a like TAM	WIPI2	0.286929	0.602	0.325	1.95E-20	2.84E-16
M2a like TAM	SLC38A7	0.286352	0.387	0.16	2.14E-23	3.11E-19
M2a like TAM	CNOT7	0.286206	0.584	0.323	1.72E-19	2.50E-15
M2a like TAM	MAT2B	0.285519	0.646	0.341	2.75E-22	4.01E-18
M2a like TAM	MARS	0.285483	0.365	0.14	1.02E-25	1.49E-21
M2a like TAM	ZNF33A	0.285096	0.38	0.162	3.19E-22	4.65E-18
M2a like TAM	MTHFD1L	0.284855	0.328	0.121	2.02E-25	2.94E-21
M2a like TAM	BTBD1	0.284823	0.412	0.177	3.71E-24	5.40E-20
M2a like TAM	ZNF787	0.284715	0.336	0.118	2.88E-28	4.19E-24
M2a like TAM	CALCOCO1	0.284615	0.27	0.104	1.62E-19	2.36E-15
M2a like TAM	DYNC1I2	0.284529	0.807	0.504	6.52E-20	9.49E-16
M2a like TAM	VPS51	0.284349	0.434	0.201	1.28E-21	1.86E-17
M2a like TAM	CD276	0.284171	0.474	0.272	2.41E-13	3.51E-09
M2a like TAM	TRAPPC12	0.284068	0.38	0.152	1.39E-25	2.02E-21
M2a like TAM	DUSP16	0.283685	0.292	0.078	1.77E-37	2.57E-33
M2a like TAM	SLC19A2	0.283539	0.27	0.061	5.57E-44	8.10E-40
M2a like TAM	NSUN4	0.283474	0.288	0.087	4.20E-31	6.12E-27

M2a like TAM	GNPDA1	0.283379	0.664	0.408	5.15E-17	7.49E-13
M2a like TAM	SIGLEC14	0.282851	0.321	0.182	1.05E-09	1.53E-05
M2a like TAM	REV1	0.282563	0.354	0.133	2.93E-26	4.26E-22
M2a like TAM	BIN2	0.282003	0.5	0.261	3.47E-18	5.05E-14
M2a like TAM	MRE11A	0.281994	0.299	0.111	6.64E-23	9.67E-19
M2a like TAM	HEXIM1	0.28198	0.383	0.194	6.78E-15	9.87E-11
M2a like TAM	ATP10D	0.281878	0.336	0.14	7.31E-21	1.06E-16
M2a like TAM	ZNF652	0.2817	0.398	0.146	3.50E-30	5.09E-26
M2a like TAM	WDR70	0.2812	0.365	0.132	1.07E-28	1.55E-24
M2a like TAM	RTN1	0.281142	0.376	0.151	4.15E-24	6.03E-20
M2a like TAM	KIAA0319L	0.280997	0.332	0.141	5.18E-20	7.54E-16
M2a like TAM	DGKD	0.280966	0.248	0.058	6.38E-39	9.28E-35
M2a like TAM	ATRN	0.280872	0.299	0.103	4.67E-26	6.79E-22
M2a like TAM	GZF1	0.280607	0.325	0.125	6.16E-23	8.97E-19
M2a like TAM	RYK	0.280506	0.314	0.108	8.34E-27	1.21E-22
M2a like TAM	ANKFY1	0.280284	0.401	0.175	1.02E-22	1.49E-18
M2a like TAM	PDS5B	0.280146	0.387	0.169	3.69E-21	5.36E-17
M2a like TAM	MIA2	0.28011	0.391	0.156	4.68E-25	6.81E-21
M2a like TAM	PARP8	0.27997	0.383	0.132	7.32E-32	1.07E-27
M2a like TAM	DENND4A	0.279117	0.321	0.11	6.92E-28	1.01E-23
M2a like TAM	ARHGAP35	0.278874	0.328	0.105	3.41E-32	4.96E-28
M2a like TAM	DDI2	0.278859	0.376	0.175	1.87E-18	2.72E-14
M2a like TAM	TRIOBP	0.27852	0.394	0.189	2.06E-18	3.00E-14
M2a like TAM	KIAA0430	0.278472	0.394	0.162	2.72E-24	3.96E-20
M2a like TAM	ANKRD13D	0.278431	0.394	0.178	1.78E-20	2.59E-16
M2a like TAM	SLC26A3	0.278342	0.168	0.013	1.17E-88	1.70E-84
M2a like TAM	ZNF24	0.278119	0.471	0.234	3.41E-20	4.97E-16
M2a like TAM	RCBTB2	0.27791	0.467	0.229	4.02E-19	5.84E-15
M2a like TAM	ABL1	0.277712	0.303	0.095	4.40E-31	6.40E-27
M2a like TAM	ARHGAP30	0.277337	0.591	0.357	2.05E-15	2.99E-11
M2a like TAM	TMED4	0.277189	0.624	0.341	4.12E-21	5.99E-17
M2a like TAM	RAF1	0.27647	0.449	0.212	2.14E-21	3.12E-17
M2a like TAM	TAF15	0.276366	0.511	0.259	1.32E-20	1.92E-16
M2a like TAM	KIAA0232	0.276313	0.394	0.14	4.25E-31	6.18E-27
M2a like TAM	PXN	0.27596	0.35	0.142	3.03E-22	4.40E-18
M2a like TAM	CTDSP1	0.275753	0.456	0.21	1.53E-22	2.22E-18
M2a like TAM	NUP98	0.275694	0.387	0.159	1.86E-24	2.71E-20
M2a like TAM	USP38	0.27564	0.325	0.124	1.27E-23	1.84E-19
M2a like TAM	TMEM255A	0.275624	0.245	0.049	6.58E-46	9.57E-42
M2a like TAM	PFKFB2	0.275572	0.31	0.088	6.00E-36	8.74E-32
M2a like TAM	LOXL3	0.275449	0.288	0.082	3.03E-34	4.41E-30

M2a like TAM	C5orf24	0.275282	0.416	0.198	4.73E-19	6.89E-15
M2a like TAM	MBD4	0.275118	0.599	0.372	5.97E-14	8.69E-10
M2a like TAM	CD59	0.275073	0.854	0.566	6.31E-17	9.18E-13
M2a like TAM	TLR6	0.27504	0.296	0.101	6.33E-26	9.21E-22
M2a like TAM	ZNF644	0.275019	0.431	0.203	5.82E-21	8.47E-17
M2a like TAM	ZNF592	0.275004	0.325	0.095	3.57E-36	5.19E-32
M2a like TAM	TMEM131	0.274981	0.398	0.17	5.83E-23	8.48E-19
M2a like TAM	MRFAP1L1	0.274889	0.369	0.158	2.42E-21	3.52E-17
M2a like TAM	TOMM20	0.274526	0.872	0.587	8.36E-16	1.22E-11
M2a like TAM	CDK12	0.274452	0.431	0.215	6.98E-18	1.02E-13
M2a like TAM	PPTC7	0.274178	0.394	0.18	5.35E-20	7.79E-16
M2a like TAM	ANKS1A	0.274121	0.299	0.107	3.94E-24	5.73E-20
M2a like TAM	USP51	0.273786	0.215	0.041	3.92E-44	5.71E-40
M2a like TAM	NNT	0.27365	0.361	0.174	1.96E-16	2.86E-12
M2a like TAM	CEBPZ	0.273548	0.445	0.22	4.99E-19	7.26E-15
M2a like TAM	PCMTD2	0.273514	0.303	0.086	4.71E-35	6.86E-31
M2a like TAM	TRPM4	0.273418	0.299	0.091	1.81E-31	2.63E-27
M2a like TAM	TOPORS	0.272733	0.387	0.159	2.80E-24	4.08E-20
M2a like TAM	CHID1	0.27246	0.558	0.303	2.14E-19	3.12E-15
M2a like TAM	PDE3B	0.272374	0.255	0.071	4.17E-31	6.06E-27
M2a like TAM	ARHGEF7	0.27233	0.321	0.111	9.39E-28	1.37E-23
M2a like TAM	INPP4A	0.272216	0.409	0.165	5.54E-26	8.06E-22
M2a like TAM	ELL	0.272204	0.343	0.139	2.50E-22	3.64E-18
M2a like TAM	BDP1	0.2721	0.416	0.192	4.07E-20	5.92E-16
M2a like TAM	DIAPH2	0.271879	0.369	0.155	4.59E-22	6.68E-18
M2a like TAM	SF3B3	0.271744	0.383	0.184	5.83E-18	8.48E-14
M2a like TAM	AGO4	0.271442	0.372	0.135	1.63E-28	2.37E-24
M2a like TAM	BAZ2A	0.27142	0.438	0.201	2.61E-21	3.80E-17
M2a like TAM	INO80D	0.27132	0.354	0.137	1.97E-24	2.86E-20
M2a like TAM	RBM38	0.271242	0.288	0.092	1.40E-28	2.04E-24
M2a like TAM	SLC2A8	0.271129	0.394	0.17	2.38E-22	3.46E-18
M2a like TAM	ATP11A	0.27108	0.42	0.204	1.40E-18	2.04E-14
M2a like TAM	TRIM33	0.270983	0.453	0.229	1.50E-18	2.18E-14
M2a like TAM	SFSWAP	0.270736	0.369	0.143	1.14E-25	1.67E-21
M2a like TAM	FAM65A	0.270708	0.391	0.161	2.59E-24	3.76E-20
M2a like TAM	CCNT1	0.270521	0.339	0.113	1.25E-30	1.82E-26
M2a like TAM	TLR10	0.270367	0.168	0.016	9.51E-74	1.38E-69
M2a like TAM	NAGLU	0.270314	0.416	0.188	2.18E-21	3.17E-17
M2a like TAM	ANKIB1	0.269649	0.394	0.151	2.43E-27	3.54E-23
M2a like TAM	ATXN2L	0.269533	0.372	0.153	1.78E-23	2.59E-19
M2a like TAM	ZNF445	0.269494	0.223	0.05	1.03E-36	1.50E-32

M2a like TAM	MAN2B2	0.269367	0.365	0.169	6.37E-18	9.27E-14
M2a like TAM	PDZD8	0.269335	0.318	0.102	1.72E-30	2.51E-26
M2a like TAM	CMTR2	0.26931	0.332	0.13	4.73E-23	6.88E-19
M2a like TAM	TTC17	0.269086	0.449	0.195	2.15E-24	3.13E-20
M2a like TAM	PI4KB	0.2689	0.398	0.161	1.03E-25	1.49E-21
M2a like TAM	HMG20A	0.268715	0.325	0.121	1.71E-24	2.48E-20
M2a like TAM	KBTBD2	0.268547	0.347	0.125	2.52E-27	3.67E-23
M2a like TAM	CERK	0.268537	0.307	0.104	2.40E-27	3.49E-23
M2a like TAM	STX16	0.268377	0.416	0.216	6.02E-16	8.75E-12
M2a like TAM	LARS	0.268258	0.529	0.274	7.28E-20	1.06E-15
M2a like TAM	LASP1	0.268254	0.73	0.466	6.70E-16	9.75E-12
M2a like TAM	FAM13B	0.268046	0.321	0.118	5.58E-25	8.12E-21
M2a like TAM	MAST3	0.267883	0.277	0.083	4.74E-30	6.90E-26
M2a like TAM	NLRP1	0.267503	0.23	0.063	1.77E-28	2.58E-24
M2a like TAM	TTBK2	0.267498	0.27	0.085	1.12E-26	1.62E-22
M2a like TAM	ADNP	0.267365	0.394	0.184	3.65E-19	5.31E-15
M2a like TAM	METAP2	0.267355	0.708	0.452	2.68E-16	3.90E-12
M2a like TAM	ACTN4	0.267272	0.606	0.349	1.63E-16	2.37E-12
M2a like TAM	SP3	0.267081	0.482	0.223	1.42E-23	2.07E-19
M2a like TAM	DMTF1	0.266544	0.277	0.114	2.62E-17	3.81E-13
M2a like TAM	ME2	0.266536	0.715	0.444	4.31E-17	6.26E-13
M2a like TAM	PDPK1	0.266384	0.449	0.219	2.95E-19	4.29E-15
M2a like TAM	MED1	0.266232	0.347	0.144	3.91E-21	5.70E-17
M2a like TAM	RNMT	0.265924	0.599	0.337	2.12E-18	3.08E-14
M2a like TAM	NPIPB5	0.265698	0.266	0.08	3.48E-29	5.07E-25
M2a like TAM	ZNF280D	0.265697	0.328	0.103	1.33E-32	1.93E-28
M2a like TAM	JAZF1	0.265471	0.303	0.1	1.04E-27	1.52E-23
M2a like TAM	CTR9	0.265154	0.423	0.194	1.23E-20	1.79E-16
M2a like TAM	PPM1M	0.265138	0.442	0.214	2.59E-19	3.77E-15
M2a like TAM	SGTA	0.26494	0.412	0.182	6.03E-22	8.77E-18
M2a like TAM	ACIN1	0.264799	0.445	0.224	2.01E-18	2.92E-14
M2a like TAM	GDE1	0.264791	0.522	0.28	7.50E-18	1.09E-13
M2a like TAM	KIAA1147	0.264582	0.314	0.115	4.66E-24	6.78E-20
M2a like TAM	PMEPA1	0.264462	0.245	0.075	1.92E-25	2.79E-21
M2a like TAM	PPP6R1	0.264111	0.453	0.241	5.02E-16	7.31E-12
M2a like TAM	LAMB2	0.264062	0.266	0.076	8.14E-31	1.18E-26
M2a like TAM	ATAD2B	0.264055	0.274	0.103	2.70E-20	3.92E-16
M2a like TAM	TMEM181	0.263684	0.336	0.15	9.71E-18	1.41E-13
M2a like TAM	RABGAP1	0.263657	0.365	0.143	3.44E-25	5.01E-21
M2a like TAM	ALG13	0.263651	0.398	0.211	1.31E-14	1.90E-10
M2a like TAM	ELMSAN1	0.263249	0.347	0.162	1.38E-16	2.01E-12

M2a like TAM	RBM33	0.263031	0.391	0.172	7.37E-21	1.07E-16
M2a like TAM	HMBOX1	0.262803	0.314	0.111	1.46E-25	2.13E-21
M2a like TAM	RALGAPA1	0.26265	0.296	0.118	1.89E-19	2.75E-15
M2a like TAM	ITPK1	0.2626	0.438	0.205	3.49E-21	5.08E-17
M2a like TAM	FAM110B	0.262502	0.168	0.016	1.59E-73	2.32E-69
M2a like TAM	MINK1	0.262437	0.383	0.164	4.77E-22	6.93E-18
M2a like TAM	SRP72	0.262133	0.715	0.447	1.98E-16	2.87E-12
M2a like TAM	CLPX	0.262072	0.387	0.165	1.51E-22	2.20E-18
M2a like TAM	SUPT5H	0.262012	0.485	0.267	1.05E-15	1.53E-11
M2a like TAM	GAB2	0.261825	0.387	0.154	1.48E-25	2.15E-21
M2a like TAM	CASD1	0.261817	0.263	0.082	8.07E-27	1.17E-22
M2a like TAM	MFHAS1	0.261573	0.321	0.138	5.94E-18	8.64E-14
M2a like TAM	SEC16A	0.261094	0.394	0.164	4.64E-24	6.75E-20
M2a like TAM	LIMD1	0.260774	0.358	0.148	6.57E-22	9.56E-18
M2a like TAM	H6PD	0.260694	0.336	0.121	1.51E-26	2.20E-22
M2a like TAM	ZNF710	0.260489	0.325	0.129	1.65E-21	2.40E-17
M2a like TAM	CEACAM5	0.260455	0.237	0.047	6.82E-46	9.92E-42
M2a like TAM	MAN2C1	0.260074	0.383	0.149	1.34E-26	1.95E-22
M2a like TAM	HERC4	0.25999	0.456	0.211	5.15E-22	7.49E-18
M2a like TAM	HERC3	0.259613	0.321	0.121	3.61E-23	5.26E-19
M2a like TAM	TLE4	0.259371	0.423	0.203	5.73E-19	8.34E-15
M2a like TAM	SEC23B	0.259193	0.529	0.292	1.57E-17	2.28E-13
M2a like TAM	DUSP22	0.259116	0.42	0.191	2.86E-21	4.17E-17
M2a like TAM	PIGT	0.25881	0.595	0.346	1.12E-16	1.64E-12
M2a like TAM	SCAMP1	0.258796	0.332	0.141	1.91E-19	2.78E-15
M2a like TAM	DDOST	0.258776	0.704	0.417	1.62E-16	2.36E-12
M2a like TAM	RALGAPB	0.258315	0.318	0.123	6.77E-22	9.85E-18
M2a like TAM	RNF166	0.258284	0.431	0.203	1.33E-20	1.94E-16
M2a like TAM	ZZZ3	0.258262	0.274	0.092	1.65E-24	2.40E-20
M2a like TAM	IL17RA	0.258011	0.474	0.271	1.97E-13	2.87E-09
M2a like TAM	KDM6A	0.257689	0.285	0.08	4.17E-34	6.07E-30
M2a like TAM	FBXW2	0.25758	0.372	0.179	1.23E-17	1.79E-13
M2a like TAM	LGALS8	0.257537	0.544	0.311	2.33E-16	3.39E-12
M2a like TAM	CCNT2	0.257189	0.318	0.129	1.84E-20	2.68E-16
M2a like TAM	QSER1	0.25712	0.332	0.13	1.14E-22	1.66E-18
M2a like TAM	MVB12B	0.257098	0.27	0.07	4.48E-36	6.52E-32
M2a like TAM	RFX1	0.256692	0.259	0.084	6.14E-25	8.93E-21
M2a like TAM	ATG16L2	0.256449	0.332	0.132	1.43E-21	2.09E-17
M2a like TAM	MED29	0.256444	0.489	0.239	1.52E-20	2.21E-16
M2a like TAM	SHPRH	0.256349	0.299	0.123	6.91E-19	1.01E-14
M2a like TAM	HDAC5	0.256342	0.328	0.113	2.18E-28	3.18E-24

M2a like TAM	EHMT1	0.256304	0.347	0.137	1.63E-23	2.37E-19
M2a like TAM	PKD2	0.255856	0.263	0.104	9.30E-18	1.35E-13
M2a like TAM	ADIPOR2	0.255819	0.387	0.167	3.55E-22	5.17E-18
M2a like TAM	DDX39B	0.255691	0.277	0.108	9.77E-20	1.42E-15
M2a like TAM	KDM3B	0.255573	0.38	0.154	3.93E-24	5.73E-20
M2a like TAM	TRPC4AP	0.255387	0.383	0.174	1.01E-19	1.47E-15
M2a like TAM	MIB1	0.255046	0.285	0.107	5.32E-21	7.73E-17
M2a like TAM	P2RY12	0.255015	0.139	0.003	5.90E-192	8.58E-188
M2a like TAM	TMEM8A	0.25447	0.405	0.18	2.52E-21	3.67E-17
M2a like TAM	RAB11FIP2	0.254405	0.296	0.124	1.07E-17	1.55E-13
M2a like TAM	EDEM2	0.254377	0.526	0.309	3.70E-14	5.39E-10
M2a like TAM	ZNF431	0.25416	0.281	0.106	1.40E-20	2.04E-16
M2a like TAM	GOPC	0.253865	0.38	0.18	2.02E-17	2.94E-13
M2a like TAM	RABGGTB	0.253776	0.536	0.296	6.56E-17	9.54E-13
M2a like TAM	MYSM1	0.253666	0.296	0.11	2.35E-22	3.42E-18
M2a like TAM	PDE4A	0.253628	0.354	0.177	2.72E-14	3.96E-10
M2a like TAM	BEST1	0.253458	0.292	0.11	2.00E-21	2.91E-17
M2a like TAM	MGA	0.253441	0.35	0.13	1.09E-25	1.59E-21
M2a like TAM	DPP7	0.253307	0.912	0.674	5.07E-13	7.38E-09
M2a like TAM	CABIN1	0.253089	0.296	0.098	2.65E-27	3.86E-23
M2a like TAM	HLTF	0.252987	0.274	0.089	1.00E-25	1.46E-21
M2a like TAM	SNX14	0.252895	0.489	0.276	3.22E-15	4.69E-11
M2a like TAM	CRKL	0.252862	0.328	0.134	2.12E-20	3.08E-16
M2a like TAM	HIVEP3	0.2527	0.255	0.101	7.92E-17	1.15E-12
M2a like TAM	STT3A	0.252449	0.427	0.216	1.76E-17	2.56E-13
M2a like TAM	AKT2	0.252384	0.358	0.145	9.42E-23	1.37E-18
M2a like TAM	DOPEY2	0.252319	0.35	0.15	2.54E-20	3.69E-16
M2a like TAM	PLXNA2	0.252273	0.179	0.029	9.57E-45	1.39E-40
M2a like TAM	NEDD4L	0.251664	0.277	0.08	1.90E-31	2.77E-27
M2a like TAM	LRCH1	0.25163	0.292	0.103	7.55E-24	1.10E-19
M2a like TAM	DTNA	0.251515	0.19	0.039	3.12E-35	4.54E-31
M2a like TAM	CRCP	0.251399	0.274	0.088	6.83E-27	9.93E-23
M2a like TAM	DENND4B	0.251259	0.314	0.15	9.91E-15	1.44E-10
M2a like TAM	TBC1D16	0.251184	0.255	0.064	8.79E-36	1.28E-31
M2a like TAM	FAF2	0.250689	0.361	0.16	1.89E-19	2.74E-15
M2a like TAM	EDA	0.250613	0.179	0.012	6.12E-113	8.91E-109
M2a like TAM	MTAP	0.250313	0.361	0.144	4.39E-24	6.39E-20
M2a like TAM	BTAF1	0.250285	0.299	0.099	1.32E-27	1.93E-23
M2a like TAM	SPTY2D1	0.250084	0.376	0.183	1.13E-16	1.65E-12
M2b like TAM	CXCL3	2.095882	0.722	0.38	6.22E-93	9.06E-89

M2b like TAM	CCL3	1.875592	0.854	0.613	1.12E-94	1.63E-90
M2b like TAM	IER2	1.863793	0.862	0.542	1.05E-131	1.53E-127
M2b like TAM	CXCL2	1.817181	0.769	0.386	8.70E-109	1.27E-104
M2b like TAM	IER3	1.759327	0.88	0.59	9.52E-123	1.39E-118
M2b like TAM	EGR2	1.694365	0.561	0.19	1.68E-118	2.45E-114
M2b like TAM	EGR1	1.672503	0.885	0.467	4.53E-144	6.59E-140
M2b like TAM	CFD	1.478279	0.837	0.52	1.40E-101	2.04E-97
M2b like TAM	VMO1	1.475395	0.68	0.424	6.37E-63	9.27E-59
M2b like TAM	NR4A2	1.418036	0.882	0.539	1.50E-119	2.19E-115
M2b like TAM	GADD45B	1.401309	0.8	0.506	2.03E-92	2.96E-88
M2b like TAM	PLTP	1.365971	0.775	0.523	5.90E-71	8.58E-67
M2b like TAM	CD83	1.36584	0.788	0.505	8.28E-100	1.20E-95
M2b like TAM	JUNB	1.347097	0.93	0.728	6.16E-103	8.97E-99
M2b like TAM	ATF3	1.338961	0.767	0.518	4.40E-77	6.40E-73
M2b like TAM	NFKBIA	1.337283	0.891	0.734	5.59E-78	8.13E-74
M2b like TAM	CCL4	1.264908	0.676	0.504	1.37E-35	1.99E-31
M2b like TAM	JUN	1.23198	0.868	0.697	5.55E-64	8.08E-60
M2b like TAM	DUSP2	1.20505	0.425	0.239	1.92E-33	2.79E-29
M2b like TAM	BTG2	1.193223	0.713	0.475	1.17E-61	1.70E-57
M2b like TAM	KLF4	1.179028	0.726	0.453	4.79E-67	6.97E-63
M2b like TAM	PNRC1	1.141039	0.915	0.735	4.45E-97	6.48E-93
M2b like TAM	FOS	1.132745	0.994	0.845	1.78E-126	2.59E-122
M2b like TAM	PPP1R15A	1.098176	0.707	0.483	1.91E-61	2.78E-57
M2b like TAM	CCL13	1.06511	0.278	0.168	6.72E-12	9.77E-08
M2b like TAM	NFKBIZ	1.038751	0.621	0.411	1.54E-42	2.24E-38
M2b like TAM	OTUD1	0.986425	0.456	0.292	6.73E-28	9.79E-24
M2b like TAM	LMNA	0.97078	0.695	0.57	1.01E-29	1.46E-25
M2b like TAM	SOCS3	0.956866	0.773	0.546	2.80E-56	4.07E-52
M2b like TAM	IL1B	0.954799	0.357	0.288	1.07E-06	0.015552
M2b like TAM	BTG1	0.944023	0.868	0.746	1.90E-49	2.76E-45
M2b like TAM	HLA-DRB1	0.905249	0.996	0.958	2.48E-64	3.62E-60
M2b like TAM	DUSP1	0.887623	0.988	0.868	8.95E-92	1.30E-87

M2b like TAM	CST3	0.874172	0.998	0.958	2.77E-101	4.03E-97
M2b like TAM	CHMP1B	0.866585	0.649	0.554	3.50E-22	5.09E-18
M2b like TAM	MSR1	0.865858	0.825	0.626	5.25E-47	7.64E-43
M2b like TAM	TNF	0.830758	0.287	0.127	5.71E-29	8.31E-25
M2b like TAM	HBEGF	0.820445	0.538	0.401	4.57E-20	6.65E-16
M2b like TAM	IFITM2	0.809911	0.616	0.445	1.13E-33	1.65E-29
M2b like TAM	CD14	0.791072	0.973	0.857	1.56E-48	2.27E-44
M2b like TAM	RGS2	0.781738	0.693	0.589	1.26E-18	1.83E-14
M2b like TAM	EREG	0.737552	0.157	0.095	7.36E-07	0.010704
M2b like TAM	CCL3L3	0.733198	0.186	0.04	8.47E-54	1.23E-49
M2b like TAM	OSM	0.731577	0.299	0.185	1.67E-13	2.43E-09
M2b like TAM	S100A4	0.692915	0.911	0.832	6.92E-31	1.01E-26
M2b like TAM	TSC22D1	0.689595	0.431	0.379	3.27E-07	0.004758
M2b like TAM	FCGR1A	0.683024	0.67	0.551	1.10E-26	1.60E-22
M2b like TAM	TCOF1	0.663665	0.285	0.181	5.05E-14	7.35E-10
M2b like TAM	EGR3	0.653574	0.254	0.096	2.95E-33	4.29E-29
M2b like TAM	HLA-DQA1	0.652658	0.979	0.901	4.69E-45	6.82E-41
M2b like TAM	PMAIP1	0.62145	0.264	0.192	4.32E-08	0.000629
M2b like TAM	IL10	0.611585	0.2	0.114	4.28E-11	6.23E-07
M2b like TAM	H3F3B	0.607616	0.975	0.936	1.27E-47	1.84E-43
M2b like TAM	CITED2	0.605067	0.458	0.368	8.97E-13	1.31E-08
M2b like TAM	RASGEF1B	0.59785	0.505	0.39	3.46E-16	5.03E-12
M2b like TAM	GOLIM4	0.584306	0.447	0.359	6.61E-14	9.62E-10
M2b like TAM	GPR183	0.58148	0.699	0.649	1.84E-11	2.68E-07
M2b like TAM	IER5	0.572154	0.553	0.502	8.32E-11	1.21E-06
M2b like TAM	ABL2	0.546018	0.394	0.34	5.90E-08	0.000859
M2b like TAM	PILRA	0.528298	0.571	0.501	4.57E-14	6.64E-10
M2b like TAM	EVI2A	0.520809	0.565	0.502	5.46E-14	7.95E-10
M2b like TAM	HSPA1A	0.501688	0.771	0.697	4.09E-14	5.95E-10
M2b like TAM	HSPA1B	0.497139	0.559	0.502	2.36E-08	0.000343
M2b like TAM	CD37	0.495822	0.662	0.575	1.23E-19	1.78E-15
M2b like TAM	HLA-DRA	0.484555	1	0.99	7.60E-36	1.11E-31
M2b like TAM	CSRNP1	0.483537	0.262	0.177	2.68E-10	3.90E-06
M2b like TAM	SRGN	0.481424	1	0.96	1.36E-41	1.97E-37
M2b like TAM	FOSL2	0.459189	0.449	0.384	1.40E-08	0.000204
M2b like TAM	FCGR1B	0.458542	0.289	0.219	8.79E-08	0.001279
M2b like TAM	YPEL3	0.457647	0.311	0.233	6.22E-09	9.05E-05
M2b like TAM	SERTAD1	0.434844	0.315	0.246	9.86E-08	0.001434
M2b like TAM	2-Mar	0.432025	0.404	0.349	1.48E-08	0.000215
M2b like TAM	TYROBP	0.420059	1	0.982	2.45E-50	3.57E-46
M2b like TAM	HSD17B11	0.394608	0.515	0.493	2.61E-08	0.00038

M2b like TAM	GPX3	0.393368	0.452	0.373	2.17E-08	0.000316
M2b like TAM	EIF4A2	0.385096	0.687	0.624	2.16E-13	3.14E-09
M2b like TAM	HCST	0.378666	0.781	0.714	2.21E-12	3.21E-08
M2b like TAM	GLTSCR2	0.376785	0.563	0.521	3.76E-09	5.47E-05
M2b like TAM	LAPTM4A	0.374609	0.814	0.786	1.83E-15	2.67E-11
M2b like TAM	CLEC2B	0.366221	0.67	0.622	7.78E-13	1.13E-08
M2b like TAM	AP1S2	0.358143	0.565	0.52	8.89E-09	0.000129
M2b like TAM	DDAH2	0.356201	0.447	0.419	8.71E-07	0.012667
M2b like TAM	DPEP2	0.348703	0.19	0.12	6.81E-08	0.000991
M2b like TAM	UBE2B	0.311674	0.579	0.569	2.70E-07	0.003932
M2b like TAM	APOE	0.30769	0.843	0.797	2.92E-06	0.042481
M2b like TAM	TOMM7	0.304997	0.907	0.851	4.41E-16	6.42E-12
M2b like TAM	FXYD5	0.255211	0.703	0.724	4.82E-07	0.007011
M2c like TAM	F13A1	1.927396	0.5	0.16	2.78E-243	4.05E-239
M2c like TAM	THBS1	1.543158	0.331	0.113	2.22E-134	3.23E-130
M2c like TAM	LYVE1	1.479388	0.182	0.025	2.46E-187	3.58E-183
M2c like TAM	CD163	1.080001	0.964	0.659	1.42E-259	2.07E-255
M2c like TAM	JUND	1.079124	0.983	0.67	0	0
M2c like TAM	MPEG1	1.020294	0.952	0.591	7.23E-280	1.05E-275
M2c like TAM	ZFP36L1	1.014111	0.971	0.711	6.57E-238	9.56E-234
M2c like TAM	TXNIP	0.975711	0.967	0.735	1.02E-195	1.48E-191
M2c like TAM	TTYH3	0.934375	0.916	0.538	2.62E-291	3.82E-287
M2c like TAM	AHR	0.921304	0.825	0.405	6.41E-269	9.32E-265
M2c like TAM	AHNAK	0.919246	0.836	0.462	4.63E-213	6.73E-209
M2c like TAM	CPVL	0.916074	0.878	0.533	4.59E-186	6.68E-182
M2c like TAM	CD209	0.906104	0.636	0.259	1.02E-217	1.48E-213
M2c like TAM	ZEB2	0.883491	0.963	0.609	8.29E-271	1.21E-266
M2c like TAM	TIMP2	0.880443	0.981	0.701	5.77E-303	8.40E-299

M2c like TAM	NRP1	0.878602	0.896	0.504	3.26E-266	4.75E-262
M2c like TAM	HNRNPU	0.877037	0.965	0.674	8.95E-212	1.30E-207
M2c like TAM	NAMPT	0.863806	0.953	0.684	3.18E-216	4.63E-212
M2c like TAM	PTPRC	0.862015	0.983	0.708	6.33E-297	9.22E-293
M2c like TAM	FCGR2A	0.842103	0.984	0.799	2.37E-194	3.45E-190
M2c like TAM	SPRED1	0.824422	0.845	0.433	2.87E-251	4.17E-247
M2c like TAM	WWP1	0.823045	0.56	0.185	2.03E-258	2.96E-254
M2c like TAM	LGMN	0.808716	0.953	0.731	5.40E-119	7.86E-115
M2c like TAM	LRP1	0.807276	0.831	0.432	2.68E-240	3.90E-236
M2c like TAM	PLAU	0.806013	0.716	0.448	4.00E-106	5.83E-102
M2c like TAM	HIF1A	0.802981	0.931	0.646	2.18E-178	3.17E-174
M2c like TAM	MAFB	0.80236	0.974	0.717	1.80E-189	2.61E-185
M2c like TAM	IQGAP1	0.782539	0.959	0.638	3.37E-251	4.91E-247
M2c like TAM	VMP1	0.769604	0.956	0.657	1.51E-166	2.19E-162
M2c like TAM	MBNL1	0.760678	0.916	0.523	2.41E-261	3.50E-257
M2c like TAM	IQGAP2	0.759746	0.83	0.435	3.21E-228	4.67E-224
M2c like TAM	DUSP6	0.742549	0.661	0.333	1.08E-151	1.57E-147
M2c like TAM	THBD	0.716967	0.586	0.296	3.36E-119	4.88E-115
M2c like TAM	ACTR2	0.713771	0.987	0.768	2.02E-251	2.94E-247
M2c like TAM	LILRB5	0.711625	0.469	0.162	1.81E-187	2.63E-183
M2c like TAM	MAN1A1	0.708759	0.558	0.23	3.21E-170	4.67E-166

M2c like TAM	DMXL2	0.694867	0.805	0.404	2.81E-221	4.09E-217
M2c like TAM	DSE	0.691982	0.825	0.436	1.64E-213	2.38E-209
M2c like TAM	SDC3	0.691641	0.714	0.323	1.48E-205	2.15E-201
M2c like TAM	FAM198B	0.687144	0.742	0.325	1.59E-234	2.32E-230
M2c like TAM	RGL1	0.68488	0.642	0.272	5.60E-197	8.16E-193
M2c like TAM	ARHGAP18	0.679262	0.946	0.609	1.83E-212	2.66E-208
M2c like TAM	SNX6	0.67425	0.936	0.59	3.51E-204	5.11E-200
M2c like TAM	COLEC12	0.670032	0.411	0.154	8.54E-140	1.24E-135
M2c like TAM	SLC16A10	0.666111	0.599	0.297	1.30E-129	1.89E-125
M2c like TAM	CD84	0.652766	0.904	0.579	1.38E-181	2.01E-177
M2c like TAM	EPB41L3	0.649809	0.802	0.398	1.74E-218	2.53E-214
M2c like TAM	LHFPL2	0.648914	0.802	0.482	1.79E-153	2.61E-149
M2c like TAM	SAMHD1	0.64244	0.961	0.683	2.84E-195	4.13E-191
M2c like TAM	ANKRD12	0.640435	0.838	0.445	1.14E-211	1.66E-207
M2c like TAM	RUNX1	0.636556	0.617	0.267	3.97E-174	5.78E-170
M2c like TAM	NABP1	0.635529	0.775	0.42	9.41E-170	1.37E-165
M2c like TAM	CR1	0.634346	0.366	0.119	2.51E-155	3.66E-151
M2c like TAM	RBM47	0.626474	0.874	0.501	3.71E-194	5.41E-190
M2c like TAM	TGOLN2	0.622181	0.934	0.589	5.02E-195	7.31E-191
M2c like TAM	OLFML2B	0.620991	0.684	0.311	2.69E-188	3.92E-184
M2c like TAM	RBPJ	0.619966	0.925	0.609	1.92E-150	2.80E-146

M2c like TAM	CCDC88A	0.618138	0.879	0.498	3.37E-197	4.90E-193
M2c like TAM	MYO5A	0.617884	0.676	0.275	7.95E-234	1.16E-229
M2c like TAM	REG1A	0.613363	0.188	0.063	2.58E-68	3.75E-64
M2c like TAM	YBX3	0.611815	0.861	0.556	4.28E-135	6.23E-131
M2c like TAM	TFRC	0.606372	0.812	0.552	3.34E-92	4.86E-88
M2c like TAM	CCR1	0.602039	0.837	0.493	1.49E-150	2.17E-146
M2c like TAM	PRNP	0.597869	0.86	0.512	1.52E-163	2.21E-159
M2c like TAM	1-Mar	0.596659	0.873	0.521	4.31E-175	6.28E-171
M2c like TAM	TGFBI	0.591762	0.976	0.763	3.61E-113	5.26E-109
M2c like TAM	ROCK1	0.590135	0.821	0.434	1.65E-194	2.40E-190
M2c like TAM	TPP1	0.579862	0.973	0.721	1.45E-165	2.11E-161
M2c like TAM	GNS	0.579601	0.929	0.609	3.90E-180	5.67E-176
M2c like TAM	PTBP3	0.579379	0.801	0.398	2.07E-204	3.01E-200
M2c like TAM	CYBB	0.571271	0.973	0.774	4.60E-139	6.69E-135
M2c like TAM	CECR1	0.570358	0.844	0.53	9.33E-139	1.36E-134
M2c like TAM	FPR3	0.566259	0.949	0.688	4.43E-152	6.44E-148
M2c like TAM	ITGAM	0.564942	0.684	0.347	2.25E-146	3.27E-142
M2c like TAM	RBMS1	0.564039	0.836	0.471	6.96E-175	1.01E-170
M2c like TAM	ZBTB20	0.562397	0.477	0.169	1.11E-179	1.62E-175
M2c like TAM	PMP22	0.55235	0.905	0.633	7.74E-125	1.13E-120
M2c like TAM	RHOQ	0.550752	0.785	0.385	1.15E-192	1.68E-188
M2c like TAM	DYNC1H1	0.549873	0.745	0.364	1.60E-186	2.32E-182

M2c like TAM	ATP6V0A1	0.549701	0.695	0.312	1.48E-194	2.15E-190
M2c like TAM	GNPTAB	0.548712	0.746	0.377	4.27E-181	6.21E-177
M2c like TAM	CTSZ	0.54725	0.994	0.884	1.07E-127	1.56E-123
M2c like TAM	APLP2	0.540335	0.971	0.737	1.50E-131	2.18E-127
M2c like TAM	TAOK1	0.538702	0.659	0.276	7.27E-205	1.06E-200
M2c like TAM	CMIP	0.53684	0.737	0.361	7.02E-177	1.02E-172
M2c like TAM	GNB1	0.532202	0.903	0.555	2.12E-164	3.08E-160
M2c like TAM	FMN1	0.529907	0.545	0.189	7.00E-220	1.02E-215
M2c like TAM	IL13RA1	0.525721	0.815	0.453	8.22E-170	1.20E-165
M2c like TAM	CTSB	0.52509	1	0.956	4.34E-112	6.32E-108
M2c like TAM	LILRB2	0.522977	0.756	0.421	4.75E-136	6.91E-132
M2c like TAM	MSN	0.522527	0.935	0.663	1.13E-142	1.65E-138
M2c like TAM	FAM91A1	0.522008	0.648	0.272	1.08E-194	1.57E-190
M2c like TAM	ADAM9	0.515947	0.829	0.495	6.70E-153	9.75E-149
M2c like TAM	UGCG	0.512351	0.618	0.293	2.47E-149	3.60E-145
M2c like TAM	CD46	0.510153	0.745	0.376	3.85E-173	5.61E-169
M2c like TAM	CTSS	0.508022	0.999	0.918	3.12E-158	4.53E-154
M2c like TAM	RAB31	0.504618	0.946	0.689	1.34E-133	1.95E-129
M2c like TAM	IVNS1ABP	0.502404	0.773	0.421	3.24E-144	4.72E-140
M2c like TAM	SIGLEC1	0.50196	0.539	0.231	1.54E-144	2.24E-140
M2c like TAM	WIPF1	0.501455	0.888	0.561	2.40E-154	3.49E-150

M2c like TAM	DAPK1	0.500468	0.707	0.352	7.83E-163	1.14E-158
M2c like TAM	SGPL1	0.500047	0.776	0.471	1.29E-120	1.88E-116
M2c like TAM	TMEM2	0.49826	0.491	0.162	7.62E-200	1.11E-195
M2c like TAM	EMILIN2	0.493609	0.765	0.413	5.60E-155	8.15E-151
M2c like TAM	RAP2B	0.490346	0.859	0.516	3.92E-143	5.71E-139
M2c like TAM	CLEC10A	0.482116	0.294	0.135	5.05E-63	7.34E-59
M2c like TAM	CDV3	0.477876	0.866	0.523	3.84E-147	5.59E-143
M2c like TAM	NPTN	0.474248	0.689	0.306	2.07E-177	3.02E-173
M2c like TAM	PLEKHO2	0.473877	0.707	0.344	2.03E-165	2.95E-161
M2c like TAM	PAPSS2	0.467427	0.549	0.226	1.47E-158	2.14E-154
M2c like TAM	TET2	0.465676	0.528	0.193	2.54E-187	3.70E-183
M2c like TAM	SLC11A2	0.46468	0.608	0.296	9.70E-133	1.41E-128
M2c like TAM	IDH1	0.461911	0.839	0.528	8.70E-133	1.27E-128
M2c like TAM	LENG8	0.461238	0.636	0.274	7.46E-173	1.09E-168
M2c like TAM	SATB1	0.46088	0.536	0.224	3.15E-150	4.58E-146
M2c like TAM	B3GNT5	0.459499	0.572	0.261	1.18E-135	1.72E-131
M2c like TAM	ALCAM	0.457958	0.686	0.395	1.20E-104	1.75E-100
M2c like TAM	CMKLR1	0.454829	0.654	0.33	1.22E-132	1.77E-128
M2c like TAM	KIF1B	0.454644	0.488	0.174	5.65E-178	8.22E-174
M2c like TAM	LITAF	0.452599	0.965	0.829	2.27E-92	3.30E-88
M2c like TAM	TNS3	0.449907	0.698	0.372	1.45E-124	2.12E-120
M2c like TAM	PRPF40A	0.448479	0.799	0.454	7.34E-137	1.07E-132

M2c like TAM	PBRM1	0.448284	0.653	0.302	3.85E-162	5.60E-158
M2c like TAM	ITGA4	0.447651	0.481	0.182	1.38E-160	2.00E-156
M2c like TAM	FCGR2B	0.446767	0.577	0.34	2.79E-68	4.06E-64
M2c like TAM	CAB39	0.445855	0.697	0.339	6.34E-156	9.22E-152
M2c like TAM	EHBP1L1	0.445025	0.701	0.337	7.39E-164	1.07E-159
M2c like TAM	ATF6	0.444658	0.656	0.322	3.70E-146	5.38E-142
M2c like TAM	KPNA4	0.443429	0.611	0.274	5.29E-157	7.70E-153
M2c like TAM	ZBTB7A	0.441584	0.581	0.247	3.77E-161	5.48E-157
M2c like TAM	MOB1B	0.440533	0.529	0.199	3.25E-182	4.73E-178
M2c like TAM	AGFG1	0.438755	0.571	0.235	4.95E-163	7.20E-159
M2c like TAM	RASSF4	0.438344	0.809	0.493	1.64E-112	2.38E-108
M2c like TAM	HSPH1	0.437562	0.755	0.459	1.08E-98	1.58E-94
M2c like TAM	LCP1	0.43726	0.942	0.725	1.16E-95	1.69E-91
M2c like TAM	GAS7	0.437082	0.472	0.178	1.19E-156	1.73E-152
M2c like TAM	NRP2	0.436752	0.677	0.357	5.63E-121	8.19E-117
M2c like TAM	LAMP2	0.436052	0.947	0.703	1.32E-121	1.91E-117
M2c like TAM	MAP3K1	0.433878	0.546	0.213	1.21E-169	1.76E-165
M2c like TAM	TNKS2	0.433863	0.611	0.258	7.34E-170	1.07E-165
M2c like TAM	DDX3Y	0.431627	0.287	0.132	1.12E-64	1.64E-60
M2c like TAM	PIK3AP1	0.428465	0.792	0.443	2.36E-133	3.43E-129
M2c like TAM	TM9SF3	0.428363	0.797	0.437	3.05E-149	4.43E-145
M2c like TAM	CYTH1	0.428354	0.692	0.34	4.57E-146	6.66E-142
M2c like TAM	SERINC3	0.427271	0.719	0.372	1.52E-143	2.21E-139

M2c like TAM	CAST	0.425454	0.833	0.513	3.64E-124	5.30E-120
M2c like TAM	SLC38A2	0.425116	0.608	0.313	4.65E-112	6.76E-108
M2c like TAM	EFR3A	0.422624	0.672	0.309	1.76E-160	2.56E-156
M2c like TAM	ACAP2	0.422077	0.726	0.378	5.17E-142	7.52E-138
M2c like TAM	WDR26	0.419946	0.666	0.332	3.96E-135	5.77E-131
M2c like TAM	KIAA0930	0.41969	0.708	0.366	4.36E-138	6.34E-134
M2c like TAM	PRKAR1A	0.419654	0.855	0.53	1.77E-133	2.57E-129
M2c like TAM	PHACTR2	0.418696	0.651	0.32	1.16E-141	1.68E-137
M2c like TAM	USP8	0.415181	0.653	0.311	2.46E-151	3.58E-147
M2c like TAM	RNF13	0.413566	0.914	0.632	4.88E-113	7.11E-109
M2c like TAM	DSC2	0.410813	0.52	0.199	5.21E-166	7.59E-162
M2c like TAM	SGPP1	0.410603	0.552	0.235	1.81E-150	2.63E-146
M2c like TAM	CDK6	0.41022	0.452	0.184	8.23E-126	1.20E-121
M2c like TAM	LRRC8C	0.409702	0.495	0.195	3.32E-153	4.83E-149
M2c like TAM	CTNNA1	0.409361	0.757	0.415	1.98E-137	2.88E-133
M2c like TAM	BLOC1S6	0.408608	0.642	0.304	6.25E-146	9.09E-142
M2c like TAM	IGF2R	0.407924	0.654	0.357	1.03E-104	1.49E-100
M2c like TAM	LRRFIP1	0.40769	0.931	0.665	7.12E-112	1.04E-107
M2c like TAM	SMG1	0.407351	0.55	0.222	3.27E-159	4.75E-155
M2c like TAM	ME1	0.401754	0.407	0.15	1.61E-134	2.34E-130
M2c like TAM	CYTH4	0.399225	0.642	0.324	4.85E-124	7.06E-120

M2c like TAM	COL4A3BP	0.398535	0.567	0.245	1.55E-148	2.26E-144
M2c like TAM	PAK2	0.396981	0.834	0.508	6.69E-125	9.73E-121
M2c like TAM	CSNK1A1	0.396702	0.853	0.531	1.17E-117	1.70E-113
M2c like TAM	EPAS1	0.39591	0.44	0.176	8.35E-128	1.22E-123
M2c like TAM	NCEH1	0.394237	0.641	0.326	4.60E-117	6.70E-113
M2c like TAM	DOCK11	0.391595	0.498	0.191	6.12E-159	8.91E-155
M2c like TAM	ASPH	0.390872	0.61	0.311	1.51E-111	2.19E-107
M2c like TAM	FMNL2	0.389819	0.639	0.333	3.48E-115	5.06E-111
M2c like TAM	DUSP3	0.387807	0.665	0.364	1.68E-111	2.44E-107
M2c like TAM	SIPA1L1	0.385652	0.411	0.152	2.67E-134	3.88E-130
M2c like TAM	CRTAP	0.382116	0.716	0.402	4.67E-109	6.80E-105
M2c like TAM	JAK1	0.381418	0.773	0.44	1.25E-117	1.82E-113
M2c like TAM	TMOD3	0.380798	0.677	0.346	9.54E-130	1.39E-125
M2c like TAM	CYBRD1	0.379234	0.412	0.168	8.38E-114	1.22E-109
M2c like TAM	ZYG11B	0.379058	0.444	0.165	6.08E-148	8.85E-144
M2c like TAM	7-Mar	0.377295	0.551	0.235	5.43E-150	7.90E-146
M2c like TAM	SYNCRIP	0.375542	0.766	0.441	1.13E-111	1.65E-107
M2c like TAM	KIF5B	0.373318	0.87	0.546	5.64E-115	8.20E-111
M2c like TAM	AES	0.372393	0.728	0.402	6.77E-120	9.85E-116
M2c like TAM	BNIP2	0.371628	0.774	0.45	5.75E-119	8.37E-115
M2c like TAM	ARL8A	0.371585	0.639	0.331	1.83E-118	2.67E-114

M2c like TAM	ETV6	0.371133	0.567	0.251	5.72E-139	8.33E-135
M2c like TAM	EVA1B	0.370086	0.523	0.225	1.25E-135	1.82E-131
M2c like TAM	UBE3A	0.36961	0.606	0.288	9.09E-131	1.32E-126
M2c like TAM	ANTXR2	0.367227	0.401	0.143	1.04E-139	1.51E-135
M2c like TAM	CREB5	0.366955	0.369	0.151	6.05E-99	8.80E-95
M2c like TAM	FNDC3A	0.365949	0.567	0.261	8.33E-127	1.21E-122
M2c like TAM	USP15	0.364016	0.615	0.312	5.95E-115	8.66E-111
M2c like TAM	ARHGAP26	0.363944	0.455	0.177	1.14E-137	1.66E-133
M2c like TAM	ERRFI1	0.361091	0.304	0.131	8.20E-71	1.19E-66
M2c like TAM	SH3PXD2B	0.360119	0.519	0.248	2.95E-109	4.29E-105
M2c like TAM	MCTP1	0.36002	0.491	0.2	2.03E-138	2.96E-134
M2c like TAM	SH3BP2	0.359607	0.613	0.301	3.14E-123	4.57E-119
M2c like TAM	TLR8	0.359418	0.404	0.141	2.75E-144	4.00E-140
M2c like TAM	MTMR6	0.35865	0.551	0.242	6.17E-137	8.98E-133
M2c like TAM	RNF130	0.357315	0.972	0.763	3.90E-88	5.68E-84
M2c like TAM	SPPL2A	0.355455	0.842	0.559	2.36E-97	3.44E-93
M2c like TAM	LDLR	0.355068	0.203	0.065	1.58E-81	2.30E-77
M2c like TAM	STOM	0.354654	0.798	0.498	7.14E-98	1.04E-93
M2c like TAM	SPAG9	0.354374	0.577	0.271	1.88E-124	2.73E-120
M2c like TAM	NCSTN	0.353735	0.615	0.33	7.61E-104	1.11E-99
M2c like TAM	G3BP1	0.352967	0.766	0.464	5.75E-98	8.36E-94
M2c like TAM	GFRA2	0.352732	0.175	0.039	8.28E-113	1.21E-108
M2c like TAM	SLC36A1	0.351246	0.476	0.205	6.42E-120	9.35E-116
M2c like TAM	APC	0.350843	0.392	0.132	2.27E-149	3.30E-145
M2c like TAM	ACSL3	0.350411	0.629	0.323	2.53E-109	3.68E-105

M2c like TAM	SNX27	0.349669	0.581	0.29	3.82E-112	5.56E-108
M2c like TAM	LPGAT1	0.348936	0.521	0.22	3.95E-134	5.75E-130
M2c like TAM	OSBPL11	0.348201	0.497	0.203	4.69E-138	6.83E-134
M2c like TAM	CD36	0.347766	0.452	0.317	6.13E-24	8.91E-20
M2c like TAM	BACH1	0.347724	0.567	0.26	6.10E-126	8.87E-122
M2c like TAM	CALU	0.346431	0.749	0.455	3.40E-91	4.95E-87
M2c like TAM	FAM20A	0.345457	0.401	0.174	5.55E-96	8.07E-92
M2c like TAM	FAR1	0.34524	0.579	0.261	2.77E-134	4.03E-130
M2c like TAM	EIF4G2	0.343515	0.956	0.711	4.51E-96	6.56E-92
M2c like TAM	RHOU	0.343464	0.337	0.117	6.76E-119	9.83E-115
M2c like TAM	DDX42	0.343046	0.472	0.187	6.25E-142	9.09E-138
M2c like TAM	CHSY1	0.341811	0.408	0.154	3.74E-127	5.45E-123
M2c like TAM	N4BP1	0.340204	0.498	0.217	2.74E-122	3.99E-118
M2c like TAM	HSD17B4	0.340198	0.689	0.38	8.87E-112	1.29E-107
M2c like TAM	SCPEP1	0.340002	0.879	0.623	9.69E-77	1.41E-72
M2c like TAM	FKBP15	0.339934	0.608	0.306	1.43E-112	2.08E-108
M2c like TAM	LMO2	0.339843	0.454	0.189	7.83E-119	1.14E-114
M2c like TAM	DDHD1	0.339025	0.413	0.157	4.25E-127	6.19E-123
M2c like TAM	FES	0.338316	0.522	0.236	1.94E-120	2.82E-116
M2c like TAM	SYNE1	0.337874	0.342	0.11	2.89E-136	4.21E-132
M2c like TAM	CAPNS1	0.336963	0.809	0.513	8.00E-94	1.16E-89
M2c like TAM	FAM129A	0.336883	0.461	0.22	2.01E-88	2.93E-84
M2c like TAM	GM2A	0.336256	0.828	0.594	6.96E-57	1.01E-52
M2c like TAM	SNX2	0.334758	0.91	0.618	4.30E-101	6.25E-97
M2c like TAM	CLIP1	0.334128	0.539	0.251	4.66E-115	6.79E-111

M2c like TAM	TMEM127	0.333057	0.572	0.28	9.63E-112	1.40E-107
M2c like TAM	AP3B1	0.331942	0.552	0.254	1.31E-124	1.90E-120
M2c like TAM	MBD2	0.331465	0.672	0.347	2.72E-114	3.96E-110
M2c like TAM	CNOT1	0.330943	0.529	0.248	9.14E-113	1.33E-108
M2c like TAM	UBA6	0.330738	0.548	0.269	4.20E-106	6.11E-102
M2c like TAM	SFT2D2	0.32848	0.625	0.332	2.72E-101	3.96E-97
M2c like TAM	CAPRIN1	0.328258	0.658	0.348	6.60E-114	9.60E-110
M2c like TAM	VOPP1	0.325619	0.8	0.531	3.76E-72	5.47E-68
M2c like TAM	ACSL4	0.324142	0.591	0.298	7.29E-106	1.06E-101
M2c like TAM	PDCD6IP	0.323728	0.683	0.371	1.32E-105	1.91E-101
M2c like TAM	ANO6	0.323359	0.533	0.248	1.47E-113	2.14E-109
M2c like TAM	UBE2Q1	0.321447	0.509	0.226	3.23E-116	4.70E-112
M2c like TAM	TMEM87B	0.320496	0.521	0.234	4.82E-120	7.02E-116
M2c like TAM	GIMAP6	0.320242	0.471	0.196	3.04E-124	4.42E-120
M2c like TAM	UBE2R2	0.320081	0.653	0.364	7.99E-92	1.16E-87
M2c like TAM	ATP6V1A	0.319999	0.783	0.488	9.59E-90	1.40E-85
M2c like TAM	PLEKHB2	0.319972	0.862	0.572	5.38E-91	7.82E-87
M2c like TAM	ROCK2	0.318204	0.376	0.124	2.04E-146	2.97E-142
M2c like TAM	TMEM30A	0.315153	0.717	0.417	1.08E-94	1.58E-90
M2c like TAM	PPP1CB	0.314345	0.878	0.58	2.61E-94	3.79E-90
M2c like TAM	AFTPH	0.31338	0.452	0.188	8.55E-117	1.24E-112
M2c like TAM	MTPN	0.312529	0.814	0.517	1.10E-90	1.60E-86
M2c like TAM	SEMA4A	0.312339	0.475	0.248	9.92E-76	1.44E-71
M2c like TAM	EHD4	0.311782	0.57	0.285	6.71E-106	9.77E-102
M2c like TAM	HPS5	0.311559	0.457	0.194	5.39E-116	7.84E-112

M2c like TAM	FAM208A	0.311037	0.456	0.185	1.87E-126	2.71E-122
M2c like TAM	MDM4	0.310716	0.572	0.274	1.08E-112	1.57E-108
M2c like TAM	SCYL2	0.310484	0.463	0.19	6.83E-124	9.95E-120
M2c like TAM	AGPAT3	0.310092	0.451	0.188	5.04E-117	7.34E-113
M2c like TAM	DCAF7	0.309978	0.675	0.375	4.52E-99	6.58E-95
M2c like TAM	UBE2W	0.308579	0.583	0.293	7.04E-109	1.02E-104
M2c like TAM	AKAP10	0.307301	0.351	0.122	5.16E-125	7.51E-121
M2c like TAM	BHLHE40	0.306881	0.493	0.252	1.13E-79	1.65E-75
M2c like TAM	TLR1	0.306265	0.507	0.225	2.18E-119	3.17E-115
M2c like TAM	ZBTB1	0.304507	0.392	0.164	2.14E-99	3.11E-95
M2c like TAM	LARP4	0.303785	0.48	0.206	6.46E-119	9.40E-115
M2c like TAM	ACE	0.302884	0.358	0.144	2.44E-97	3.55E-93
M2c like TAM	RYBP	0.302103	0.386	0.151	1.67E-110	2.43E-106
M2c like TAM	STAT5A	0.301548	0.438	0.177	2.87E-121	4.17E-117
M2c like TAM	NUMB	0.300441	0.58	0.282	1.52E-107	2.21E-103
M2c like TAM	C20orf194	0.299346	0.373	0.139	1.95E-117	2.83E-113
M2c like TAM	MAPK1	0.299214	0.687	0.389	1.42E-92	2.06E-88
M2c like TAM	PPP3CA	0.29912	0.505	0.228	1.41E-109	2.05E-105
M2c like TAM	WDR11	0.298885	0.508	0.232	1.79E-108	2.61E-104
M2c like TAM	LAIR1	0.298591	0.845	0.589	2.03E-66	2.95E-62
M2c like TAM	EML4	0.298197	0.717	0.453	2.76E-71	4.02E-67
M2c like TAM	NFKB1	0.297974	0.555	0.284	2.44E-91	3.55E-87
M2c like TAM	CERS6	0.29797	0.515	0.239	6.55E-107	9.54E-103
M2c like TAM	SCD	0.297935	0.6	0.372	4.21E-56	6.12E-52
M2c like TAM	LRRC25	0.297659	0.694	0.402	9.83E-87	1.43E-82
M2c like TAM	CRYBG3	0.296559	0.34	0.108	2.14E-139	3.12E-135

M2c like TAM	PAPD4	0.295598	0.531	0.256	1.06E-105	1.54E-101
M2c like TAM	VPS8	0.295098	0.451	0.189	7.12E-115	1.04E-110
M2c like TAM	RC3H2	0.294678	0.371	0.131	5.44E-129	7.92E-125
M2c like TAM	CSGALNACT2	0.294396	0.509	0.241	2.84E-100	4.13E-96
M2c like TAM	CCPG1	0.293648	0.609	0.322	3.01E-95	4.38E-91
M2c like TAM	PANK3	0.293195	0.435	0.186	1.13E-106	1.64E-102
M2c like TAM	PPP2CB	0.292454	0.573	0.278	8.34E-112	1.21E-107
M2c like TAM	TSPAN33	0.292348	0.354	0.138	1.82E-101	2.65E-97
M2c like TAM	GALC	0.292313	0.495	0.24	3.41E-93	4.96E-89
M2c like TAM	ADAMTSL4	0.291336	0.361	0.143	9.72E-101	1.41E-96
M2c like TAM	ABI1	0.290838	0.723	0.438	3.64E-87	5.29E-83
M2c like TAM	NOTCH2	0.290471	0.387	0.178	5.43E-81	7.90E-77
M2c like TAM	GCNT1	0.290048	0.308	0.124	9.02E-86	1.31E-81
M2c like TAM	SLC20A1	0.289559	0.568	0.339	2.38E-55	3.46E-51
M2c like TAM	ARHGEF2	0.289419	0.449	0.21	4.46E-92	6.50E-88
M2c like TAM	RAB8B	0.289114	0.575	0.308	8.81E-87	1.28E-82
M2c like TAM	RUFY1	0.28776	0.493	0.236	1.42E-97	2.07E-93
M2c like TAM	PIP4K2A	0.287369	0.542	0.268	1.69E-98	2.46E-94
M2c like TAM	DYNC1LI2	0.28729	0.438	0.2	8.45E-96	1.23E-91
M2c like TAM	CRK	0.286571	0.494	0.221	2.70E-110	3.93E-106
M2c like TAM	RXRA	0.285552	0.506	0.231	2.05E-107	2.99E-103
M2c like TAM	KIAA0368	0.285532	0.412	0.152	3.16E-132	4.60E-128
M2c like TAM	SPTAN1	0.285362	0.476	0.241	1.24E-79	1.81E-75
M2c like TAM	UBE2Z	0.284907	0.581	0.289	3.83E-103	5.58E-99
M2c like TAM	C2CD5	0.284382	0.328	0.103	3.04E-135	4.42E-131
M2c like TAM	ZFC3H1	0.284126	0.334	0.118	1.00E-114	1.46E-110
M2c like TAM	TRAM1	0.283804	0.878	0.602	4.21E-76	6.13E-72

M2c like TAM	MAP3K3	0.283523	0.335	0.119	4.38E-113	6.38E-109
M2c like TAM	PTP4A2	0.283283	0.892	0.617	4.84E-80	7.04E-76
M2c like TAM	GALNT7	0.282262	0.367	0.135	1.59E-117	2.31E-113
M2c like TAM	SNAP23	0.280689	0.655	0.368	1.81E-91	2.64E-87
M2c like TAM	POLK	0.280418	0.413	0.177	2.63E-99	3.83E-95
M2c like TAM	CCDC186	0.280122	0.352	0.141	1.67E-96	2.43E-92
M2c like TAM	CBX4	0.279543	0.346	0.119	1.20E-122	1.75E-118
M2c like TAM	IL4R	0.279022	0.46	0.208	4.05E-99	5.89E-95
M2c like TAM	CHD8	0.277835	0.358	0.132	4.71E-114	6.86E-110
M2c like TAM	STT3B	0.277617	0.66	0.378	1.06E-84	1.54E-80
M2c like TAM	TSPAN3	0.277202	0.582	0.31	1.55E-85	2.25E-81
M2c like TAM	SLC30A7	0.27613	0.509	0.24	2.03E-98	2.96E-94
M2c like TAM	PSMD1	0.275983	0.664	0.374	7.87E-87	1.15E-82
M2c like TAM	PDP1	0.275777	0.352	0.128	8.64E-115	1.26E-110
M2c like TAM	CTBS	0.274529	0.55	0.277	4.32E-92	6.28E-88
M2c like TAM	COPB2	0.272061	0.599	0.323	2.08E-87	3.03E-83
M2c like TAM	KDM7A	0.271694	0.358	0.142	9.60E-98	1.40E-93
M2c like TAM	RECQL	0.270961	0.517	0.246	2.95E-100	4.29E-96
M2c like TAM	ATP6AP1	0.27046	0.91	0.669	3.65E-64	5.32E-60
M2c like TAM	GSAP	0.269763	0.496	0.242	8.21E-91	1.19E-86
M2c like TAM	C9orf72	0.269584	0.46	0.215	6.84E-89	9.95E-85
M2c like TAM	HSP90B1	0.268692	0.976	0.798	5.89E-45	8.57E-41
M2c like TAM	NAAA	0.268557	0.627	0.361	1.21E-76	1.76E-72
M2c like TAM	ACBD5	0.26801	0.42	0.182	2.09E-99	3.04E-95
M2c like TAM	LMO4	0.267841	0.685	0.394	3.16E-81	4.60E-77
M2c like TAM	SLC25A24	0.26774	0.639	0.353	4.03E-88	5.87E-84
M2c like TAM	PYGL	0.267526	0.502	0.24	1.25E-95	1.81E-91
M2c like TAM	NECAP2	0.267522	0.704	0.434	5.29E-81	7.70E-77
M2c like TAM	CERS2	0.267064	0.493	0.23	3.06E-99	4.45E-95
M2c like TAM	IFNAR1	0.264826	0.704	0.42	6.60E-83	9.61E-79
M2c like TAM	CPSF2	0.263402	0.447	0.211	1.20E-87	1.75E-83
M2c like TAM	FERMT3	0.262926	0.797	0.534	2.86E-66	4.16E-62
M2c like TAM	TRAPPC8	0.262285	0.354	0.129	3.09E-114	4.49E-110
M2c like TAM	PPFIA1	0.261426	0.449	0.2	2.14E-99	3.11E-95
M2c like TAM	IQSEC1	0.260851	0.37	0.156	6.39E-90	9.30E-86

M2c like TAM	RASA1	0.260673	0.322	0.117	1.32E-105	1.92E-101
M2c like TAM	SP1	0.260614	0.389	0.16	7.86E-101	1.14E-96
M2c like TAM	RAB3GAP2	0.26059	0.367	0.15	1.23E-95	1.79E-91
M2c like TAM	DIAPH1	0.260409	0.478	0.235	7.28E-84	1.06E-79
M2c like TAM	EXOC1	0.260107	0.447	0.199	4.99E-99	7.26E-95
M2c like TAM	HK1	0.259842	0.539	0.28	3.54E-86	5.15E-82
M2c like TAM	CTSO	0.259346	0.369	0.145	6.41E-104	9.32E-100
M2c like TAM	GAS2L3	0.258859	0.309	0.11	3.63E-101	5.28E-97
M2c like TAM	HRH2	0.258395	0.402	0.187	1.41E-79	2.06E-75
M2c like TAM	ATP6AP2	0.258381	0.963	0.785	8.73E-59	1.27E-54
M2c like TAM	OSTM1	0.257036	0.632	0.367	6.21E-77	9.04E-73
M2c like TAM	JARID2	0.256723	0.425	0.195	5.94E-86	8.64E-82
M2c like TAM	CD180	0.256143	0.375	0.171	3.36E-77	4.89E-73
M2c like TAM	KPNA3	0.255906	0.527	0.253	1.72E-100	2.50E-96
M2c like TAM	MYO1E	0.255276	0.383	0.175	9.11E-78	1.33E-73
M2c like TAM	PRDM2	0.25511	0.396	0.174	2.67E-89	3.89E-85
M2c like TAM	ADAM17	0.254619	0.589	0.317	1.97E-85	2.87E-81
M2c like TAM	PITPNA	0.254163	0.542	0.282	1.10E-88	1.60E-84
M2c like TAM	FOXN2	0.254017	0.413	0.184	3.39E-91	4.93E-87
M2c like TAM	SOS1	0.253625	0.329	0.115	7.99E-113	1.16E-108
M2c like TAM	PHTF2	0.253401	0.367	0.15	1.01E-94	1.47E-90
M2c like TAM	CUL5	0.253067	0.46	0.211	1.23E-92	1.78E-88
M2c like TAM	PAFAH1B2	0.252398	0.563	0.304	1.71E-79	2.48E-75
M2c like TAM	SESTD1	0.251926	0.279	0.105	2.09E-84	3.04E-80
M2c like TAM	ARHGEF10L	0.251405	0.325	0.135	4.52E-81	6.57E-77
M2c like TAM	UBE2M	0.251054	0.565	0.288	1.44E-92	2.10E-88
M2c like TAM	ALDH1A1	0.250243	0.318	0.157	4.29E-52	6.25E-48
M1 like Macro	CXCL10	3.381733	0.755	0.127	0	0
M1 like Macro	ISG15	2.34811	0.912	0.581	5.62E-218	8.18E-214
M1 like Macro	CXCL9	2.199931	0.536	0.171	2.21E-189	3.21E-185
M1 like Macro	CCL8	2.191577	0.36	0.1	2.32E-136	3.38E-132
M1 like Macro	GBP1	1.966079	0.876	0.387	0	0

M1 like Macro	IFIT1	1.840755	0.529	0.135	2.49E-258	3.62E-254
M1 like Macro	CXCL11	1.762721	0.324	0.027	0	0
M1 like Macro	IFIT3	1.753421	0.723	0.207	0	0
M1 like Macro	IFIT2	1.598949	0.545	0.116	0	0
M1 like Macro	TNFSF10	1.547927	0.677	0.206	8.92E-297	1.30E-292
M1 like Macro	MX1	1.493253	0.717	0.281	5.08E-234	7.39E-230
M1 like Macro	RSAD2	1.423842	0.479	0.078	0	0
M1 like Macro	IFI6	1.323946	0.919	0.615	7.14E-155	1.04E-150
M1 like Macro	IFI44L	1.281084	0.675	0.214	2.42E-253	3.52E-249
M1 like Macro	GBP5	1.273063	0.604	0.18	1.58E-253	2.30E-249
M1 like Macro	ISG20	1.254482	0.556	0.157	5.95E-231	8.66E-227
M1 like Macro	FAM26F	1.236795	0.787	0.419	8.82E-159	1.28E-154
M1 like Macro	STAT1	1.221658	0.953	0.612	2.23E-233	3.24E-229
M1 like Macro	IFI27	1.20367	0.501	0.272	2.06E-58	3.00E-54
M1 like Macro	LY6E	1.194145	0.88	0.542	1.04E-151	1.52E-147
M1 like Macro	VAMP5	1.147515	0.865	0.48	3.72E-186	5.42E-182
M1 like Macro	IFITM3	1.128485	0.955	0.683	2.24E-169	3.26E-165
M1 like Macro	WARS	1.11338	0.717	0.341	1.21E-175	1.76E-171
M1 like Macro	IDO1	1.111126	0.264	0.023	1.53E-301	2.22E-297
M1 like Macro	IL4I1	1.101595	0.831	0.428	3.53E-185	5.14E-181
M1 like Macro	GBP4	1.058934	0.657	0.237	6.13E-217	8.93E-213
M1 like Macro	TAP1	1.025168	0.775	0.359	2.15E-214	3.13E-210
M1 like Macro	EPSTI1	1.01263	0.871	0.482	3.55E-207	5.16E-203
M1 like Macro	LAP3	0.977736	0.946	0.676	3.47E-197	5.04E-193

M1 like Macro	TNFSF13B	0.967552	0.96	0.687	3.76E-181	5.47E-177
M1 like Macro	SAMD9L	0.953371	0.708	0.31	5.87E-187	8.54E-183
M1 like Macro	ANKRD22	0.951411	0.543	0.174	8.76E-190	1.27E-185
M1 like Macro	OAS1	0.928328	0.641	0.257	2.36E-175	3.44E-171
M1 like Macro	IFIH1	0.908214	0.583	0.205	1.56E-188	2.27E-184
M1 like Macro	MX2	0.90384	0.523	0.189	3.67E-153	5.34E-149
M1 like Macro	PARP14	0.903054	0.72	0.426	1.94E-110	2.82E-106
M1 like Macro	OAS2	0.90222	0.602	0.213	5.52E-192	8.04E-188
M1 like Macro	PLEK	0.87783	0.825	0.568	1.55E-85	2.25E-81
M1 like Macro	IFI35	0.877518	0.794	0.391	7.36E-178	1.07E-173
M1 like Macro	PSMB9	0.842783	0.908	0.565	7.15E-163	1.04E-158
M1 like Macro	SNX10	0.813897	0.85	0.569	2.40E-106	3.50E-102
M1 like Macro	XAF1	0.812713	0.604	0.246	1.42E-151	2.07E-147
M1 like Macro	SLAMF7	0.810621	0.59	0.249	1.76E-130	2.57E-126
M1 like Macro	CMPK2	0.80845	0.468	0.101	1.16E-249	1.69E-245
M1 like Macro	RNF213	0.78594	0.861	0.572	1.73E-120	2.52E-116
M1 like Macro	CD40	0.78456	0.725	0.406	7.68E-115	1.12E-110
M1 like Macro	GCH1	0.784107	0.503	0.136	1.09E-212	1.58E-208
M1 like Macro	IFI44	0.77579	0.612	0.276	3.85E-132	5.61E-128
M1 like Macro	SERPING1	0.771069	0.74	0.372	1.90E-132	2.76E-128
M1 like Macro	RARRES3	0.759882	0.66	0.308	4.90E-128	7.13E-124
M1 like Macro	MT2A	0.758023	0.926	0.625	3.16E-110	4.60E-106

M1 like Macro	TYMP	0.756541	0.997	0.914	2.66E-146	3.87E-142
M1 like Macro	IRF7	0.754697	0.544	0.219	2.35E-137	3.42E-133
M1 like Macro	NT5C3A	0.754254	0.417	0.169	2.99E-96	4.34E-92
M1 like Macro	OAS3	0.733204	0.483	0.167	6.51E-144	9.47E-140
M1 like Macro	SP110	0.719812	0.663	0.329	4.62E-123	6.73E-119
M1 like Macro	APOBEC3A	0.692281	0.152	0.019	1.55E-125	2.26E-121
M1 like Macro	UBE2L6	0.687925	0.835	0.495	3.04E-141	4.43E-137
M1 like Macro	DYNLT1	0.684174	0.885	0.623	1.51E-111	2.20E-107
M1 like Macro	CD38	0.67929	0.562	0.215	3.98E-143	5.79E-139
M1 like Macro	HERC5	0.676848	0.363	0.123	8.62E-106	1.25E-101
M1 like Macro	APOL6	0.654614	0.679	0.371	7.76E-101	1.13E-96
M1 like Macro	IRF1	0.614435	0.497	0.293	2.32E-48	3.37E-44
M1 like Macro	CTSC	0.613322	0.987	0.866	2.62E-69	3.82E-65
M1 like Macro	PARP9	0.610124	0.63	0.323	9.80E-101	1.43E-96
M1 like Macro	EIF2AK2	0.609321	0.593	0.322	2.97E-83	4.32E-79
M1 like Macro	GBP2	0.593416	0.658	0.356	9.26E-96	1.35E-91
M1 like Macro	PLSCR1	0.591432	0.875	0.627	3.16E-90	4.59E-86
M1 like Macro	OASL	0.579905	0.309	0.065	1.38E-160	2.00E-156
M1 like Macro	HLA-A	0.577968	0.997	0.936	1.74E-94	2.53E-90
M1 like Macro	STAT2	0.57311	0.639	0.346	1.03E-89	1.50E-85
M1 like Macro	CD48	0.57055	0.811	0.537	3.84E-83	5.58E-79
M1 like Macro	NMI	0.569886	0.668	0.352	8.76E-107	1.27E-102
M1 like Macro	TRIM22	0.567365	0.579	0.278	1.87E-96	2.72E-92
M1 like Macro	CD274	0.560376	0.345	0.107	1.38E-109	2.01E-105
M1 like Macro	CD47	0.55872	0.866	0.633	5.85E-86	8.51E-82
M1 like Macro	PLEKHO1	0.553785	0.765	0.521	1.11E-69	1.62E-65
M1 like Macro	DRAM1	0.551382	0.687	0.418	2.39E-74	3.47E-70

M1 like Macro	USP18	0.541691	0.307	0.061	1.80E-172	2.61E-168
M1 like Macro	APOL3	0.537601	0.415	0.137	2.95E-124	4.29E-120
M1 like Macro	GIMAP4	0.536795	0.798	0.517	1.06E-84	1.54E-80
M1 like Macro	BST2	0.532925	0.953	0.736	1.23E-81	1.79E-77
M1 like Macro	HAPLN3	0.529455	0.369	0.092	8.39E-158	1.22E-153
M1 like Macro	LILRB4	0.519135	0.875	0.65	3.65E-68	5.31E-64
M1 like Macro	HLA-B	0.517671	0.999	0.96	3.51E-97	5.11E-93
M1 like Macro	SERPINA1	0.498262	0.843	0.633	1.82E-51	2.65E-47
M1 like Macro	ICAM1	0.491728	0.686	0.452	6.03E-48	8.77E-44
M1 like Macro	LYN	0.479419	0.853	0.631	2.21E-57	3.22E-53
M1 like Macro	BAZ1A	0.477672	0.657	0.409	1.90E-61	2.76E-57
M1 like Macro	NUB1	0.475892	0.559	0.303	6.02E-69	8.75E-65
M1 like Macro	PSTPIP2	0.475339	0.409	0.141	6.61E-110	9.62E-106
M1 like Macro	PML	0.463931	0.437	0.176	1.27E-93	1.85E-89
M1 like Macro	SAMD9	0.45911	0.409	0.21	4.34E-54	6.32E-50
M1 like Macro	LYSMD2	0.458307	0.679	0.381	1.27E-82	1.85E-78
M1 like Macro	HLA-C	0.458113	0.991	0.913	1.58E-64	2.29E-60
M1 like Macro	SQRDL	0.45723	0.805	0.53	1.18E-74	1.72E-70
M1 like Macro	ATF5	0.448474	0.679	0.401	2.73E-67	3.97E-63
M1 like Macro	NAGK	0.44669	0.799	0.556	5.23E-61	7.61E-57
M1 like Macro	LGALS3BP	0.445573	0.666	0.43	3.80E-49	5.52E-45
M1 like Macro	MNDA	0.444778	0.877	0.662	5.73E-49	8.34E-45
M1 like Macro	DDX60L	0.441198	0.383	0.179	5.69E-58	8.28E-54
M1 like Macro	SLC31A2	0.436428	0.712	0.452	1.26E-65	1.84E-61
M1 like Macro	NCF1	0.428339	0.68	0.447	2.83E-44	4.12E-40
M1 like Macro	IFITM1	0.423793	0.19	0.036	6.12E-105	8.91E-101
M1 like Macro	TFEC	0.423772	0.661	0.419	1.37E-57	2.00E-53
M1 like Macro	GMPR	0.422575	0.243	0.05	3.33E-125	4.85E-121
M1 like Macro	RABGAP1L	0.414678	0.363	0.169	3.30E-55	4.80E-51
M1 like Macro	SLAMF8	0.413642	0.75	0.525	4.37E-52	6.36E-48
M1 like Macro	TCN2	0.412122	0.619	0.356	1.04E-61	1.51E-57
M1 like Macro	DDX58	0.40501	0.276	0.077	4.71E-100	6.85E-96
M1 like Macro	PLA2G7	0.401462	0.839	0.674	1.33E-33	1.94E-29
M1 like Macro	CASP4	0.401211	0.705	0.458	1.08E-62	1.57E-58
M1 like Macro	B2M	0.393876	1	0.998	1.83E-88	2.67E-84

M1 like Macro	DTX3L	0.391547	0.535	0.296	5.92E-59	8.61E-55
M1 like Macro	GRINA	0.381172	0.866	0.658	1.63E-45	2.38E-41
M1 like Macro	XRN1	0.375931	0.422	0.212	3.85E-54	5.60E-50
M1 like Macro	DNAJA1	0.375518	0.836	0.624	1.27E-45	1.85E-41
M1 like Macro	ADAR	0.372406	0.699	0.488	1.27E-42	1.85E-38
M1 like Macro	NAPA	0.370969	0.686	0.431	2.85E-62	4.14E-58
M1 like Macro	CD86	0.367205	0.852	0.653	1.71E-43	2.49E-39
M1 like Macro	HSPA5	0.36483	0.871	0.675	9.35E-36	1.36E-31
M1 like Macro	RNF19B	0.363182	0.411	0.236	4.45E-36	6.47E-32
M1 like Macro	SECTM1	0.362285	0.482	0.231	2.92E-68	4.25E-64
M1 like Macro	LGALS9	0.356084	0.839	0.621	5.34E-48	7.77E-44
M1 like Macro	CBR1	0.35543	0.621	0.39	3.74E-49	5.44E-45
M1 like Macro	PSMA3	0.35324	0.787	0.55	9.16E-55	1.33E-50
M1 like Macro	HES4	0.352203	0.261	0.147	1.44E-21	2.10E-17
M1 like Macro	SRI	0.349308	0.746	0.507	9.52E-53	1.39E-48
M1 like Macro	PDIA3	0.348365	0.902	0.742	3.13E-37	4.56E-33
M1 like Macro	TAP2	0.344759	0.455	0.231	7.09E-59	1.03E-54
M1 like Macro	P2RY6	0.342722	0.433	0.221	8.13E-51	1.18E-46
M1 like Macro	RTCB	0.340587	0.573	0.363	3.42E-42	4.98E-38
M1 like Macro	LACTB	0.330345	0.673	0.453	1.17E-42	1.70E-38
M1 like Macro	CSF2RB	0.329238	0.45	0.265	3.41E-37	4.95E-33
M1 like Macro	CALR	0.32266	0.951	0.824	1.25E-27	1.82E-23
M1 like Macro	SCIMP	0.321727	0.541	0.295	5.40E-56	7.85E-52
M1 like Macro	LILRB1	0.320299	0.526	0.326	2.60E-37	3.78E-33
M1 like Macro	PARP12	0.318445	0.475	0.269	1.36E-45	1.98E-41
M1 like Macro	APOL4	0.316369	0.29	0.1	6.00E-73	8.73E-69
M1 like Macro	MYD88	0.313871	0.455	0.259	3.71E-41	5.40E-37
M1 like Macro	IFIT5	0.309529	0.312	0.125	5.78E-61	8.42E-57
M1 like Macro	SUCNR1	0.307517	0.353	0.197	2.38E-32	3.46E-28
M1 like Macro	ZNFX1	0.301734	0.387	0.213	1.78E-37	2.59E-33
M1 like Macro	HELZ2	0.298742	0.222	0.071	1.40E-62	2.04E-58
M1 like Macro	STX11	0.298467	0.496	0.283	4.11E-44	5.98E-40
M1 like Macro	NR1H3	0.29636	0.495	0.327	8.26E-28	1.20E-23
M1 like Macro	SDS	0.292553	0.516	0.387	1.79E-13	2.60E-09
M1 like Macro	ST3GAL5	0.292491	0.415	0.233	8.21E-38	1.19E-33
M1 like Macro	SERPINB1	0.29216	0.815	0.629	8.26E-35	1.20E-30
M1 like Macro	ETV7	0.291922	0.204	0.026	4.47E-168	6.51E-164
M1 like Macro	P2RX7	0.287848	0.331	0.178	9.99E-33	1.45E-28
M1 like Macro	MYOF	0.286304	0.357	0.181	9.44E-41	1.37E-36
M1 like Macro	IL15RA	0.284657	0.288	0.103	6.42E-68	9.34E-64
M1 like Macro	SPTLC2	0.283242	0.372	0.203	2.91E-36	4.23E-32

M1 like Macro	FPR2	0.281899	0.224	0.079	3.17E-52	4.61E-48
M1 like Macro	ERP44	0.281415	0.741	0.569	1.24E-31	1.81E-27
M1 like Macro	CD63	0.281405	0.996	0.948	1.70E-32	2.48E-28
M1 like Macro	HPSE	0.28083	0.394	0.245	4.07E-26	5.92E-22
M1 like Macro	DDX60	0.280774	0.304	0.133	8.63E-50	1.26E-45
M1 like Macro	PDCD1LG2	0.280221	0.298	0.12	8.59E-56	1.25E-51
M1 like Macro	IL27	0.279752	0.165	0.021	5.15E-136	7.50E-132
M1 like Macro	B4GALT5	0.278056	0.335	0.19	2.14E-28	3.11E-24
M1 like Macro	CD53	0.277627	0.947	0.801	8.37E-32	1.22E-27
M1 like Macro	CD80	0.275988	0.329	0.154	2.81E-45	4.08E-41
M1 like Macro	APOL2	0.272866	0.359	0.175	1.27E-46	1.84E-42
M1 like Macro	KCNMA1	0.272224	0.561	0.38	3.65E-27	5.31E-23
M1 like Macro	UBE2D1	0.271159	0.673	0.483	4.09E-32	5.96E-28
M1 like Macro	APOL1	0.268054	0.31	0.128	1.25E-55	1.82E-51
M1 like Macro	ACOT9	0.266702	0.511	0.328	7.81E-33	1.14E-28
M1 like Macro	NBN	0.263592	0.412	0.24	4.41E-35	6.42E-31
M1 like Macro	PTPN2	0.262479	0.547	0.359	7.11E-31	1.03E-26
M1 like Macro	IL18BP	0.260564	0.409	0.239	5.77E-32	8.40E-28
M1 like Macro	GLA	0.255633	0.612	0.427	5.47E-27	7.96E-23
M1 like Macro	C19orf66	0.252713	0.359	0.194	1.54E-35	2.25E-31
M1 like Macro	JAK2	0.252613	0.299	0.144	6.49E-39	9.45E-35
M1 like Macro	LILRA5	0.25084	0.315	0.169	1.81E-30	2.64E-26
M1 like Macro	ACP2	0.250345	0.682	0.483	2.18E-31	3.17E-27
GNLY+ Monolike Macro	GNLY	2.772225	0.404	0.036	0	0
GNLY+ Monolike Macro	GZMA	2.601719	0.565	0.026	0	0
GNLY+ Monolike Macro	CD3D	2.207493	0.769	0.025	0	0
GNLY+ Monolike Macro	IL32	2.164363	0.863	0.188	0	0
GNLY+ Monolike Macro	CCL5	2.160681	0.679	0.158	2.23E-297	3.25E-293
GNLY+ Monolike Macro	GZMB	2.07655	0.459	0.024	0	0
GNLY+ Monolike Macro	KLRB1	1.824588	0.498	0.017	0	0
GNLY+ Monolike Macro	CD2	1.820283	0.716	0.02	0	0
GNLY+ Monolike Macro	CD7	1.801639	0.646	0.026	0	0
GNLY+ Monolike Macro	CD69	1.699124	0.688	0.106	0	0
GNLY+ Monolike Macro	CXCL13	1.650158	0.263	0.012	0	0
GNLY+ Monolike Macro	LTB	1.485407	0.529	0.106	2.74E-243	3.99E-239
GNLY+ Monolike Macro	CD3E	1.403688	0.64	0.015	0	0
GNLY+ Monolike Macro	NKG7	1.392235	0.579	0.194	9.74E-145	1.42E-140

GNLY+ Monolike Macro	MZB1	1.359042	0.223	0.025	1.84E-162	2.67E-158
GNLY+ Monolike Macro	CD3G	1.246806	0.555	0.009	0	0
GNLY+ Monolike Macro	TNFRSF18	1.034422	0.407	0.021	0	0
GNLY+ Monolike Macro	ETS1	1.032028	0.505	0.033	0	0
GNLY+ Monolike Macro	BIRC3	1.006652	0.588	0.147	8.82E-202	1.28E-197
GNLY+ Monolike Macro	CD52	0.997214	0.839	0.367	1.78E-149	2.59E-145
GNLY+ Monolike Macro	CD27	0.961611	0.412	0.01	0	0
GNLY+ Monolike Macro	TNFRSF4	0.942365	0.296	0.039	1.61E-191	2.34E-187
GNLY+ Monolike Macro	FKBP11	0.918176	0.312	0.046	2.50E-182	3.63E-178
GNLY+ Monolike Macro	GPR171	0.914549	0.389	0.015	0	0
GNLY+ Monolike Macro	TNFAIP3	0.910598	0.872	0.478	5.55E-104	8.07E-100
GNLY+ Monolike Macro	FYN	0.909737	0.474	0.026	0	0
GNLY+ Monolike Macro	KLRD1	0.909663	0.318	0.008	0	0
GNLY+ Monolike Macro	RORA	0.902561	0.426	0.015	0	0
GNLY+ Monolike Macro	DUSP4	0.876924	0.535	0.147	7.73E-159	1.13E-154
GNLY+ Monolike Macro	DERL3	0.851179	0.146	0.03	2.26E-58	3.29E-54
GNLY+ Monolike Macro	ACAP1	0.844278	0.559	0.108	1.84E-265	2.67E-261
GNLY+ Monolike Macro	IKZF3	0.837975	0.426	0.011	0	0
GNLY+ Monolike Macro	SPOCK2	0.837275	0.43	0.009	0	0
GNLY+ Monolike Macro	LCK	0.817264	0.441	0.01	0	0
GNLY+ Monolike Macro	PIM2	0.811451	0.348	0.046	3.13E-224	4.55E-220
GNLY+ Monolike Macro	ITM2A	0.807149	0.375	0.007	0	0
GNLY+ Monolike Macro	GZMH	0.796058	0.26	0.005	0	0
GNLY+ Monolike Macro	CLEC2D	0.782496	0.505	0.071	0	0
GNLY+ Monolike Macro	CD8A	0.779595	0.302	0.006	0	0
GNLY+ Monolike Macro	TIGIT	0.779515	0.366	0.006	0	0
GNLY+ Monolike Macro	XBP1	0.772719	0.716	0.513	1.07E-23	1.56E-19
GNLY+ Monolike Macro	BATF	0.772642	0.459	0.142	4.14E-116	6.02E-112
GNLY+ Monolike Macro	CTLA4	0.763403	0.328	0.012	0	0
GNLY+ Monolike Macro	HOPX	0.747504	0.277	0.011	0	0
GNLY+ Monolike Macro	GZMK	0.745628	0.19	0.005	0	0
GNLY+ Monolike Macro	ID2	0.737393	0.81	0.505	2.30E-66	3.34E-62

GNLY+ Monolike Macro	CD96	0.730107	0.36	0.01	0	0
GNLY+ Monolike Macro	CCND2	0.714387	0.521	0.176	4.04E-115	5.88E-111
GNLY+ Monolike Macro	SSR4	0.707295	0.956	0.812	5.47E-22	7.97E-18
GNLY+ Monolike Macro	IL2RB	0.705367	0.36	0.005	0	0
GNLY+ Monolike Macro	PRF1	0.704104	0.328	0.011	0	0
GNLY+ Monolike Macro	IFNG	0.703759	0.22	0.005	0	0
GNLY+ Monolike Macro	IL2RG	0.69766	0.745	0.386	4.88E-85	7.11E-81
GNLY+ Monolike Macro	CYTIP	0.686932	0.729	0.362	6.73E-99	9.79E-95
GNLY+ Monolike Macro	EVL	0.677796	0.717	0.355	8.42E-92	1.22E-87
GNLY+ Monolike Macro	OCIAD2	0.657357	0.483	0.091	1.26E-220	1.84E-216
GNLY+ Monolike Macro	STK17A	0.653342	0.497	0.15	7.77E-132	1.13E-127
GNLY+ Monolike Macro	CTSW	0.641223	0.289	0.019	0	0
GNLY+ Monolike Macro	BCL11B	0.626273	0.324	0.007	0	0
GNLY+ Monolike Macro	PTPN7	0.625568	0.477	0.162	1.85E-102	2.69E-98
GNLY+ Monolike Macro	RAC2	0.623079	0.745	0.422	4.35E-77	6.33E-73
GNLY+ Monolike Macro	CD247	0.616816	0.336	0.007	0	0
GNLY+ Monolike Macro	CST7	0.615551	0.495	0.142	1.40E-126	2.03E-122
GNLY+ Monolike Macro	FAM46C	0.606938	0.295	0.012	0	0
GNLY+ Monolike Macro	VAMP2	0.603056	0.764	0.427	5.80E-77	8.43E-73
GNLY+ Monolike Macro	DNAJB1	0.585552	0.74	0.502	1.93E-34	2.80E-30
GNLY+ Monolike Macro	IKZF1	0.583	0.638	0.299	1.03E-84	1.50E-80
GNLY+ Monolike Macro	CD79A	0.581545	0.161	0.011	1.98E-171	2.87E-167
GNLY+ Monolike Macro	RASGRP1	0.563159	0.328	0.017	0	0
GNLY+ Monolike Macro	ANKRD28	0.557713	0.471	0.199	1.20E-64	1.74E-60
GNLY+ Monolike Macro	RAB27A	0.553486	0.426	0.124	2.64E-111	3.84E-107
GNLY+ Monolike Macro	SYNE2	0.55138	0.316	0.026	4.97E-304	7.23E-300
GNLY+ Monolike Macro	RUNX3	0.550344	0.53	0.22	9.21E-78	1.34E-73
GNLY+ Monolike Macro	PPP2R5C	0.532602	0.682	0.383	2.22E-61	3.22E-57
GNLY+ Monolike Macro	ICAM3	0.531635	0.398	0.069	2.35E-194	3.42E-190
GNLY+ Monolike Macro	SLC38A1	0.522137	0.347	0.034	5.45E-285	7.93E-281
GNLY+ Monolike Macro	DDIT4	0.510973	0.584	0.282	1.26E-60	1.83E-56
GNLY+ Monolike Macro	SH2D2A	0.508949	0.289	0.014	0	0

GNLY+ Monolike Macro	1-Sep	0.506964	0.309	0.035	3.62E-228	5.26E-224
GNLY+ Monolike Macro	LBH	0.505108	0.305	0.013	0	0
GNLY+ Monolike Macro	SEC11C	0.498622	0.641	0.437	1.40E-25	2.04E-21
GNLY+ Monolike Macro	LAG3	0.49426	0.348	0.121	4.47E-63	6.51E-59
GNLY+ Monolike Macro	C12orf75	0.492499	0.298	0.061	6.46E-117	9.39E-113
GNLY+ Monolike Macro	H1FX	0.488974	0.643	0.375	1.76E-44	2.57E-40
GNLY+ Monolike Macro	ITGA1	0.487443	0.255	0.008	0	0
GNLY+ Monolike Macro	EEF1D	0.485558	0.991	0.875	5.57E-60	8.11E-56
GNLY+ Monolike Macro	LIMD2	0.481313	0.546	0.217	2.08E-84	3.03E-80
GNLY+ Monolike Macro	PYHIN1	0.477988	0.258	0.008	0	0
GNLY+ Monolike Macro	ODF2L	0.476544	0.368	0.097	1.78E-106	2.59E-102
GNLY+ Monolike Macro	TBC1D10C	0.469553	0.336	0.059	1.48E-159	2.16E-155
GNLY+ Monolike Macro	FNBP1	0.467323	0.714	0.443	8.75E-49	1.27E-44
GNLY+ Monolike Macro	MGEA5	0.466588	0.552	0.284	5.38E-52	7.84E-48
GNLY+ Monolike Macro	LAT	0.465381	0.345	0.063	7.05E-156	1.03E-151
GNLY+ Monolike Macro	ANAPC16	0.459747	0.825	0.532	3.65E-59	5.31E-55
GNLY+ Monolike Macro	6-Sep	0.459302	0.546	0.273	2.01E-56	2.92E-52
GNLY+ Monolike Macro	SYTL3	0.456318	0.33	0.065	3.31E-137	4.81E-133
GNLY+ Monolike Macro	LPIN1	0.448739	0.4	0.124	4.89E-92	7.12E-88
GNLY+ Monolike Macro	PRKCH	0.447645	0.334	0.083	7.39E-104	1.07E-99
GNLY+ Monolike Macro	RNF19A	0.446074	0.406	0.179	5.11E-50	7.44E-46
GNLY+ Monolike Macro	CXCR6	0.446031	0.24	0.003	0	0
GNLY+ Monolike Macro	CD8B	0.443596	0.195	0.003	0	0
GNLY+ Monolike Macro	KLRC1	0.443141	0.105	0.002	2.49E-204	3.62E-200
GNLY+ Monolike Macro	ITK	0.441829	0.252	0.007	0	0
GNLY+ Monolike Macro	ICOS	0.439091	0.202	0.003	0	0
GNLY+ Monolike Macro	C12orf57	0.435904	0.653	0.337	1.27E-59	1.85E-55
GNLY+ Monolike Macro	SOCS1	0.433298	0.441	0.172	1.58E-67	2.31E-63
GNLY+ Monolike Macro	STK4	0.432071	0.691	0.417	3.86E-49	5.61E-45
GNLY+ Monolike Macro	TUBA4A	0.431811	0.29	0.05	2.65E-139	3.86E-135
GNLY+ Monolike Macro	GATA3	0.424963	0.246	0.02	9.57E-235	1.39E-230

GNLY+ Monolike Macro	OXNAD1	0.424684	0.274	0.042	2.75E-148	4.00E-144
GNLY+ Monolike Macro	SKAP1	0.421334	0.274	0.006	0	0
GNLY+ Monolike Macro	PCSK7	0.416386	0.535	0.262	1.08E-56	1.57E-52
GNLY+ Monolike Macro	KIAA1551	0.415964	0.488	0.258	2.13E-40	3.10E-36
GNLY+ Monolike Macro	GLCCI1	0.412374	0.277	0.059	2.83E-101	4.11E-97
GNLY+ Monolike Macro	CD6	0.408169	0.243	0.011	0	0
GNLY+ Monolike Macro	SLFN5	0.40609	0.432	0.18	6.26E-59	9.10E-55
GNLY+ Monolike Macro	KLRC2	0.405714	0.15	0.003	1.04E-291	1.52E-287
GNLY+ Monolike Macro	G3BP2	0.403422	0.57	0.341	4.13E-36	6.01E-32
GNLY+ Monolike Macro	RHOF	0.399623	0.342	0.111	5.52E-72	8.03E-68
GNLY+ Monolike Macro	CYFIP2	0.397643	0.231	0.012	1.28E-296	1.87E-292
GNLY+ Monolike Macro	BCL2L11	0.395813	0.371	0.142	4.92E-57	7.15E-53
GNLY+ Monolike Macro	C9orf142	0.392443	0.574	0.286	6.22E-58	9.06E-54
GNLY+ Monolike Macro	TERF2IP	0.388717	0.631	0.375	1.03E-42	1.50E-38
GNLY+ Monolike Macro	APOBEC3C	0.387985	0.497	0.274	9.97E-38	1.45E-33
GNLY+ Monolike Macro	LEPROTL1	0.382695	0.761	0.505	1.86E-42	2.71E-38
GNLY+ Monolike Macro	PDE4D	0.374771	0.236	0.028	5.54E-164	8.06E-160
GNLY+ Monolike Macro	SH2D1A	0.372786	0.21	0.004	0	0
GNLY+ Monolike Macro	CDC42SE2	0.372102	0.67	0.398	2.52E-42	3.67E-38
GNLY+ Monolike Macro	ERN1	0.369424	0.304	0.126	1.16E-39	1.69E-35
GNLY+ Monolike Macro	PPM1K	0.368797	0.384	0.158	7.12E-51	1.04E-46
GNLY+ Monolike Macro	PBXIP1	0.363558	0.337	0.108	6.63E-72	9.65E-68
GNLY+ Monolike Macro	TSTD1	0.361053	0.518	0.233	4.13E-60	6.01E-56
GNLY+ Monolike Macro	NDFIP2	0.359736	0.252	0.027	4.09E-192	5.95E-188
GNLY+ Monolike Macro	TRAF3IP3	0.35745	0.362	0.133	3.43E-59	4.99E-55
GNLY+ Monolike Macro	ZNRF1	0.357355	0.234	0.02	1.54E-213	2.24E-209
GNLY+ Monolike Macro	LYST	0.355844	0.363	0.137	4.01E-57	5.83E-53
GNLY+ Monolike Macro	CCDC88C	0.355544	0.24	0.022	1.12E-204	1.63E-200
GNLY+ Monolike Macro	TRAT1	0.353964	0.202	0.003	0	0
GNLY+ Monolike Macro	SMCHD1	0.353133	0.454	0.237	7.05E-36	1.03E-31
GNLY+ Monolike Macro	KLF13	0.352771	0.381	0.179	1.46E-39	2.13E-35
GNLY+ Monolike Macro	FOXP3	0.352525	0.15	0.002	0	0
GNLY+ Monolike Macro	PTPN22	0.351802	0.398	0.174	5.21E-48	7.58E-44
GNLY+ Monolike Macro	CHST12	0.351308	0.325	0.114	4.02E-58	5.85E-54

GNLY+ Monolike Macro	GRAP2	0.347623	0.205	0.004	0	0
GNLY+ Monolike Macro	JAK3	0.344254	0.325	0.099	1.25E-73	1.81E-69
GNLY+ Monolike Macro	APOBEC3G	0.341563	0.4	0.179	1.84E-47	2.68E-43
GNLY+ Monolike Macro	SEMA4D	0.341329	0.448	0.21	8.27E-46	1.20E-41
GNLY+ Monolike Macro	ARHGEF1	0.340514	0.406	0.174	1.27E-50	1.85E-46
GNLY+ Monolike Macro	SIT1	0.339003	0.226	0.02	1.36E-205	1.98E-201
GNLY+ Monolike Macro	GZMM	0.338897	0.184	0.004	0	0
GNLY+ Monolike Macro	SPCS1	0.336038	0.895	0.687	7.37E-26	1.07E-21
GNLY+ Monolike Macro	RSBN1L	0.335704	0.494	0.258	3.21E-40	4.68E-36
GNLY+ Monolike Macro	PDE7A	0.335293	0.34	0.131	3.64E-51	5.30E-47
GNLY+ Monolike Macro	CDKN1B	0.331989	0.467	0.231	1.86E-41	2.71E-37
GNLY+ Monolike Macro	GIMAP7	0.329101	0.559	0.303	5.37E-40	7.81E-36
GNLY+ Monolike Macro	CBLB	0.323307	0.35	0.148	4.43E-43	6.45E-39
GNLY+ Monolike Macro	TMIGD2	0.321661	0.146	0.003	1.59E-294	2.32E-290
GNLY+ Monolike Macro	PRDX4	0.319061	0.6	0.452	9.36E-13	1.36E-08
GNLY+ Monolike Macro	BTN3A2	0.317099	0.421	0.185	2.58E-49	3.76E-45
GNLY+ Monolike Macro	SIRPG	0.314627	0.196	0.003	0	0
GNLY+ Monolike Macro	FAM107B	0.313035	0.565	0.338	4.57E-30	6.65E-26
GNLY+ Monolike Macro	TRIM56	0.309314	0.441	0.222	5.48E-38	7.98E-34
GNLY+ Monolike Macro	RASSF5	0.307147	0.451	0.224	2.30E-38	3.35E-34
GNLY+ Monolike Macro	ZAP70	0.302861	0.191	0.004	0	0
GNLY+ Monolike Macro	ADAM19	0.301795	0.243	0.045	1.00E-103	1.46E-99
GNLY+ Monolike Macro	SLAMF1	0.296175	0.181	0.015	1.60E-168	2.33E-164
GNLY+ Monolike Macro	OPTN	0.295759	0.348	0.132	5.69E-53	8.28E-49
GNLY+ Monolike Macro	EPC1	0.294494	0.429	0.218	3.45E-34	5.02E-30
GNLY+ Monolike Macro	RCAN3	0.294033	0.295	0.106	2.29E-50	3.34E-46
GNLY+ Monolike Macro	CD79B	0.291552	0.181	0.013	9.72E-183	1.41E-178
GNLY+ Monolike Macro	CCDC12	0.288783	0.468	0.25	1.34E-35	1.95E-31
GNLY+ Monolike Macro	GPR174	0.2884	0.169	0.002	0	0
GNLY+ Monolike Macro	AIM1	0.287018	0.404	0.224	4.67E-26	6.79E-22
GNLY+ Monolike Macro	DUSP5	0.286443	0.242	0.074	2.53E-52	3.68E-48
GNLY+ Monolike Macro	PPP1R2	0.285325	0.643	0.412	1.36E-30	1.98E-26
GNLY+ Monolike Macro	PIM1	0.280727	0.36	0.169	3.15E-34	4.58E-30
GNLY+ Monolike Macro	GADD45A	0.280486	0.263	0.091	5.61E-46	8.16E-42
GNLY+ Monolike Macro	POU2AF1	0.280459	0.114	0.005	5.37E-165	7.81E-161
GNLY+ Monolike Macro	RSBN1	0.277579	0.334	0.139	1.32E-42	1.93E-38

GNLY+ Monolike Macro	MAST4	0.27743	0.178	0.014	1.05E-173	1.53E-169
GNLY+ Monolike Macro	TAGAP	0.273733	0.453	0.239	1.25E-32	1.82E-28
GNLY+ Monolike Macro	CLIC3	0.27353	0.137	0.009	1.26E-152	1.84E-148
GNLY+ Monolike Macro	TSPYL2	0.272754	0.246	0.059	2.12E-76	3.09E-72
GNLY+ Monolike Macro	SCML4	0.271484	0.129	0.004	4.62E-227	6.72E-223
GNLY+ Monolike Macro	STAT4	0.266499	0.251	0.076	5.76E-56	8.38E-52
GNLY+ Monolike Macro	C9orf16	0.265309	0.805	0.604	4.61E-27	6.70E-23
GNLY+ Monolike Macro	EPB41	0.260663	0.283	0.112	1.32E-39	1.92E-35
GNLY+ Monolike Macro	PTK2B	0.260627	0.445	0.254	7.16E-26	1.04E-21
GNLY+ Monolike Macro	VPS37B	0.259787	0.284	0.124	8.46E-33	1.23E-28
GNLY+ Monolike Macro	FAM102A	0.259592	0.157	0.021	1.76E-96	2.56E-92
GNLY+ Monolike Macro	ERAP2	0.258469	0.375	0.199	2.17E-27	3.16E-23
GNLY+ Monolike Macro	TRAF5	0.257891	0.257	0.083	2.77E-51	4.04E-47
GNLY+ Monolike Macro	SUPT3H	0.257574	0.184	0.035	6.31E-77	9.19E-73
GNLY+ Monolike Macro	ITGAL	0.256642	0.29	0.135	1.09E-28	1.58E-24
GNLY+ Monolike Macro	TTC39C	0.255871	0.246	0.083	9.41E-47	1.37E-42
GNLY+ Monolike Macro	MLLT6	0.255475	0.343	0.184	1.52E-23	2.22E-19
GNLY+ Monolike Macro	THEMIS	0.255322	0.138	0.001	0	0
GNLY+ Monolike Macro	CAMK4	0.25353	0.147	0.002	0	0
GNLY+ Monolike Macro	PTPN4	0.252867	0.19	0.036	2.39E-79	3.47E-75
GNLY+ Monolike Macro	LAIR2	0.252435	0.105	0.008	1.17E-108	1.70E-104
MKI67+ Macro	HIST1H4C	2.65031	0.767	0.289	3.11E-280	4.52E-276
MKI67+ Macro	TOP2A	1.97224	0.622	0.024	0	0
MKI67+ Macro	MKI67	1.526096	0.588	0.019	0	0
MKI67+ Macro	CENPF	1.316617	0.468	0.025	0	0
MKI67+ Macro	DEK	1.275619	0.949	0.589	7.65E-290	1.11E-285
MKI67+ Macro	NUCKS1	1.157616	0.94	0.593	3.97E-245	5.78E-241
MKI67+ Macro	SMC4	1.13969	0.673	0.166	0	0
MKI67+ Macro	CCNB1	1.089686	0.38	0.032	0	0
MKI67+ Macro	MCM7	1.07588	0.588	0.064	0	0
MKI67+ Macro	RAD51AP1	1.008761	0.617	0.104	0	0
MKI67+ Macro	TMPO	0.971766	0.696	0.218	2.45E-262	3.57E-258
MKI67+ Macro	NASP	0.963705	0.747	0.322	4.46E-199	6.48E-195

MKI67+ Macro	PARP1	0.962551	0.794	0.365	5.50E-202	8.00E-198
MKI67+ Macro	DHFR	0.916066	0.528	0.07	0	0
MKI67+ Macro	AURKB	0.876499	0.387	0.012	0	0
MKI67+ Macro	GTSE1	0.856428	0.395	0.007	0	0
MKI67+ Macro	CEP55	0.853711	0.45	0.014	0	0
MKI67+ Macro	HELLS	0.814917	0.45	0.027	0	0
MKI67+ Macro	UHRF1	0.800809	0.453	0.013	0	0
MKI67+ Macro	MCM6	0.776295	0.425	0.079	8.49E-257	1.24E-252
MKI67+ Macro	ARL6IP1	0.767738	0.92	0.735	1.11E-54	1.62E-50
MKI67+ Macro	RRM1	0.765773	0.529	0.105	1.28E-305	1.86E-301
MKI67+ Macro	SHCBP1	0.757427	0.462	0.018	0	0
MKI67+ Macro	ASPM	0.744485	0.318	0.008	0	0
MKI67+ Macro	RAD21	0.722432	0.708	0.353	7.73E-123	1.13E-118
MKI67+ Macro	MCM3	0.714727	0.489	0.105	1.93E-256	2.81E-252
MKI67+ Macro	ATAD2	0.69102	0.418	0.042	0	0
MKI67+ Macro	DDX39A	0.675352	0.617	0.271	1.62E-127	2.36E-123
MKI67+ Macro	USP1	0.67419	0.515	0.15	7.84E-184	1.14E-179
MKI67+ Macro	CKAP2	0.661746	0.423	0.081	2.06E-237	3.00E-233
MKI67+ Macro	ANLN	0.657738	0.325	0.007	0	0
MKI67+ Macro	PLK1	0.635442	0.271	0.009	0	0
MKI67+ Macro	MCM4	0.630961	0.434	0.061	0	0
MKI67+ Macro	ZDHHC12	0.626393	0.707	0.345	5.78E-118	8.41E-114
MKI67+ Macro	RACGAP1	0.624629	0.398	0.058	4.53E-289	6.59E-285
MKI67+ Macro	SET	0.615633	0.913	0.665	1.10E-87	1.60E-83
MKI67+ Macro	MYBL2	0.611481	0.334	0.01	0	0
MKI67+ Macro	BRCA1	0.608811	0.361	0.031	0	0
MKI67+ Macro	DLGAP5	0.600629	0.288	0.006	0	0
MKI67+ Macro	BRI3BP	0.599674	0.515	0.171	6.57E-144	9.55E-140
MKI67+ Macro	CCDC34	0.597542	0.38	0.051	8.96E-298	1.30E-293
MKI67+ Macro	KPNB1	0.596669	0.816	0.513	1.22E-86	1.78E-82

MKI67+ Macro	PRC1	0.593572	0.338	0.015	0	0
MKI67+ Macro	FADS1	0.593279	0.459	0.172	1.68E-104	2.44E-100
MKI67+ Macro	HNRNPD	0.571364	0.818	0.511	7.41E-95	1.08E-90
MKI67+ Macro	HMGA1	0.562497	0.691	0.401	1.42E-80	2.06E-76
MKI67+ Macro	SMC3	0.554447	0.596	0.272	2.41E-101	3.51E-97
MKI67+ Macro	SMC1A	0.554402	0.589	0.264	1.44E-103	2.09E-99
MKI67+ Macro	CACYBP	0.545034	0.811	0.507	2.92E-90	4.25E-86
MKI67+ Macro	PAK1	0.535322	0.649	0.32	5.33E-99	7.75E-95
MKI67+ Macro	CASC5	0.533381	0.277	0.014	0	0
MKI67+ Macro	TROAP	0.530221	0.273	0.011	0	0
MKI67+ Macro	PGP	0.522655	0.614	0.28	4.11E-106	5.97E-102
MKI67+ Macro	ORC6	0.513687	0.366	0.031	0	0
MKI67+ Macro	SSRP1	0.513345	0.527	0.196	8.52E-125	1.24E-120
MKI67+ Macro	SDF2L1	0.511319	0.83	0.532	2.80E-72	4.08E-68
MKI67+ Macro	TFDP1	0.508358	0.569	0.237	8.99E-113	1.31E-108
MKI67+ Macro	TMEM109	0.505515	0.615	0.297	8.87E-97	1.29E-92
MKI67+ Macro	BARD1	0.502872	0.375	0.066	1.20E-222	1.75E-218
MKI67+ Macro	HSP90AA1	0.501799	0.986	0.918	1.47E-63	2.13E-59
MKI67+ Macro	NCAPD2	0.500139	0.334	0.051	1.10E-228	1.60E-224
MKI67+ Macro	MCM2	0.494269	0.306	0.024	0	0
MKI67+ Macro	PAICS	0.484434	0.456	0.15	1.38E-125	2.01E-121
MKI67+ Macro	E2F1	0.484106	0.283	0.01	0	0
MKI67+ Macro	KIFC1	0.480958	0.275	0.007	0	0
MKI67+ Macro	MLEC	0.480222	0.773	0.482	4.48E-76	6.52E-72
MKI67+ Macro	ERP29	0.466409	0.884	0.657	1.39E-64	2.03E-60
MKI67+ Macro	SRSF7	0.454148	0.829	0.566	1.01E-65	1.47E-61
MKI67+ Macro	GLRX5	0.452981	0.646	0.326	6.23E-89	9.06E-85
MKI67+ Macro	FAM111B	0.449456	0.262	0.006	0	0
MKI67+ Macro	FANCI	0.449102	0.292	0.021	0	0
MKI67+ Macro	C20orf27	0.440543	0.597	0.303	6.11E-78	8.89E-74
MKI67+ Macro	DTL	0.438276	0.276	0.006	0	0
MKI67+ Macro	BUB1	0.437798	0.277	0.014	0	0
MKI67+ Macro	SRSF10	0.435224	0.657	0.377	1.67E-69	2.43E-65

MKI67+ Macro	LSM7	0.431933	0.788	0.493	3.40E-67	4.95E-63
MKI67+ Macro	TMEM14B	0.431839	0.812	0.533	1.88E-71	2.73E-67
MKI67+ Macro	CDCA5	0.429643	0.278	0.007	0	0
MKI67+ Macro	MTDH	0.426939	0.934	0.713	4.76E-55	6.93E-51
MKI67+ Macro	PXMP2	0.426576	0.33	0.051	2.36E-221	3.43E-217
MKI67+ Macro	CDC25B	0.423447	0.331	0.082	4.06E-132	5.91E-128
MKI67+ Macro	RPA1	0.42273	0.489	0.202	3.02E-88	4.39E-84
MKI67+ Macro	TECR	0.421032	0.681	0.414	2.00E-63	2.91E-59
MKI67+ Macro	HNRNPR	0.420219	0.73	0.432	1.51E-72	2.19E-68
MKI67+ Macro	VMA21	0.415833	0.805	0.526	9.24E-68	1.34E-63
MKI67+ Macro	PRR11	0.414493	0.28	0.042	3.96E-192	5.76E-188
MKI67+ Macro	CKAP2L	0.410708	0.232	0.002	0	0
MKI67+ Macro	ITGB7	0.409903	0.459	0.227	1.12E-55	1.62E-51
MKI67+ Macro	HINT2	0.408797	0.613	0.279	4.42E-96	6.43E-92
MKI67+ Macro	RFC3	0.406893	0.304	0.032	1.64E-285	2.39E-281
MKI67+ Macro	MANF	0.403113	0.738	0.464	1.14E-54	1.66E-50
MKI67+ Macro	BRCA2	0.402548	0.289	0.06	2.13E-140	3.11E-136
MKI67+ Macro	RAD23A	0.401761	0.752	0.497	1.08E-53	1.57E-49
MKI67+ Macro	SUPT16H	0.398297	0.501	0.253	4.95E-60	7.21E-56
MKI67+ Macro	KIF14	0.39657	0.197	0.005	0	0
MKI67+ Macro	ITGB1BP1	0.393653	0.618	0.319	5.62E-75	8.18E-71
MKI67+ Macro	TMED9	0.391952	0.774	0.56	5.38E-45	7.83E-41
MKI67+ Macro	NRM	0.384958	0.332	0.072	1.98E-151	2.89E-147
MKI67+ Macro	ATAD5	0.384444	0.258	0.029	6.93E-230	1.01E-225
MKI67+ Macro	LMNB2	0.382645	0.339	0.072	4.54E-160	6.60E-156
MKI67+ Macro	ILF3	0.380352	0.598	0.314	5.14E-68	7.47E-64
MKI67+ Macro	BUB1B	0.377497	0.247	0.005	0	0
MKI67+ Macro	BCL2L12	0.377005	0.375	0.103	1.43E-127	2.08E-123
MKI67+ Macro	XPO1	0.376518	0.609	0.347	6.04E-56	8.79E-52
MKI67+ Macro	TMEM97	0.374335	0.277	0.044	1.86E-178	2.71E-174
MKI67+ Macro	PLP2	0.374281	0.599	0.39	5.95E-37	8.65E-33

MKI67+ Macro	SPAG5	0.373807	0.214	0.012	4.88E-301	7.10E-297
MKI67+ Macro	CCT2	0.373373	0.639	0.363	1.36E-61	1.98E-57
MKI67+ Macro	TOPBP1	0.371585	0.369	0.114	1.04E-104	1.51E-100
MKI67+ Macro	CBX1	0.370968	0.45	0.185	2.26E-79	3.28E-75
MKI67+ Macro	GPAA1	0.367413	0.572	0.307	5.93E-61	8.63E-57
MKI67+ Macro	MCMBP	0.367207	0.45	0.21	8.18E-64	1.19E-59
MKI67+ Macro	KHDRBS1	0.365131	0.761	0.493	3.05E-56	4.44E-52
MKI67+ Macro	ACTL6A	0.364461	0.422	0.156	4.51E-93	6.57E-89
MKI67+ Macro	MARCKSL1	0.355782	0.474	0.24	1.63E-52	2.36E-48
MKI67+ Macro	MAZ	0.355579	0.5	0.244	2.04E-64	2.97E-60
MKI67+ Macro	SUZ12	0.355302	0.475	0.232	1.35E-58	1.97E-54
MKI67+ Macro	CMSS1	0.354758	0.378	0.117	1.53E-106	2.23E-102
MKI67+ Macro	FANCD2	0.354551	0.261	0.03	6.68E-224	9.72E-220
MKI67+ Macro	TEX30	0.354472	0.341	0.086	7.16E-128	1.04E-123
MKI67+ Macro	SNRNP40	0.35388	0.482	0.221	3.46E-71	5.03E-67
MKI67+ Macro	COX20	0.352453	0.699	0.422	4.10E-56	5.96E-52
MKI67+ Macro	MCM10	0.3497	0.211	0.002	0	0
MKI67+ Macro	IQGAP3	0.349266	0.194	0.025	5.71E-151	8.31E-147
MKI67+ Macro	ARPC5L	0.348951	0.559	0.256	1.77E-82	2.58E-78
MKI67+ Macro	ATP5A1	0.347412	0.841	0.62	4.15E-45	6.05E-41
MKI67+ Macro	C14orf80	0.345668	0.288	0.044	6.66E-192	9.69E-188
MKI67+ Macro	ICMT	0.345627	0.389	0.154	1.53E-75	2.22E-71
MKI67+ Macro	SUMO3	0.344928	0.857	0.629	2.98E-52	4.34E-48
MKI67+ Macro	NCAPD3	0.343446	0.28	0.059	2.42E-133	3.52E-129
MKI67+ Macro	GALM	0.343137	0.623	0.357	2.30E-53	3.35E-49
MKI67+ Macro	NUP210	0.342463	0.292	0.071	2.13E-116	3.09E-112
MKI67+ Macro	WEE1	0.340661	0.245	0.025	8.66E-231	1.26E-226
MKI67+ Macro	ST14	0.337477	0.605	0.353	1.95E-46	2.84E-42
MKI67+ Macro	CCT5	0.337345	0.711	0.473	4.09E-42	5.95E-38
MKI67+ Macro	CLEC11A	0.337222	0.468	0.286	1.70E-30	2.47E-26
MKI67+ Macro	NDC1	0.336587	0.301	0.065	1.88E-137	2.74E-133

MKI67+ Macro	LIG1	0.33548	0.271	0.037	8.47E-205	1.23E-200
MKI67+ Macro	RANGAP1	0.33481	0.334	0.105	1.93E-92	2.81E-88
MKI67+ Macro	RBBP4	0.33472	0.65	0.362	3.49E-62	5.08E-58
MKI67+ Macro	KIF4A	0.333845	0.205	0.004	0	0
MKI67+ Macro	NCAPG2	0.333502	0.245	0.021	1.34E-271	1.95E-267
MKI67+ Macro	TPM3	0.333476	0.955	0.805	1.15E-38	1.67E-34
MKI67+ Macro	RPP25	0.332	0.368	0.132	4.03E-84	5.87E-80
MKI67+ Macro	ELAVL1	0.331228	0.61	0.342	4.77E-56	6.94E-52
MKI67+ Macro	HDGF	0.331026	0.589	0.329	2.19E-52	3.19E-48
MKI67+ Macro	NETO2	0.328694	0.301	0.078	3.20E-107	4.66E-103
MKI67+ Macro	ASRGL1	0.327756	0.324	0.096	7.42E-98	1.08E-93
MKI67+ Macro	P4HB	0.327734	0.881	0.711	2.04E-36	2.96E-32
MKI67+ Macro	RER1	0.326247	0.794	0.575	2.33E-43	3.39E-39
MKI67+ Macro	STOML2	0.325982	0.59	0.327	5.54E-57	8.06E-53
MKI67+ Macro	ETFB	0.324806	0.766	0.491	2.86E-55	4.16E-51
MKI67+ Macro	MRPL41	0.32382	0.821	0.57	1.12E-53	1.64E-49
MKI67+ Macro	HJURP	0.323735	0.197	0.003	0	0
MKI67+ Macro	SLC1A5	0.320607	0.368	0.156	7.08E-60	1.03E-55
MKI67+ Macro	UNG	0.319928	0.226	0.048	6.69E-107	9.73E-103
MKI67+ Macro	RHNO1	0.319078	0.375	0.142	4.03E-74	5.86E-70
MKI67+ Macro	RPA2	0.318084	0.453	0.205	1.35E-67	1.97E-63
MKI67+ Macro	KDELR2	0.317076	0.882	0.666	3.70E-42	5.38E-38
MKI67+ Macro	CCT4	0.31593	0.76	0.522	4.93E-46	7.18E-42
MKI67+ Macro	POLD2	0.313677	0.38	0.132	2.92E-88	4.25E-84
MKI67+ Macro	RDX	0.312871	0.633	0.386	7.00E-44	1.02E-39
MKI67+ Macro	APEX1	0.310711	0.667	0.442	3.22E-42	4.69E-38
MKI67+ Macro	TUFM	0.310645	0.761	0.536	4.03E-40	5.86E-36
MKI67+ Macro	FBL	0.310104	0.551	0.293	1.65E-55	2.41E-51
MKI67+ Macro	C1orf35	0.309392	0.394	0.155	1.71E-72	2.49E-68
MKI67+ Macro	CDC6	0.309376	0.208	0.008	0	0
MKI67+ Macro	PSMD7	0.307654	0.77	0.544	4.79E-38	6.97E-34
MKI67+ Macro	CEP78	0.30751	0.258	0.062	3.80E-101	5.53E-97
MKI67+ Macro	DDX46	0.305449	0.671	0.401	1.18E-49	1.72E-45
MKI67+ Macro	RFC1	0.304431	0.426	0.194	1.72E-59	2.50E-55
MKI67+ Macro	SNRPA	0.30192	0.449	0.199	2.65E-67	3.86E-63
MKI67+ Macro	HS3ST1	0.299064	0.416	0.211	3.12E-42	4.54E-38
MKI67+ Macro	SPCS3	0.298842	0.884	0.656	1.09E-40	1.58E-36

MKI67+ Macro	POLD1	0.298191	0.294	0.073	8.57E-112	1.25E-107
MKI67+ Macro	TIMELESS	0.297095	0.251	0.034	4.23E-185	6.15E-181
MKI67+ Macro	SLC43A3	0.296505	0.66	0.445	6.24E-32	9.08E-28
MKI67+ Macro	REEP4	0.296345	0.404	0.189	7.81E-53	1.14E-48
MKI67+ Macro	DNAJC1	0.293348	0.503	0.267	2.41E-48	3.51E-44
MKI67+ Macro	IKBIP	0.292647	0.502	0.253	1.60E-57	2.33E-53
MKI67+ Macro	UQCRC1	0.291323	0.749	0.507	6.78E-40	9.87E-36
MKI67+ Macro	ATL3	0.289445	0.543	0.308	1.19E-45	1.74E-41
MKI67+ Macro	KIF20A	0.287708	0.153	0.002	0	0
MKI67+ Macro	EMC8	0.286576	0.427	0.201	8.22E-55	1.20E-50
MKI67+ Macro	RABL6	0.286488	0.501	0.251	6.50E-57	9.46E-53
MKI67+ Macro	EAF2	0.285498	0.374	0.138	1.22E-76	1.78E-72
MKI67+ Macro	NABP2	0.285456	0.329	0.121	3.06E-67	4.45E-63
MKI67+ Macro	C17orf89	0.284931	0.562	0.312	6.39E-49	9.29E-45
MKI67+ Macro	MSH6	0.283037	0.268	0.08	2.16E-78	3.14E-74
MKI67+ Macro	KIF15	0.282615	0.171	0.004	0	0
MKI67+ Macro	ARPC1A	0.281429	0.538	0.308	4.39E-47	6.39E-43
MKI67+ Macro	POLR2J	0.281292	0.7	0.457	2.76E-42	4.01E-38
MKI67+ Macro	RFWD3	0.279342	0.255	0.074	2.00E-76	2.91E-72
MKI67+ Macro	NUP50	0.278415	0.422	0.197	4.12E-55	6.00E-51
MKI67+ Macro	MRPL27	0.275886	0.676	0.411	2.47E-48	3.59E-44
MKI67+ Macro	CCDC18	0.274711	0.226	0.04	1.54E-129	2.23E-125
MKI67+ Macro	NUP107	0.274292	0.327	0.119	1.38E-67	2.01E-63
MKI67+ Macro	RBM17	0.274082	0.691	0.408	3.61E-54	5.25E-50
MKI67+ Macro	MRPS26	0.274002	0.44	0.203	1.83E-58	2.67E-54
MKI67+ Macro	PRADC1	0.273994	0.357	0.127	2.01E-78	2.93E-74
MKI67+ Macro	KDELC2	0.272827	0.243	0.064	5.57E-85	8.10E-81
MKI67+ Macro	MMS22L	0.271266	0.205	0.039	2.03E-108	2.96E-104
MKI67+ Macro	CLECL1	0.271077	0.332	0.151	1.58E-43	2.30E-39
MKI67+ Macro	CLPP	0.269835	0.523	0.288	3.91E-47	5.69E-43
MKI67+ Macro	SSNA1	0.26931	0.628	0.38	1.73E-45	2.51E-41
MKI67+ Macro	HMGXB4	0.269239	0.39	0.162	7.98E-62	1.16E-57
MKI67+ Macro	CCT7	0.268605	0.6	0.364	9.63E-43	1.40E-38
MKI67+ Macro	RBBP8	0.268422	0.278	0.078	7.09E-88	1.03E-83
MKI67+ Macro	NPM3	0.268101	0.383	0.163	2.79E-58	4.05E-54
MKI67+ Macro	PPP2R4	0.267326	0.463	0.244	9.92E-44	1.44E-39
MKI67+ Macro	GNB2	0.267169	0.801	0.588	9.74E-33	1.42E-28
MKI67+ Macro	HNRNPDL	0.264785	0.859	0.653	4.03E-33	5.87E-29

MKI67+ Macro	TTF2	0.264332	0.268	0.081	4.03E-76	5.86E-72
MKI67+ Macro	NDUFC2	0.264131	0.88	0.7	1.11E-32	1.62E-28
MKI67+ Macro	TP53	0.26409	0.41	0.186	7.76E-57	1.13E-52
MKI67+ Macro	CLNS1A	0.263955	0.632	0.4	4.54E-39	6.61E-35
MKI67+ Macro	RQCD1	0.263471	0.38	0.175	2.80E-49	4.08E-45
MKI67+ Macro	SEC11A	0.263188	0.882	0.682	1.04E-36	1.52E-32
MKI67+ Macro	CDCA7	0.261046	0.177	0.018	7.98E-167	1.16E-162
MKI67+ Macro	CDCA2	0.260115	0.163	0.003	0	0
MKI67+ Macro	RBL1	0.259907	0.204	0.039	8.72E-107	1.27E-102
MKI67+ Macro	CEP57	0.259797	0.378	0.168	7.78E-54	1.13E-49
MKI67+ Macro	BRIX1	0.258607	0.372	0.152	4.27E-63	6.22E-59
MKI67+ Macro	DAZAP1	0.256692	0.518	0.282	1.11E-46	1.61E-42
MKI67+ Macro	NFATC2IP	0.256531	0.372	0.184	5.45E-40	7.93E-36
MKI67+ Macro	ZNF367	0.256116	0.17	0.007	1.58E-275	2.29E-271
MKI67+ Macro	E2F2	0.254532	0.16	0.004	0	0
MKI67+ Macro	FAM207A	0.25413	0.467	0.241	6.70E-47	9.74E-43
MKI67+ Macro	ECT2	0.253521	0.187	0.042	2.49E-80	3.63E-76
MKI67+ Macro	TSPAN15	0.253383	0.392	0.19	1.97E-43	2.86E-39
MKI67+ Macro	MESDC2	0.252544	0.696	0.455	5.66E-38	8.23E-34
MKI67+ Macro	ALG5	0.250813	0.613	0.376	4.75E-40	6.92E-36
STMN1+ Macro	STMN1	2.874759	0.877	0.408	3.75E-76	5.46E-72
STMN1+ Macro	UBE2C	2.528574	0.377	0.057	3.16E-67	4.60E-63
STMN1+ Macro	HMGN2	2.263344	0.851	0.77	1.54E-43	2.24E-39
STMN1+ Macro	CKS1B	2.218519	0.519	0.227	9.26E-30	1.35E-25
STMN1+ Macro	TYMS	2.144681	0.416	0.072	2.91E-65	4.24E-61
STMN1+ Macro	RRM2	2.143258	0.338	0.045	1.80E-69	2.62E-65
STMN1+ Macro	TUBB	2.110786	0.909	0.768	1.03E-53	1.51E-49
STMN1+ Macro	PCNA	2.052229	0.519	0.277	1.41E-22	2.05E-18
STMN1+ Macro	CDK1	2.02567	0.325	0.069	3.37E-39	4.91E-35
STMN1+ Macro	TK1	1.943187	0.364	0.084	2.81E-40	4.10E-36
STMN1+ Macro	PTTG1	1.922604	0.331	0.139	7.95E-16	1.16E-11
STMN1+ Macro	TUBA1B	1.910262	0.968	0.874	1.37E-56	1.99E-52
STMN1+ Macro	HMGB1	1.837917	0.877	0.85	5.30E-42	7.72E-38
STMN1+ Macro	BIRC5	1.767075	0.318	0.059	3.71E-44	5.40E-40
STMN1+ Macro	DUT	1.69857	0.513	0.445	1.41E-09	2.05E-05
STMN1+ Macro	CENPM	1.672994	0.286	0.057	3.38E-36	4.91E-32
STMN1+ Macro	NUSAP1	1.671488	0.26	0.078	1.13E-19	1.64E-15
STMN1+ Macro	RANBP1	1.661775	0.584	0.535	7.77E-13	1.13E-08
STMN1+ Macro	ZWINT	1.643829	0.26	0.092	1.90E-15	2.76E-11

STMN1+ Macro	CDC20	1.567619	0.182	0.036	1.26E-22	1.83E-18
STMN1+ Macro	CDKN3	1.341555	0.214	0.061	1.17E-16	1.71E-12
STMN1+ Macro	MAD2L1	1.323869	0.221	0.068	1.51E-15	2.20E-11
STMN1+ Macro	RAN	1.316478	0.656	0.733	2.40E-11	3.49E-07
STMN1+ Macro	ANP32B	1.311372	0.636	0.607	1.84E-12	2.68E-08
STMN1+ Macro	PTMA	1.183535	1	0.983	1.46E-55	2.13E-51
STMN1+ Macro	CYCS	1.175644	0.636	0.724	7.37E-09	0.000107
STMN1+ Macro	TMEM106C	1.150239	0.26	0.121	8.03E-10	1.17E-05
STMN1+ Macro	CENPA	1.135286	0.143	0.028	5.27E-18	7.66E-14
STMN1+ Macro	CCNB2	1.02926	0.169	0.045	4.58E-14	6.67E-10
STMN1+ Macro	HMMR	1.029148	0.117	0.038	1.74E-07	0.002528
STMN1+ Macro	CCNA2	1.018416	0.11	0.034	1.04E-07	0.001507
STMN1+ Macro	CENPK	0.990731	0.169	0.059	7.09E-10	1.03E-05
STMN1+ Macro	CLSPN	0.988876	0.143	0.047	5.18E-09	7.54E-05
STMN1+ Macro	HNRNPA1	0.981458	0.708	0.814	1.43E-08	0.000209
STMN1+ Macro	SLC25A5	0.976596	0.708	0.794	6.37E-10	9.27E-06
STMN1+ Macro	YWHAH	0.975112	0.805	0.819	5.33E-15	7.76E-11
STMN1+ Macro	SNRPG	0.973147	0.636	0.75	2.99E-07	0.004354
STMN1+ Macro	ASF1B	0.967325	0.123	0.037	6.02E-09	8.76E-05
STMN1+ Macro	PPIA	0.955275	0.903	0.92	1.08E-22	1.56E-18
STMN1+ Macro	GINS2	0.913736	0.117	0.043	2.06E-06	0.029933
STMN1+ Macro	SUB1	0.907641	0.786	0.856	3.16E-12	4.60E-08
STMN1+ Macro	CHCHD2	0.90751	0.812	0.861	1.35E-17	1.96E-13
STMN1+ Macro	CALM2	0.903394	0.864	0.916	3.88E-17	5.65E-13
STMN1+ Macro	NPM1	0.898233	0.695	0.845	4.23E-07	0.00616
STMN1+ Macro	COX6C	0.891463	0.773	0.883	1.07E-12	1.55E-08
STMN1+ Macro	RPSA	0.886721	0.844	0.875	2.59E-18	3.77E-14
STMN1+ Macro	CDT1	0.877816	0.123	0.04	6.76E-08	0.000983
STMN1+ Macro	ARHGDIB	0.872688	0.805	0.871	8.19E-13	1.19E-08
STMN1+ Macro	AP2S1	0.857658	0.825	0.876	2.35E-16	3.43E-12
STMN1+ Macro	COTL1	0.822698	0.727	0.846	1.69E-06	0.024633
STMN1+ Macro	NDC80	0.814841	0.104	0.027	2.33E-09	3.39E-05
STMN1+ Macro	NCAPG	0.814445	0.117	0.025	2.08E-13	3.02E-09
STMN1+ Macro	UQCRQ	0.806802	0.669	0.829	2.05E-06	0.029796
STMN1+ Macro	LDHA	0.79601	0.721	0.809	9.43E-08	0.001372
STMN1+ Macro	ATP5L	0.793089	0.812	0.911	2.36E-13	3.44E-09
STMN1+ Macro	ATP5G3	0.774841	0.701	0.809	1.51E-07	0.002196
STMN1+ Macro	ARPC3	0.762366	0.864	0.922	1.10E-13	1.60E-09
STMN1+ Macro	PFN1	0.752308	0.942	0.971	7.62E-18	1.11E-13
STMN1+ Macro	HINT1	0.748883	0.76	0.87	3.46E-10	5.04E-06
STMN1+ Macro	AIF1	0.742762	0.942	0.958	4.85E-17	7.06E-13

STMN1+ Macro	RPLP0	0.721229	0.818	0.888	1.06E-11	1.54E-07
STMN1+ Macro	TPI1	0.709409	0.74	0.881	1.66E-06	0.024207
STMN1+ Macro	SUMO2	0.694829	0.708	0.876	6.55E-07	0.009537
STMN1+ Macro	GSTP1	0.683878	0.779	0.885	3.50E-09	5.09E-05
STMN1+ Macro	UQCR10	0.669875	0.708	0.851	3.75E-07	0.005461
STMN1+ Macro	COX6B1	0.650007	0.76	0.879	5.56E-09	8.10E-05
STMN1+ Macro	ACTB	0.628293	0.994	0.997	4.71E-21	6.85E-17
STMN1+ Macro	CFL1	0.591275	0.909	0.956	9.59E-12	1.40E-07
STMN1+ Macro	H3F3A	0.580907	0.916	0.955	4.80E-10	6.99E-06
STMN1+ Macro	ARPC2	0.579854	0.812	0.919	6.84E-11	9.95E-07
STMN1+ Macro	YBX1	0.560989	0.825	0.944	3.94E-07	0.005734
STMN1+ Macro	GAPDH	0.527713	0.948	0.97	7.11E-10	1.03E-05
STMN1+ Macro	VAMP8	0.526838	0.812	0.926	2.46E-06	0.035726
STMN1+ Macro	RPLP1	0.4125	0.981	0.993	1.01E-10	1.46E-06
STMN1+ Macro	RAB11B	0.388568	0.097	0.308	9.07E-07	0.0132
STMN1+ Macro	KDELR1	0.382679	0.188	0.508	2.02E-08	0.000294
STMN1+ Macro	NDUFS2	0.341788	0.175	0.447	1.49E-06	0.021676
STMN1+ Macro	PCBD1	0.337548	0.221	0.52	9.94E-07	0.014458
STMN1+ Macro	CDC37	0.297822	0.201	0.488	7.60E-07	0.01106
STMN1+ Macro	UBE2E1	0.273352	0.13	0.399	4.40E-08	0.00064
STMN1+ Macro	CCDC28A	0.271579	0.084	0.275	2.05E-06	0.029818
STMN1+ Macro	HSPA9	0.265447	0.201	0.477	8.59E-07	0.012504
STMN1+ Macro	TMEM60	0.264461	0.084	0.318	3.89E-08	0.000566
STMN1+ Macro	ECH1	0.259276	0.149	0.432	1.22E-08	0.000178
STMN1+ Macro	DYNC1LI1	0.25461	0.084	0.29	6.41E-07	0.009329
FTL+ Macro	C1orf56	0.795918	0.118	0.25	4.96E-29	7.21E-25
FTL+ Macro	FTL	0.759869	0.999	1	7.07E-153	1.03E-148
FTL+ Macro	PRDX1	0.528914	0.784	0.966	8.37E-27	1.22E-22
FTL+ Macro	UQCRB	0.478379	0.679	0.958	6.92E-09	0.000101
FTL+ Macro	PHGR1	0.474739	0.095	0.208	1.60E-27	2.33E-23
FTL+ Macro	PPDPF	0.472268	0.499	0.85	1.46E-08	0.000212
FTL+ Macro	LY96	0.464075	0.46	0.859	1.34E-22	1.96E-18
FTL+ Macro	TMSB10	0.453262	0.984	0.999	3.53E-96	5.13E-92
FTL+ Macro	SERF2	0.443596	0.907	0.994	2.54E-65	3.70E-61
FTL+ Macro	NDUFA1	0.42389	0.479	0.88	4.62E-17	6.72E-13
FTL+ Macro	PHKG1	0.422079	0.061	0.115	5.64E-12	8.20E-08
FTL+ Macro	ATP5E	0.406487	0.879	0.995	1.68E-43	2.44E-39
FTL+ Macro	RNF181	0.3862	0.303	0.733	1.72E-87	2.50E-83
FTL+ Macro	SH3BGRL	0.382965	0.417	0.845	5.81E-40	8.45E-36
FTL+ Macro	C4orf3	0.379707	0.457	0.872	2.21E-28	3.22E-24
FTL+ Macro	IFI27L2	0.376564	0.318	0.73	4.67E-75	6.80E-71

FTL+ Macro	LGALS1	0.369104	0.898	0.982	2.70E-27	3.93E-23
FTL+ Macro	S100A11	0.366944	0.919	0.993	1.76E-24	2.56E-20
FTL+ Macro	SEC61G	0.365534	0.51	0.902	1.02E-16	1.49E-12
FTL+ Macro	AGR2	0.360143	0.095	0.252	3.55E-46	5.16E-42
FTL+ Macro	FABP3	0.353094	0.091	0.206	1.23E-29	1.79E-25
FTL+ Macro	NDUFA13	0.336168	0.412	0.824	6.18E-39	8.99E-35
FTL+ Macro	SUMO1	0.335839	0.274	0.698	7.83E-102	1.14E-97
FTL+ Macro	TFF3	0.335286	0.128	0.327	3.40E-56	4.94E-52
FTL+ Macro	OAZ1	0.324703	0.864	0.992	2.39E-24	3.48E-20
FTL+ Macro	NEDD8	0.319613	0.424	0.849	6.16E-41	8.96E-37
FTL+ Macro	GABARAPL2	0.308155	0.274	0.711	1.64E-109	2.38E-105
FTL+ Macro	OTOA	0.308063	0.137	0.34	6.51E-56	9.47E-52
FTL+ Macro	LAMTOR2	0.302598	0.355	0.789	2.35E-72	3.42E-68
FTL+ Macro	PAIP2	0.292041	0.222	0.63	3.16E-123	4.60E-119
FTL+ Macro	PRDX5	0.291092	0.313	0.743	5.21E-88	7.59E-84
FTL+ Macro	BLOC1S2	0.285844	0.188	0.567	2.51E-126	3.66E-122
FTL+ Macro	CYSTM1	0.284784	0.216	0.585	1.89E-105	2.75E-101
FTL+ Macro	SDHB	0.279435	0.211	0.6	2.35E-119	3.42E-115
FTL+ Macro	KRT19	0.265513	0.047	0.128	1.17E-25	1.70E-21
FTL+ Macro	FAM96A	0.263787	0.253	0.668	3.13E-108	4.55E-104
FTL+ Macro	ALDOA	0.263467	0.105	0.324	1.21E-75	1.76E-71
FTL+ Macro	SAT1	0.256869	0.937	0.997	4.48E-07	0.006513
FTL+ Macro	HNMT	0.256211	0.328	0.777	6.17E-89	8.98E-85
FTL+ Macro	FAM162A	0.256024	0.15	0.472	1.04E-112	1.51E-108
