# Supplementary material for: Infiltration of Apoptotic M2 Macrophage Subpopulation Is Negatively Correlated with the Immunotherapy Response in Colorectal Cancer
Source: Int J Mol Sci. 2022 Sep 20;23(19):11014. doi: 10.3390/ijms231911014 (PMC9569653; doi:10.3390/ijms231911014)
Supplement: Supplementary file 1 [file ijms-23-11014-s001.zip › Supplementary Table S2.pdf]

Table S2. Macrophage-related signature gene sets

Description	Source	Gene sets
Antigen processing and presentation	https://doi.org/10.1016/j.cell.2018.05.060	CTSB,CREB1,PDIA3,HSPA5,B2M,RFX5,RFXAP,CTSS,NFYC,CD8A,CD4,KLRC3,KLRC1,KLRC2,KLRD1,RFXANK,IFI30,CALR,HSPA8,KIR3DL2,KIR3DL1,NFYB,IFNG,HSPA4,CANX,PSME3,HSPA2,LGMN,HSP90AA1,CTSL,PSME2,PSME1,CIITA,TAPBP,TAP1,TAP2,HSPA1A,HSPA1B,HSPA1L,TNF,HSP90AB1,NFYA,CD74,KLRC4,HLA-DOA,HLA-F,KIR2DL1,KIR2DL3,KIR2DS4,HLA-DQA1,HLA-DMB,HLA-DRA,HLA-DPB1,HSPA6,HLA-DMA,HLA-DPA1,HLA-G,HLA-A,HLA-DRB5,HLA-E,HLA-DOB,HLA-B,CD8B,HLA-C,KIR2DL4,KIR2DS1,KIR2DS3,KIR2DS5,HLA-DQB1,KIR2DL5A,KIR2DS2,KIR2DL2,HLA-DQA2,HLA-DRB1,KIR3DL3,HLA-DRB3,HLA-DRB4
Phagocytosis	https://doi.org/10.1016/j.cell.2018.05.060	RPS6KB1,RAC2,PRKCD,PRKCB,SYK,VASP,INPPL1,AKT1,GSN,INPP5D,PTPRC,PLA2G4A,AKT3,ARPC2,ARPC5,FCGR2B,VAV2,PLA2G4E,HCK,PLCG1,ARPC5L,PLA2G4B,PLA2G4D,PLA2G4F,PLD1,PIP5K1A,VAV3,PIK3CA,LYN,ASAP3,PIK3CD,PIK3R3,MARCKSL1,WASF2,CDC42,LIMK1,ARPC1B,ARPC3,NCF1,RAC1,ARPC1A,WASF3,RAF1,WASL,ARPC4,GAB2,AKT2,SPHK2,PAK1,LAT,MAPK3,PLCG2,PIK3R2,DNM2,MAP2K1,PIK3CB,WAS,WASF1,MARCKS,PIP5K1C,CRK,PRKCA,LIMK2,DOCK2,PLD2,SPHK1,ASAP2,SCIN,CFL2,ARF6,AMPH,PIK3R1,ASAP1,MYO10,PLA2G6,MAPK1,CRKL,PRKCE,VAV1,BIN1,PIP5K1B,RPS6KB2,CFL1,PRKCG,FCGR2A,FCGR1A,IGH,FCGR3A,PLPP1,PLPP3,PLPP2
Angiogenesis	https://doi.org/10.1016/j.cell.2018.05.060	PRG2,JAG2,COL5A2,COL3A1,VAV2,ITGAV,JAG1,THBD,POSTN,S100A4,PDGFA,LRPAP1,MSX1,PF4,SPP1,CCND2,OLR1,KCNJ8,PGLYRP1,NRP1,FGFR1,LPL,SLCO2A1,TIMP1,LUM,APOH,VTN,SERPINA5,VCAN,STC1,PTK2,APP,FSTL1,TNFRSF21,VEGFA,CXCL6
JAK-STAT signaling pathway	hsa04630	IL2,IL3,IL4,IL5,IL6,IL7,IL9,IL10,IL11,IL12A,IL12B,IL13,IL15,IL17D,IL19,IL20,IL21,IL22,IL23A,IL24,IFNA1,IFNA2,IFNA4,IFNA5,IFNA6,IFNA7,IFNA8,IFNA10,IFNA13,IFNA14,IFNA16,IFNA17,IFNA21,IFNB1,IFNG,IFNE,IFNK,IFNL1,IFNL2,IFNL3,IFNW1,OSM,LIF,TSLP,CTF1,CSF2,CNTF,CSF3,EPO,GH1,GH2,CSH1,CSH2,LEP,THPO,PRL,EGF,PDGFA,PDGFB,IL2RA,IL2RB,IL2RG,IL3RA,IL4R,IL5RA,IL6R,IL7R,IL9R,IL10RA,IL10RB,IL11RA,IL12RB1,IL12RB2,IL13RA1,IL13RA2,IL15RA,IL20RA,IL20RB,IL21R,IL22RA1,IL22RA2,IL23R,IL27RA,IL6ST,IFNAR1,IFNAR2,IFNGR1,IFNGR2,IFNLR1,OSMR,LIFR,CRLF2,CNTFR,CSF2RA,CSF2RB,CSF3R,EPOR,GHR,LEPR,MPL,PRLR,EGFR,PDGFRA,PDGFRB,JAK1,JAK2,JAK3,TYK2,STAT1,STAT2,STAT3,STAT4,STAT5A,STAT5B,STAT6,CISH,SOCS1,SOCS2,SOCS3,SOCS4,SOCS5,SOCS7,SOCS6,BCL2,MCL1,BCL2L1,PIM1,MYC,CCND1,CCND2,CCND3,CDKN1A,AOX1,GFAP,STAM2,STAM,PTPN2,PTPN6,IRF9,CREBBP,EP300,PIAS1,PI

		AS2,PIAS3,PIAS4,FHL1,PTPN11,GRB2,SOS1,SOS2,HRAS,RAF1,PIK3CA,PIK3CD,PIK3CB,PIK3R1,PIK3R2,PIK3R3,AKT1,AKT2,AKT3,MTOR
NF-kappaB signaling pathway	hsa04064	LCK,ZAP70,LAT,PLCG1,PRKCQ,IGH,SYK,LYN,BLNK,BTK,PLCG2,PRKCB,CARD10,CARD11,CARD14,BCL10,MALT1,IL1B,IL1R1,MYD88,IRAK1,IRAK4,TRAF6,TNF,TNFRSF1A,RIPK1,TRADD,TRAF2,TRAF5,BIRC2,BIRC3,EDA,EDAR,EDARADD,CYLD,EDA2R,DDX58,TRIM25,LBP,CD14,TLR4,LY96,TIRAP,TICAM2,TICAM1,CD40LG,CD40,TRAF3,TNFSF11,TNFRSF11A,LTA,LTB,TNFSF14,LTBR,MAP3K14,MAP3K7,TAB1,TAB2,TAB3,TNFSF13B,TNFRSF13C,IKBKG,CHUK,IKBKB,PARP1,PIAS4,UBE2I,ATM,PIDD1,ERC1,NFKBIA,NFKB1,RELA,CFLAR,XIAP,BCL2L1,BCL2,GADD45A,GADD45B,GADD45G,TRAF1,BCL2A1,NFKB2,CXCL8,TNFAIP3,PTGS2,CCL4,CCL4L2,CCL4L1,CXCL1,CXCL2,CXCL3,VCAM1,PLAU,CSNK2A1,CSNK2A2,CSNK2A3,CSNK2B,RELB,CCL13,CCL19,CCL21,CXCL12,ICAM1
PI3K-AKT signaling pathway	hsa04151	EGF,TGFA,EREG,AREG,FGF1,FGF2,FGF3,FGF4,FGF17,FGF6,FGF7,FGF8,FGF9,FGF10,FGF16,FGF5,FGF18,FGF20,FGF22,FGF19,FGF21,FGF23,NGF,BDNF,NTF3,NTF4,INS,IGF1,IGF2,PDGFA,PDGFB,PDGFC,PDGFD,CSF1,KITLG,FLT3LG,VEGFA,VEGFB,PGF,VEGFC,VEGFD,HGF,ANGPT1,ANGPT2,ANGPT4,EFNA1,EFNA2,EFNA3,EFNA4,EFNA5,EGFR,ERBB2,ERBB3,ERBB4,FGFR1,FGFR2,FGFR3,FGFR4,NGFR,NTRK1,NTRK2,INSR,IGF1R,PDGFRA,PDGFRB,CSF1R,KIT,FLT3,FLT1,FLT4,KDR,MET,TEK,EPHA2,GRB2,SOS1,SOS2,HRAS,KRAS,NRAS,RAF1,MAP2K1,MAP2K2,MAPK1,MAPK3,IRS1,TLR2,TLR4,RAC1,IGH,SYK,CD19,PIK3AP1,GH1,GH2,CSH1,CSH2,PRL,OSM,IL2,IL3,IL6,IL4,IL7,IFNA1,IFNA2,IFNA4,IFNA5,IFNA6,IFNA7,IFNA8,IFNA10,IFNA13,IFNA14,IFNA16,IFNA17,IFNA21,IFNB1,EPO,CSF3,GHR,PRLR,OSMR,IL2RA,IL2RB,IL2RG,IL3RA,IL6R,IL4R,IL7R,IFNAR1,IFNAR2,EPOR,CSF3R,JAK1,JAK2,JAK3,COL1A1,COL1A2,COL2A1,COL4A2,COL4A4,COL4A6,COL4A1,COL4A5,COL4A3,COL6A1,COL6A2,COL6A3,COL6A6,COL6A5,COL9A1,COL9A2,COL9A3,LAMA1,LAMA2,LAMA3,LAMA5,LAMA4,LAMB1,LAMB2,LAMB3,LAMB4,LAMC1,LAMC2,LAMC3,CHAD,RELN,THBS1,COMP,THBS2,THBS3,THBS4,FN1,SPP1,VTN,TNC,TNN,TNR,TNXB,VWF,IBSP,ITGA1,ITGA2,ITGA2B,ITGA3,ITGA4,ITGA5,ITGA6,ITGA7,ITGA8,ITGA9,ITGA10,ITGA11,ITGAV,ITGB1,ITGB3,ITGB4,ITGB5,ITGB6,ITGB7,ITGB8,PTK2,PIK3CA,PIK3CD,PIK3CB,PIK3R1,PIK3R2,PIK3R3,F2R,CHRM1,CHRM2,LPAR1,LPAR2,LPAR3,LPAR4,LPAR5,LPAR6,GNB1,GNB2,GNB3,GNB4,GNB5,GNG2,GNG3,GNG4,GNG5,GNG7,GNG8,GNG10,GNG11,GNG12,GNG13,GNGT1,GNGT2,PIK3CG,PIK3R5,PIK3R6,PDPK1,STK11,PRKAA1,PRKAA2,DDIT4,TSC1,TSC2,RHEB,MLST8,MTOR,RPTOR,EIF4EBP1,EIF4E,EIF4E2,EIF4E1B,RPS

		6KB1,RPS6KB2,EIF4B,RPS6,PRKCA,PKN1,PKN2,PKN3,SGK1,SGK2,SGK3,C8orf44-SGK3,AKT1,AKT2,AKT3,MAGI1,MAGI2,PTEN,THEM4,PPP2CA,PPP2CB,PPP2R1B,PPP2R1A,PPP2R2A,PPP2R2B,PPP2R2C,PPP2R2D,PPP2R3B,PPP2R3C,PPP2R3A,PPP2R5B,PPP2R5C,PPP2R5D,PPP2R5E,PPP2R5A,HSP90AA1,HSP90AB1,HSP90B1,CDC37,CRTC2,PHLPP1,PHLPP2,TCL1A,TCL1B,MTCP1,NOS3,BRCA1,GSK3B,GYS2,GYS1,PCK1,PCK2,G6PC1,G6PC2,G6PC3,MYC,CCND1,CDKN1A,CDKN1B,CDK2,CDK4,CDK6,CCND2,CCND3,CCNE1,CCNE2,FOXO3,RBL2,FASLG,BCL2L11,YWHAZ,YWHAB,YWHAQ,YWHAE,YWHAH,YWHAG,BAD,BCL2L1,BCL2,CASP9,CREB1,ATF2,ATF4,CREB3,CREB3L1,CREB3L2,CREB3L3,CREB3L4,CREB5,ATF6B,MCL1,RXRA,NR4A1,IKBKG,CHUK,IKBKB,RELA,NFKB1,MYB,MDM2,TP53
Amplify Inflammation	https://doi.org/10.1016/j.cell.2021.08.003	MMP12,MMP9,CCL18,IL7R,LMNA,MMP14,VMO1,PGD,FCGR2A,HMOX1,SLC39A8,TSC22D1,SPARC,ADAMDEC1,ATP6AP2,PEA15,CTSZ,TNS3,DSTN,PDPN,COL6A1,FPR3,TFRC,CTSK,COL6A2,ICAM1,C1orf54,NRP1,RAB10,IL2RG,NR1H3,ARID5B,MT1H,SLAMF8,SH3PXD2B,RCAN1,CCL5,PTRF,GSN,SPTAN1,RP3-412A9.11,ADIPOR1,MT1G,RRAS,SLC7A11,CALM1,PPIC,SPRED1,SEMA4A,ARL6IP5,DRAM1,TMEM50A,ACO1,CLIC4,HNMT,SPPL2A,TSPAN3,NQO1,DBI,HTATIP2,ECM1,SGPP1,IL13RA1,PDE4DIP,EPB41L3,SGTB,OLFML2B,RALA,RAP1B,PAPSS2,C1S,TSPAN15,PTAFR,DUSP3,LRP12,CD40,GCLM,GPX3,VIMP,SLC48A1,RAP1A,ACE,CREBL2,LILRB2,MMP1,NCK1,B4GALT1,MMP19,11-Sep,CALU,RP11-1008C21.1,SUCNR1,TFPI,IGF2R,CSAR2,ATP2C1,MMP10,CYP27B1,MFAP5,PRKCDBP,ASPH,STX12,BEX3,CLDND1,CPQ,BHLHE41,PDLIM4,STAU1,OAT,10-Sep,CYB5A,CRIM1,LXN,AK8,TNIK,PTPN12,TNNT1,MSMO1,SRC,LMO4,RND3,CNIH4,ITGB5,P4HA2,NNMT,TCEAL9,MYC,MSANTD3,AIFM2,ADI1,CDC42EP1,TMCO3,CHST2,MSN,FAM213B,FGD5,TXNRD1,SIGLEC15,LCP1,PIR,TTC39B,CYP2S1,TLR8,ST3GAL1,TWSG1,G3BP1,LSS,VAC14,RIT1,SERINC1
Resolve inflammation	https://doi.org/10.1016/j.cell.2021.08.003	APOE,APOC1,GPNMB,NUPR1,PLD3,ACP5,PSAP,CTSD,HEXB,GM2A,GRN,CYP27A1,KCNMA1,LIPA,LGMN,SCD,CXCL16,LILRB4,LRPAP1,SCPEP1,LAMP2,HEXA,HLA-A,DNASE2,VAT1,CPM,TIMP2,ACP2,FABP3,SDS,CD84,CD68,SMPDL3A,TSPAN4,TCN2,BCAP31,DPP7,SLC15A3,HSD17B14,ABCA1,NPL,C2,RARRES1,OTOA,KLHDC8B,ATP6AP1,MPP1,CADM1,SLC38A6,FMNL2,SMS,PEBP1,CHI3L1,CCPG1,APLP2,GUSB,GLMP,GAA,RBP1,NENF,ACOT13,HS3ST2,PILRA,RAB20,ABCG1,SCCPDH,WIPI1,APOC2,CHIT1,SOAT1,USF2,CD59,SLC29A1,MCOLN1,C9orf16,NAGLU,RENBP,ATP1B1,SLC1A3,RDX,SLC29A3,PLBD2,

		SCARB2,AVPI1,SCARB1,CDS2,PIK3IP1,AMDHD2,ANOS1,DDRGK1,NTAN1,TMEM37,CHCHD6,MFSD7,SLC35F6,PLEKHM2,CD72,AC079767.4,RAB42,DHRS3,CLIP4,TPRA1,RRAGD,EEPD1,TMEM140,APH1B,RP1-265C24.9,IQGAP2,MCFD2,EPHX1,TMED4,SLC26A11,PINK1,CECR1,ADCY3,BIRC7,SORBS3,SNX24,GLB1,PKD2L1,VEGFB,KLHL6,SMPD1,FXYD6,PIGT,SLC17A5,HAMP,OSCAR,SLC47A1,DNAJC5B,SERINC2,FARP1,KCNE1,GINM1,DENND2D,RP11-760N9.1,AAMDC,MMP2,PLA2G2D,DLEU7,TMEM255A,TBC1D2,PLPP3,FAM213A,KCNJ5,ALDH1A1,PHYH,GBA,SEPHS2,EPAS1
Lipid mediators	https://doi.org/10.1016/j.cell.2018.05.060	ADIPOQ,CD137,AgRP,ANGPT1,ANGPT2,ANGPTL3,ANGPTL4,ANGPTL6,BAFF,BMP4,CTSD,CTSS,CCL2,CCL5,RARRES2,CFD,CRP,CXCL8,CD26,ESM1,S100A12,AHSG,FGF1,FGF2,FGF21,FGF23,LGALS3,GCG,GH1,HGF,ICAM1,IGFBP1,IGFBP2,IGFBP3,IGFBP4,IGFBP6,IGFBP7,IL1b,IL6,IL10,IL11,INS,LEP,LIF,NGAL,MCSF,MIF,MPO,GHRL,GHSR,LIPE,LPL,AGT,CEBPA,CFD,FABP4,FASN,IRS2,KLF15,PPARG,PPARGC1A,RETN,SRT3,SLC2A4,SREBF1,ACACB,AXIN1,CCND1,CDK4,CEBPB,CEBPD,DKK1,E2F1,FABP4,FASN
M1 Macrophage Polarization	https://www.nature.com/articles/nri.2017.76	CCR7,IL2RA,IL15RA,IL7R,CXCL11,CCL19,CXCL10,CXCL9,TNF,CCL5,CCL15,IL12B,IL15,TRAIL,IL6,CCL20,PBEF1,PBEF1,BCL2A1,FAS,BIRC3,GADD45G,HSXIAPAF1,SLC7A5,SLC21A15,SLC2A6,SLC31A2,INDO,PLA1A,OASL,CHI3L2,HSD11B1,AK3,SPHK1,PFKFB3,PSME2,PFKP,PSMB9,PSMA2,OAS2,PTX3,CSPG2,APOL3,IGFBP4,APOL1,PDGFA,EDN1,APOL2,INHBA,APOL6,HESX1,IRF1,ATF3,IRF7
M2 Macrophage Polarization	https://www.nature.com/articles/nri.2017.76	GPR86,P2RY5,TGFBR2,HRH1,TLR5,DCL-1,MSR1,CXCR4,DECTIN1,P2RY14,DCSIGN,CLECSF13,MS4A6A,CD36,MS4A4A,MRC1,IGF1,CCL23,CCL18,CCL13,SLC21A9,SLC4A7,SLC38A6,CTSC,HEXB,LIPA,ADK,HNMT,TPST2,CERK,HS3ST2,LTA4H,CA2,ALOX15,HS3ST1,TGFBI,SEPP1,CHN2,FN1,FGL2,GAS7,EGR2,MAF
Hypoxia/HIF regulated	https://doi.org/10.1016/j.cell.2018.05.060	AL2,ALDOA,AM,BCL2,BCL2L1,FOS,JUN,SRC,CBP,CCR6,CEBPB,ENOLAS,FAS,FASLG,FKBP52,GAPDH,GLUT1,GLUT3,CSF2,IFNG,IFNB1,IL13,IL1R1,IL2RA,IL4,IL5,IL6R,IL8,IL10R,IL15R,ITGA6,ITK,JUN D,CLB54,MAP3K5,MAP2K1,MAP2K2,MIF,NFATC1,NFKB2,NFKBIE,P53,TGM6,TNF,TNFRSF1B,TRADD,TRAIL,TRAP1,VEGF
Glycolysis	https://doi.org/10.1016/j.cell.2021.08.003	C15orf48,LDHA,TPI1,GAPDH,MIF,ERO1A,CSTB,PGK1,BNIP3L,MT1X,C4orf3,BNIP3,LGALS1,VIM,P4HA1,SLAMF9,CLEC5A,ENO1,HK2,SDC2,PGAM1,FAM162A,FCGR2B,S100A10,MT1F,PKM,NDRG1,MT1E,PPDPF,GPI,VKORC1,BCAT1,H2AFY,ENO2,ALDOA,SEC61G,ADM,GBE1,FLT1,VDAC1,CD63,SLC2A1,RAB13,DARS,PLIN2,FCER1G,NT5E,AK4,QSOX1,PFKP,MXI1,GUK1,INSIG2,TNS1,

		RP11-841O20.2,TMEM45A,CA12,ANGPTL4,C1orf122,PDK1,ADAM8,MT1M,ZNF395,MARCO,TMEM38B,LAT,H1F0,ALDOC,RP4-614O4.5,PLOD2,UGP2,RP11-202P11.1,PDLIM7,MIR210HG,CLLU1OS,SCG5,PIK3CB,CD82,PPBP,NMB,WDR54,IGFBP2,SLC6A8,SPAG4,CD109,MRPS6,AGR2,EGLN1,CTB-138E5.1,WNT5A,CFDP1,RP11-798M19.6,FCRLB,PAM,ADSSL1,SLC2A5,SERPINE1,KCNN4,KIAA1147,MMP8,ZGLP1,GYS1,NOL3,CDK14,TRNAU1AP,FAH,DPCD
Recruit myeloid cells	https://doi.org/10.1016/j.cell.2021.08.003	CCL2,FABP5,FCGR3A,CCL7,RNASE1,RGCC,CTSL,MSR1,HCST,ALCAM,TUBA1C,ARHGAP18,C1orf162,PHLDA1,TREM2,ITGAM,TGFBI,TSPO,FBP1,CD9,SLC43A3,LPL,MFSD12,FN1,MATK,GLIPR2,SPP1,HTRA1,PLXDC2,CMTM3,ME2,HSD3B7,SELT,COLEC12,RP11-792D21.2,MGST3,ADAM9,TGM2,RHOC,GCHFR,ENG,CD151,PMP22,LRP1,15-Sep,SLC16A10,CAP1,CD276,LEPROT,EIF4E,C5AR1,ELOC,HAVCR2,SCP2,HCFC1R1,SLC11A1,ANXA4,PLAU,LSP1,LHFPL2,PPT1,CTSB,NPC1,IFI27L2,TMEM70,ADAM10,NEK6,MRC1,PAPSS1,ANXA11,SORT1,PDXK,REG1A,FXYD5,VSIG4,PPARG,ROMO1,FAM20C,EMC10,ARF6,CAPZA2,BCL2L1,BTF3L4,TPST2,HPCAL1,C19orf60,CCR1,NDUFB2,RTN4,TPD52L2,SPINK4,EMILIN2,CAB39,SH3GLB1,VTI1B,LPCAT2,DDAH2,HOMER3,IRAK1,TM4SF19,SLC39A11,MMP7,ZYX,MRAS,NDUFS5,GYPC,BSG,CSF1,PPM1M,NDUFA3,CD99,GLT25D1,LILRA6,MAPK13,HK3,SPOCD1,TNFRSF12A,ZNHIT1,P2RX4,ARPC4,REG1B,MYO1E,JAKMIP2,RP11-742B18.1,REG3A,TPM3,MRC1L1,SLC25A19,NCLN,NDUFB7,TPRG1,STX4,SNTB1,RP11-356N1.2,CERS2,PTPRO,NCEH1,MESDC1,NUS1,ABHD2,PLA2G15,TUBB2A,RASGRP3,RETN,RAPH1,PYGL,AGRP,NEDD8,FAM195A,AC147651.4
Stimulate growth	https://doi.org/10.1016/j.cell.2021.08.003	TIMP1,SERPINB9,VEGFA,SLC2A3,EREG,ATP1B3,THBD,CD300E,HBEGF,ATP13A3,CD44,NLRP3,ETS2,UPP1,CREM,YBX3,HIF1A,CD93,CYTOR,YWHAZ,SERPINB1,DSE,PFKFB3,ANPEP,TNFRSF1B,PLAUR,SLC44A1,PPP1CB,GK,SLC43A2,HPSE,MAP3K8,FAM49A,JARID2,BZW1,MAP3K20,LIMS1,AC068491.1,PNP,ADGRE2,METRNL,ADAM19,GABARAPL1,SYAP1,TPM4,PRNP,GNA15,TOP1,VDR,BACH1,RNF19B,INSIG1,STARD4,SNAI1,ITGAX,OLR1,MAP2K1,SLC25A37,BTG1,RASSF5,PICALM,XBP1,FLNA,VASP,ACTN1,GK5,CHD1,TLR2,EMP1,ACSL3,SKIL,ERGIC1,KYNU,STK24,RAB31,PDE4A,NFAT5,CHSY1,IL1RAP,MOB3B,ARFGAP3,B3GNT5,SEMA6B,CEBPB,SIPA1L1,SPHK1,TES,ELL2,SATB1,ITGA5,PPP1R3B,NEDD9,PID1,IL1R1,HNRNPK,ERRFI1,CCDC109B,EHD1,ACSL4,FAM102B,TRMT6,SSBP3,CKAP4,RHBDD2,FNDC3B,TLE3,E

		LK3,CAPN2,PURB,PFDN1,SFPQ,AKAP12,TAF13,PDE4D,TMEM158,CDC42EP2,PTPRE,ATP1A1,IL4R,C19orf59,AGO2
Immune activating alarmins	https://doi.org/10.1016/j.cell.2021.08.003	S100A8,S100A9,S100A12,FCN1,VCAN,CTSS,CSTA,S100A4,RP11-1143G9.4,MNDA,CD55,FPR1,LYZ,RGS2,GCA,CYBA,VMP1,STXBP2,TYROBP,CFD,CLEC4E,AIF1,RBP7,THBS1,CYBB,AP1S2,CD36,CDA,LTA4H,RNASE2,AGTRAP,METTL9,FAM45A,LAMTOR4,VNN2,CALM2,SELL,CYP1B1,H3F3A,PLBD1,ASGR1,RP6-159A1.4,BST1,LRRK2,GMFG,MGST1,RAB27A
Attract monocytes and neutrophils	https://doi.org/10.1016/j.cell.2021.08.003	CCL3,CCL4,CXCL3,CXCL2,CXCL8,CCL20,IL1B,PTGS2,SOD2,CXCL1,CCL3L1,IL1A,IL6,TNFAIP6,WTAP,CXCL5,NINJ1,TNF,RNF144B,INHBA,CCL3L3,TNIP3,EIF1B,TNFSF15,F3,IL23A,RIPK2,CSF3,GPR84,SERPINB2,CCL4L2,ITGB8,KB-1507C5.4,TNIP1,TRGC1,IRAK2,ZC3H12A,DNAAF1,ATP2B1,BTG3,TNFAIP8,MTF1,TRGC2,AZIN1,TM4SF1,CCL4L1,SAV1,PTX3,DLL1,SLAMF1,ACSL5,EDN1,HS3ST3B1,PLD1,IL24,STK26,RP11-37B2.1,RP11-701P16.5,HIVEP2,HEY1,GJB2,MIR3142HG,C2CD4B,RP6-99M1.2,KANK1,CTB-58E17.1,ELOVL7,ZP3,RP11-214O1.3,RP11-79H23.3,RP3-333H23.10,BBIP1,IL36G,POMZP3,LIF,RP11-955H22.1,MFSD2A,C7orf60,FAM124A,DUSP16,RP11-284N8.3,PTGES,RP11-362A1.1,VAMP4,KMO,CEMIP,CDKN2B,SLCO4A1,MET,CTA-293F17.1,CXCL6,AC061992.2,RP11-420G6.4,AC092580.4,CHIC2,CTB-58E17.3,RP11-214O1.2,PNPLA1,PLA2G2A,RP11-127L20.6,FERMT2,TFF2,ZC3H12C,KCNA3,RP5-885L7.10,AF124730.4,PDE6H,DNAH17,RP5-973M2.2,GSTM3,C2CD4A,RP11-378A13.2,DEFA5,AL590452.1,PPP2R5B,BAALC,DNER,ACOD1,NEU4,SLC9B2,C12orf61,RBFOX3,RP11-338I21.1,PLAGL2,LINC00677,RP11-367G18.1,SLC2A6,GXYLT2,AC018755.24,RP11-561B11.3,SEC14L2,LRRC32,AQP5,C9orf152,TRGV5,NPNT,RNVU1-20,RP11-631N16.4,TKTL1,CTD-2147F2.1,NCR1,AF064858.6,KLRC4,RP11-431K24.1,CTD-2371O3.3,GSTO2,AC002331.1,RP4-549L20.3,KLRC1,CTD-2521M24.8
Defense response to bacterium	GO:0042742	ADAM17,ADAMTS5,ADGRB1,ANG,ANKRD17,ANXA3,AQP1,ARG2,AZU1,BCL3,BPI,BPIFA1,BPIFA2,C10orf99,C5AR1,CAMP,CARD9,CCL20,CD160,CD36,CD4,CEBPB,CEBPE,CFP,CHGA,COCH,COLEC12,CRP,CST11,CTSG,CXCL13,CXCL6,CYBA,DCD,DEFA1,DEFA1B,DEFA3,DEFA4,DEFA5,DEFA6,DEFB1,DEFB103A,DEFB103B,DEFB104A,DEFB104B,DEFB105A,DEFB105B,DEFB106A,DEFB106B,DEFB108A,DEFB108B,DEFB109B,DEFB110,DEFB112,DE

		FB113,DEFB114,DEFB115,DEFB116,DEFB118,DEFB119,DEFB121,DEFB123,DEFB124,DEFB125,DEFB126,DEFB127,DEFB128,DEFB129,DEFB130A,DEFB130B,DEFB131A,DEFB131B,DEFB132,DEFB133,DEFB134,DEFB135,DEFB136,DEFB4A,DMBT1,DROSHA,ELANE,EPHA2,EPPIN,EPX,F2,F2RL1,FCER1G,FCN2,FGA,FGB,FGR,FOXP1,FPR2,GALP,GBP2,GBP4,GBP6,GBP7,GNLY,GRN,GSDMA,GSDMB,GSDMC,GSDMD,H2BC10,H2BC11,H2BC12,H2BC21,H2BC4,H2BC6,H2BC7,H2BC8,H2BS1,HAMP,HAVCR2,HLA-A,HLA-E,HMGB2,HP,HTN1,HTN3,IFNE,IGHA1,IGHA2,IGHD,IGHE,IGHG1,IGHG2,IGHG3,IGHG4,IGHM,IGHV1-18,IGHV1-24,IGHV1-3,IGHV1-45,IGHV1-58,IGHV1-69,IGHV1-69-2,IGHV1-69D,IGHV1OR15-1,IGHV2-26,IGHV2-5,IGHV2-70,IGHV2-70D,IGHV3-11,IGHV3-13,IGHV3-15,IGHV3-16,IGHV3-20,IGHV3-21,IGHV3-23,IGHV3-30,IGHV3-33,IGHV3-35,IGHV3-38,IGHV3-43,IGHV3-48,IGHV3-49,IGHV3-53,IGHV3-64,IGHV3-64D,IGHV3-66,IGHV3-7,IGHV3-72,IGHV3-73,IGHV3-74,IGHV4-28,IGHV4-31,IGHV4-34,IGHV4-39,IGHV4-4,IGHV4-59,IGHV4-61,IGHV5-10-1,IGHV5-51,IGHV6-1,IGHV7-4-1,IGHV7-81,IGKC,IGKV3-20,IGLC1,IGLC2,IGLC3,IGLC6,IGLC7,IGLL1,IGLL5,IL10,IL12A,IL12B,IL17A,IL17F,IL22RA1,IL23A,IL23R,IL27RA,IL6,IL6R,IL7R,IRF8,IRGM,ISG15,JCHAIN,KLK3,KLK5,KLK7,KRT6A,LACRT,LALBA,LBP,LCE3A,LCE3B,LCE3C,LCN2,LEAP2,LPO,LTA,LTF,LYG1,LYG2,LYPD8,LYST,LYZ,LYZL6,MAPKBP1,MAVS,MBL2,MICA,MIR140,MIR181B1,MIR223,MPEG1,MPO,MR1,MYD88,NAIP,NLRC4,NLRP1,NLRP10,NOD1,NOD2,NOS2,NOTCH2,NR1H4,OCIAD1,OCIAD2,OPTN,PGC,PGLYRP1,PGLYRP2,PGLYRP3,PGLYRP4,PI3,PLA2G1B,PLA2G2A,PLA2G6,PLAC8,PPBP,PPP1R11,PRB3,PRG2,PRKCD,PYCARD,RAB14,RAB1A,RAG2,RARRES2,RBPJ,REG3G,RIPK2,RNASE3,RNASE6,RNASE7,RNASE8,ROMO1,RPL30,RPL39,RPS19,S100A12,S100A14,S100A7,S100A8,S100A9,SEH1L,SELP,SEMG1,SEMG2,SERPINE1,SFTPD,SHC1,SIGLEC16,SIRT2,SLAMF8,SLC11A1,SLPI,SPAG11A,SPAG11B,SPINK5,SPN,SSC5D,STAB1,STAB2,STATH,SYK,SYT11,TBK1,TF,TIRAP,TLR2,TLR3,TLR4,TLR5,TLR6,TLR9,TMF1,TNF,TNFRSF14,TNFRSF1A,TNFSF8,TRAV27,TRBC1,TRBC2,TRDC,TREM2,TUSC2,UMOD,VGF,WFDC10A,WFDC10B,WFDC11,WFDC12,WFDC13,WFDC2,WFDC3,WFDC5,WFDC9,ZG16
Defense response to virus	https://genomemedicine.biomedcentral.com/articles/10.	ABCC9,ABCE1,ABCF3,ACOD1,ADAR,ADARB1,AGBL4,AGBL5,AICDA,AIM2,AIMP1,AKAP1,ANKRD17,APOBEC1,APOBEC3A,APOBEC3B,APOBEC3C,APOBEC3D,APOBEC3F,APOBEC3G,APOBEC3H,ATAD3A,ATG7,AZU1,BCL2,BCL2L1,BECN1,BIRC2,BIRC3,BNIP3,BNIP3L,BPIFA1,BST2,C1QBP,CARD9,CD207,CD40,CGAS,CLPB,CNOT7,CRCP,CXCL10,CXCL9,DDIT4,DDX1,DDX17,DD

	1186/s13073-021-00881-3	X21,DDX58,DDX60,DEFA1,DEFA1B,DEFA3,DHX36,DHX58,DHX9,DMBT1,DNAJC3,DTX3L,EIF2AK2,EIF2AK4,ELMOD2,EXOC1,EXOSC4,EXOSC5,F2RL1,FADD,FCN3,FGL2,FLNA,G3BP1,GBP1,GBP3,GPAM,GPATCH3,HERC5,HSP90AA1,HTRA1,HYAL2,IFI16,IFI27,IFI44L,IFI6,IFIH1,IFIT1,IFIT1B,IFIT2,IFIT3,IFIT5,IFITM1,IFITM2,IFITM3,IFNA1,IFNA10,IFNA13,IFNA14,IFNA16,IFNA17,IFNA2,IFNA21,IFNA4,IFNA5,IFNA6,IFNA7,IFNA8,IFNAR2,IFNB1,IFNE,IFNG,IFNK,IFNL1,IFNL2,IFNL3,IFNL4,IFNLR1,IFNW1,IL10RB,IL12B,IL12RB1,IL15,IL21,IL23A,IL23R,IL27,IL2RA,IL33,IL4,IL6,ILF3,ILRUN,IRF1,IRF2,IRF3,IRF5,IRF7,IRF9,ISG15,ISG20,ITCH,KCNJ8,LILRB1,LSM14A,LYST,MAP3K14,MAVS,MICA,MICB,MIR26B,MIR708,MLKL,MMP12,MOV10,MUL1,MX1,MX2,NCBP3,NLRC5,NLRP1,NLRP9,NLRX1,NOP53,NPLOC4,NT5C3A,OAS1,OAS2,OAS3,OASL,OPRK1,PARP9,PCBP2,PDE12,PHB,PHB2,PLA2G10,PLSCR1,PMAIP1,PML,POLR3A,POLR3B,POLR3C,POLR3D,POLR3E,POLR3F,POLR3G,POLR3H,POLR3K,PPM1B,PQBP1,PRF1,PTPN22,PTPRC,PUM1,PUM2,PYCARD,RELA,RIOK3,RIPK3,RNASE2,RNASE6,RNASEL,RNF125,RNF135,RNF216,RNF26,RSAD2,RTP4,SAMHD1,SEC14L1,SELENOK,SERINC3,SERINC5,SETD2,SHFL,SIN3A,SKP2,SLFN11,SLFN13,STAT1,STAT2,STING1,TARBP2,TBK1,TICAM1,TKFC,TLR3,TLR7,TLR8,TLR9,TNFAIP3,TOMM70,TRAF3,TRAF3IP1,TRAF3IP2,TREX1,TRIM11,TRIM15,TRIM22,TRIM34,TRIM44,TRIM5,TRIM52,TRIM56,TRIM6,TSPAN32,TSPAN6,TTC4,UFD1,UNC13D,UNC93B1,USP15,USP17L2,VAMP8,ZBP1,ZC3H12A,ZC3HAV1,ZCCHC3,ZDHHC1,ZDHHC11,ZMPSTE24,ZMYND11,ZNF175
Positive regulation of cytokine production	https://genomemedicine.biomedcentral.com/articles/10.1186/s13073-021-00881-3	ABL1,ADAM17,ADAM8,ADCYAP1,ADIPOQ,ADRA2A,AFAP1L2,AGER,AGPAT1,AGPAT2,AGT,AIF1,AIM2,AKIRIN2,ALOX15B,ANXA1,APOA2,APP,APPL1,ARFGEF2,ARHGEF2,ARNT,ATF2,ATF4,ATP6AP2,AZU1,B2M,BCL10,BCL3,BRCA1,C1QTNF3,C1QTNF4,C3,C3AR1,C5,C5AR1,CADM1,CARD11,CARD8,CARD9,CASP1,CASP8,CCBE1,CCDC88B,CCL1,CCL19,CCL3,CCR2,CCR7,CD14,CD160,CD2,CD200,CD226,CD244,CD274,CD276,CD28,CD34,CD36,CD3E,CD4,CD40,CD40LG,CD46,CD58,CD6,CD74,CD80,CD81,CD83,CD86,CEBPB,CEBPG,CGAS,CHIA,CHUK,CLEC6A,CLEC7A,CLEC9A,CLNK,CLU,CRCP,CREB1,CREBBP,CRLF2,CRTAM,CSF1R,CSF2,CTNNB1,CXCL17,CYBA,CYP1B1,CYRIB,DDIT3,DDT,DDX21,DDX3X,DDX41,DDX58,DEFB124,DHX33,DHX36,DHX58,DHX9,DRD2,EBI3,EGR1,EIF2AK2,EIF2AK3,ELANE,EP300,EPX,EREG,F2R,F2RL1,F3,FADD,FCER1G,FCN1,FERMT1,FFAR2,FFAR3,FGR,FLOT1,FLT4,FOXP3,FRMD8,FURIN,FZD5,GAPDH,GATA3,GATA4,GATA6,GBP5,GDF2,GLMN,GPRC5B,GPSM3,GSDMD,H19,HAVCR2,HDAC2,HEG1,HGF,HHLA2,HIF1A,HILPDA,HK1,HLA-A,HLA-DPA1,HLA-DPB1,HLA-E,HLA-F,HLA-

		G,HMGB1,HMGB2,HMHB1,HMOX1,HPSE,HRAS,HSP90AA1,HSPA1A,HSPA1B,HSPB1,HSPD1,HTR2B,HYAL2,IDO1,IFI16,IFIH1,IFNG,IFNGR1,IFNL1,IGHD,IL10,IL12A,IL12B,IL12RB1,IL12RB2,IL13,IL15,IL16,IL17A,IL17B,IL17D,IL17F,IL17RA,IL17RC,IL18,IL18R1,IL1A,IL1B,IL1R1,IL1RAP,IL1RL1,IL1RL2,IL2,IL20RB,IL21,IL23A,IL23R,IL26,IL27,IL27RA,IL33,IL4,IL4R,IL6,IL6R,IL6ST,IL7,IL9,INAVA,INS,IRAK1,IRF1,IRF3,IRF4,IRF5,IRF7,IRF8,ISL1,JAK2,KIR2DL4,KPNA6,LAMTOR5,LAPTM5,LBP,LEP,LGALS9,LILRA2,LILRA5,LILRB1,LILRB2,LPL,LRP1,LRRFIP1,LTA,LTB,LUM,LURAP1,LY9,LY96,MALT1,MAP3K7,MAPK11,MAPK13,MAPK14,MAPKAPK2,MAVS,MBP,MCOLN2,MDK,MIF,MIR132,MIR144,MIR145,MIR149,MIR17,MIR182,MIR206,MIR21,MIR27B,MIR657,MIR675,MIR92A1,MMP12,MMP8,MNDA,MRE11,MYB,MYD88,NFAM1,NFATC4,NFKB1,NFKB2,NLRC4,NLRP1,NLRP10,NLRP12,NLRP2,NLRP3,NLRP9,NOD1,NOD2,NODAL,NOX1,NR4A3,ORM1,ORM2,OSM,P2RX7,PAEP,PANX1,PANX2,PANX3,PARK7,PDE4B,PDE4D,PELI1,PF4,PIBF1,PIK3R1,PLA2G1B,PLA2G3,PLCB1,PLCG2,POLR1C,POLR2E,POLR2F,POLR2H,POLR2K,POLR2L,POLR3A,POLR3B,POLR3C,POLR3D,POLR3E,POLR3F,POLR3G,POLR3GL,POLR3H,POLR3K,POSTN,POU2AF1,POU2F2,PQBP1,PRG3,PRKCQ,PRKCZ,PRKD2,PRKDC,PSEN1,PTAFR,PTGER4,PTGS2,PTPN11,PTPN22,PTPRC,PTPRJ,PYCARD,PYDC1,PYHIN1,RAB7B,RAET1G,RARA,RASGRP1,RELA,RGCC,RIOK3,RIPK1,RIPK2,RNF135,ROCK2,RORA,RPS3,RSAD2,RUNX1,SAA1,SASH3,SCAMP5,SCIMP,SCRIB,SELENOK,SEMA7A,SERPINB7,SERPINE1,SERPINF2,SETD2,SIGLEC16,SLAMF6,SLC11A1,SLC7A5,SMAD3,SOD1,SORL1,SPHK1,SPN,SPTBN1,STAT1,STAT3,STAT5B,STAT6,STING1,STMP1,SULF1,SULF2,SYK,TBK1,TGFB1,THBS1,TICAM1,TIGIT,TIRAP,TLR1,TLR2,TLR3,TLR4,TLR5,TLR6,TLR7,TLR8,TLR9,TMF1,TMIGD2,TNF,TNFRSF14,TNFRSF8,TNFSF4,TOMM70,TRAF2,TRAF6,TREM2,TRIM15,TRIM16,TRIM32,TRIM6,TSLP,TUSC2,TWIST1,TXK,TYROBP,UCN,USP50,WNT11,WNT3A,WNT5A,XBP1,XCL1,XRCC5,XRCC6,ZBP1,ZCCHC3,ZFPM1,ZNF580,ZP3
Response to interferon gamma	https://genomemedicine.biomedcentral.com/articles/10.1186/s13073-021-00881-3	ACOD1,ACTG1,ACTR2,ACTR3,ADAMTS13,AIF1,AQP4,ARG1,ASS1,BST2,CALCOCO2,CASP1,CCL1,CCL11,CCL13,CCL14,CCL15,CCL16,CCL17,CCL18,CCL19,CCL2,CCL20,CCL21,CCL22,CCL23,CCL24,CCL25,CCL26,CCL3,CCL3L1,CCL3L3,CCL4,CCL5,CCL7,CCL8,CD40,CD47,CD58,CD74,CDC37,CDC42,CDC42EP2,CDC42EP4,CIITA,CITED1,CLDN1,CX3CL1,CXCL16,CYP27B1,DAPK1,DAPK3,EDN1,EPRS1,FASLG,FLNB,GAPDH,GBP1,GBP2,GBP3,GBP4,GBP5,GBP6,GBP7,GCH1,GSN,HCK,HLA-DPA1,HPX,HSP90AB1,IFITM1,IFITM2,IFITM3,IFNG,IL12B,IL12RB1,IL23R,IRF1,IRF8,IRGM,JAK1,JAK2,KIF5B,KYNU,LGALS9,MED1,MEFV,MRC1,MYC,MYO1C,NLRC5,NMI,NOS2,NR1H2,NR1

		H3,NUB1,OTOP1,PARP14,PARP9,PDE12,PPARG,PTPN2,RAB20,RAB43,RAB7B,RPL13A,RPS6KB1,SHFL,SIRPA,SLC11A1,SLC26A6,SLC30A8,SNCA,SOCS1,SP100,STAT1,STX4,STX8,STXBP1,STXBP3,STXBP4,SYNCRIP,TDGF1,TLR2,TLR3,TLR4,TNF,TP53,TRIM21,TXK,UBD,VAMP3,VIM,VPS26B,WAS,WNT5A,XCL1,XCL2,ZYX
Response to tumor necrosis factor	https://genomemedicine.biomedcentral.com/articles/10.1186/s13073-021-00881-3	ACOD1,ACTN4,ADAM10,ADAM17,ADAM9,ADAMTS12,ADAMTS13,ADAMTS7,ADIPOQ,AFF3,AIM2,AKAP12,AKT1,ANKRD1,APOA1,APOB,ARHGEF2,ASAH1,ASS1,BAG4,BIRC2,BIRC3,BIRC7,BRCA1,CACTIN,CALCA,CAMP,CARD14,CARD16,CARD8,CASP1,CASP3,CASP4,CASP8,CCDC3,CCL1,CCL11,CCL13,CCL14,CCL15,CCL16,CCL17,CCL18,CCL19,CCL2,CCL20,CCL21,CCL22,CCL23,CCL24,CCL25,CCL26,CCL3,CCL3L1,CCL3L3,CCL4,CCL5,CCL7,CCL8,CD14,CD40,CD58,CD70,CDIP1,CEBPA,CHI3L1,CHUK,CIB1,CLDN1,CLDN18,COL1A1,COMMD7,CPNE1,CRHBP,CX3CL1,CXCL16,CXCL8,CYBA,CYLD,CYP1B1,DAB2IP,DCSTAMP,DHX9,EDA2R,EDN1,EIF5A,ENDOG,ERBIN,EXT1,F2RL1,FABP4,FAS,FOXO3,GAS6,GATA3,GBA,GBP1,GBP2,GBP3,GCH1,GFER,GPD1,GPER1,GPS2,GSDME,GSTP1,H2BC11,HAMP,HAS2,HES1,HIPK1,HMHB1,HSPA1A,HSPA1B,HYAL1,HYAL2,HYAL3,IGBP1,IKBKB,IL18BP,ILK,INPP5K,JAK2,KAT2A,KLF2,KRT18,LAPTM5,LIMS1,MAP2K7,MAP3K5,MAP4K3,MAPK1,MAPK14,MAPK3,MBP,MIR1246,MIR142,MIR152,MIR181B1,MIR20B,MIR24-1,MIR27B,MIR30C2,MIR31,MIR34A,MIR766,MYOD1,MYOG,NAIP,NFE2L2,NFKB1,NFKBIA,NKIRAS1,NKIRAS2,NKX3-1,NLRP2B,NOL3,NPNT,NR1D1,NR1H4,NUB1,OCSTAMP,OTULIN,PCK1,PCK2,PELI3,PIAS3,PIAS4,PID1,PLVAP,PPP2CB,PRKN,PRPF8,PTGS2,PTK2B,PTPN2,PYCARD,PYDC1,PYDC2,RELA,RFFL,RIPK1,RORA,RPS3,RPS6KB1,RRAGA,SELE,SFRP1,SHARPIN,SIRT1,SLC2A4,SMPD1,SMPD3,SMPD4,SPATA2,SPHK1,SPPL2A,SPPL2B,ST18,STAT1,SYK,TANK,TCL1A,TDGF1,THBS1,TMSB4X,TNF,TNFAIP3,TNFRSF11A,TNFRSF13C,TNFRSF14,TNFRSF17,TNFRSF18,TNFRSF19,TNFRSF1A,TNFRSF1B,TNFRSF21,TNFRSF25,TNFRSF4,TNFSF11,TNFSF13B,TNFSF18,TP53,TRADD,TRAF1,TRAF2,TRAF3,TRAF3IP2,TRAF5,TRAF6,TRAIP,TRIM32,TRPV1,TXNDC17,UBD,UBE2K,VCAM1,XCL1,XCL2,XIAP,YBX3,YTHDC2,ZC3H12A,ZFAND6,ZFP36,ZFP36L1,ZFP36L2,ZNF675
Regulation of innate immune response	https://genomemedicine.biomedcentral.com/articles/10.1186/s13	A2M,ABCE1,ACOD1,ADAM8,ADAR,AIM2,AKIRIN2,AP1G1,APOE,APPL1,APPL2,ARG1,ARRB2,BCL10,BIRC2,BIRC3,BTRC,CACTIN,CADM1,CARD11,CARD9,CCL5,CD160,CD1D,CD209,CD226,CD96,CDC37,CEACAM1,CGAS,CHUK,CLEC10A,CLEC12B,CLEC4A,CLEC4C,CLEC4D,CLEC4E,CLEC6A,CLNK,CNOT7,COCH,CR1,CREBBP,CRK,CRTAM,CUL1,DCST1,DHX58,DHX9,DRD2,DUSP10,EP300,ERAP1,EREG,FADD,FAM3A,FBXW11,FCER1G,FCGR2B,

	073-021-00881-3	FCN1,FFAR2,FGR,FPR2,FYN,GBP5,GRN,HAVCR2,HCK,HEXIM1,HLA-A,HLA-B,HLA-E,HLA-F,HLA-G,HMGB1,HMGB2,HPX,HRAS,HSP90AA1,HSP90AB1,ICAM2,ICAM3,IFI16,IFI35,IFNAR2,IFNB1,IFNG,IFNGR1,IFNGR2,IFNK,IKBKB,IKBKE,IKBKG,IL12A,IL12B,IL18RAP,IL21,INS,IRAK3,IRF1,IRF3,IRF7,IRGM,JAK1,JAK2,KIR2DL4,KIR2DS2,KLRC1,KLRC2,KLRC4-KLRK1,KLRD1,KLRK1,KRAS,LAG3,LAMP1,LBP,LEP,LGALS9,LILRA2,LILRB1,LRP8,LSM14A,LYAR,LYN,MALT1,MAP3K7,MATR3,MAVS,MED1,METTL3,MICA,MIR181B1,MIR21,MIR520B,MIR520E,MMP12,MNDA,MUC1,MUC12,MUC13,MUC15,MUC16,MUC17,MUC19,MUC2,MUC20,MUC21,MUC3A,MUC4,MUC5AC,MUC5B,MUC6,MUC7,MUCL1,MUL1,NCR1,NCR3,NECTIN2,NFKB1,NLRC3,NLRC4,NLRC5,NLRX1,NMI,NOD2,NONO,NR1H2,NR1H3,NRAS,OTOP1,PAK1,PAK2,PAK3,PARP14,PARP9,PDPK1,PIAS1,PIK3R6,PLA2G5,PLCG2,PLSCR1,POLR3B,POLR3C,POLR3D,POLR3F,POLR3G,PPARG,PQBP1,PRKACA,PRKACB,PRKACG,PRKCD,PRKDC,PSMA1,PSMA2,PSMA3,PSMA4,PSMA5,PSMA6,PSMA7,PSMA8,PSMB1,PSMB10,PSMB11,PSMB2,PSMB3,PSMB4,PSMB5,PSMB6,PSMB7,PSMB8,PSMB9,PSMC1,PSMC2,PSMC3,PSMC4,PSMC5,PSMC6,PSMD1,PSMD10,PSMD11,PSMD12,PSMD13,PSMD14,PSMD2,PSMD3,PSMD4,PSMD5,PSMD6,PSMD7,PSMD8,PSMD9,PSME1,PSME2,PSME3,PSME4,PSMF1,PSPC1,PTPN1,PTPN11,PTPN2,PTPN22,PTPN6,PVR,PYCARD,PYHIN1,RAET1E,RAET1G,RAF1,RASGRP1,RBM14,RELA,RELB,RIOK3,RNASEL,RNF135,RPS19,RPS6KA5,SAMHD1,SERPINB4,SERPINB9,SERPING1,SFPQ,SH2D1A,SH2D1B,SIN3A,SKP1,SLAMF6,SLAMF8,SLC15A4,SOCS1,SOCS3,SRC,STAT1,STAT5B,STING1,SUMO1,SUSD4,SYK,TAB1,TAB2,TAB3,TASL,TBK1,TIRAP,TKFC,TLR4,TLR8,TNFAIP3,TOMM70,TRAF6,TRAFD1,TREM2,TREX1,TRIM21,TRIM5,TRIM6,TTLL12,TXK,TYRO3,TYROBP,UBE2K,USP18,VAV1,VSIG4,WNT5A,XIAP,XRCC5,XRCC6,YTHDF2,YTHDF3,ZBP1,ZCCHC3
Oxidative phosphorylation	https://doi.org/10.1016/j.cell.2020.03.048	ABCD1,ACTN3,AFG1L,AK4,ANTKMT,ATP5F1A,ATP5F1B,ATP5F1C,ATP5F1D,ATP5F1E,ATP5MC1,ATP5MC2,ATP5MC3,ATP5ME,ATP5MF,ATP5MG,ATP5PB,ATP5PD,ATP5PF,ATP5PO,ATP7A,ATPSCKMT,BID,CCNB1,CDK1,CHCHD10,CHCHD2,COA6,COQ9,COX10,COX15,COX4I1,COX4I2,COX5A,COX5B,COX6A1,COX6A2,COX6B1,COX6B2,COX6C,COX7A1,COX7A2,COX7A2L,COX7A2P2,COX7B,COX7B2,COX7C,COX8A,COX8C,CYC1,CYCS,DGUOK,DLD,DMAC2L,DNAJC15,DNAJC30,FXN,GHITM,ISCU,MIR210,MLXIPL,MSH2,MT-ATP6,MT-ATP8,MT-CO1,MT-CO2,MT-CO3,MT-CYB,MT-ND1,MT-ND2,MT-ND3,MT-ND4,MT-ND4L,MT-ND5,MT-ND6,MTCO2P12,MYOG,NDUFA1,NDUFA10,NDUFA11,NDUFA12,NDUFA13,NDUFA2,NDUFA3,NDUFA4,NDUFA5,NDUFA6,NDUF

		A7,NDUFA8,NDUFA9,NDUFAB1,NDUFAF1,NDUFB1,NDUFB10,NDUFB11,NDUFB2,NDUFB3,NDUFB4,NDUFB5,NDUFB6,NDUFB7,NDUFB8,NDUFB9,NDUFC1,NDUFC2,NDUFC2-KCTD14,NDUFS1,NDUFS2,NDUFS3,NDUFS4,NDUFS5,NDUFS6,NDUFS7,NDUFS8,NDUFV1,NDUFV2,NDUFV3,NIPSNAP2,NUPR1,PARK7,PDE12,PINK1,PPIF,SDHA,SDHAF2,SDHC,SDHD,SHMT2,SLC25A23,SLC25A33,SNCA,STOML2,SURF1,TAZ,TEFM,UQCC2,UQCC3,UQCR10,UQCR11,UQCRB,UQCRC1,UQCRC2,UQCRFS1,UQCRFS1P1,UQCRH,UQCRHL,UQCRQ,VCP
Negative regulation of macrophage activation	GO:0043031	FCGR2B,CD200,ADGRF5,NR1H3,LRFN5,VSIG4,IL31RA,BPI,IL4,ZC3H12A
Positive regulation of macrophage activation	GO:0043032	LBP,KARS1,WNT5A,HAVCR2,SPACA3,IL4R,IL13,IL1RL1,CEBPA,HAMP,THBS1,IL10,TNIP2,IL33,TLR6,HSPD1,JUND,PLA2G4A,TLR4
Macrophage differentiation	GO:0030225	ADIPOQ,APP,BMP4,C1QC,CASP8,CD4,CDC42,CEBPA,CEBPE,CSF1,CSF1R,CSF2,EIF2AK1,FADD,GAB3,GATA2,GBA,HCLS1,HLA-DRB1,HSF1,ID2,IFNG,IL15,IL31RA,IL34,INHA,INHBA,L3MBTL3,LIF,MIR145,MIR223,MMP9,NKX2-3,NRROS,PARP1,PF4,PRKCA,PTPN2,RB1,RIPK1,ROR2,SIRT1,SPI1,TGFB1,TLR2,TRIB1,TSPAN2,VEGFA,ZBTB46
Macrophage apoptotic process	GO:0071888	CDKN2A,CTSL,GHSR,IRF3,IRF7,MEF2C,NOD2,PLEKHO2,SELENOS,SIRT1,TCP1
Negative regulation of epithelial to mesenchymal transition	GO:0010719	ADIPOR1,BMP5,DAB2IP,DACT3,EFNA1,EPHA4,FOXA1,FOXA2,FUZ,GATA3,HPN,IL17RD,LDLRAD4,MAD2L2,MARK1,MIR144,MIR149,MIR29B1,MIR573,MIR590,NKX2-1,NOG,OVOL2,PPP2CA,PTEN,SDHAF2,SFRP1,SFRP2,SMAD7,SPRED1,SPRED2,SPRED3,SPRY1,SPRY2,TBX5,TGFB2,TRIM62,USF3,VASN,ZNF750
Positive regulation of epithelial to mesenchymal transition	GO:0010718	ACVR1,ALX1,AXIN2,BAMBI,BCL9L,BMP2,BMP4,BMP7,COL1A1,CRB2,CTNNB1,DAB2,ENG,EZH2,FERMT2,FOXC1,GCNT2,GLIPR2,HDAC2,IL1B,IL6,ISL1,JAG1,LEF1,LOXL2,MDK,MIR21,MIR221,MIR222,MIR519D,MTOR,NOTCH1,OLFM1,PDPN,RGCC,SDCBP,SERPINB3,SMAD2,SMAD3,SMAD4,SNAI1,TBX20,TCF7L2,TGFB1,TGFB1I1,TGFB2,TGFB3,TGFBR1,TGFBR2,TIAM1,TWIST1,WWTR1,ZNF703
Colony stimulating factor production	GO:0036301	CD34,FOXP1,HAVCR2,ISL1,LILRA2,MIR128-1,TSLP
Macrophage migration	GO:1905517	AKIRIN1,AZU1,C3AR1,C5,C5AR1,CCL2,CCL3,CCL5,CCR2,CD200,CD200R1,CD81,CD9,CKLF,CMKLR1,CSF1,CSF1R,CX3CL1,CX3CR1,CXCL17,CYP19A1,DDT,EDN2,EDNRB,EMILIN1,IL34,LGALS3,MAPK1,MAPK3,MCOLN2,MDK,MIF,MIR128-1,MIR24-1,MMP14,MMP28,MSTN,MTUS1,NUP85,P2RX4,P2RY12,PTK2,PTK2B,RARRES2,ROR2,SAA1,SFTPD,SLAMF1,SLAMF8,STAP1,TAFA4,THBS1,TREM2,TRIM55,TRPV4
