# Supplementary material for: Infiltration of Apoptotic M2 Macrophage Subpopulation Is Negatively Correlated with the Immunotherapy Response in Colorectal Cancer
Source: Int J Mol Sci. 2022 Sep 20;23(19):11014. doi: 10.3390/ijms231911014 (PMC9569653; doi:10.3390/ijms231911014)
Supplement: Supplementary file 1 [file ijms-23-11014-s001.zip › Supplementary Table S3.pdf]

Table S3. Differentially expressed genes of M2c like TAMs between dMMR and pMMR

Gene	Group	avg_log2FC	pct.1	pct.2	p_val	p_val_adj
REG1A	highly expressed in dMMR	1.504616	0.313	0.074	2.94E-38	4.28E-34
CCL2	highly expressed in dMMR	1.181042	0.435	0.252	2.47E-16	3.59E-12
CCL18	highly expressed in dMMR	1.160743	0.597	0.372	1.31E-26	1.91E-22
CXCL8	highly expressed in dMMR	1.082777	0.829	0.584	2.16E-31	3.15E-27
OLR1	highly expressed in dMMR	0.983492	0.619	0.309	3.43E-42	4.99E-38
IL7R	highly expressed in dMMR	0.976687	0.696	0.426	2.32E-31	3.38E-27
S100A8	highly expressed in dMMR	0.973108	0.534	0.252	7.74E-32	1.13E-27
PLAU	highly expressed in dMMR	0.909405	0.837	0.606	1.24E-41	1.81E-37
SPP1	highly expressed in dMMR	0.863922	0.83	0.543	3.06E-35	4.45E-31
APOC1	highly expressed in dMMR	0.81162	0.817	0.706	7.53E-11	1.10E-06
SOD2	highly expressed in dMMR	0.805976	0.962	0.867	6.71E-41	9.76E-37
APOE	highly expressed in dMMR	0.784417	0.867	0.752	1.92E-15	2.79E-11
S100A6	highly expressed in dMMR	0.756131	0.979	0.897	2.52E-41	3.67E-37
HTRA1	highly expressed in dMMR	0.716061	0.408	0.201	5.45E-21	7.93E-17
HIF1A	highly expressed in dMMR	0.702375	0.971	0.894	7.39E-46	1.08E-41
C15orf48	highly expressed in dMMR	0.699376	0.743	0.484	1.44E-28	2.10E-24
FTH1	highly expressed in dMMR	0.677829	1	1	1.71E-40	2.48E-36
VCAN	highly expressed in dMMR	0.661628	0.355	0.152	1.50E-20	2.19E-16
GPNMB	highly expressed in dMMR	0.659852	0.94	0.846	1.37E-26	1.99E-22
NUPR1	highly expressed in dMMR	0.657502	0.545	0.368	3.69E-16	5.37E-12
CXCL3	highly expressed in dMMR	0.634533	0.664	0.41	9.33E-25	1.36E-20
PLAUR	highly expressed in dMMR	0.633271	0.907	0.764	6.86E-26	9.98E-22
GPX3	highly expressed in dMMR	0.629167	0.682	0.425	3.21E-31	4.67E-27
CTSB	highly expressed in dMMR	0.619374	1	1	5.22E-50	7.60E-46
S100A9	highly expressed in dMMR	0.609795	0.916	0.765	1.96E-22	2.85E-18
CD44	highly expressed in dMMR	0.601816	0.96	0.91	4.05E-32	5.89E-28
ADAM8	highly expressed in dMMR	0.600123	0.623	0.366	4.50E-27	6.54E-23
CXCL1	highly expressed in dMMR	0.597879	0.394	0.217	2.35E-15	3.42E-11
CXCL2	highly expressed in dMMR	0.580382	0.668	0.462	2.31E-19	3.36E-15
FCGR2A	highly expressed in dMMR	0.578303	0.996	0.972	4.85E-33	7.06E-29
SDC2	highly expressed in dMMR	0.571909	0.576	0.304	1.25E-27	1.82E-23
VIM	highly expressed in dMMR	0.570308	0.987	0.964	6.31E-39	9.18E-35
GLUL	highly expressed in dMMR	0.56798	1	0.989	2.42E-38	3.52E-34
S100A4	highly expressed in dMMR	0.559583	0.926	0.811	3.27E-23	4.76E-19
S100A10	highly expressed in dMMR	0.55089	0.977	0.918	2.98E-35	4.34E-31
SPARC	highly expressed in dMMR	0.549722	0.313	0.151	4.28E-16	6.22E-12
CLEC5A	highly expressed in dMMR	0.533115	0.455	0.263	1.88E-16	2.73E-12
FLNA	highly expressed in dMMR	0.531678	0.704	0.557	1.07E-18	1.55E-14
AQP9	highly expressed in dMMR	0.531005	0.415	0.134	1.06E-36	1.54E-32
CSTB	highly expressed in dMMR	0.526671	0.991	0.946	1.42E-25	2.06E-21

FCGR3A	highly expressed in dMMR	0.513674	0.963	0.912	8.60E-23	1.25E-18
CD9	highly expressed in dMMR	0.510254	0.881	0.75	1.51E-18	2.19E-14
TGFBI	highly expressed in dMMR	0.493262	0.983	0.97	1.12E-14	1.63E-10
HMOX1	highly expressed in dMMR	0.488776	0.846	0.748	3.77E-10	5.49E-06
SLC16A10	highly expressed in dMMR	0.482161	0.721	0.489	5.03E-21	7.32E-17
LITAF	highly expressed in dMMR	0.480484	0.985	0.947	3.04E-28	4.42E-24
SLC11A1	highly expressed in dMMR	0.475317	0.747	0.531	3.29E-22	4.78E-18
THBD	highly expressed in dMMR	0.471015	0.674	0.507	1.05E-13	1.53E-09
RASSF4	highly expressed in dMMR	0.468193	0.882	0.742	1.49E-23	2.17E-19
CTSD	highly expressed in dMMR	0.467589	0.997	0.996	1.77E-13	2.57E-09
CTSL	highly expressed in dMMR	0.460617	0.971	0.942	5.83E-18	8.48E-14
LHFPL2	highly expressed in dMMR	0.45467	0.867	0.743	4.76E-22	6.93E-18
TGM2	highly expressed in dMMR	0.443407	0.488	0.305	8.55E-15	1.24E-10
ITGAX	highly expressed in dMMR	0.44152	0.755	0.548	1.91E-20	2.78E-16
MMP14	highly expressed in dMMR	0.435873	0.854	0.721	3.51E-21	5.11E-17
PAPSS1	highly expressed in dMMR	0.43431	0.777	0.615	6.90E-20	1.00E-15
GCHFR	highly expressed in dMMR	0.432701	0.443	0.28	1.93E-12	2.80E-08
IL1B	highly expressed in dMMR	0.429219	0.452	0.293	1.72E-09	2.50E-05
RAP2B	highly expressed in dMMR	0.42793	0.922	0.802	2.24E-23	3.25E-19
LTA4H	highly expressed in dMMR	0.410573	0.65	0.432	1.17E-20	1.70E-16
MXD1	highly expressed in dMMR	0.408793	0.781	0.609	5.02E-15	7.31E-11
BCAT1	highly expressed in dMMR	0.407272	0.785	0.63	3.44E-19	5.00E-15
FBP1	highly expressed in dMMR	0.401202	0.524	0.394	5.35E-11	7.78E-07
BMP2K	highly expressed in dMMR	0.401045	0.924	0.834	5.59E-20	8.13E-16
ASPH	highly expressed in dMMR	0.39901	0.696	0.532	2.88E-16	4.19E-12
OSCAR	highly expressed in dMMR	0.398047	0.528	0.292	4.15E-24	6.04E-20
ANPEP	highly expressed in dMMR	0.395128	0.428	0.246	2.46E-15	3.57E-11
GK	highly expressed in dMMR	0.389324	0.656	0.444	6.24E-18	9.08E-14
ERRFI1	highly expressed in dMMR	0.383793	0.402	0.215	5.11E-16	7.43E-12
PEA15	highly expressed in dMMR	0.383532	0.792	0.676	4.02E-14	5.85E-10
ADAM9	highly expressed in dMMR	0.383166	0.886	0.778	3.58E-20	5.21E-16
HS3ST1	highly expressed in dMMR	0.380518	0.419	0.245	3.06E-15	4.45E-11
NPC1	highly expressed in dMMR	0.375381	0.641	0.47	7.54E-16	1.10E-11
TNS3	highly expressed in dMMR	0.375303	0.768	0.634	1.62E-15	2.35E-11
ANXA2	highly expressed in dMMR	0.373577	0.976	0.917	1.12E-21	1.62E-17
RALA	highly expressed in dMMR	0.37083	0.702	0.527	3.59E-15	5.23E-11
SLC39A8	highly expressed in dMMR	0.367256	0.554	0.38	1.70E-12	2.47E-08
FNDC3B	highly expressed in dMMR	0.366791	0.79	0.668	1.90E-12	2.76E-08
ACTN1	highly expressed in dMMR	0.362	0.788	0.627	9.46E-16	1.38E-11
TSPO	highly expressed in dMMR	0.36195	0.938	0.836	5.19E-17	7.55E-13
SCD	highly expressed in dMMR	0.359503	0.686	0.522	9.77E-12	1.42E-07

ELL2	highly expressed in dMMR	0.358827	0.629	0.461	3.54E-13	5.16E-09
LAIR1	highly expressed in dMMR	0.354877	0.898	0.796	8.55E-19	1.24E-14
SLC2A3	highly expressed in dMMR	0.353713	0.751	0.638	8.73E-10	1.27E-05
IGF2R	highly expressed in dMMR	0.349527	0.741	0.574	1.50E-16	2.18E-12
PLXNC1	highly expressed in dMMR	0.349473	0.857	0.686	1.20E-16	1.75E-12
SERPINA1	highly expressed in dMMR	0.348022	0.85	0.726	8.43E-14	1.23E-09
CAPG	highly expressed in dMMR	0.347929	0.963	0.808	8.80E-15	1.28E-10
ALDH2	highly expressed in dMMR	0.345439	0.727	0.557	2.00E-13	2.92E-09
COLEC12	highly expressed in dMMR	0.340737	0.549	0.286	1.87E-22	2.72E-18
CLIC4	highly expressed in dMMR	0.338985	0.611	0.443	5.09E-15	7.40E-11
TUBA1A	highly expressed in dMMR	0.338456	0.788	0.686	1.32E-06	0.019232
LAMP1	highly expressed in dMMR	0.337101	0.98	0.954	1.60E-15	2.32E-11
ATP2C1	highly expressed in dMMR	0.335105	0.747	0.569	4.60E-18	6.69E-14
MSN	highly expressed in dMMR	0.334355	0.96	0.912	7.22E-18	1.05E-13
SEMA4A	highly expressed in dMMR	0.327494	0.568	0.391	3.63E-14	5.29E-10
TREM1	highly expressed in dMMR	0.327409	0.444	0.283	4.40E-12	6.40E-08
ZFP36L1	highly expressed in dMMR	0.327094	0.975	0.967	1.73E-13	2.52E-09
APP	highly expressed in dMMR	0.32674	0.712	0.54	3.08E-14	4.48E-10
CD109	highly expressed in dMMR	0.324676	0.391	0.204	4.96E-17	7.22E-13
LSP1	highly expressed in dMMR	0.323826	0.735	0.6	4.95E-11	7.21E-07
GNS	highly expressed in dMMR	0.321046	0.955	0.905	2.54E-19	3.69E-15
GLIPR2	highly expressed in dMMR	0.318031	0.557	0.363	5.84E-15	8.50E-11
PDPN	highly expressed in dMMR	0.314461	0.405	0.22	9.86E-16	1.43E-11
TMEM51	highly expressed in dMMR	0.30995	0.653	0.483	1.48E-13	2.15E-09
FTL	highly expressed in dMMR	0.308638	1	1	1.03E-16	1.50E-12
VASP	highly expressed in dMMR	0.30586	0.808	0.703	6.64E-11	9.66E-07
TUBA1C	highly expressed in dMMR	0.305763	0.794	0.688	3.73E-10	5.43E-06
RAB32	highly expressed in dMMR	0.304227	0.822	0.706	6.13E-10	8.92E-06
MYO1G	highly expressed in dMMR	0.303693	0.504	0.334	3.64E-13	5.29E-09
ALCAM	highly expressed in dMMR	0.302431	0.749	0.628	1.25E-09	1.83E-05
GM2A	highly expressed in dMMR	0.3022	0.887	0.773	2.03E-12	2.96E-08
FAM129A	highly expressed in dMMR	0.301471	0.546	0.384	8.04E-12	1.17E-07
TDP2	highly expressed in dMMR	0.301409	0.549	0.397	7.71E-11	1.12E-06
TXNIP	highly expressed in dMMR	0.300974	0.981	0.954	6.41E-09	9.33E-05
ATP13A3	highly expressed in dMMR	0.300385	0.794	0.656	9.35E-11	1.36E-06
VAT1	highly expressed in dMMR	0.298557	0.622	0.461	7.57E-12	1.10E-07
SLC16A3	highly expressed in dMMR	0.298238	0.899	0.767	5.46E-12	7.95E-08
EPB41L3	highly expressed in dMMR	0.298063	0.862	0.748	5.38E-13	7.83E-09
CADM1	highly expressed in dMMR	0.29682	0.367	0.257	3.47E-07	0.005051
PKM	highly expressed in dMMR	0.295125	0.939	0.844	4.39E-13	6.39E-09
ALOX5AP	highly expressed in dMMR	0.294485	0.745	0.624	4.31E-07	0.006267

SOCS3	highly expressed in dMMR	0.293327	0.838	0.739	1.23E-07	0.001783
BTG1	highly expressed in dMMR	0.292505	0.942	0.883	5.64E-08	0.00082
GSN	highly expressed in dMMR	0.29149	0.93	0.871	1.46E-10	2.12E-06
PLP2	highly expressed in dMMR	0.289008	0.499	0.308	8.14E-15	1.18E-10
NDRG1	highly expressed in dMMR	0.285819	0.504	0.316	4.59E-14	6.68E-10
FAM129B	highly expressed in dMMR	0.283998	0.463	0.287	4.01E-13	5.83E-09
HLA-DQA1	highly expressed in dMMR	0.280722	0.995	0.986	1.54E-07	0.002243
ISCU	highly expressed in dMMR	0.280597	0.798	0.684	7.27E-11	1.06E-06
ICAM1	highly expressed in dMMR	0.279854	0.747	0.648	4.01E-07	0.005829
CORO1C	highly expressed in dMMR	0.278002	0.801	0.662	1.36E-11	1.98E-07
CAB39	highly expressed in dMMR	0.276114	0.767	0.634	3.57E-12	5.19E-08
SLC43A3	highly expressed in dMMR	0.275859	0.696	0.573	3.03E-09	4.41E-05
PILRA	highly expressed in dMMR	0.274523	0.759	0.644	9.85E-10	1.43E-05
SGK1	highly expressed in dMMR	0.274518	0.95	0.867	2.46E-09	3.58E-05
CD83	highly expressed in dMMR	0.274092	0.836	0.736	9.03E-08	0.001314
DSE	highly expressed in dMMR	0.272758	0.879	0.776	6.89E-11	1.00E-06
LAPTM5	highly expressed in dMMR	0.270568	1	0.992	2.05E-16	2.98E-12
PLXDC2	highly expressed in dMMR	0.270566	0.919	0.852	7.64E-11	1.11E-06
NEK6	highly expressed in dMMR	0.269491	0.527	0.356	6.42E-13	9.34E-09
TANK	highly expressed in dMMR	0.269416	0.668	0.52	2.45E-10	3.56E-06
DNASE2	highly expressed in dMMR	0.268934	0.683	0.581	6.21E-07	0.009037
CERS2	highly expressed in dMMR	0.259907	0.582	0.413	3.39E-13	4.93E-09
SDCBP	highly expressed in dMMR	0.257984	0.996	0.975	1.33E-15	1.93E-11
SH3BGRL3	highly expressed in dMMR	0.257931	0.993	0.97	1.30E-13	1.89E-09
CD151	highly expressed in dMMR	0.256559	0.634	0.469	2.90E-12	4.22E-08
ALOX5	highly expressed in dMMR	0.256003	0.72	0.566	4.82E-11	7.02E-07
HLA-DQB1	highly expressed in dMMR	0.2555	0.996	0.988	4.85E-11	7.06E-07
CXCL16	highly expressed in dMMR	0.2549	0.939	0.929	1.75E-06	0.025395
UGP2	highly expressed in dMMR	0.25387	0.747	0.669	5.97E-07	0.008681
SPRED1	highly expressed in dMMR	0.252715	0.854	0.836	1.78E-06	0.025913
FPR3	highly expressed in dMMR	0.250249	0.966	0.934	1.27E-11	1.85E-07
UCP2	highly expressed in dMMR	0.250203	0.881	0.824	3.49E-07	0.005074
BTF3	highly expressed in pMMR	-0.25209	0.955	0.952	3.85E-09	5.61E-05
CTSH	highly expressed in pMMR	-0.25371	0.911	0.899	8.83E-08	0.001285
NUDT16	highly expressed in pMMR	-0.25438	0.456	0.538	2.03E-07	0.002959
HSP90B1	highly expressed in pMMR	-0.25648	0.975	0.977	1.51E-08	0.00022
EEF1B2	highly expressed in pMMR	-0.25849	0.95	0.961	4.70E-09	6.84E-05
NDUFA13	highly expressed in pMMR	-0.25873	0.775	0.811	4.13E-09	6.01E-05
ITPR2	highly expressed in pMMR	-0.25935	0.824	0.847	3.33E-06	0.048475
NDUFA4	highly expressed in pMMR	-0.25989	0.958	0.957	4.39E-12	6.39E-08
PRKCB	highly expressed in pMMR	-0.2625	0.34	0.45	3.14E-07	0.004574

CIRBP	highly expressed in pMMR	-0.26359	0.842	0.855	1.23E-06	0.017923
BLVRB	highly expressed in pMMR	-0.26543	0.822	0.848	1.25E-07	0.001821
MLEC	highly expressed in pMMR	-0.26822	0.656	0.707	5.54E-08	0.000806
PTEN	highly expressed in pMMR	-0.26835	0.695	0.76	7.42E-08	0.00108
CYBA	highly expressed in pMMR	-0.26852	1	1	1.38E-19	2.00E-15
SSB	highly expressed in pMMR	-0.26963	0.731	0.76	1.61E-08	0.000234
PSMA7	highly expressed in pMMR	-0.27003	0.923	0.929	2.94E-11	4.28E-07
CEBPB	highly expressed in pMMR	-0.27063	0.967	0.978	1.34E-07	0.001953
TIMM10	highly expressed in pMMR	-0.27308	0.362	0.47	1.13E-08	0.000164
LDHB	highly expressed in pMMR	-0.27331	0.723	0.76	1.79E-07	0.002602
REL	highly expressed in pMMR	-0.27393	0.779	0.84	1.61E-08	0.000234
CSF1R	highly expressed in pMMR	-0.27434	0.947	0.964	1.83E-08	0.000266
NDUFA12	highly expressed in pMMR	-0.27489	0.626	0.704	2.74E-10	3.99E-06
HSPD1	highly expressed in pMMR	-0.27738	0.777	0.824	1.65E-10	2.40E-06
CCND1	highly expressed in pMMR	-0.2791	0.272	0.385	1.24E-07	0.001798
KCNMA1	highly expressed in pMMR	-0.27944	0.491	0.575	2.76E-06	0.040168
SASH1	highly expressed in pMMR	-0.27983	0.452	0.546	1.06E-06	0.015439
RGS10	highly expressed in pMMR	-0.28136	0.91	0.905	1.60E-12	2.33E-08
CAT	highly expressed in pMMR	-0.28519	0.718	0.79	2.49E-10	3.63E-06
PRELID1	highly expressed in pMMR	-0.29363	0.772	0.771	1.10E-06	0.01604
AIF1	highly expressed in pMMR	-0.29727	0.993	0.992	2.22E-12	3.23E-08
RGL1	highly expressed in pMMR	-0.301	0.598	0.682	6.09E-07	0.008856
RNASE6	highly expressed in pMMR	-0.30356	0.912	0.941	1.57E-10	2.29E-06
CD300A	highly expressed in pMMR	-0.304	0.606	0.682	7.82E-08	0.001138
TRA2B	highly expressed in pMMR	-0.30485	0.797	0.832	2.79E-07	0.004055
MGST2	highly expressed in pMMR	-0.30629	0.653	0.75	1.09E-11	1.59E-07
SSR4	highly expressed in pMMR	-0.30914	0.915	0.922	3.21E-12	4.67E-08
LGALS3BP	highly expressed in pMMR	-0.31321	0.59	0.677	2.21E-08	0.000321
SPTLC2	highly expressed in pMMR	-0.31324	0.324	0.439	2.54E-09	3.69E-05
SESN1	highly expressed in pMMR	-0.31531	0.293	0.392	9.58E-07	0.013937
HLA-DPB1	highly expressed in pMMR	-0.31568	1	1	5.68E-16	8.26E-12
TNFRSF21	highly expressed in pMMR	-0.3167	0.484	0.589	6.38E-09	9.28E-05
NCF4	highly expressed in pMMR	-0.31839	0.598	0.697	6.63E-12	9.65E-08
FGD2	highly expressed in pMMR	-0.31843	0.476	0.579	6.09E-09	8.86E-05
ARL4C	highly expressed in pMMR	-0.31858	0.907	0.911	1.12E-08	0.000163
CD93	highly expressed in pMMR	-0.31864	0.736	0.817	2.73E-08	0.000398
NCF1	highly expressed in pMMR	-0.31968	0.52	0.663	1.74E-12	2.53E-08
WASF2	highly expressed in pMMR	-0.32468	0.78	0.838	3.94E-10	5.73E-06
SLA	highly expressed in pMMR	-0.32933	0.82	0.852	9.83E-13	1.43E-08
SLC9A9	highly expressed in pMMR	-0.33299	0.24	0.364	8.20E-10	1.19E-05
MAFB	highly expressed in pMMR	-0.33474	0.973	0.975	6.53E-07	0.009498

C1orf54	highly expressed in pMMR	-0.33826	0.641	0.719	7.24E-09	0.000105
MAT2A	highly expressed in pMMR	-0.34234	0.775	0.811	1.26E-07	0.001827
GPR82	highly expressed in pMMR	-0.34414	0.158	0.302	1.65E-12	2.41E-08
AKAP9	highly expressed in pMMR	-0.34987	0.647	0.724	1.99E-11	2.90E-07
GPR183	highly expressed in pMMR	-0.36344	0.863	0.901	4.24E-10	6.17E-06
AOAH	highly expressed in pMMR	-0.3636	0.329	0.49	1.25E-13	1.82E-09
GPR34	highly expressed in pMMR	-0.36379	0.8	0.877	7.92E-13	1.15E-08
BST2	highly expressed in pMMR	-0.36477	0.893	0.907	3.05E-14	4.44E-10
AKAP13	highly expressed in pMMR	-0.36541	0.829	0.867	1.34E-11	1.95E-07
MPEG1	highly expressed in pMMR	-0.36579	0.94	0.963	6.86E-12	9.99E-08
PPIB	highly expressed in pMMR	-0.36588	0.871	0.878	5.73E-12	8.33E-08
ADORA3	highly expressed in pMMR	-0.377	0.277	0.421	4.90E-12	7.14E-08
CD302	highly expressed in pMMR	-0.37746	0.647	0.739	1.37E-09	1.99E-05
PSME2	highly expressed in pMMR	-0.38174	0.794	0.801	2.37E-08	0.000345
HSP90AA1	highly expressed in pMMR	-0.382	0.999	0.996	1.97E-20	2.87E-16
CTSC	highly expressed in pMMR	-0.38591	0.993	0.993	6.11E-15	8.89E-11
GATM	highly expressed in pMMR	-0.38722	0.452	0.602	3.68E-14	5.35E-10
SRGAP1	highly expressed in pMMR	-0.39026	0.194	0.335	1.81E-12	2.64E-08
TPT1	highly expressed in pMMR	-0.39832	1	1	2.31E-20	3.36E-16
UTRN	highly expressed in pMMR	-0.40493	0.585	0.647	1.54E-10	2.24E-06
FAM26F	highly expressed in pMMR	-0.40698	0.422	0.589	7.21E-15	1.05E-10
SMAP2	highly expressed in pMMR	-0.40719	0.741	0.836	1.03E-13	1.50E-09
STK17B	highly expressed in pMMR	-0.41444	0.573	0.661	7.31E-10	1.06E-05
SIGLEC1	highly expressed in pMMR	-0.42054	0.472	0.6	4.57E-12	6.66E-08
CEBPD	highly expressed in pMMR	-0.42056	0.894	0.954	8.10E-18	1.18E-13
A2M	highly expressed in pMMR	-0.42091	0.861	0.899	1.38E-09	2.01E-05
ALDH1A1	highly expressed in pMMR	-0.4257	0.253	0.376	7.81E-09	0.000114
DAB2	highly expressed in pMMR	-0.43053	0.975	0.978	3.75E-07	0.005454
IGSF6	highly expressed in pMMR	-0.43157	0.847	0.853	5.31E-10	7.73E-06
SIAH2	highly expressed in pMMR	-0.43457	0.33	0.485	5.83E-15	8.48E-11
HCST	highly expressed in pMMR	-0.43535	0.808	0.867	1.64E-10	2.38E-06
MCL1	highly expressed in pMMR	-0.44181	0.984	0.986	1.95E-14	2.84E-10
VSIG4	highly expressed in pMMR	-0.44285	0.719	0.758	7.08E-09	0.000103
ITM2C	highly expressed in pMMR	-0.44927	0.154	0.324	1.66E-17	2.42E-13
SPATS2L	highly expressed in pMMR	-0.45729	0.56	0.665	3.95E-16	5.75E-12
FILIP1L	highly expressed in pMMR	-0.46335	0.285	0.448	8.66E-14	1.26E-09
C1QC	highly expressed in pMMR	-0.47285	0.975	0.984	1.97E-33	2.87E-29
ENPP2	highly expressed in pMMR	-0.47518	0.46	0.682	1.73E-21	2.51E-17
MS4A4A	highly expressed in pMMR	-0.4847	0.943	0.951	2.13E-13	3.11E-09
HLA-DPA1	highly expressed in pMMR	-0.48971	1	0.999	6.31E-23	9.18E-19
MS4A6A	highly expressed in pMMR	-0.49298	0.987	0.992	5.53E-28	8.05E-24

DDX3Y	highly expressed in pMMR	-0.49844	0.194	0.372	3.75E-16	5.45E-12
FKBP5	highly expressed in pMMR	-0.50372	0.666	0.797	4.89E-21	7.12E-17
STAB1	highly expressed in pMMR	-0.50408	0.95	0.97	9.53E-13	1.39E-08
FCGRT	highly expressed in pMMR	-0.51682	0.989	0.989	2.50E-31	3.64E-27
LTC4S	highly expressed in pMMR	-0.51852	0.194	0.376	2.24E-18	3.26E-14
CD209	highly expressed in pMMR	-0.51878	0.588	0.679	2.67E-10	3.89E-06
CPVL	highly expressed in pMMR	-0.52946	0.861	0.894	4.67E-14	6.79E-10
SAP30	highly expressed in pMMR	-0.53725	0.405	0.584	1.43E-19	2.08E-15
ZFP36L2	highly expressed in pMMR	-0.54129	0.915	0.94	8.30E-15	1.21E-10
WLS	highly expressed in pMMR	-0.54346	0.19	0.407	1.30E-24	1.89E-20
KLF9	highly expressed in pMMR	-0.55365	0.436	0.591	1.42E-16	2.06E-12
MS4A7	highly expressed in pMMR	-0.57558	0.991	0.993	1.84E-23	2.67E-19
C1QB	highly expressed in pMMR	-0.58525	0.971	0.983	3.54E-41	5.16E-37
HLA-DRB5	highly expressed in pMMR	-0.58786	0.595	0.86	3.23E-33	4.69E-29
ITM2B	highly expressed in pMMR	-0.58865	1	1	3.33E-55	4.85E-51
CLEC10A	highly expressed in pMMR	-0.59636	0.228	0.353	8.31E-11	1.21E-06
AMICA1	highly expressed in pMMR	-0.59809	0.252	0.444	6.10E-19	8.88E-15
P2RY13	highly expressed in pMMR	-0.61038	0.288	0.511	8.75E-23	1.27E-18
LY6E	highly expressed in pMMR	-0.62665	0.553	0.613	9.17E-08	0.001334
HERPUD1	highly expressed in pMMR	-0.64583	0.93	0.977	1.07E-28	1.56E-24
TSC22D3	highly expressed in pMMR	-0.64962	0.891	0.963	4.11E-31	5.99E-27
LILRB5	highly expressed in pMMR	-0.65562	0.424	0.51	7.06E-10	1.03E-05
C1QA	highly expressed in pMMR	-0.66371	0.984	0.988	6.96E-53	1.01E-48
IFI44L	highly expressed in pMMR	-0.67551	0.247	0.367	5.29E-10	7.70E-06
CD163L1	highly expressed in pMMR	-0.67622	0.284	0.462	6.62E-19	9.63E-15
IFITM3	highly expressed in pMMR	-0.68398	0.818	0.871	6.27E-25	9.12E-21
HLA-DQA2	highly expressed in pMMR	-0.79929	0.264	0.527	2.89E-28	4.20E-24
AXL	highly expressed in pMMR	-0.82065	0.405	0.718	7.78E-48	1.13E-43
FUCA1	highly expressed in pMMR	-0.85887	0.725	0.82	1.17E-18	1.71E-14
IGF1	highly expressed in pMMR	-0.87339	0.15	0.397	1.44E-29	2.10E-25
FGL2	highly expressed in pMMR	-0.90232	0.902	0.969	3.73E-40	5.42E-36
SLC40A1	highly expressed in pMMR	-1.01194	0.728	0.887	1.58E-29	2.29E-25
FOLR2	highly expressed in pMMR	-1.03459	0.529	0.671	1.45E-20	2.12E-16
CST3	highly expressed in pMMR	-1.0723	0.999	1	2.63E-93	3.83E-89
IFI27	highly expressed in pMMR	-1.08299	0.22	0.318	3.15E-07	0.004579
ISG15	highly expressed in pMMR	-1.12868	0.578	0.628	4.23E-10	6.15E-06
THBS1	highly expressed in pMMR	-1.20752	0.24	0.414	1.94E-16	2.82E-12
SEPP1	highly expressed in pMMR	-1.43021	0.584	0.756	9.98E-27	1.45E-22
PDK4	highly expressed in pMMR	-1.59266	0.427	0.655	1.57E-37	2.28E-33
