# Supplementary figures and images for: Infiltration of Apoptotic M2 Macrophage Subpopulation Is Negatively Correlated with the Immunotherapy Response in Colorectal Cancer
Source: Int J Mol Sci. 2022 Sep 20;23(19):11014. doi: 10.3390/ijms231911014 (PMC9569653; doi:10.3390/ijms231911014)

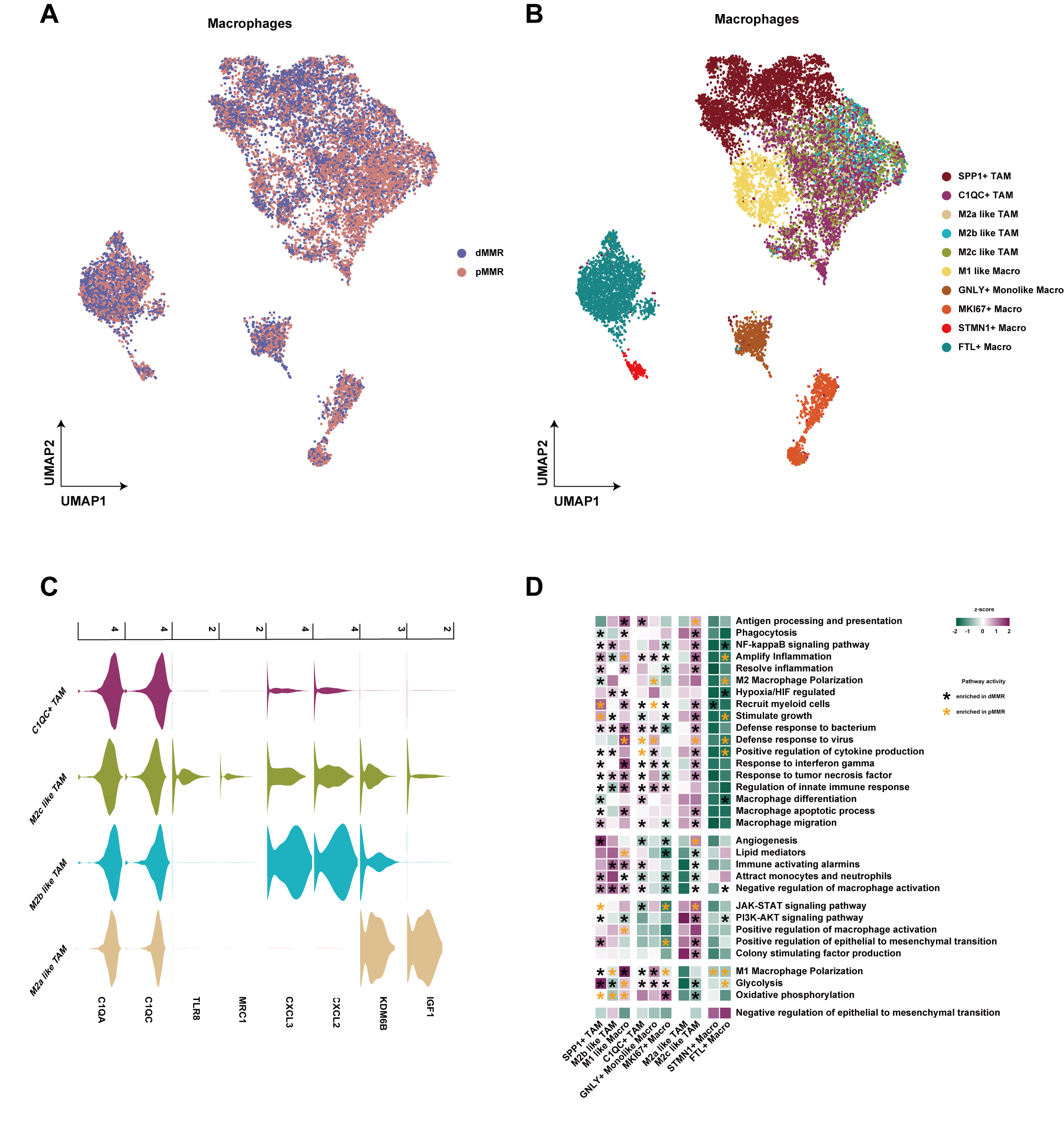

Supplement: Supplementary file 1 [file ijms-23-11014-s001.zip › Supplementary Figure S1.tif]

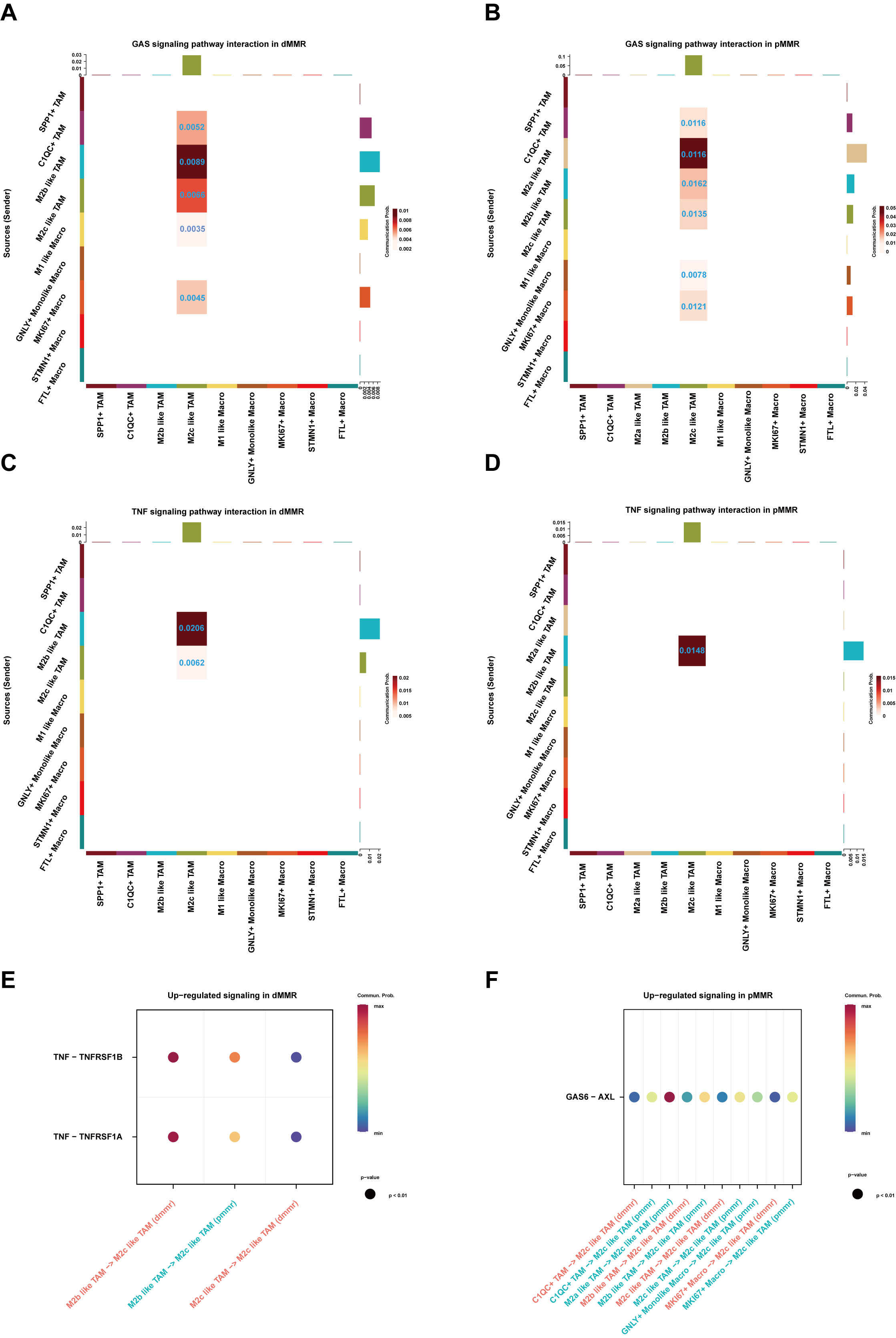

Supplement: Supplementary file 1 [file ijms-23-11014-s001.zip › Supplementary Figure S2.tif]

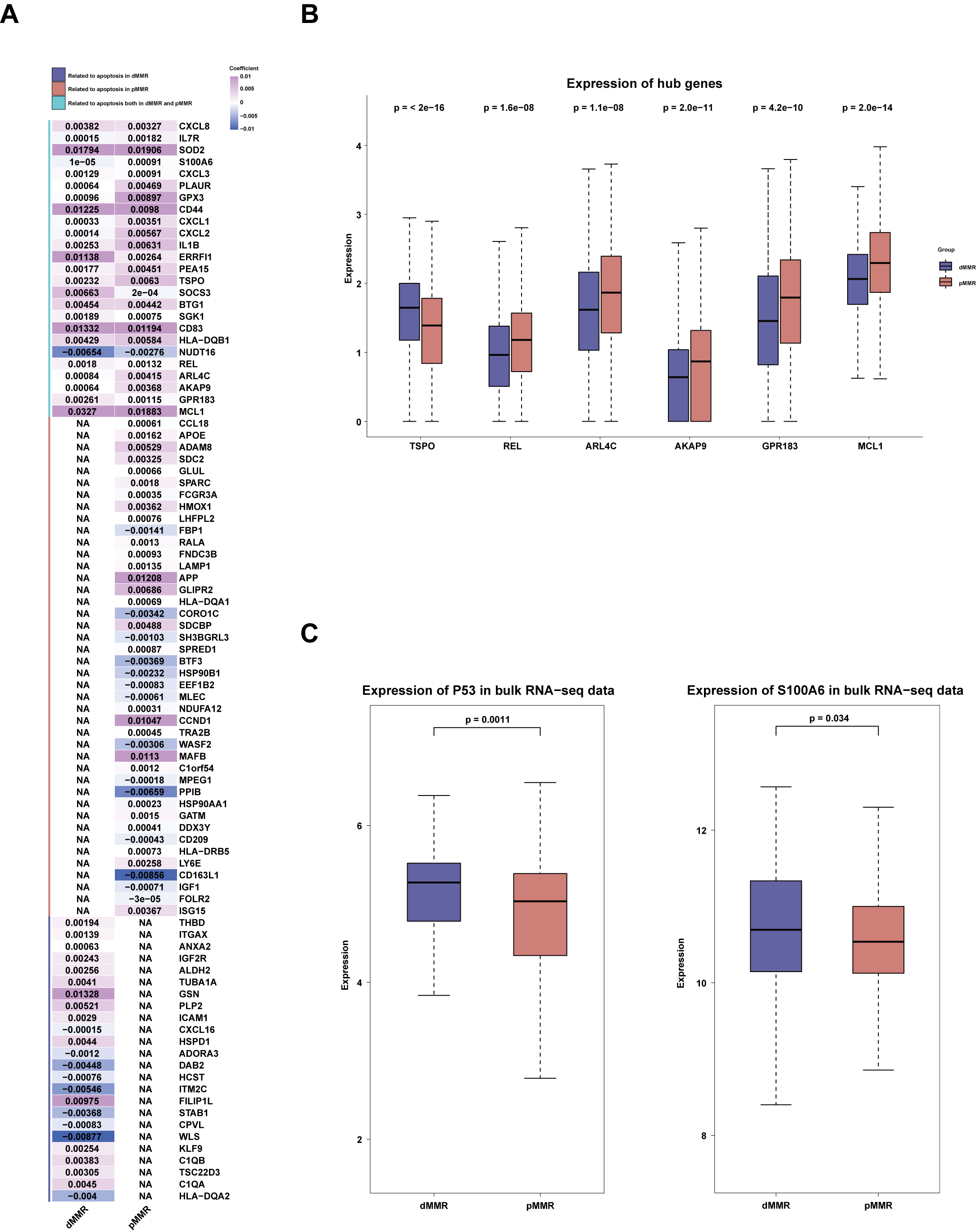

Supplement: Supplementary file 1 [file ijms-23-11014-s001.zip › Supplementary Figure S3.tif]

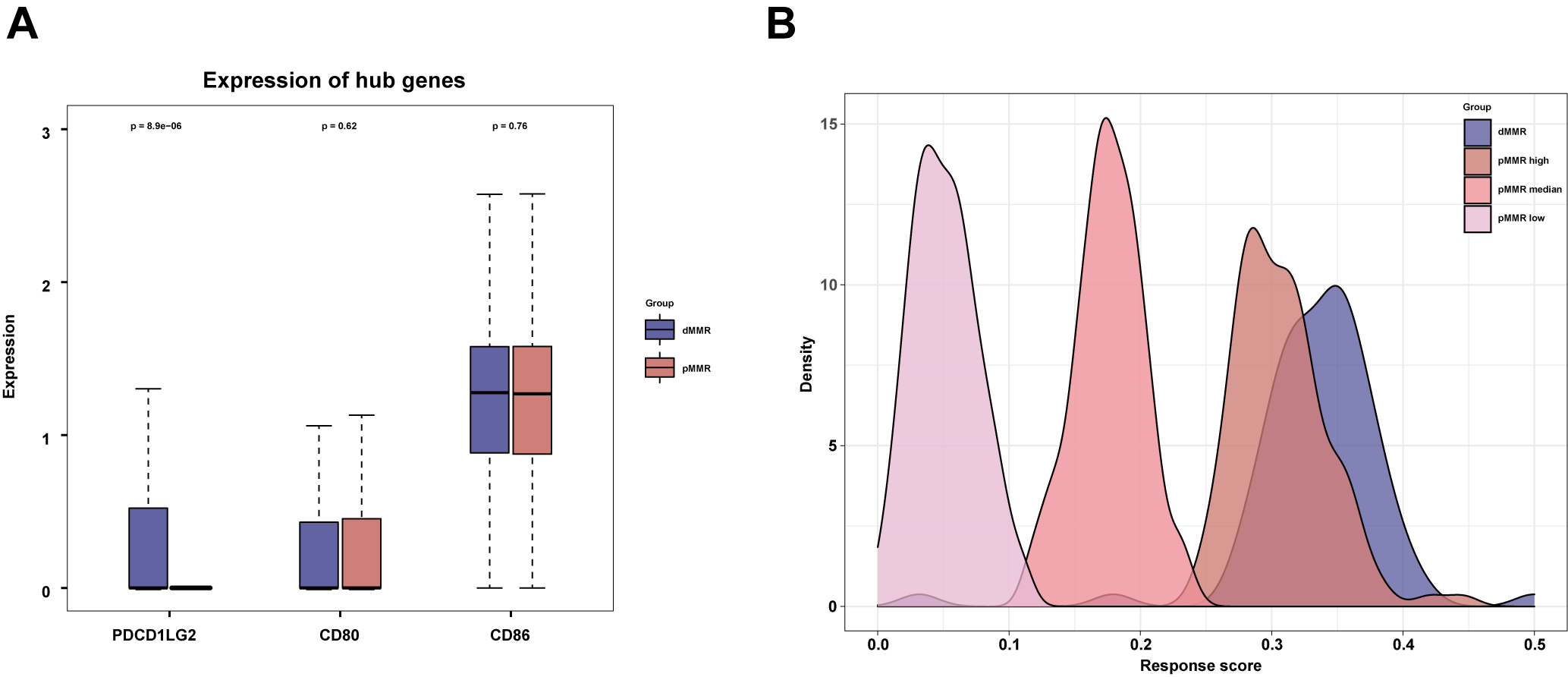

Supplement: Supplementary file 1 [file ijms-23-11014-s001.zip › Supplementary Figure S4.tif]
